# Supplementary material for: Phase transfer catalysts shift the pathway to transmetalation in biphasic Suzuki-Miyaura cross-couplings
Source: Nat Commun. 2024 Jun 27;15:5436. doi: 10.1038/s41467-024-49681-4 (PMC11211432; doi:10.1038/s41467-024-49681-4)
Supplement: Supplementary file 1 — Supplementary Information [file 41467_2024_49681_MOESM1_ESM.pdf]

SUPPLEMENTARY INFORMATION

Phase Transfer Catalysts Shift the Pathway to Transmetalation in Biphasic Suzuki-Miyaura Cross-Couplings

Yao Shi[1], Joshua S. Derasp[1], Tristan Maschmeyer[1] and *Jason E. Hein[1,2,3]*

[1] Department of Chemistry, University of British Columbia, Vancouver, British Columbia, V6T 1Z3, Canada
[2] Department of Chemistry, University of Bergen, Bergen, Norway
[3] Acceleration Consortium, University of Toronto, Toronto, ON, Canada

[*] E-mail: jderasp@chem.ubc.ca; jhein@chem.ubc.ca

General Information 2

Automated Sampling Platforms and Parameters 3

Conversion of Peak Area to Concentration 4

Experimental Details 5

Study of Speciation of Organoboron Nucleophile 24

Study of Speciation of Palladium Catalyst 34

Reaction Scope 40

Spectra 53

Supplementary References 94

General Information

Materials and reagents: All commercial reagents were purchased from Sigma-Aldrich, Fisher Scientific, Alfa Aesar, TCI America, Oakwood Chemical, Combi-Blocks or Strem Chemicals and used without purification except for the following: Anhydrous and air free 2-Me-THF was purchased from Millipore Sigma and stored over activated molecular sieves in a flame-dried Schlenk flask. Water used in coupling reactions was deionized and degassed by freeze pump thaw to remove dissolved oxygen.

Analytical equipment and methods: NMR spectra were recorded on either a Bruker Avance 300 (^{1}H 300 MHz, ^{13}C 75 MHz), Bruker Avance 400 (^{1}H 400 MHz, ^{13}C 101 MHz, ^{11}B MHz, ^{19}F MHz, ^{31}P MHz) NMR spectrometer, or a Bruker Avance 500 (^{1}H 400 MHz, ^{19}F MHz, ^{31}P MHz) NMR spectrometer. Multiplicity was reported using the following abbreviations: br = broad, s = singlet, d = doublet, t = triplet, q = quartet, quint. = quintet, sext. = sextuplet, sept = septuplet, and m = multiplet. Coupling constants are reported in Hertz (Hz).
Visualization of TLC plates was performed by UV lamp (λ = 254 nm), and/or $KMnO_4$ staining. Eluent solvent systems reported as volume/volume. Normal phase column chromatography was performed using SiliCycle silica gel (40-63 µm, 230-400 mesh). When chromatography was performed in an automated manner, a BUCHI Pure C-810 Flash Chromatography system was used with Redisep Rf Gold C18 reversed phase column (20-40 micron). Analysis *via* HPLC was conducted using an Agilent 1200 HPLC equipped with: an Agilent G1379B degasser, G4220A binary pump, G4226A autosampler, G1316C thermal column compartment, G4212A diode array detector. One HPLC method was used (see below for detailed description of the HPLC methods). Data processing and analysis was carried out using ChemStation (Agilent) software and another proprietary third-party software.

Method:

Column:	Poroshell 120 EC-C18 2.7 µm, 2.1 x 50 mm	
Column Temperature:	30 °C	
Flow Rate:	0.650 mL/min	
Detection:	230 nm	
Acquisition Time:	10 min	
Mobile Phase:	Solvent A = 0.1% formic acid in water<br>B = acetonitrile	
Mobile Phase Program:	Time<br>0.00 min<br>8.00 min<br>9.00 min<br>10.00 min	B%<br>10<br>60<br>100<br>100
Injection Volume:	0.75 µL	
Compound Name:	Retention time:	
4-methoxyphenyl Boronic acid (**6**)	1.57 min	
1,3,5-trimethoxybenzene	4.51 min	
Benzyl bromide (**1**)	5.63 min	
Benzyl chloride (**5**)	5.29 min	
Benzyl iodide (**4**)	6.33 min	
4-methoxyphenyl boronic pinacol ester (**2**)	7.06 min	
Product (**3**)	7.62 min	

Example Chromatogram

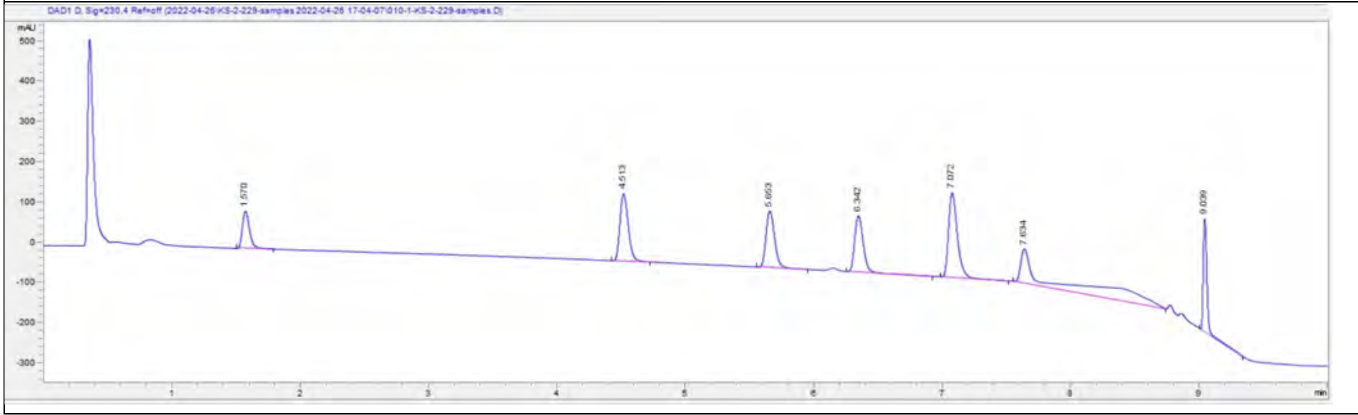

Automated Sampling Platforms and Parameters

Automated sampling technology was used to gather time course data for the reactions in this study. The sampling platform includes a Direct Inject (DI) system with online HPLC as shown in Supplementary Figure 1.

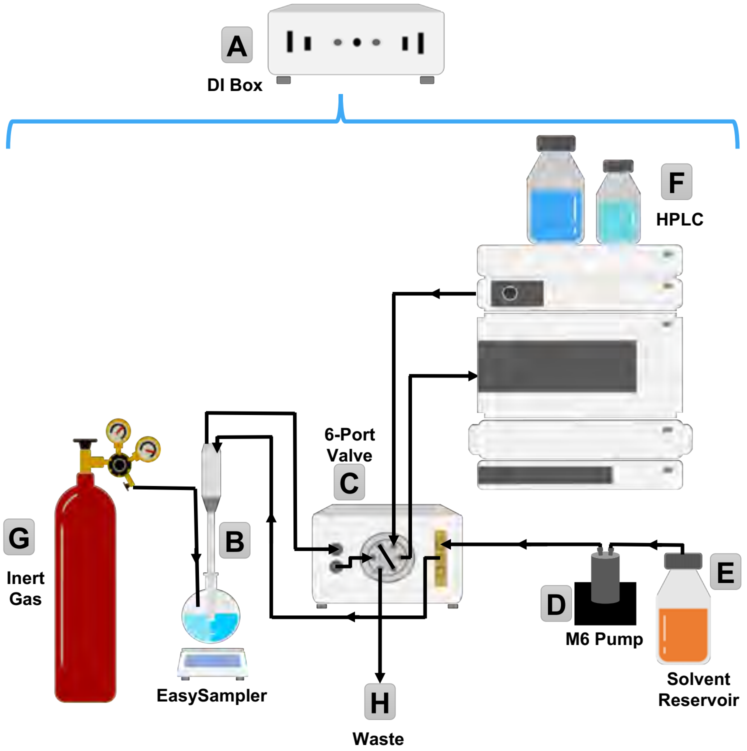

Supplementary Figure 1. Depiction of Direct Inject (DI) automated sampling system coupled with online HPLC. **A**: DI box, which controls and coordinates the entire sampling sequence, run through Python script; **B**: EasySampler probe and reaction flask; **C**: 2-position 6-port injection valve with solvent/gas selector and pressure sensor; **D**: M-6 pump; **E**: push solvent reservoir (THF); **F**: HPLC, see general information for the general information; **G**: argon tank.

Direct Inject system with online HPLC: Temporal HPLC data was obtained using a modified reaction monitoring platform similar to that which has been reported in our group previously.[1-2] All sampling

events were executed by an Arduino microcontroller which was controlled *via* a Python script. The sampling sequence begins by actuating the EasySampler to extend the sampling pocket into the reaction mixture. The system is flushed with THF (5 mL at 3.5 mL/min). The pocket is then retracted, the valve position is switched bringing the sample loop in line with the EasySampler, and THF (standard volume = 0.85 mL) is delivered (default flow rate = 5.0 mL/min) filling the sample loop with the desired reaction aliquot. The injection valve is then switched, aligning the reaction aliquot with the HPLC pump and column to allow for online analysis. The sampling lines are flushed with THF (5.0 mL, 3.5 mL/min) before reinitiating the sampling sequence. The Python sampling script is available upon request.

Conversion of Peak Area to Concentration

HPLC normalized peak area was converted to concentration (M) *via* a calibration curve as shown in Supplementary Figure 2. All calibration curves were done using commercially available reagents except for benzyl iodide (**4**). The calibration curve of **4** was done by running a Finkelstein-reaction. Benzyl chloride was treated with sodium iodide in acetone, and the formation of benzyl iodide was quantitatively formed in situ as shown in Supplementary Figure 3.

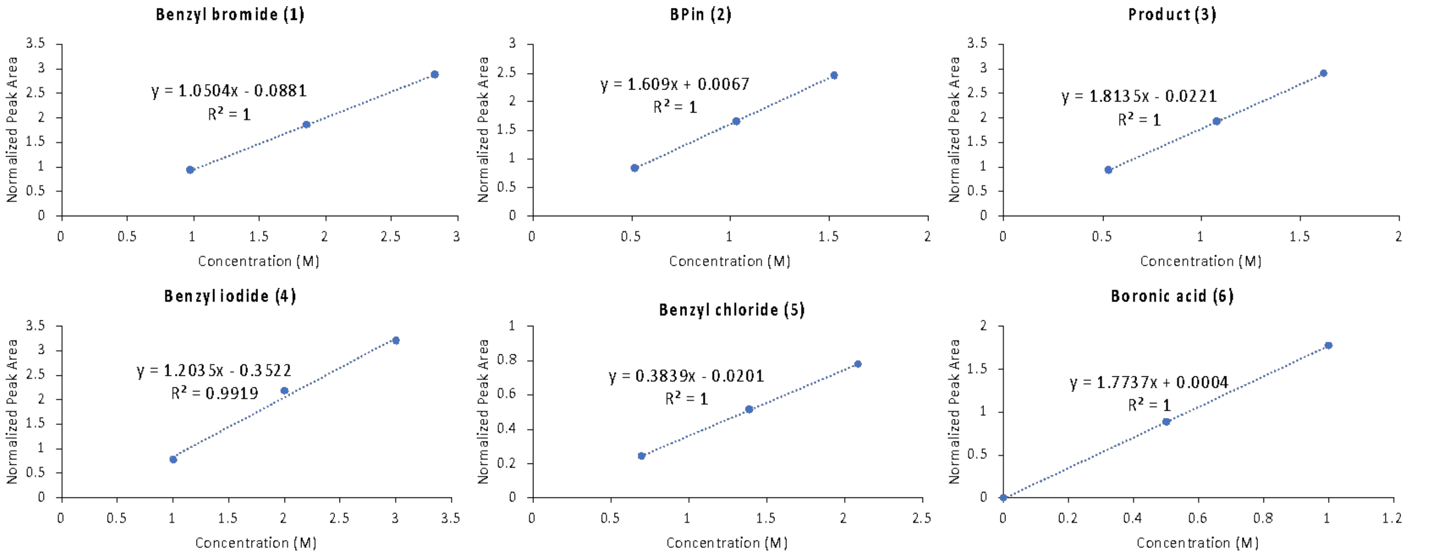

Supplementary Figure 2. HPLC Calibration curves to allow for quantification of **1, 2, 3, 4, 5** and **6** directly from normalized peak area. Integration of all UV peaks completed at a wavelength of 230 nm.

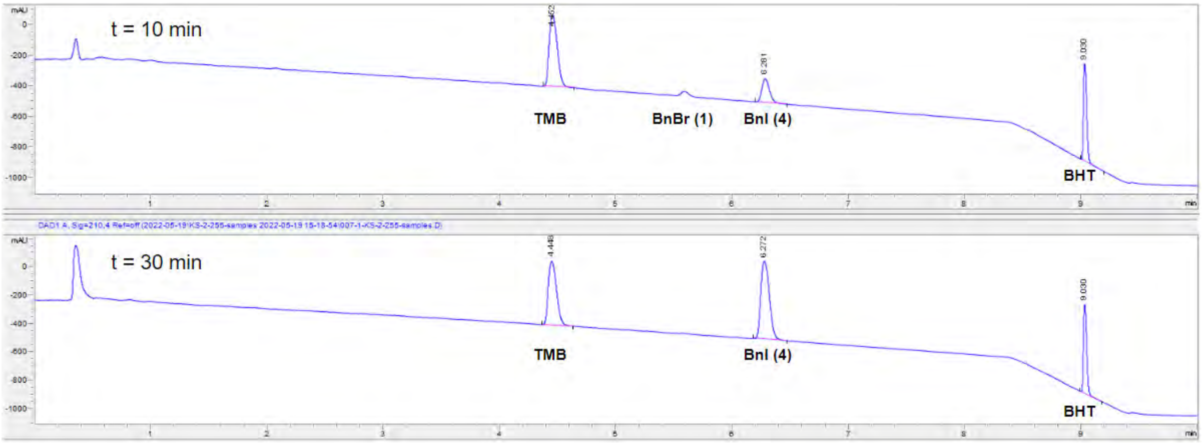

Supplementary Figure 3. The formation of benzyl iodide was confirmed by Finkelstein-reaction.

Experimental Details

General procedure A

To an oven-dried 15 mL two-neck pear shaped flask with a magnetic stir bar was added 1,3,5-trimethoxybenzene (84 mg, 0.50 mmol), 4-methoxyphenylboronic ester (**2**) (175 mg, 0.75 mmol). The flask was sealed with two rubber septa and put on a Schlenk line. The system was evacuated for five minutes and backfilled with argon three times. Meanwhile, a base solution was made by adding K_2CO_3 (1.658 g, 12 mmol) to an oven-dried 9 mL vial. The vial with K_2CO_3 was evacuated for five minutes and backfilled with argon three times before 6.0 mL of H_2O was added to the vial. A precatalyst solution was also made by adding XPhos Pd G2 (59 mg, 0.075 mmol) to an oven-dried 3 mL vial. The vial with catalyst was evacuated for five minutes and backfilled with argon three times before 1.5 mL of 2-MeTHF was added to the vial. Then, under a high flow of argon, one septum of the reaction flask was removed and the EasySampler probe was inserted into one neck of the flask. Next, 6 mL of 2-MeTHF and benzyl bromide (**1**) (171 mg, 1.0 mmol) was added to the reaction flask under argon. The reaction was heated to 80 °C and stirred at 1200 rpm. After 15 minutes at 80 °C, three test samples were taken from the reaction mixture to ensure reproducible sampling. Finally, 1.0 mL of the catalyst solution, containing XPhos Pd G2 (39.34 mg, 0.05 mmol), 3 mL of K_2CO_3 base solution were added to the reaction flask and the sampling sequence was immediately started.

Online monitoring of the hydrolysis of 4-methoxyphenyl boronic acid pinacol ester

The hydrolysis of 4-methoxyphenyl boronic acid pinacol ester was run to explore the possible coupling partner in the transmetalation step. The background rate of hydrolysis of **2** was observed to be much slower than the rate of product formation suggesting transmetalation occurs with **2** directly and not the parent boronic acid **6**.

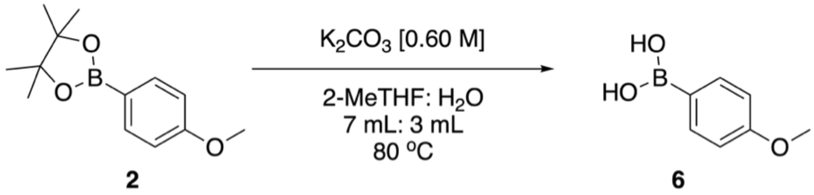

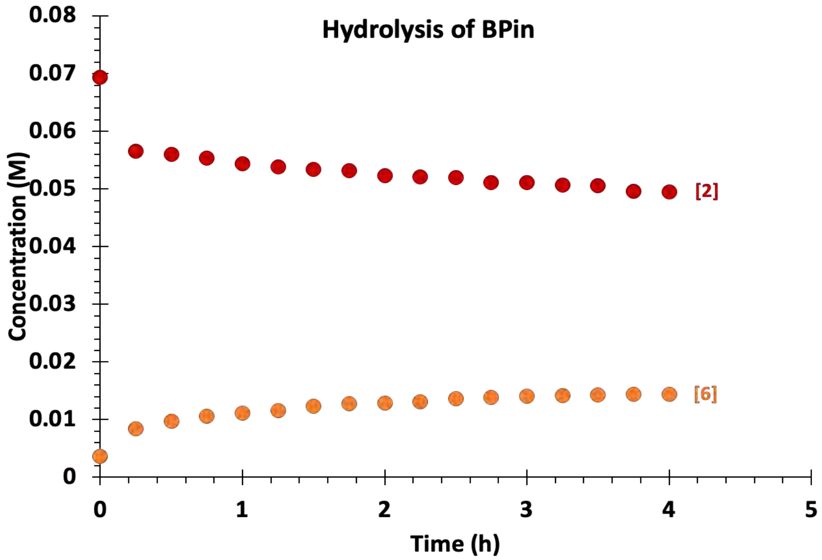

Supplementary Figure 4. The hydrolysis of 4-methoxyphenyl boronic acid pinacol ester: $[\mathbf{2}]_0 = 0.075$ M, $[K_2CO_3]_0 = 0.60$ M.

Different excess experiments and VTNA analysis

A series of different excess reactions were conducted followed by VTNA[3] to determine the orders of **1**, **2**, K_2CO_3 and XPhos Pd G2. These experiments were set up according to general procedure A using the loadings listed in Supplementary Table 1. For sake of clarity, Supplementary Figure 3 from the text shows VTNA analysis of one standard condition time course overlaid with a single different excess experiment. To further corroborate the conclusion drawn from these experiments, Supplementary Figures 5, 7, 9 and 11 provide an overlay of two different excess reactions conducted at different concentrations with the two standard conditions time course experiments. This highlights that the changes observed within the different excess experiments are well outside the bounds of reproducibly observed in the system. Supplementary Figure 6, 8, 10, 12 provide the VTNA analysis which provided the best overlay. Note that orders were determined for each set of same excess experiments separately (ie: comparing trial 1 to trial 2, separately from trial 1 and trial 3) instead of comparing all three concentrations (ie: comparing trial 1, trial 2, and trial 3). This was due to the fact that significantly poorer overlay was observed when using the latter method suggesting the order is likely changing as a result of concentration changes. The full data can be found in an attached excel spreadsheet.

Supplementary Table 1: Initial reagent conditions for determination of reaction rate dependence upon substrate concentration

Trial	$[\mathbf{1}]_0$ (M)	$[\mathbf{2}]_0$ (M)	$[K_2CO_3]$ (M)	[XPhos Pd G2] (mM)
1	0.10	0.075	0.60	5
1’	0.10	0.075	0.60	5
2	0.15	0.075	0.60	5
3	0.05	0.075	0.60	5
4	0.10	0.113	0.60	5
5	0.10	0.038	0.60	5

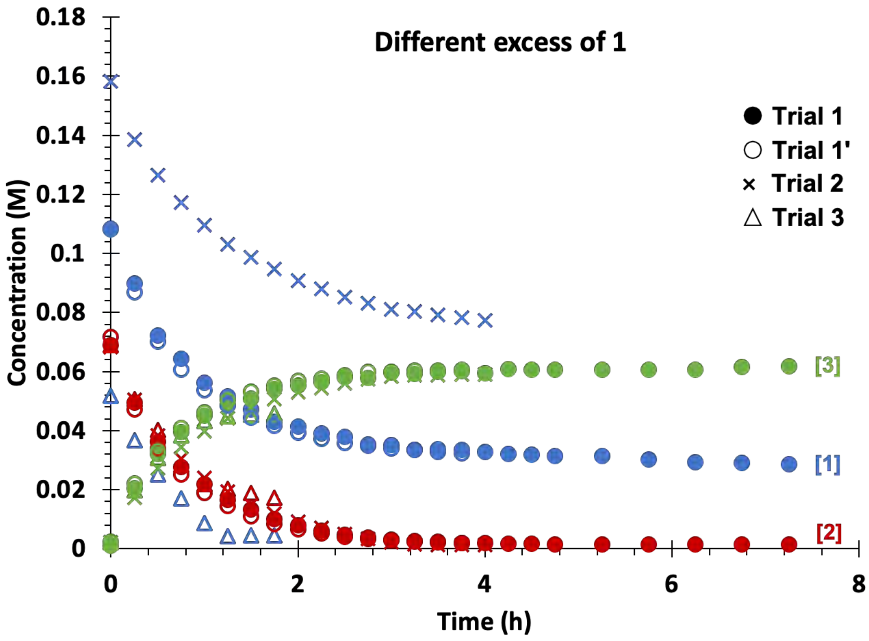

Supplementary Figure 5. Reaction time-course data for the Suzuki-Miyaura reaction with varying initial concentrations of **1**.

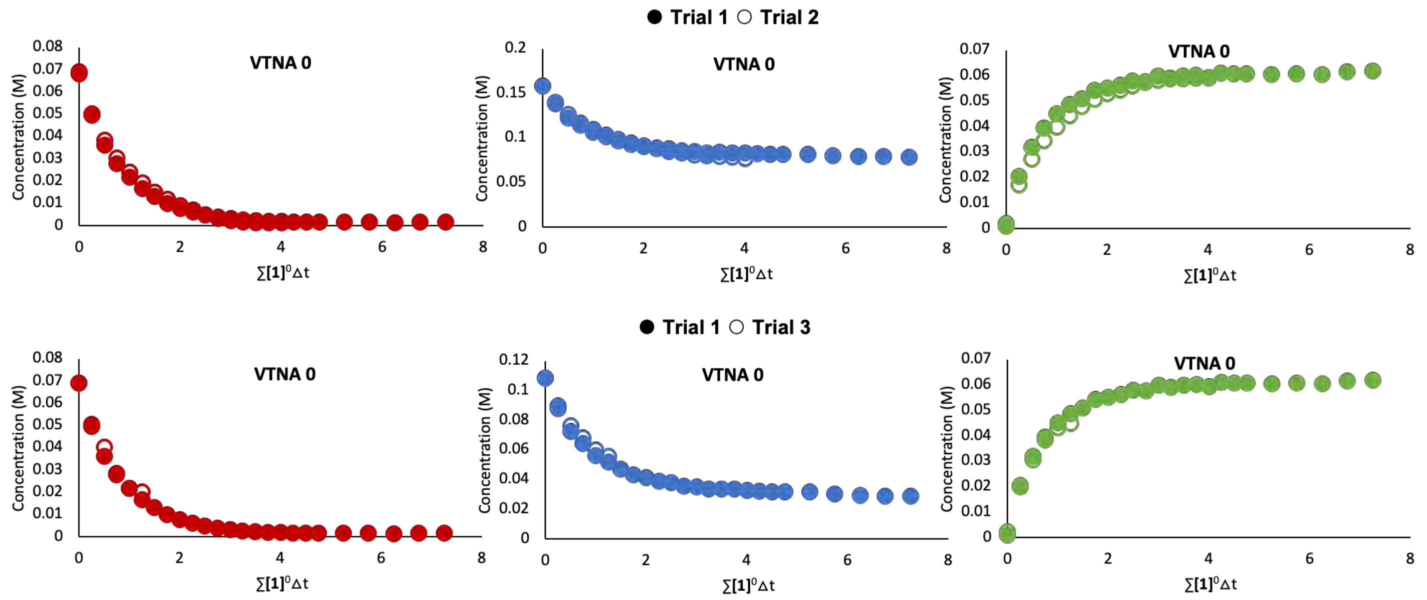

Supplementary Figure 6. Variable time normalization analysis plot to solve for order of benzyl bromide (**1**) using data from Supplementary Table 1

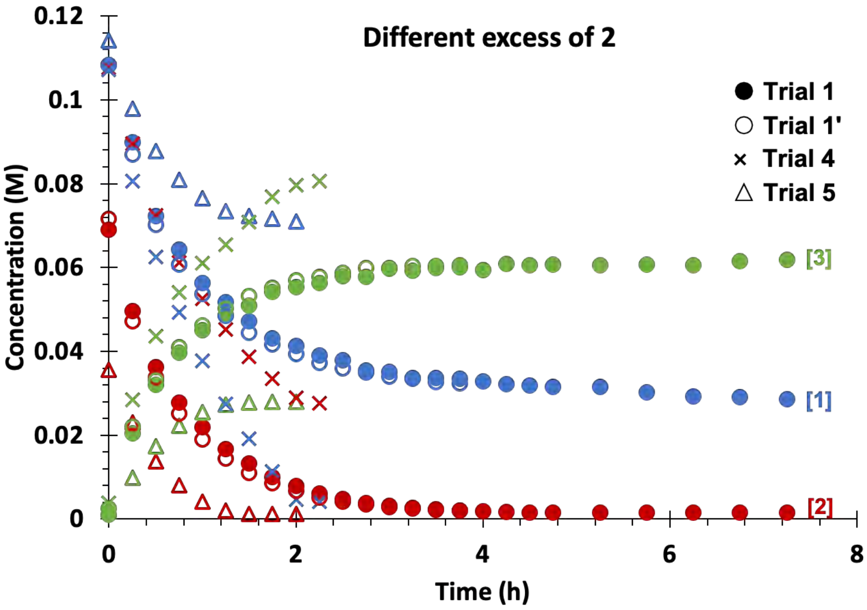

Supplementary Figure 7. Reaction time-course data for the Suzuki-Miyaura reaction with varying initial concentrations of **2**.

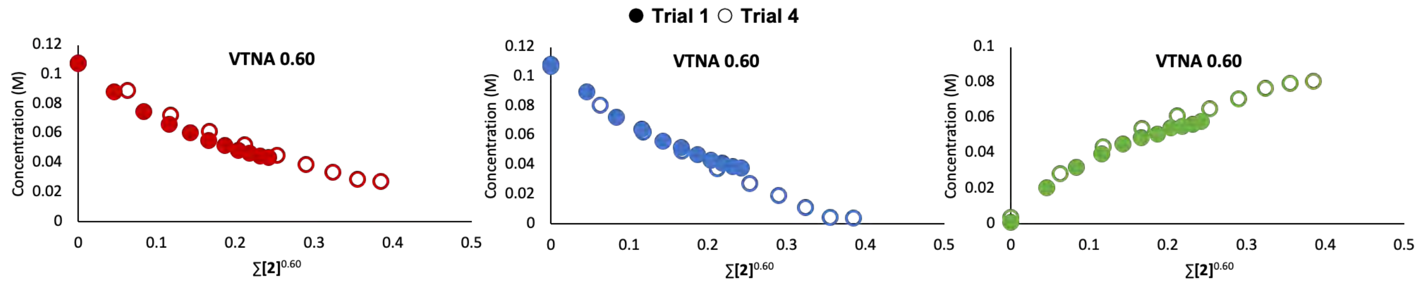

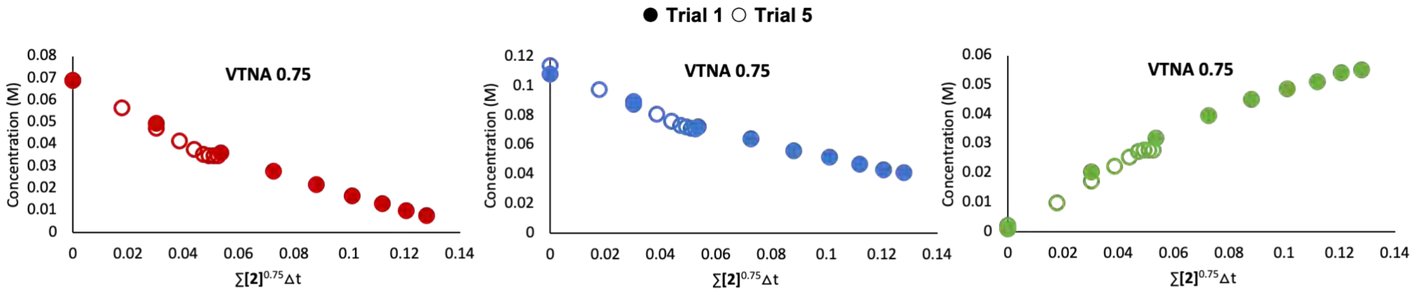

Supplementary Figure 8. Variable time normalization analysis plot to solve for order of 4-methoxyphenyboronic acid pinacol ester (**2**) using data from Supplementary Table 1.

Supplementary Table 2: Initial reagent conditions for determination of reaction rate dependence upon XPhos Pd G2 concentration

Trial	[**1**]$_0$ (M)	[**2**]$_0$ (M)	[K_2CO_3] (M)	[XPhos Pd G2] (mM)
1	0.10	0.075	0.60	5
1'	0.10	0.075	0.60	5
6	0.10	0.075	0.60	2.5
7	0.10	0.075	0.60	1.25

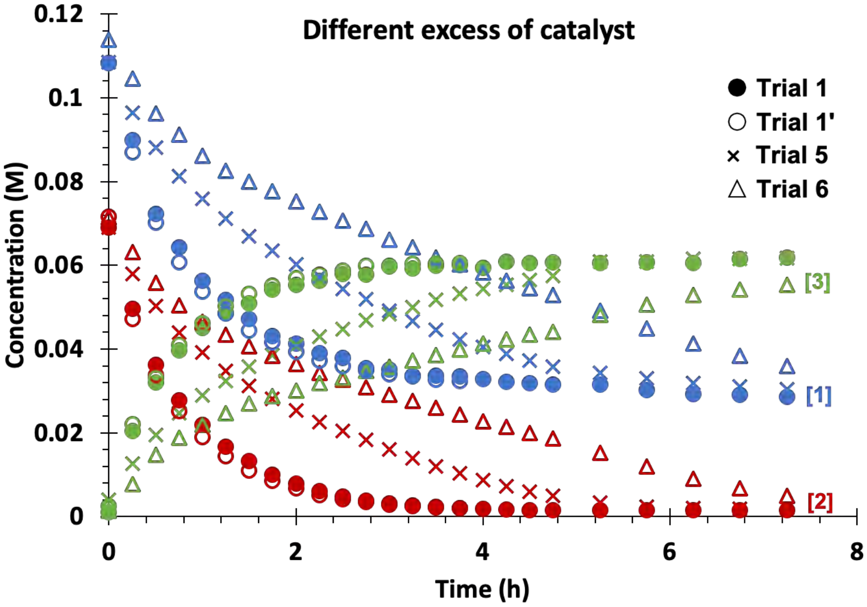

Supplementary Figure 9. Reaction time-course data for the Suzuki-Miyaura reaction with varying concentrations of XPhos Pd G2.

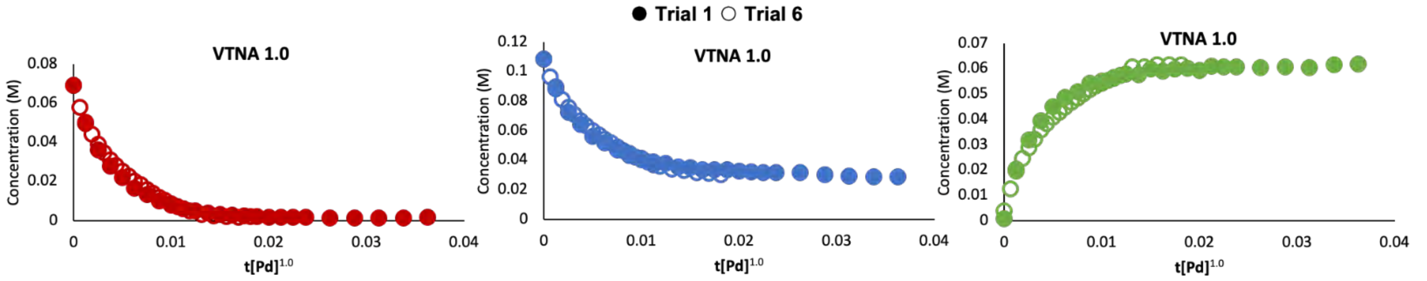

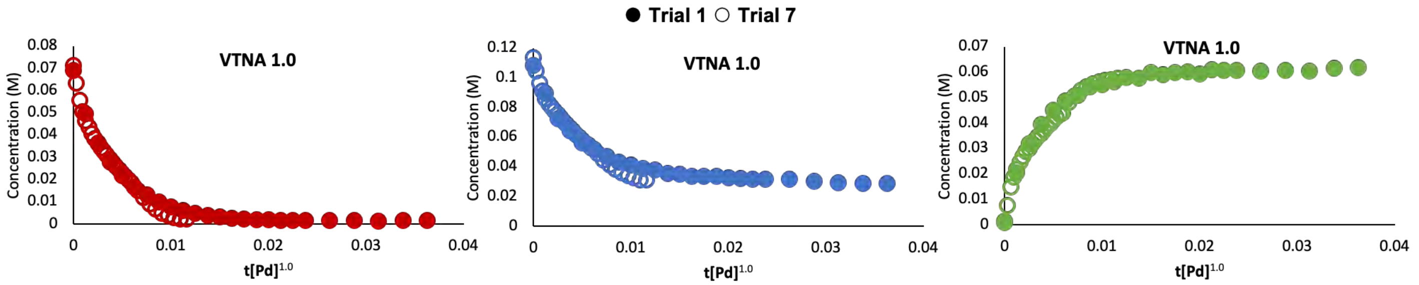

Supplementary Figure 10. Variable time normalization analysis plot to solve for order of XPhos Pd G2 using data from Supplementary Table 2.

Supplementary Table 3: Initial reagent conditions for determination of reaction rate dependence upon K_2CO_3 concentration

Trial	[**1**]$_0$ (M)	[**2**]$_0$ (M)	[K_2CO_3] (M)	[XPhos Pd G2] (mM)
1	0.10	0.075	0.60	5
1’	0.10	0.075	0.60	5
8	0.10	0.075	0.48	5
9	0.10	0.075	0.30	5

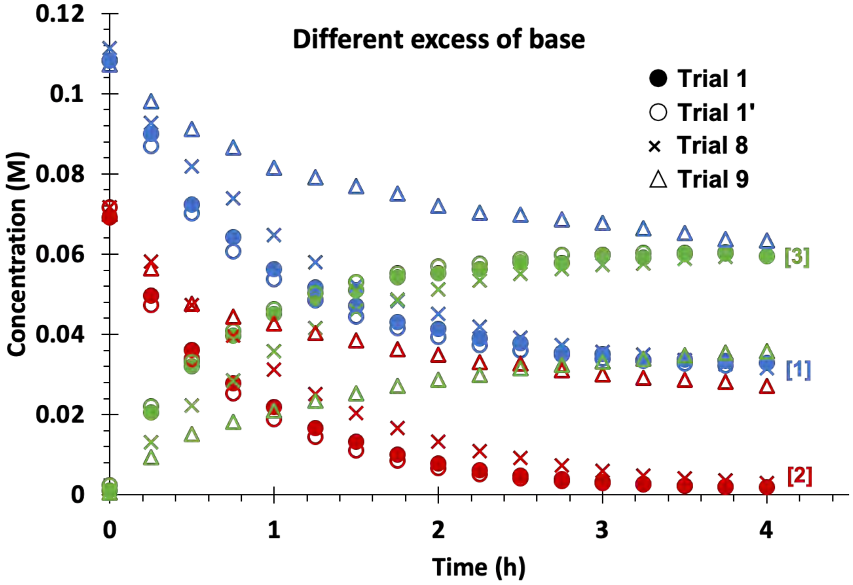

Supplementary Figure 11. Reaction time-course data for the Suzuki-Miyaura reaction with varying concentrations of K_2CO_3.

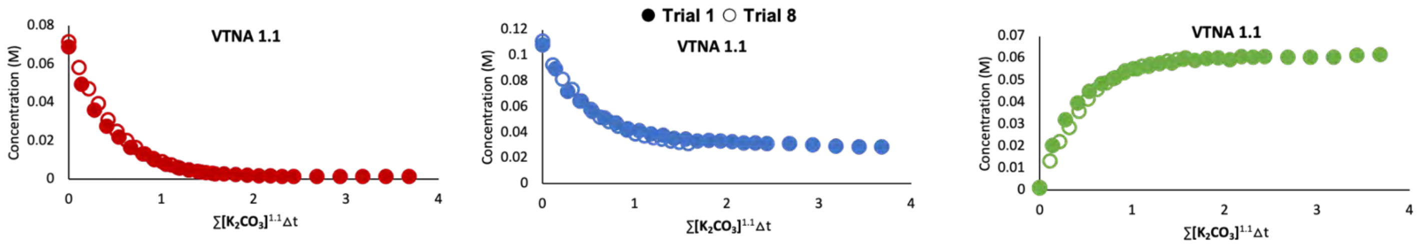

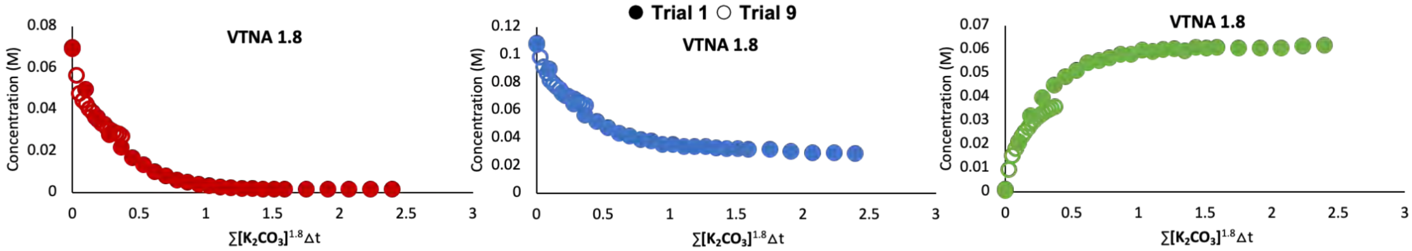

Supplementary Figure 12. Variable time normalization analysis plot to solve for order of K_2CO_3 using data from Supplementary Table 3. Note: For the VTNA analysis of the base, the concentration of K_2CO_3 was estimated as it can not be monitored directly. A 1:1 relationship between the consumption of BPin and that of K_2CO_3 was used to calculate its concentration at each time point. The assumption of a 1:1 relationship is supported by the excellent overlay achieved in the same excess experiment.

Same excess experiments

A series of same excess experiments were conducted to probe the stability of the catalyst under the reaction conditions. These experiments were set up according to general procedure A using the loadings listed in Supplementary Table 4 This was done by conducting the reaction (trial 10) with the removal of 0.050 M **1**, **2**, and K_2CO_3 while holding the catalyst concentration constant. By applying a time shift along the x-axis, we can evaluate the catalyst activity after several turnovers compared to the use of fresh catalyst. As can be gleaned from Supplementary Figure 13A, the catalyst suffers significant degradation or inhibition under the reaction conditions. Repeating the same excess experiment while in the presence of 0.050 M of KBr (trial 11) revealed near perfect overlay of both **1** and **2**. Finally, conducting the trial 12 in the presence of both 0.050 M of KBr and the product (**3**) produced the same result. Taken together, these results suggest that degradation of the active catalyst is negligible under the reaction conditions and the product does not display catalyst inhibition. However, KBr was observed to significantly inhibit the catalyst from same excess reactions. Notably, the lack of measurable catalyst degradation supports our conclusion that Pd nanoparticles are unlikely to be kinetically relevant under the targeted conditions. This is further supported by the absence of observable Pd black formation during the reaction (Supplementary Figure 13B).

Supplementary Table 4: Initial reagent conditions to probe the stability of the catalyst

Trial	[**1**]$_0$ (M)	[**2**]$_0$ (M)	[K_2CO_3] (M)	[KBr] (M)	[**3**]$_0$ (M)	[XPhos Pd G2] (mM)
1	0.10	0.075	0.60	0	0	5
10	0.05	0.025	0.55	0	0	5
11	0.05	0.025	0.55	0.05	0	5
12	0.05	0.025	0.55	0.05	0.05	5

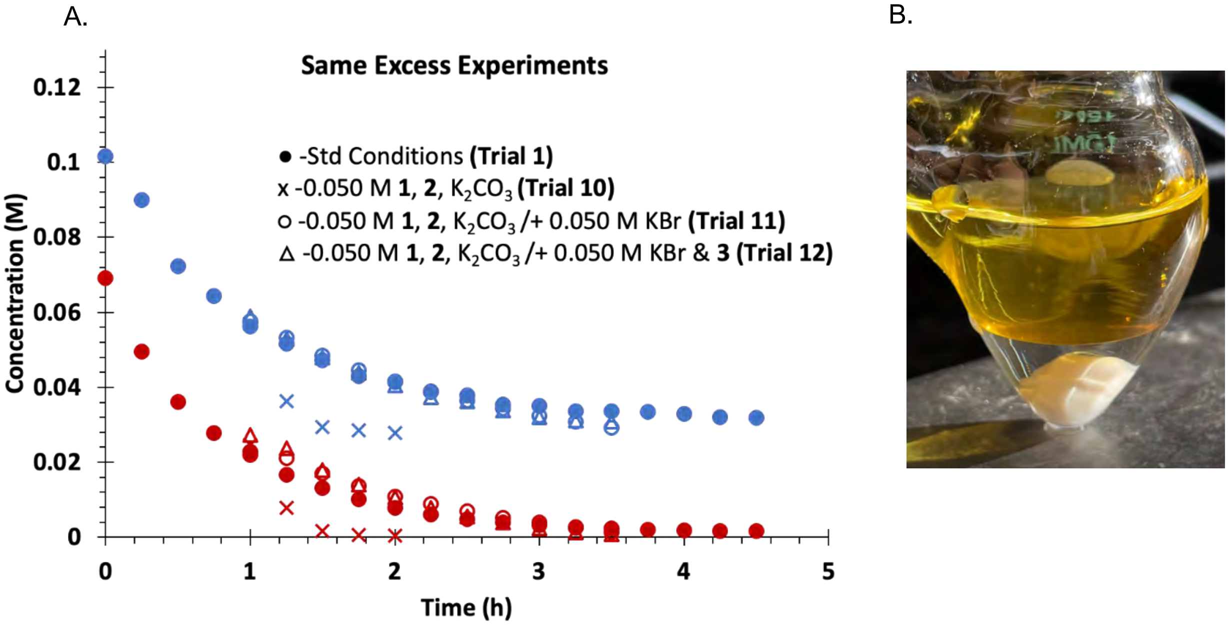

Supplementary Figure 13. A. Time course plots of same excess experiments probing catalyst degradation/inhibition. B. Picture of the standard reaction solution (**Trial 1**)

Probing the Inhibitory effect of potassium halides

To an oven-dried 15 mL two-neck pear shaped flask with a magnetic stir bar was added 1,3,5-trimethoxybenzene (84 mg, 0.50 mmol), 4-methoxyphenylboronic ester (**2**) (175 mg, 0.75 mmol). The flask was sealed with two rubber septa and put on a Schlenk line. The system was evacuated for five minutes and backfilled with argon three times. Meanwhile, a base solution was made by adding K_2CO_3 (1.658 g, 12 mmol) to an oven-dried 9 mL vial. The vial with K_2CO_3 was evacuated for five minutes and backfilled with argon three times before 6.0 mL of water was added to the vial. A precatalyst solution was also made by adding XPhos Pd G2 (59 mg, 0.075 mmol) to an oven-dried 3 mL vial. The vial with catalyst was evacuated for five minutes and backfilled with argon three times before 1.5 mL of 2-MeTHF was added to the vial. Then, under a high flow of argon, one septum of the reaction flask was removed and the EasySampler probe was inserted into one neck of the flask. Next, 6 mL of 2-MeTHF and benzyl bromide (**1**) (171 mg, 1.0 mmol) were added to the reaction flask under argon. The reaction was heated to 80 °C and stirred at 1200 rpm. After 15 minutes at 80 °C, three test samples were taken from the reaction mixture to ensure reproducible sampling. Then, under positive pressure, one septum was removed and solid KBr (119 mg, 1.0 mmol) or KCl (74.5 mg, 1.0 mmol) or KI (166 mg, 1.0 mmol) was added to the reaction flask. Finally, 1.0 mL of the catalyst solution, containing XPhos Pd G2 (39.34 mg, 0.05 mmol), 3 mL of K_2CO_3 base solution was added to the reaction flask and the sampling sequence was immediately started.

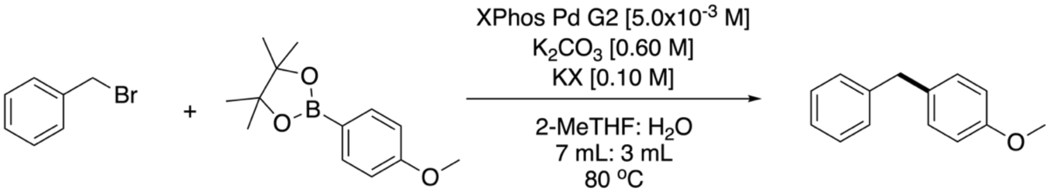

1 **2** **3**

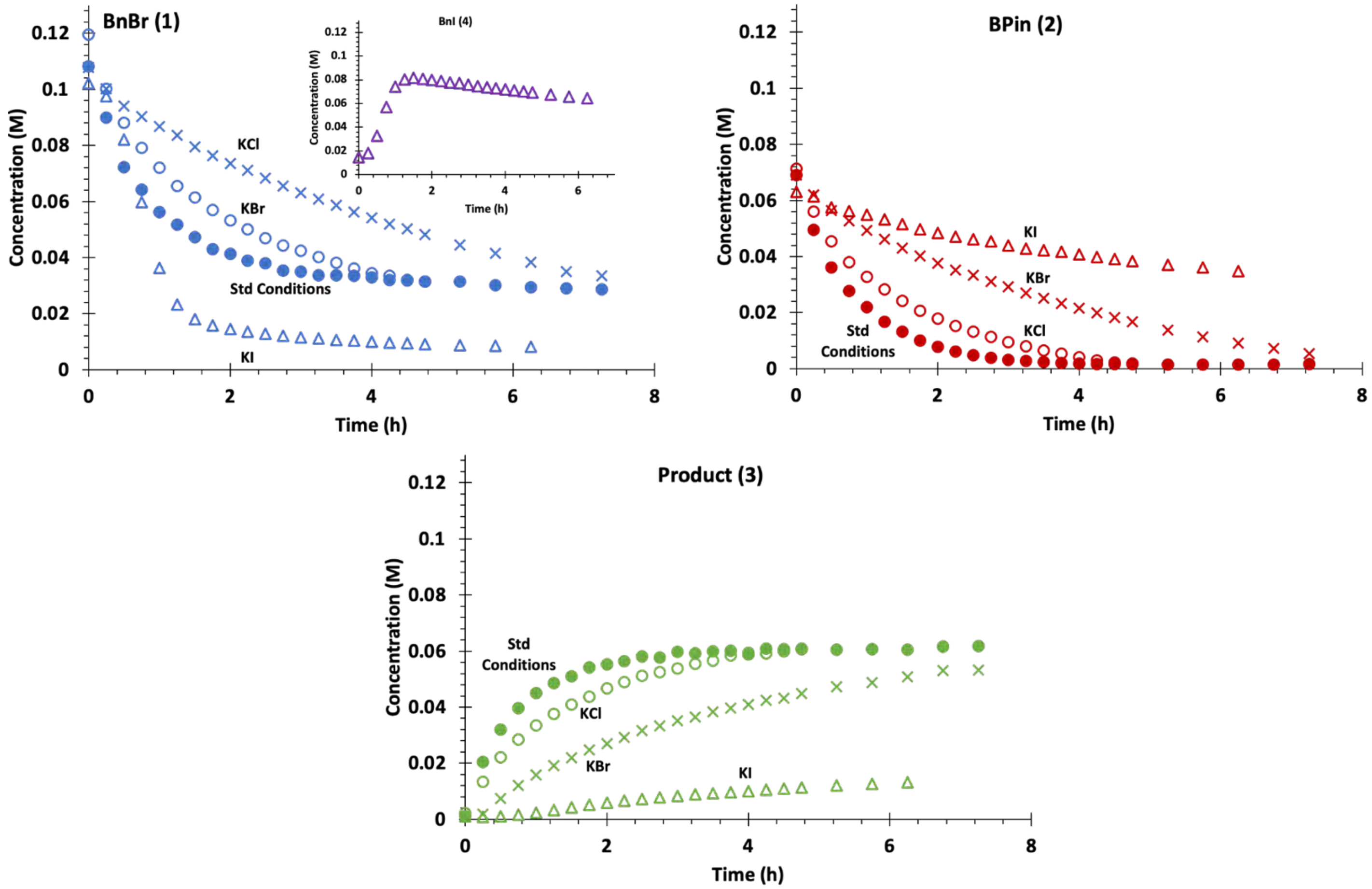

Supplementary Figure 14. Time course data probing inhibitory effect of KCl, KBr, and KI compared to standard conditions.

Kinetic study the competition reaction containing benzyl bromide and benzyl chloride

To an oven-dried 15 mL two-neck pear shaped flask with a magnetic stir bar was added 1,3,5-trimethoxybenzene (84 mg, 0.50 mmol), 4-methoxyphenylboronic ester (**2**) (175 mg, 0.75 mmol). The flask was sealed with two rubber septa and put on a Schlenk line. The system was evacuated for five minutes and backfilled with argon three times. Meanwhile, a base solution was made by adding K_2CO_3 (1.658 g, 12 mmol) to an oven-dried 9 mL vial. The vial with K_2CO_3 was evacuated for five minutes and backfilled with argon three times before 6.0 mL of water was added to the vial. A precatalyst solution was also made by adding XPhos Pd G2 (59 mg, 0.075 mmol) to an oven-dried 3 mL vial. The vial with catalyst was evacuated for five minutes and backfilled with argon three times before 1.5 mL of 2-MeTHF was added to the vial. Then, under a high flow of argon, one septum of the reaction flask was removed and the EasySampler probe was inserted into one neck of the flask. Next, 6 mL of 2-MeTHF, benzyl bromide (**1**) (171 mg, 1.0 mmol) and benzyl chloride (**5**) (126.6 mg, 1.0 mmol) were added to the reaction flask under argon. The reaction was heated to 80 °C and stirred at 1200 rpm. After 15 minutes at 80 °C, three test samples were taken from the reaction mixture to ensure reproducible sampling. Finally, 1.0 mL of the catalyst solution, containing XPhos Pd G2 (39.34 mg, 0.05 mmol), 3 mL of K_2CO_3 base solution were added to the reaction flask and the sampling sequence was immediately started.

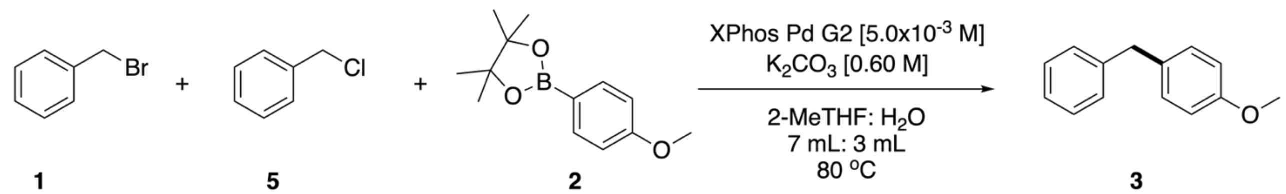

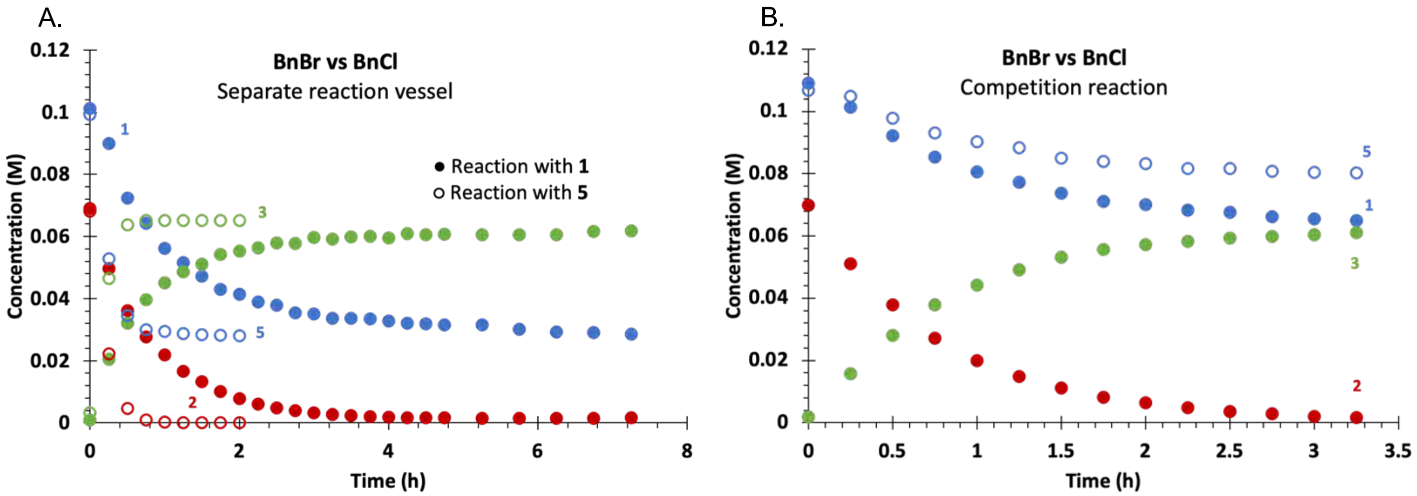

Supplementary Figure 15. Time course overlay of SMC using electrophiles **1** and **5** in separate reaction systems (A), and as a competition reaction in the same flask (B).

Control reaction using benzyl chloride with KBr additive to check the benzyl bromide formation

The control reaction using benzyl chloride **5** with the KBr as additive was run and observed no formation of benzyl bromide **3**, suggesting that benzyl chloride **5** is stable in the presence of KBr and the palladium catalyst.

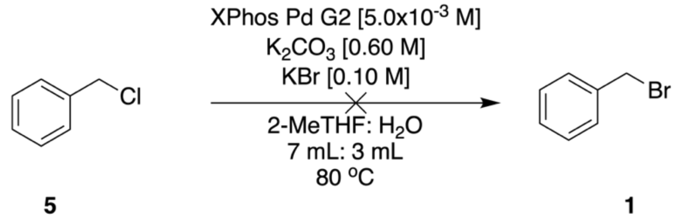

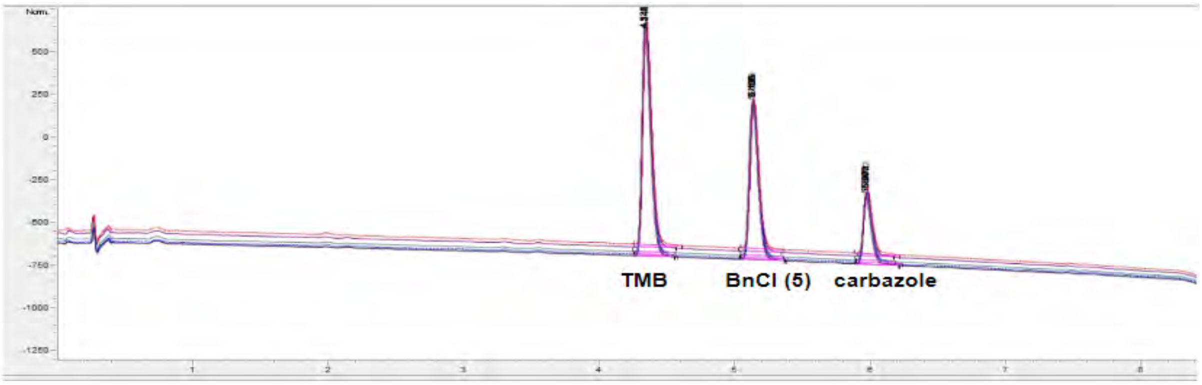

Supplementary Figure 16. Stacked view of HPLC profiles of the control reaction using benzyl chloride in the presence of KBr and XPhos Pd G2 catalyst showing no reactivity across 5 samples over the course of 1 hour.

Conclusions from time course reactions of 1 and 5

The reactivity of **5** is clearly superior to that of **1** in separate reaction vessels (Supplementary Figure 15, top). This provides further evidence of the lack of kinetic relevance the oxidative addition step has on the observed kinetics as bond strengths would suggest the opposite trend. Instead, this highlights the important role the halide identity plays on transmetalation. Running a competition reaction reveals a slight preference for the consumption of **1** over **5** despite the higher reactivity **5** in separate vessels (Supplementary Figure 15, top). Moreover, the total reaction time of the competition reaction is quite similar to that of **1** on its own. We believe this suggests the oxidative addition is non-reversible and **1** has a lower barrier to oxidative addition resulting in its preferential consumption under the reaction conditions. In contrast, if oxidative addition was reversible, one would expect a preference for the consumption of **5** as it provides the lowest barrier for the turnover limiting step. To ensure the observed results are not confounded by *in situ* speciation of the electrophile a control reaction was run (Supplementary Figure 16) showing that **5** does not convert to **1** under the reaction conditions in the presence of added KBr.

The 12-fold rate acceleration by the addition of tetrabutylammonium bromide

To an oven-dried 15 mL two-neck pear shaped flask with a magnetic stir bar was added 1,3,5-trimethoxybenzene (84 mg, 0.50 mmol), 4-methoxyphenylboronic ester (**2**) (175 mg, 0.75 mmol) and solid TBAB (322.3 mg, 1.0 mmol). The flask was sealed with two rubber septa and put on a Schlenk line. The system was evacuated for five minutes and backfilled with argon three times. Meanwhile, a base solution was made by adding K_2CO_3 (1.658 g, 12 mmol) to an oven-dried 9 mL vial. The vial with K_2CO_3 was evacuated for five minutes and backfilled with argon three times before 6.0 mL of water was added to the vial. A precatalyst solution was also made by adding XPhos Pd G2 (59 mg, 0.075 mmol) to an oven-dried 3 mL vial. The vial with catalyst was evacuated for five minutes and backfilled with argon three times before 1.5 mL of 2-MeTHF was added to the vial. Then, under a high flow of argon, one septum of the reaction flask was removed and the EasySampler probe was inserted into one neck of the flask. Next, 6 mL of 2-MeTHF, benzyl bromide (**1**) (171 mg, 1.0 mmol) were added to the reaction flask under argon. The reaction was heated to 80 °C and stirred at 1200 rpm. After 15 minutes at 80 °C, three test samples were taken from the reaction mixture to ensure reproducible sampling. Finally, 1.0 mL of the catalyst solution, containing Xphos Pd G2 (39.34 mg, 0.05 mmol), 3 mL of K_2CO_3 base solution were added to the reaction flask and the sampling sequence was immediately started.

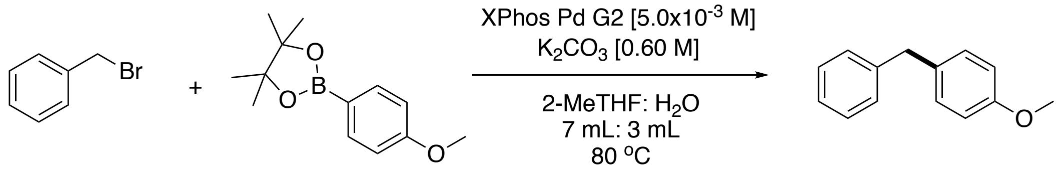

1 **2** **3**

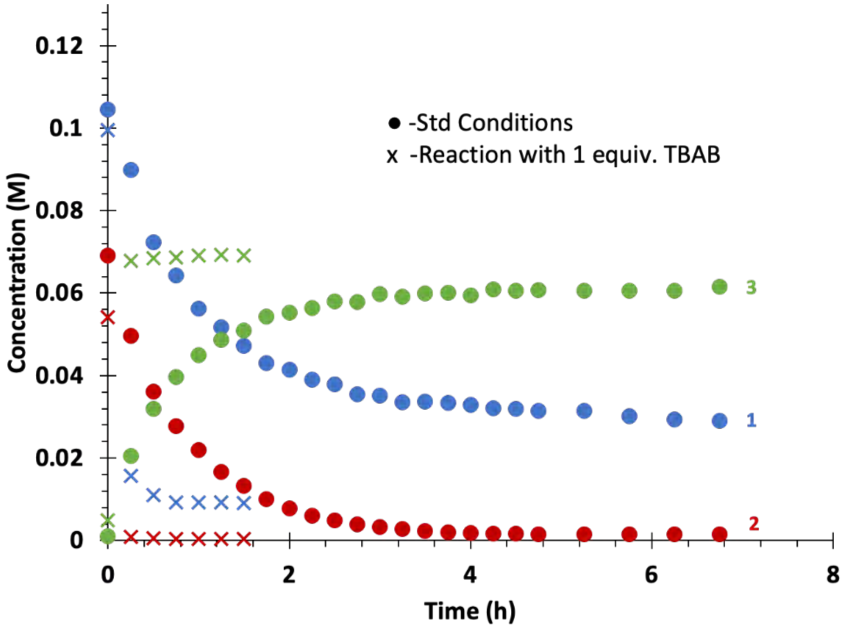

Supplementary Figure 17. The substantial rate acceleration by the addition of TBAB

A Comment on the use of k_{rel}

To better contextualize the results provided in this work we targeted the use of relative rates however, there are significant limitations to this approach as will be delineated here. Typically, relative rates (k_{rel}) are achieved through a comparison of initial rates where one set of conditions serves as the standard by which all other reactions are compared to. Best practice when conducting initial rates would be to target a line of best fit over four data points at approximately 10 to 20% consumption of starting material to ensure steady state kinetics have been reached. This was not amenable to the study at hand due to the relatively low data density coupled with the lack of linearity of the time course in these regions. In this work, we calculated k_{rel} in almost all cases by obtaining initial rates by analysing t=0 and t=15 min. We believe the context provided through this is useful however, obvious examples of its limitations when used as single descriptor of a reaction are present. For example, when applied to the use of different organoboron nucleophiles (Figure 6 of the manuscript) the boronic acid species **6** provides the greatest relative rate despite the neopentyl glycol derivative **7** clearly reaching completion in almost half the reaction time. A further example would be its use when comparing the inhibitory impact of potassium salts (Supplementary Figure 14). In this case KBr clearly outperforms KI however, when comparing conversion up until 15 minutes, they perform similarly.

Finally, one may instead favor using time-to-completion as a manner to calculate k_{rel} while limiting the impact of the outliers when analyzing only the start of the reaction. This is in fact what we leveraged when analysing Supplementary Figure 17 as the reaction rate in the presence of TBAB resulted in the complete conversion prior to the second sample being acquired (t=15 min). As such, measuring initial rates in this case would be misleading. Instead, we compare the reduction in the time it took to achieve complete consumption of the limiting reagent which changes from 300 minutes to a maximum of 15 minutes with the TBAB additives. Again, such a method is not universal and also suffers limitation as, not all reactions reach complete conversion.

Overall, we believe there is value in including these analyses to enable easy comparisons for readers to contextualize the findings reported herein, however one must always analyze such data with caution and always favor an analysis of the complete time course profile whenever possible.

The reaction rates comparison of different tetrabutylammonium salts or potassium salts under milder conditions

To an oven-dried 15 mL two-neck pear shaped flask with a magnetic stir bar was added 1,3,5-trimethoxybenzene (84 mg, 0.50 mmol), 4-methoxyphenylboronic ester (**2**) (175 mg, 0.75 mmol) and solid 1.0 mmol TBAX salts or KX salts. The flask was sealed with two rubber septa and put on a Schlenk line. The system was evacuated for five minutes and backfilled with argon three times. Meanwhile, a base solution was made by adding K_2CO_3 (830 mg, 6.0 mmol) to an oven-dried 6 mL vial. The vial with K_2CO_3 was evacuated for five minutes and backfilled with argon three times before 3.0 mL of water was added to the vial. A precatalyst solution was also made by adding Xphos Pd G2 (14.75 mg, 0.01875 mmol) to an oven-dried 3 mL vial. The vial with catalyst was evacuated for five minutes and backfilled with argon three times before 1.5 mL of 2-MeTHF was added to the vial. Then, under a high flow of argon, one septum of the reaction flask was removed and the EasySampler probe was inserted into one neck of the flask. Next, 7.5 mL of 2-MeTHF and benzyl bromide (**1**) (171 mg, 1.0 mmol) were added to the reaction flask under argon. The reaction was heated to 50 °C and stirred at 1200 rpm. After 15 minutes at 50 °C, three test samples were taken from the reaction mixture to ensure reproducible sampling. Finally, 1.0 mL of the catalyst solution, containing Xphos Pd G2 (9.84 mg, 0.0125 mmol), 1.5 mL of K_2CO_3 base solution were added to the reaction flask and the sampling sequence was immediately started.

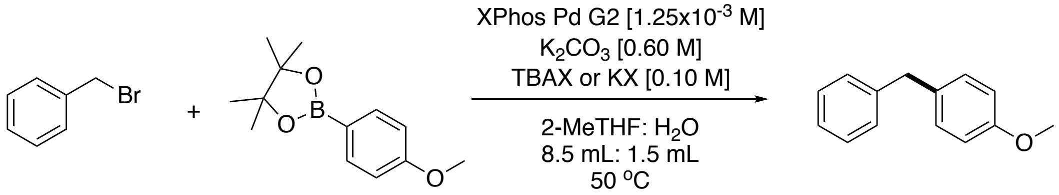

1 **2** **3**

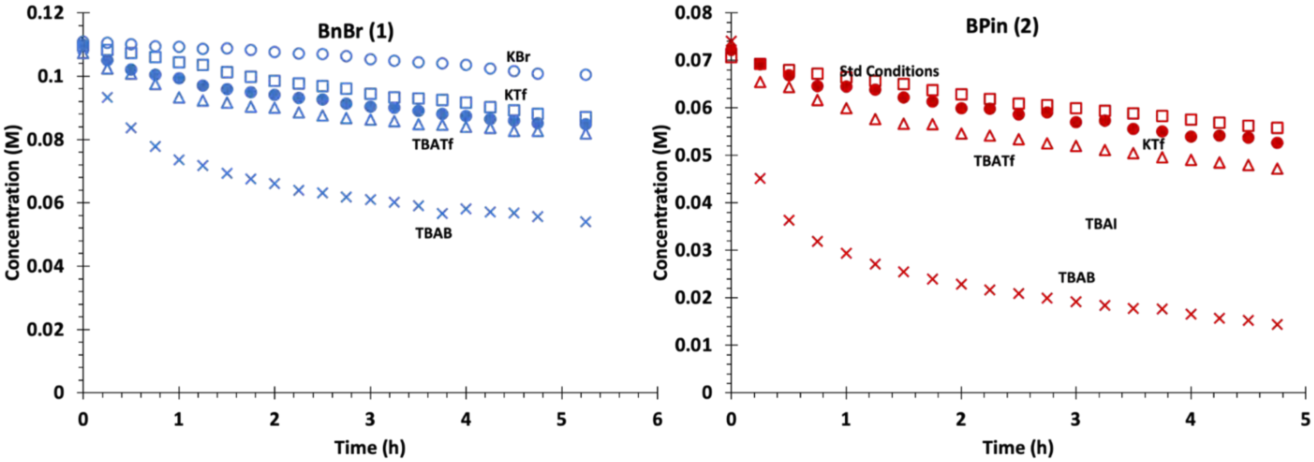

Supplementary Figure 18. Time course data of **1, 2** probing the effect of TBAB, TBATf, KTf, and KBr compared to standard conditions without these additives.

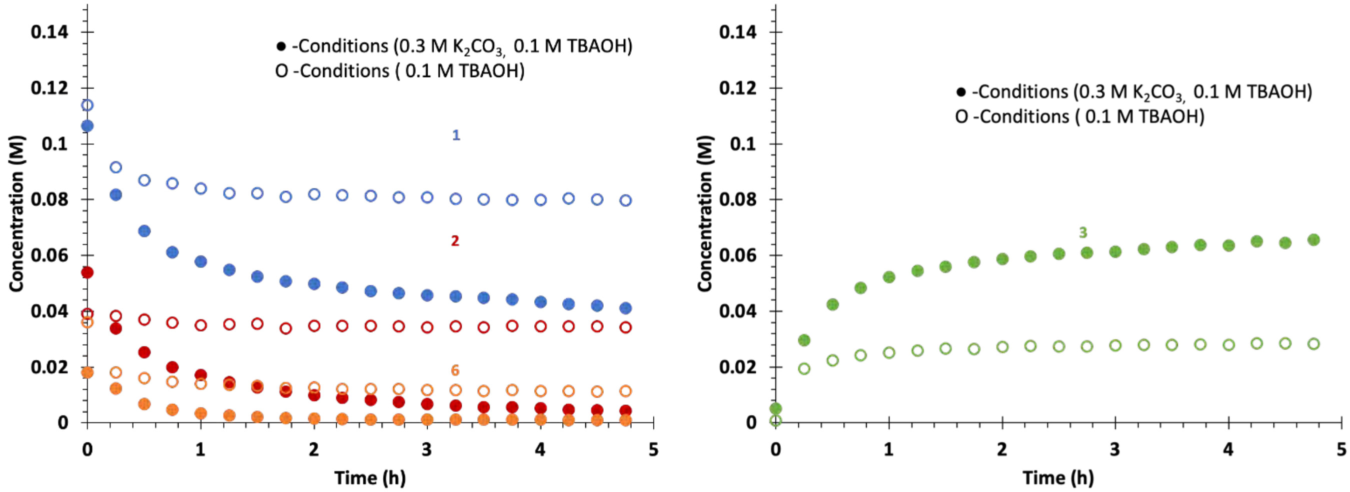

Supplementary Figure 19. Time course data of reactions probing the necessity of K_2CO_3 while using TBAOH.

Different excess experiments and VTNA analysis in the presence of TBAB

In order to confirm the catalyst resting state does not change under the reaction conditions with tetrabutylammonium halides, a series of different excess reactions were conducted followed by VTNA[2] to determine the orders of **1**, **2**, K_2CO_3 and XPhos Pd G2 in the presence of TBAB. These experiments were set up according to general procedure A in the presence of TBAB using the loadings listed in Supplementary Table 5. The full data can be found in an attached excel spreadsheet.

Supplementary Table 5: Initial reagent conditions for determination of reaction rate dependence upon each reagent concentration

Trial	$[\mathbf{1}]_0$ (M)	$[\mathbf{2}]_0$ (M)	$[K_2CO_3]$ (M)	[XPhos Pd G2] (mM)
10	0.10	0.075	0.30	1.25
11	0.85	0.075	0.30	1.25
12	0.10	0.0375	0.30	1.25
13	0.10	0.075	0.30	0.625
14	0.10	0.075	0.15	1.25

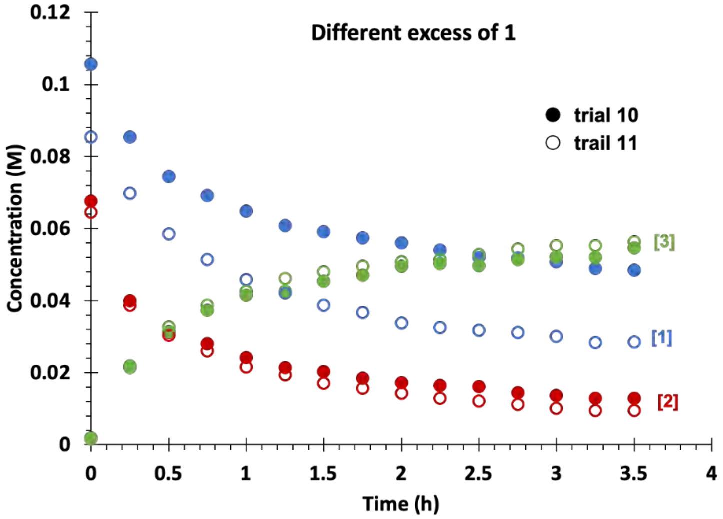

Supplementary Figure 20. Reaction time-course data for the Suzuki-Miyaura reaction with varying initial concentrations of **1** in the presence of TBAB.

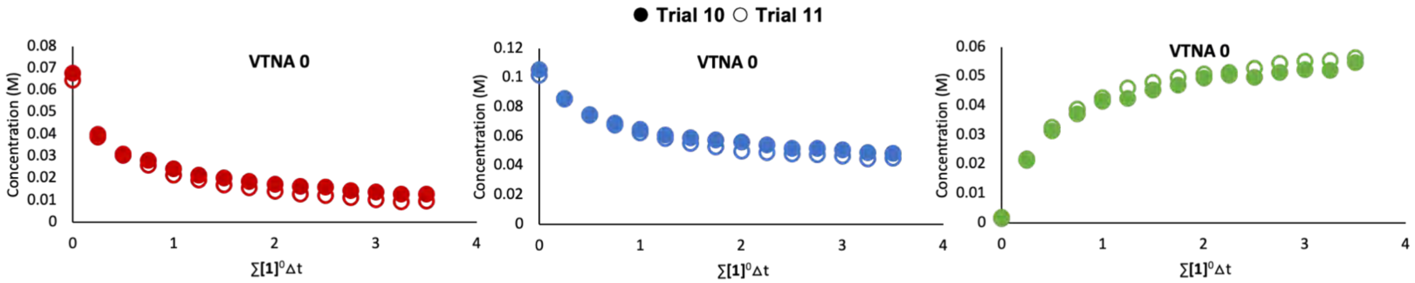

Supplementary Figure 21. Variable time normalization analysis plot to solve for order of benzyl bromide (**1**) using data from Supplementary Table 5

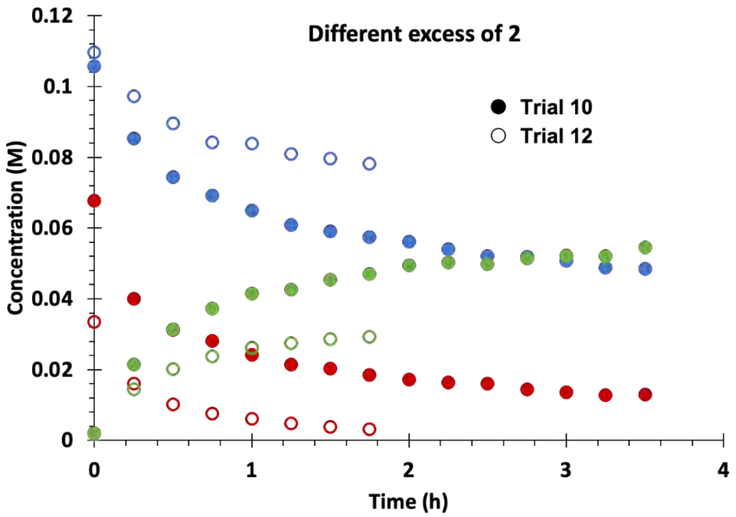

Supplementary Figure 22. Reaction time-course data for the Suzuki-Miyaura reaction with varying initial concentrations of **2** in the presence of TBAB.

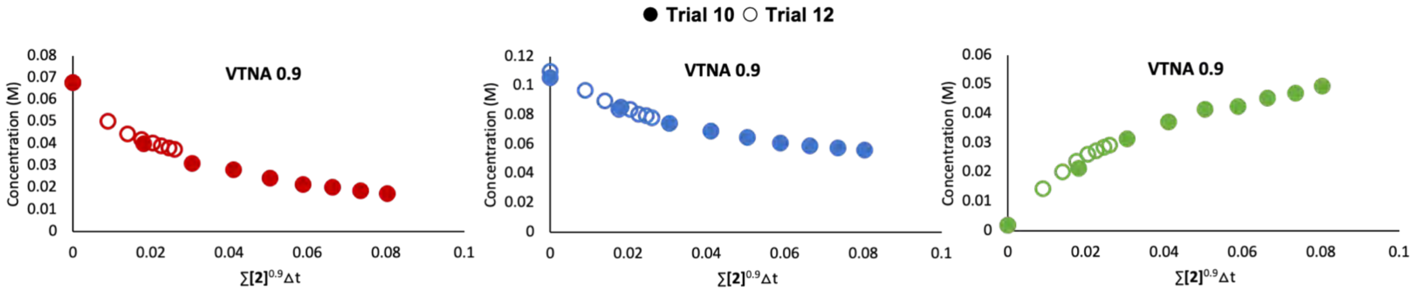

Supplementary Figure 23. Variable time normalization analysis plot to solve for order of 4-methoxyphenyboronic acid pinacol ester (**2**) using data from Supplementary Table 5.

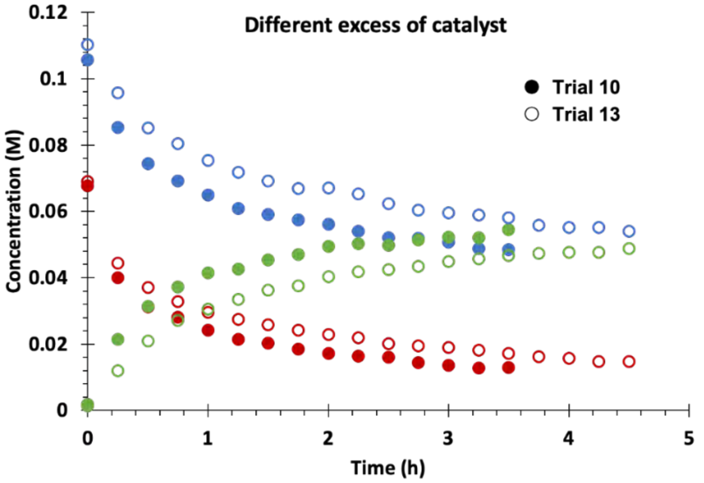

Supplementary Figure 24. Reaction time-course data for the Suzuki-Miyaura reaction with varying concentrations of XPhos Pd G2 in the presence of TBAB.

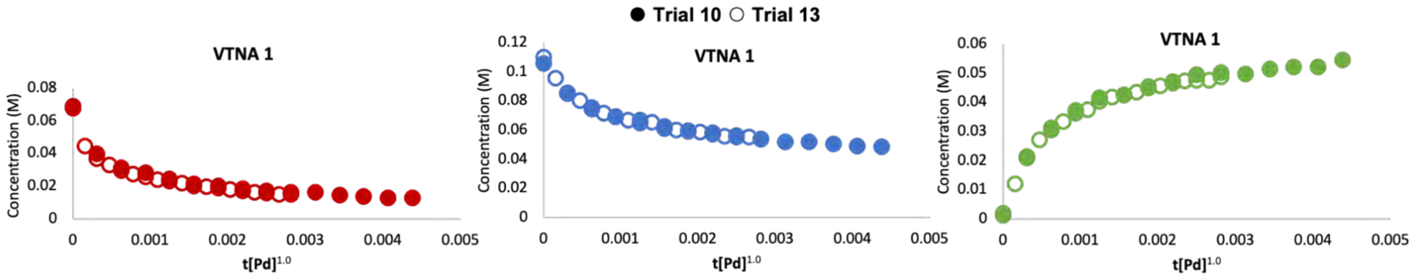

Supplementary Figure 25. Variable time normalization analysis plot to solve for order of XPhos Pd G2 using data from Supplementary Table 5.

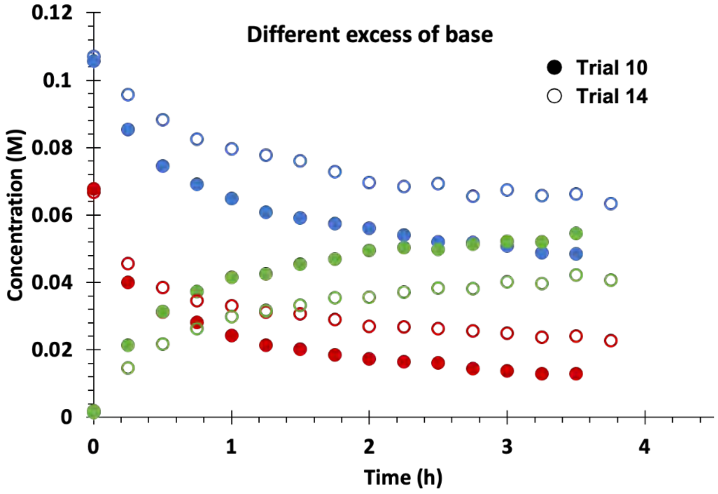

Supplementary Figure 26. Reaction time-course data for the Suzuki-Miyaura reaction with varying concentrations of K_2CO_3 in the presence of TBAB.

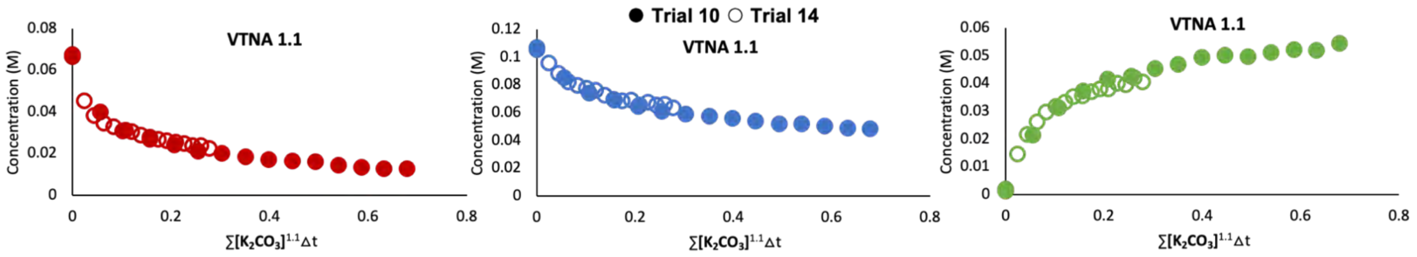

Supplementary Figure 27. Variable time normalization analysis plot to solve for order of K_2CO_3 using data from Supplementary Table 5.

The reaction rates comparison of different organoboron nucleophiles

To an oven-dried 15 mL two-neck pear shaped flask with a magnetic stir bar was added 1,3,5-trimethoxybenzene (84 mg, 0.50 mmol), **2** (175 mg, 0.75 mmol) or **6** (114 mg, 0.75 mmol) or **7** (134 mg, 0.75 mmol) or **8** (165 mg, 0.75 mmol). The flask was sealed with two rubber septa and put on a Schlenk line. The system was evacuated for five minutes and backfilled with argon three times. Meanwhile, a base solution was made by adding K_2CO_3 (1.658 g, 12 mmol) to an oven-dried 9 mL vial. The vial was evacuated for five minutes and backfilled with argon three times before 6.0 mL of water was added. A precatalyst solution was also made by adding XPhos Pd G2 (59 mg, 0.075 mmol) to an oven-dried 3 mL vial. The vial was evacuated for five minutes and backfilled with argon three times before 1.5 mL of MeTHF was added. Then, under a high flow of argon, one septum of the reaction flask was removed and the EasySampler probe was inserted into one neck of the flask. Next, 6 mL of 2-Me-THF followed by benzyl bromide (**1**) (171 mg, 1.0 mmol) were added to the flask. The reaction was heated to 80 °C. After 15 minutes, three test samples were taken from the reaction mixture to ensure reproducible sampling. Finally, 1.0 mL of the catalyst solution, containing XPhos Pd G2 (39.34 mg, 0.05 mmol), 3 mL of K_2CO_3 base solution were added to the flask and the sampling sequence was immediately started.

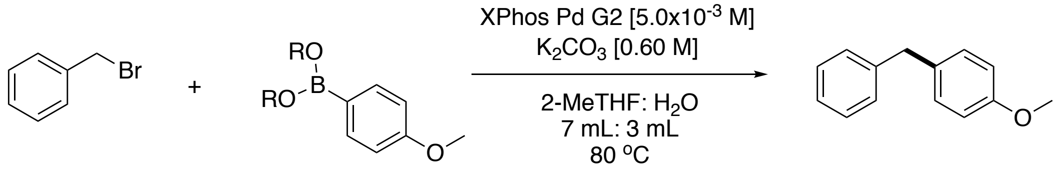

1 **3**

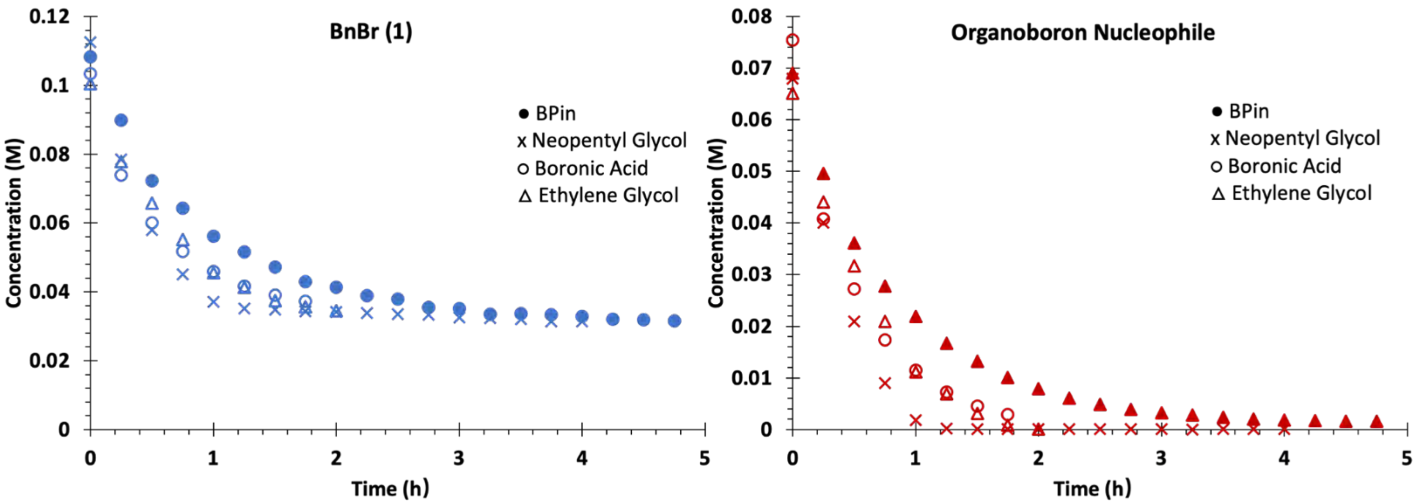

Supplementary Figure 28. Time course data probing the use of **6**, ethylene glycol (**7**), and neopentyl glycol (**8**) to standard conditions. Note: **7** and **8** are hydrolyzed to the parent boronic acid on HPLC and thus were monitored as **6** the latter throughout their respective experiments.

Probing the impact of solvent ratios

To an oven-dried 15 mL two-neck pear shaped flask with a magnetic stir bar was added 1,3,5-trimethoxybenzene (84 mg, 0.50 mmol), 4-methoxyphenylboronic ester (**2**) (175 mg, 0.75 mmol). The flask was sealed with two rubber septa and put on a Schlenk line. The system was evacuated for five minutes and backfilled with argon three times. Meanwhile, a base solution was made by adding K_2CO_3 (1.658 g, 12 mmol) to an oven-dried 9 mL vial. The vial with K_2CO_3 was evacuated for five minutes and backfilled with argon three times before 2X mL of water was added to the vial. A precatalyst solution was also made by adding XPhos Pd G2 (59 mg, 0.075 mmol) to an oven-dried 3 mL vial. The vial with catalyst was evacuated for five minutes and backfilled with argon three times before 1.5 mL of 2-MeTHF was added to the vial. Then, under a high flow of argon, one septum of the reaction flask was removed and the EasySampler probe was inserted into one neck of the flask. Next, 2-MeTHF, benzyl bromide (**1**) (171 mg, 1.0 mmol) were added to the reaction flask under argon. The reaction was heated to 80 °C and stirred at 1200 rpm. After 15 minutes at 80 °C, three test samples were taken from the reaction mixture to ensure reproducible sampling. Finally, 1.0 mL of the catalyst solution, containing XPhos Pd G2 (39.34 mg, 0.05 mmol), X mL of K_2CO_3 base solution containing 6 mmol K_2CO_3 were added to the reaction flask and the sampling sequence was immediately started.

Entry	Aqueous (mL)	Organic (mL)	Total Volume (mL)	Entry	Aqueous (mL)	Organic (mL)	Total Volume (mL)
**1**	1.5	7	8.5	**4**	1.5	8.5	10
**2**	3	7	10	**5**	3	7	10
**3**	4.6 (6)	5.4 (7)	10	**6**	6	4	10

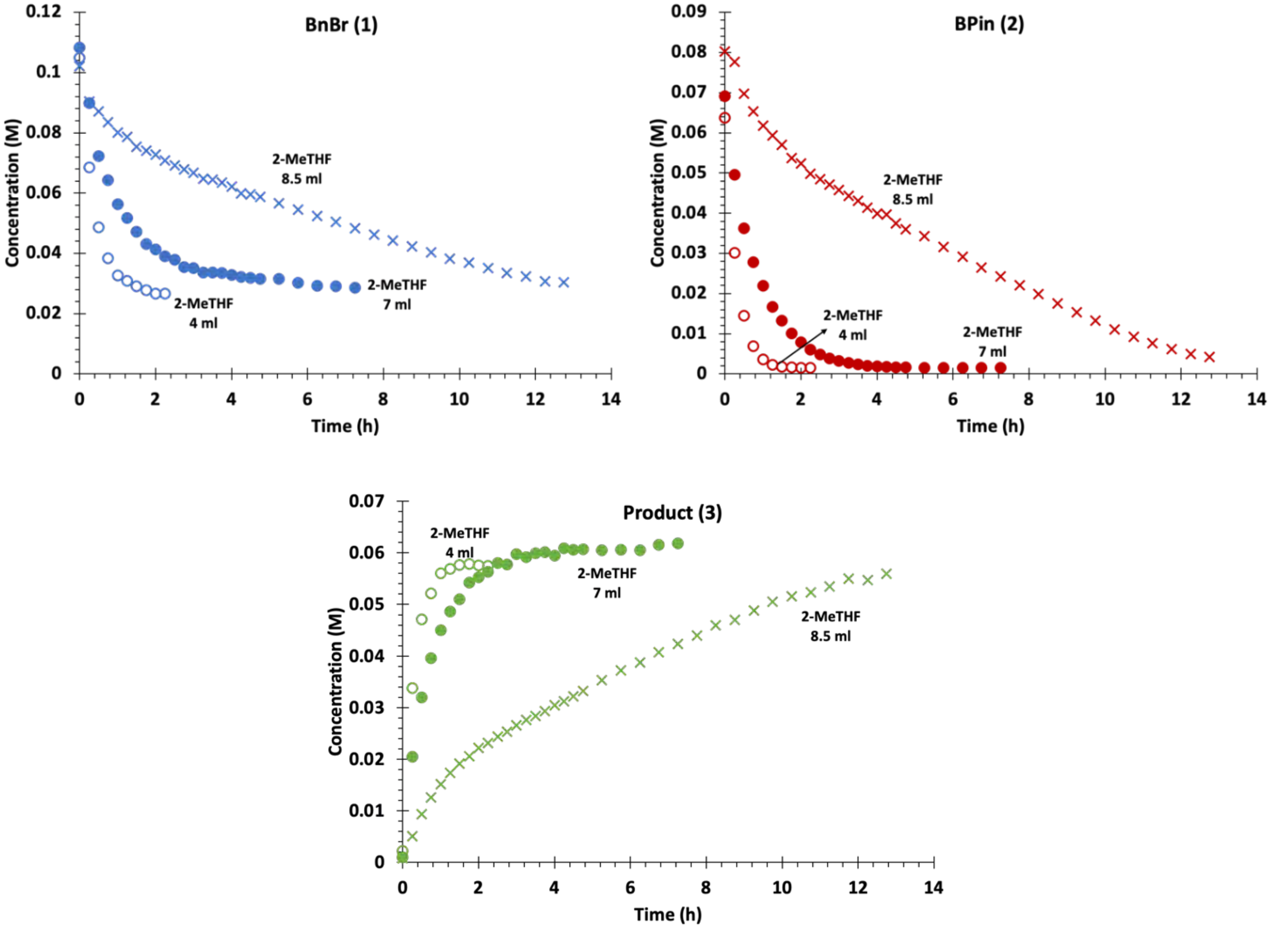

Supplementary Figure 29. Time course probing solvent composition impact on reaction rate

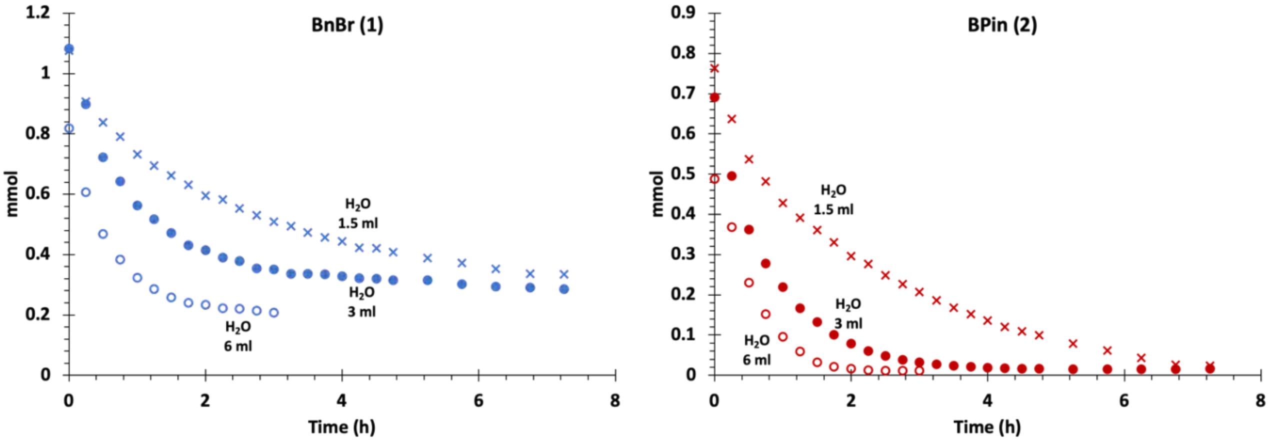

Supplementary Figure 30. Time course overlay probing the effect of different proportions of water while holding the organic phase volume constant. Note: the '6 mL H_2O' reaction was conducted on a reduced scale (4.6 mL H_2O, 5.4 mL 2-MeTHF) to enable the use of the same reaction flask. This avoids any potential kinetic impacts arising from mixing behavior in different flask. Consequently, the mmol of starting material and product are lower than the 3 mL and 1.5 mL counterpart.

Control reactions to check the rate acceleration impact of tetrabutylammonium chloride in C(*sp*²)-C(*sp*²) system

To an oven-dried 15 mL two-neck pear shaped flask with a magnetic stir bar was added 1,3,5-trimethoxybenzene (84 mg, 0.50 mmol) and TBACl (278 mg, 1.0 mmol). The flask was sealed with two rubber septa and put on a Schlenk line. The system was evacuated for five minutes and backfilled with argon three times. Meanwhile, a base solution was made by adding K_2CO_3 (415 mg, 3.0 mmol) to an oven-dried 6 mL vial. The vial with K_2CO_3 was evacuated for five minutes and backfilled with argon three times before 3.0 mL of water was added to the vial. A precatalyst solution was also made by adding SPhos Pd G2 (54 mg, 0.10 mmol) to an oven-dried 3 mL vial. The vial with catalyst was evacuated for five minutes and backfilled with argon three times before 1.5 mL of 2-MeTHF was added to the vial. Then, under a high flow of argon, one septum of the reaction flask was removed and the EasySampler probe was inserted into one neck of the flask. Next, 7.5 mL of 2-MeTHF, 4-fluorophenylboronic ester (**10**) (160 uL, 167 mg, 0.75 mmol) and chlorobenzene (102 uL, 113 mg, 1.0 mmol) were added to the reaction flask under argon. The reaction was stirred at 1200 rpm at room temperature. After 15 minutes, three test samples were taken from the reaction mixture to ensure reproducible sampling. Finally, 1.0 mL of the catalyst solution, containing SPhos Pd G2 (36 mg, 0.05 mmol) and 1.5 mL of K_2CO_3, containing K_2CO_3 (207 mg, 1.5 mmol) were added to the reaction flask and the sampling sequence was immediately started.

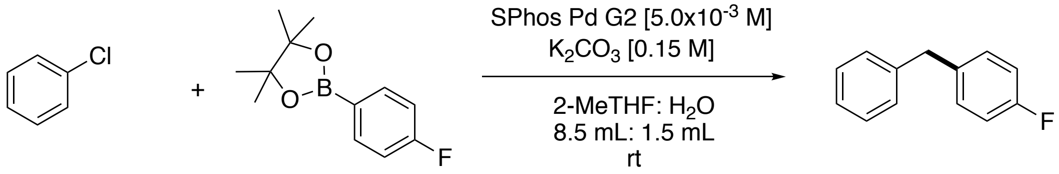

9 **10** **11**

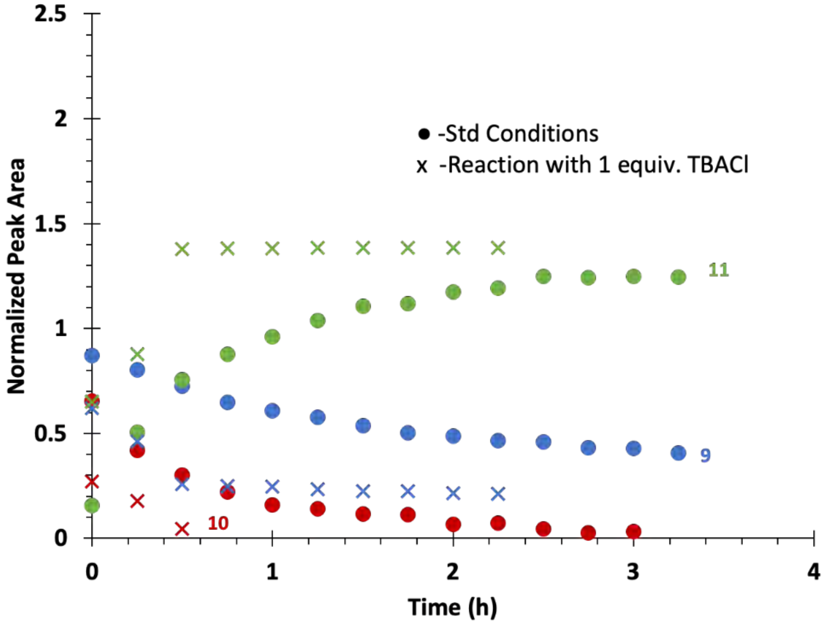

Supplementary Figure 31. The substantial rate acceleration by the addition of TBACl in *sp*^{2}-*sp*^{2} cross-coupling system

Study of Speciation of Organoboron Nucleophile

Synthesis of boronate salt 12 of 4-fluorophenyl boronic pinacol ester

4-Fluorophenyl boronic pinacol ester (**10**) (10.6 ul, 0.05 mmol, 1 equiv.) and TBAOH (120 mg, 0.15 mmol, 3 equiv.) were weighed out into a vial. 1.5 mL THF-d_8 was added and the mixture was stirred until a solution was formed. 1 mL of the solution was transferred to a quartz NMR tube and ^{11}B and ^{19}F NMR spectra were measured.

Synthesis of boronate salt 14 of 4-fluorophenyl boronic acid

4-Fluorophenyl boronic acid (**13**) (7.0 mg, 0.05 mmol, 1 equiv.), and TBAOH (120 mg, 0.15 mmol, 3 equiv.) were weighed out into a vial. 1.5 mL THF-d_8 was added and the mixture was stirred until a solution was formed. 1 mL of the solution was transferred to a quartz NMR tube and ^{11}B and ^{19}F NMR spectra were measured.

Comparison of spectra to procedure from literature precedent

12 and 14 were prepared in D_2O according to the literature procedure[4] and measured by NMR. 1.0 mL THF-d8 was added in the NMR tube to extract the respective boronate salts 12 and 14 from D_2O. Then THF-d_8 extractions were transferred to a quartz NMR tube and ^{11}B and ^{19}F NMR spectra were measured.

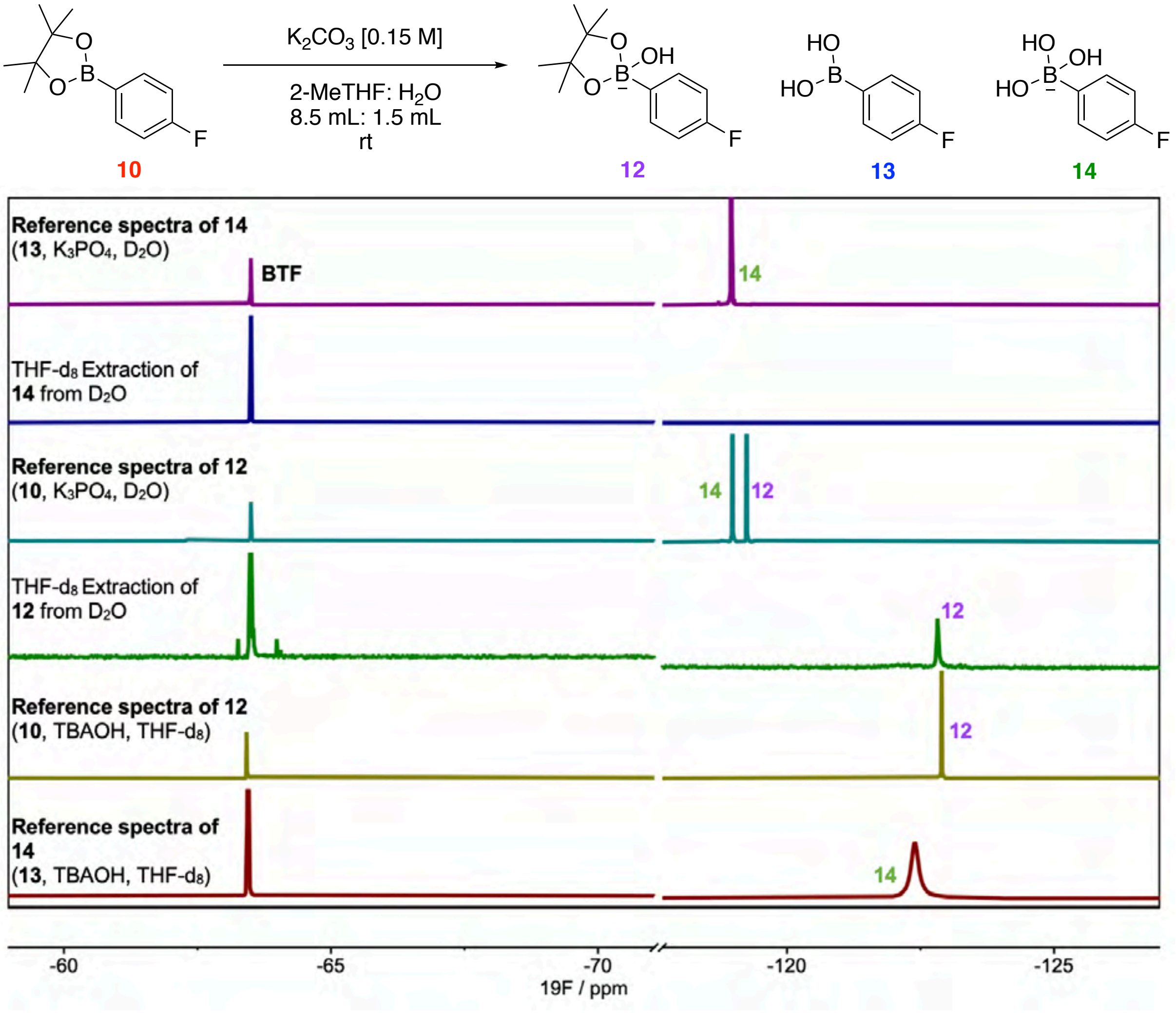

Supplementary Figure 32. Stacked ^{19}F NMR spectra of **12** and **14**.

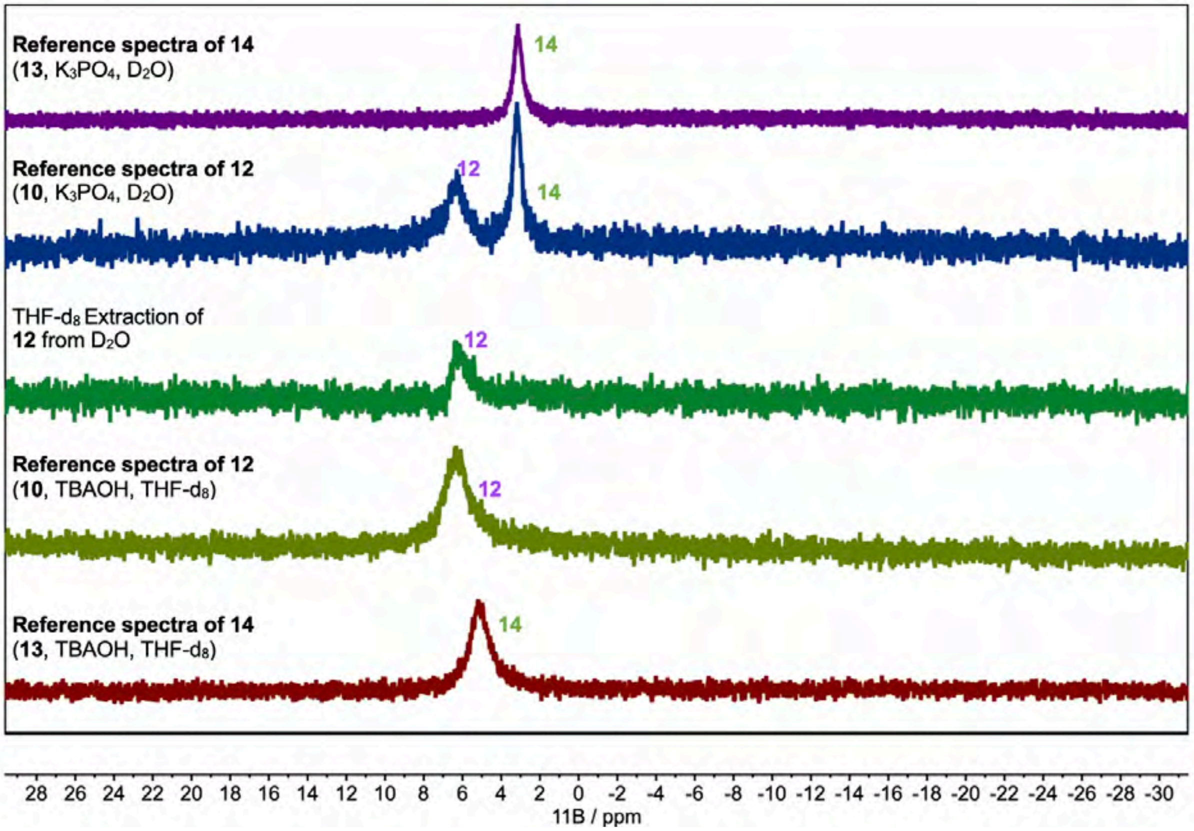

Supplementary Figure 33. Stacked $^{11}B\{^{1}H\}$ NMR spectra of **12** and **14**.

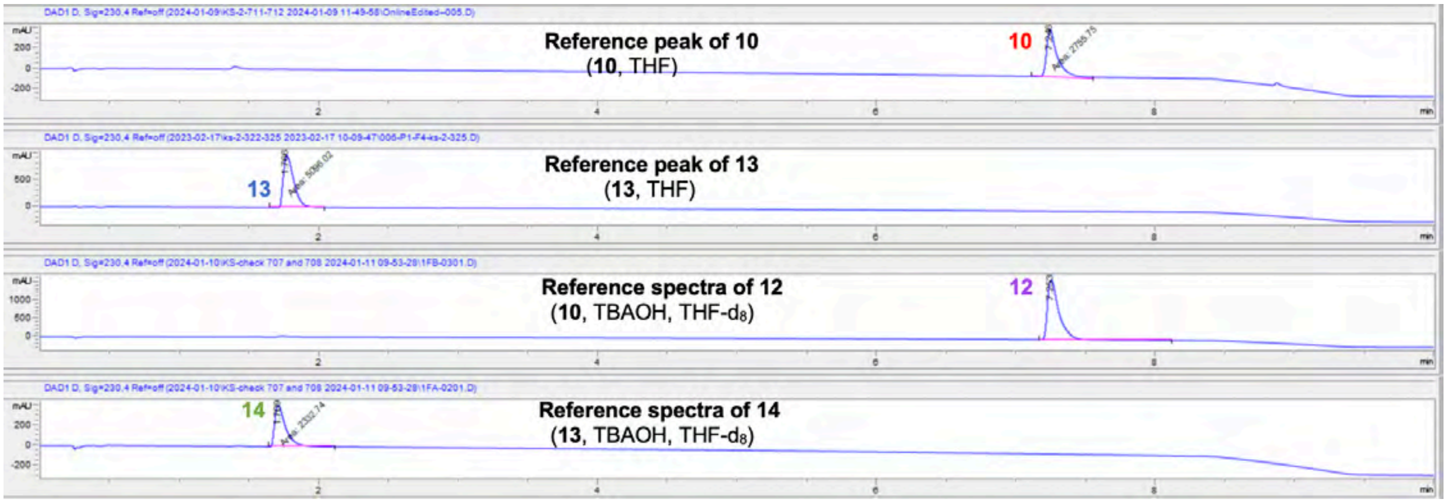

Supplementary Figure 34. Stacked HPLC profiles of standard compounds and their boronate salts.

Discussion on the synthesis of 12 and 14

The synthesis and characterization of boronate salts is difficult due to the ease of speciation including oligomerization and hydrolysis. Consequently, we leveraged a combination of ^{19}F, ^{11}B, and HPLC analysis to support the formation of both **12** and **14**. These were conducted in THF-d_8 to enable the analysis of nucleophile speciation in our biphasic setting. Both parent 6-B-3 compounds **10** and **13** were stirred in THF-d_8 in the presence of excess TBAOH. In both cases, the resulting ^{11}B NMR spectra were consistent with the formation of an 8-B-4 derivative with no detectable signal for the parent

compounds (Supplementary Figure 33). Furthermore, the ^{19}F NMR spectra of both solutions revealed the formation of a single new peak (Supplementary Figure 32). Finally, a sample of each solution was injected onto HPLC which revealed a single peak in each case consistent with their parent 6-B-3 species. This latter result suggests the species formed *in situ* upon exposure to base are readily reverted to **10** and **13** as is expected (Supplementary Figure 34).

To further corroborate our peak assignment, we targeted the synthesis of both **12** and **14** following the procedure recently reported by Lloyd-Jones and Watson.[3] Their procedures formed each respective boronate salt in D_2O which obfuscated a direct comparison to our data obtained in THF-d_8. However, in the case of **12** we were able to obtain NMR data consistent with their reports followed by an extraction of the boronate salt into THF-d_8 (Supplementary Figure 32 and 33). The resulting NMR spectra aligned perfectly with our parent data obtained THF-d_8 with TBAOH. Its worth noting that the competitive hydrolysis of **10** to the boronic acid was observed under these aqueous conditions. When the same procedure was attempted with **13**, we were unable to extract detectable quantities of the boronate salt into the organic phase for subsequent NMR analysis.

Equilibrium between 4-fluorophenyl boronic pinacol ester and its boronate complexes in the presence of potassium chloride.

To three separate oven-dried 15 mL reaction vials with magnetic stir bars were charged with potassium chloride (7.4 mg, 0.1 mmol, 0.13 equiv.), potassium chloride (37.3 mg, 0.5 mmol, 0.66 equiv.), potassium chloride (74.5 mg, 1 mmol, 1.33 equiv.). The reaction vials were sealed with open top screw caps fitted with a Teflon septum and attached to a Schlenk line *via* needles. The reaction vials were evacuated for five minutes and backfilled with argon three times. Meanwhile, a base stock solution was made by adding K_2CO_3 (1.658 g, 12 mmol, 16 equiv.) to an oven-dried 15 mL vial. The vial with K_2CO_3 was evacuated *via* a needle for five minutes and backfilled with argon three times before 12.0 mL of water was added. Then, 8.5 ml of 2-MeTHF, 4-fluorophenyl boronic pinacol ester (**10**) (159 μl, 0.75 mmol, 1 equiv.) and 1.5 mL of base solution containing 207.3 mg K_2CO_3 (1.5 mmol, 2 equiv.) were added through syringe to the reaction vials under argon. The reactions were stirred at room temperature for 20 minutes. After separation of the phases, 0.5 mL of each organic phase, 0.1 mL THF-d8 and 2,4-dichlorobenzotrifluoride (BTF) (2 ml, 0.0138 mmol) as the internal standard were added to the NMR tubes for analysis. The equilibrium between 4-fluorophenyl boronic pinacol ester and its boronate complexes was monitored *via* ^{19}F NMR and ^{11}B NMR spectroscopy.

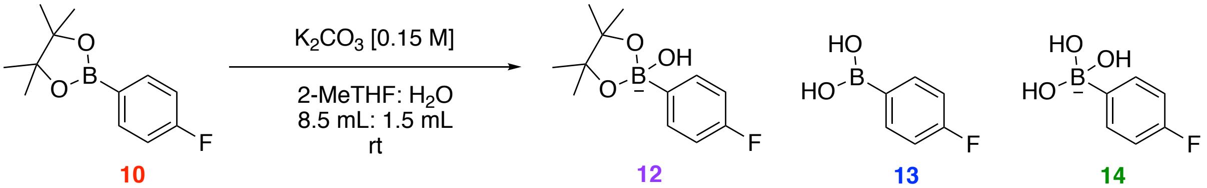

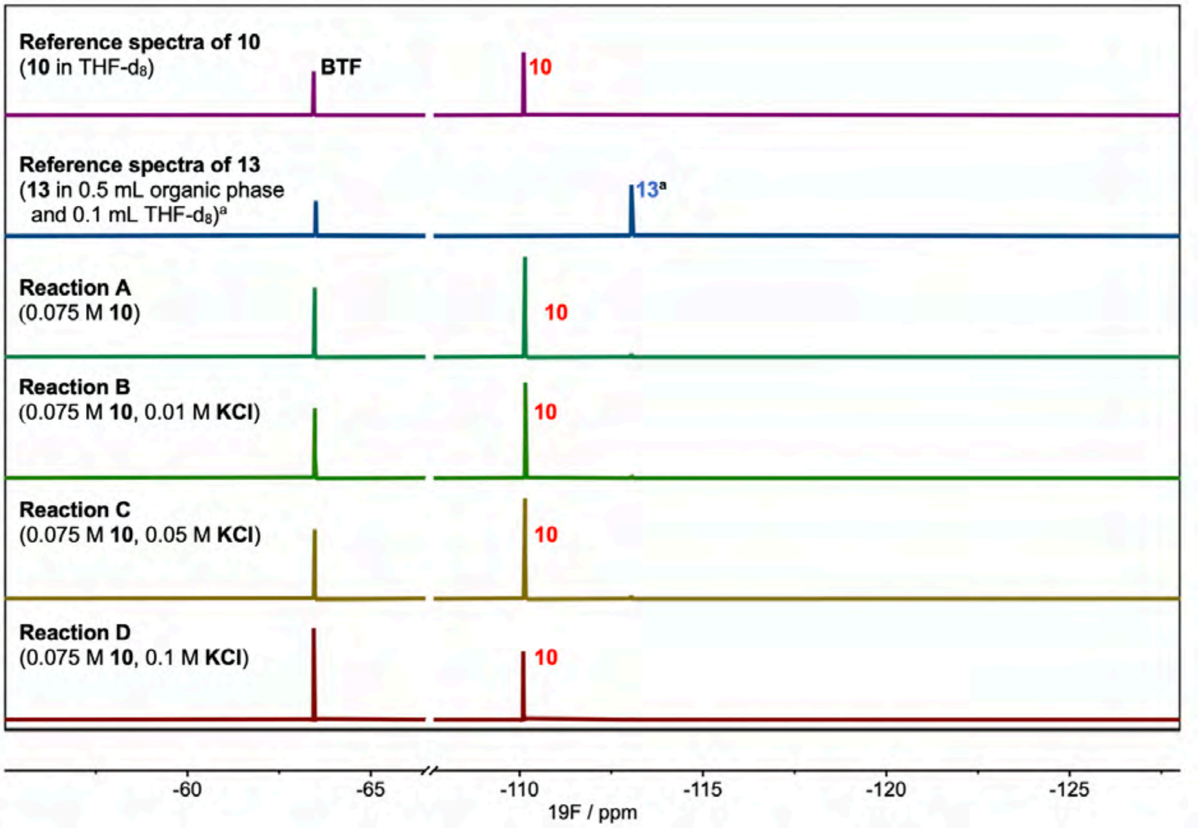

a. The ^{19}F NMR chemical shifts for aryl fluorides can be pH dependent,[5] consequently NMR spectra of **10** and **13** under basic conditions were measured for more accurate comparison to reaction mixtures. (8.5 mL 2-Me-THF and 1.5 mL 0.15 M K_2CO_3 biphasic reaction solution was prepared, then 0.5 mL of the organic phase was taken and used to dissolve **10** and **13** separately. These solutions were transferred into NMR tubes with 0.1 mL THF-d_8 for analysis. The impact of organic phase basicity on the resulting chemical shift of **10** was negligible and thus is not shown in the figure above. The difference in chemical shift of **13** was significantly impacted and is shown above as a reference for the analysis.)

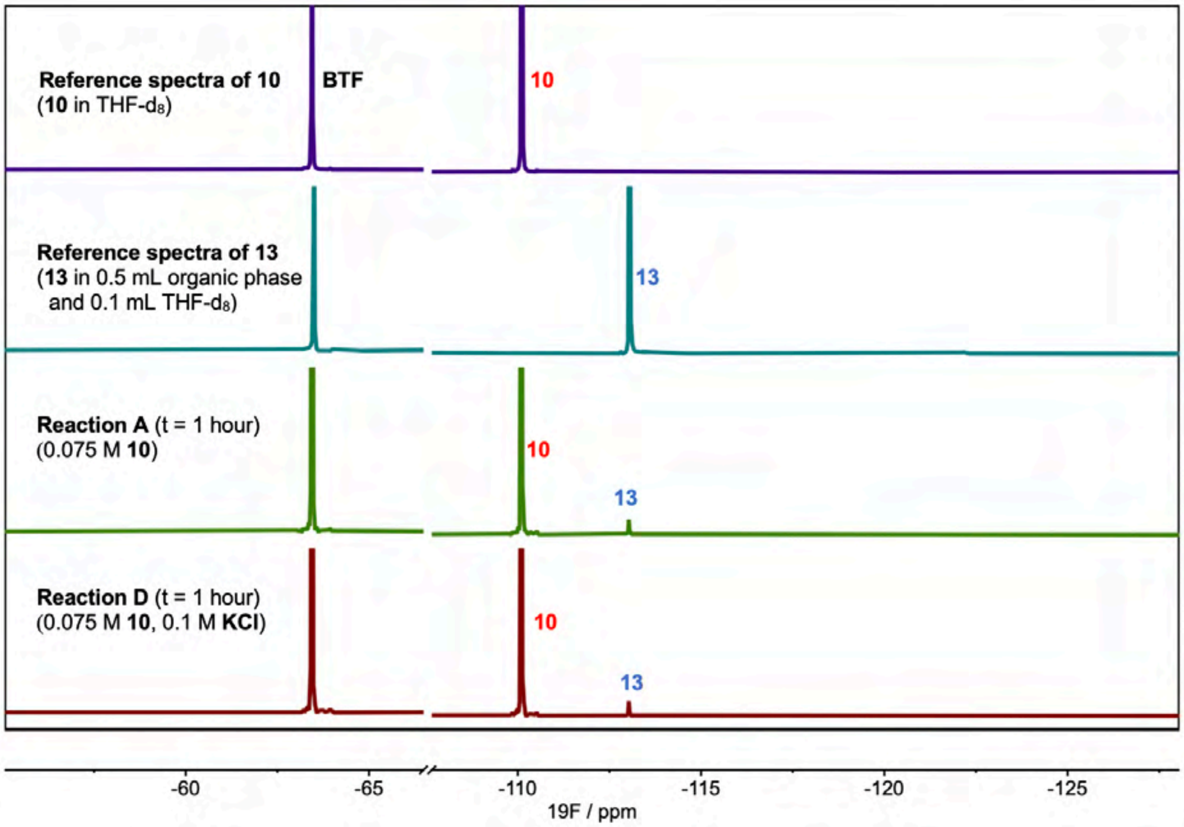

Supplementary Figure 35. Stacked ^{19}F NMR spectra of organic phase aliquots.

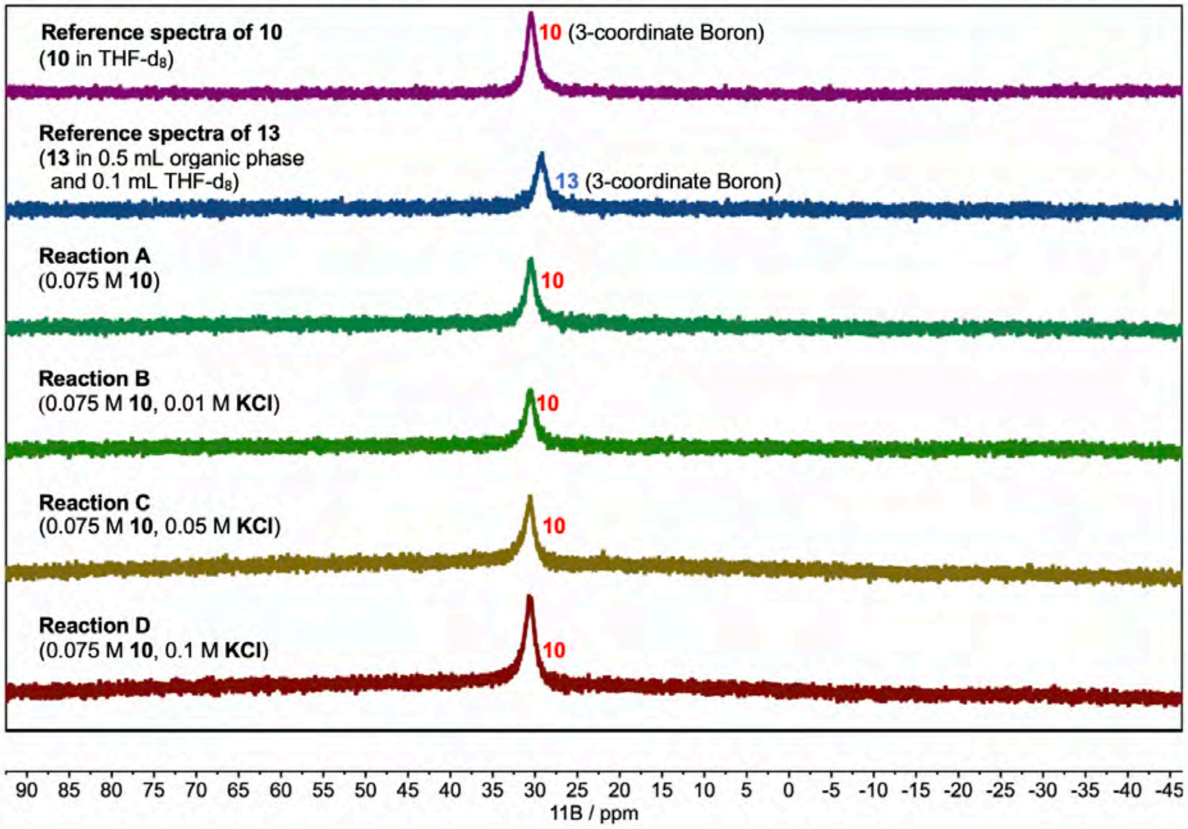

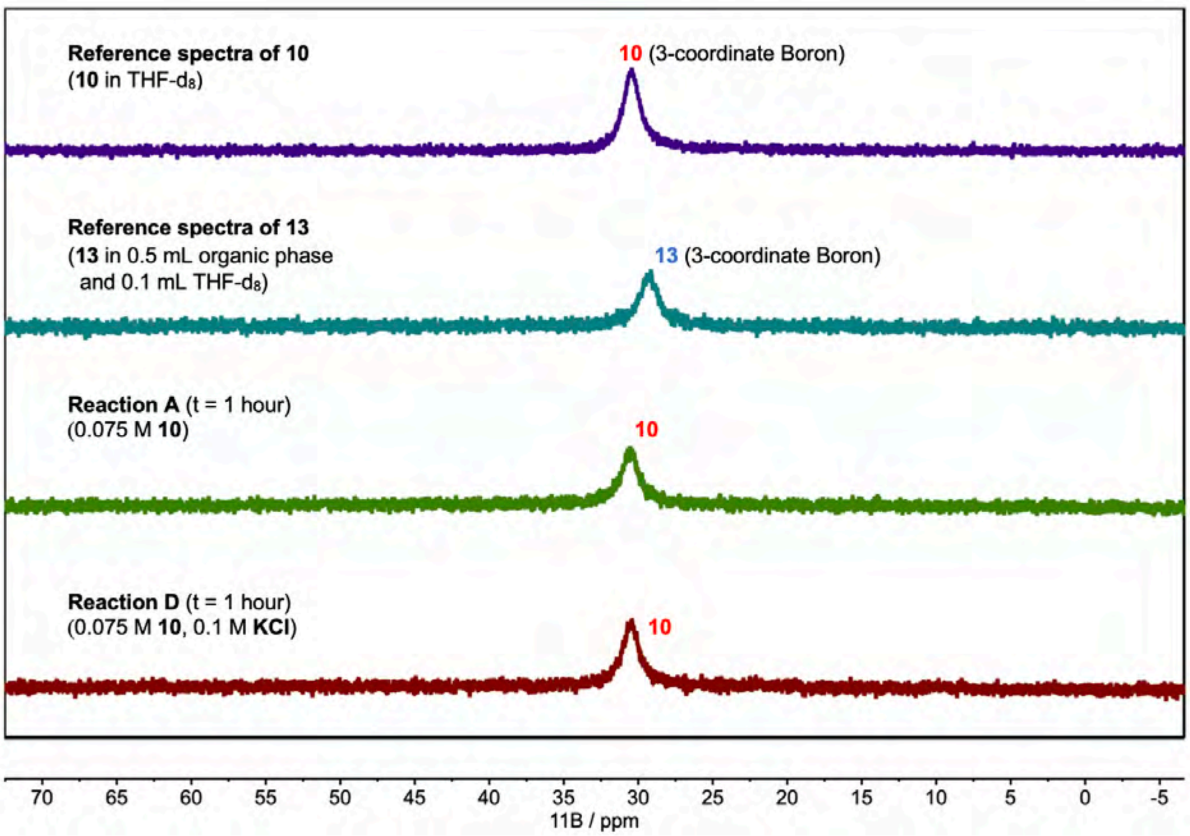

Supplementary Figure 36. Stacked $^{11}B\{^1H\}$ NMR spectra of organic phase aliquots

Conclusion from boron equilibration experiment in the presence of KCl:

Under standard reaction conditions without the addition of the KCl additive, the organic phase is made up predominately of **10** with small amounts of boronic acid **13** both present as 6-B-3 species. This is further corroborated by ^{11}B NMR which lacked any signal representative of 8-B-4 species. Upon sequential additions of KCl to the solution, no significant change occurred in the ^{19}F NMR spectra. Moreover, no 4-coordinate boronate salt of **10** could be detected by ^{11}B NMR. These reactions were shown to have reached equilibrium prior to analysis by taking a second spectra after one hour with no significant change save for a slight increase of **13** as a result of background hydrolysis. Overall these results suggest that potassium salts have minimal impact on boron speciation. In contrast, similar experiments conducted with SPhosPd(Ph)(OH) by Milner et al.[6] has shown potassium salts to significantly shift this equilibrium towards SPhosPd(Ph)(X) where KI showed the greatest impact while KCl showed the smallest. Taken together, these data strongly support that path B is active in the absence of TBA additives and that the inhibitory impact of halides in such settings is a consequence of their impact on palladium speciation towards the inactive SPhosPd(Ph)(X).

Equilibrium between 4-fluorophenyl boronic pinacol ester and its boronate complexes in the presence of tetrabutylammonium chloride.

To three separate oven-dried 15 mL reaction vials with magnetic stir bars were charged with tetrabutylammonium chloride (27.8 mg, 0.1 mmol, 0.13 equiv.), tetrabutylammonium chloride (138.9 mg, 0.5 mmol, 0.66 equiv.), tetrabutylammonium chloride (277.9 mg, 1 mmol, 1.33 equiv.). The reaction vials were sealed with open top screw caps fitted with a Teflon septum and attached to a Schlenk line *via* needles. The reaction vials were evacuated for five minutes and backfilled with argon three times. Meanwhile, a base stock solution was made by adding K_2CO_3 (1.658 g, 12 mmol, 16 equiv.) to an oven-dried 15 mL vial. The vial with K_2CO_3 was evacuated *via* a needle for five minutes and backfilled with argon three times before 12.0 mL of water was added. Then, 8.5 ml of 2-MeTHF, 4-fluorophenyl boronic pinacol ester (**10**) (159 ul, 0.75 mmol, 1 equiv.) and 1.5 mL of base solution containing 207.3 mg K_2CO_3 (1.5 mmol, 2 equiv.) were added through syringe to the reaction vials under argon. The reactions were stirred at room temperature for 20 minutes. After separation of the phases, 0.5 mL of each organic phase and 0.1 mL THF-d_8 and 2,4-dichlorobenzotrifluoride (BTF) (2 μl, 0.0138 mmol) were added to the NMR tubes for NMR analysis. The equilibrium between 4-fluorophenyl boronic pinacol ester and its boronate complex was monitored *via* ^{19}F NMR and ^{11}B NMR spectroscopy. The concentration of **10** and **13** were determined by ^{19}F qNMR using 2,4-dichlorobenzotrifluoride as the external standard.

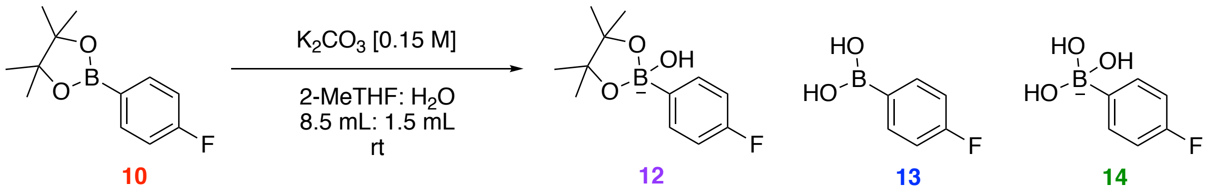

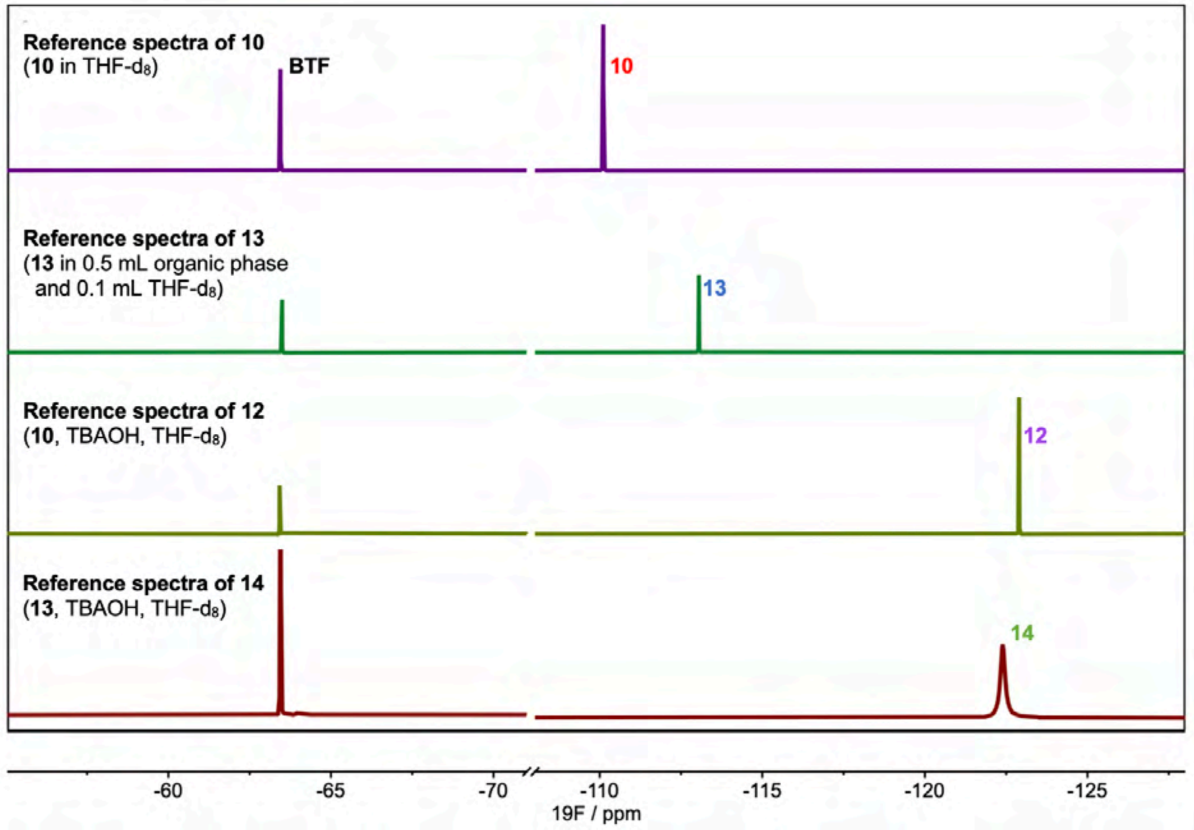

Supplementary Figure 37. Stacked ^{19}F NMR spectra of reference spectra

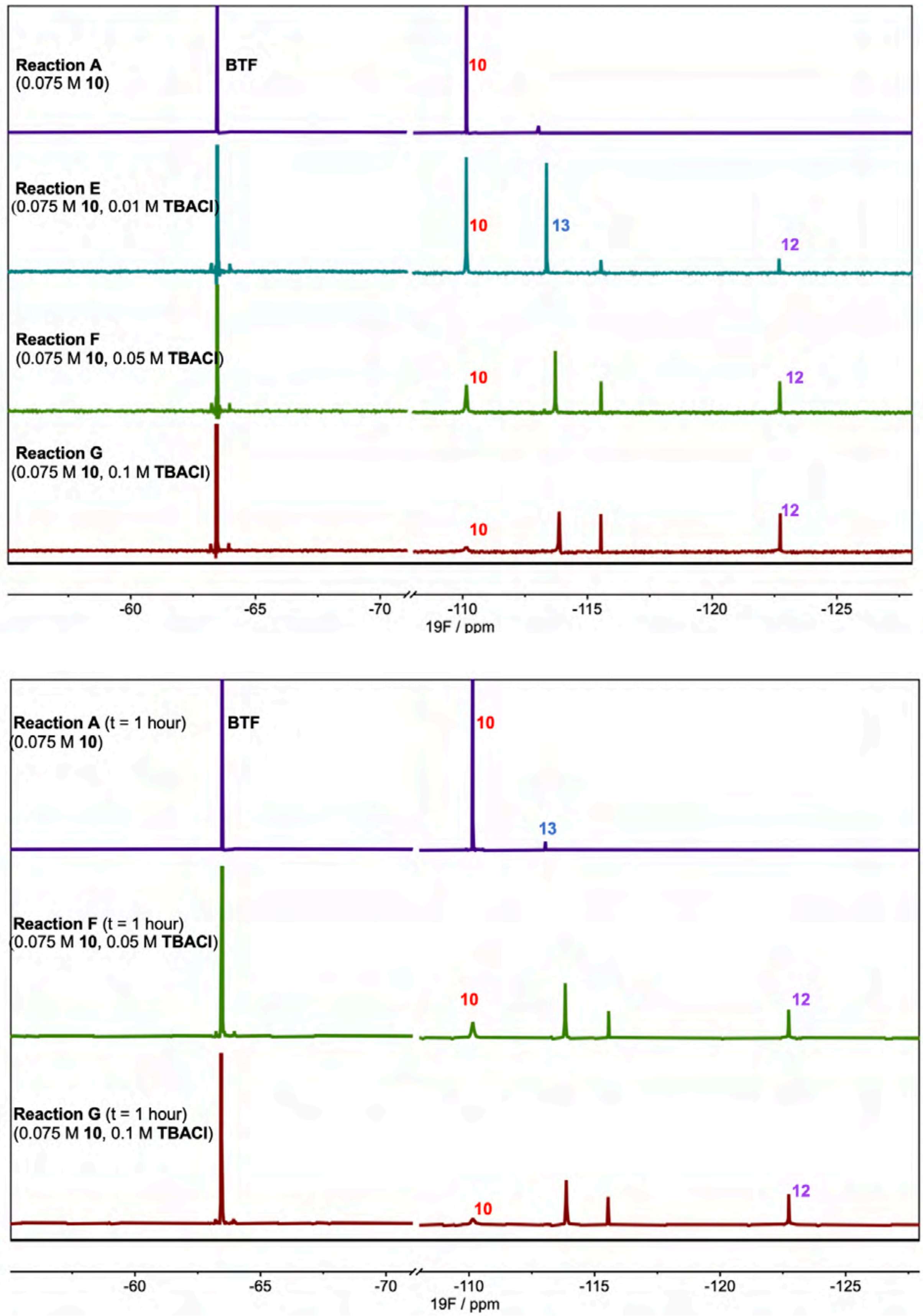

Supplementary Figure 38. Stacked ^{19}F NMR spectra of organic phase aliquots

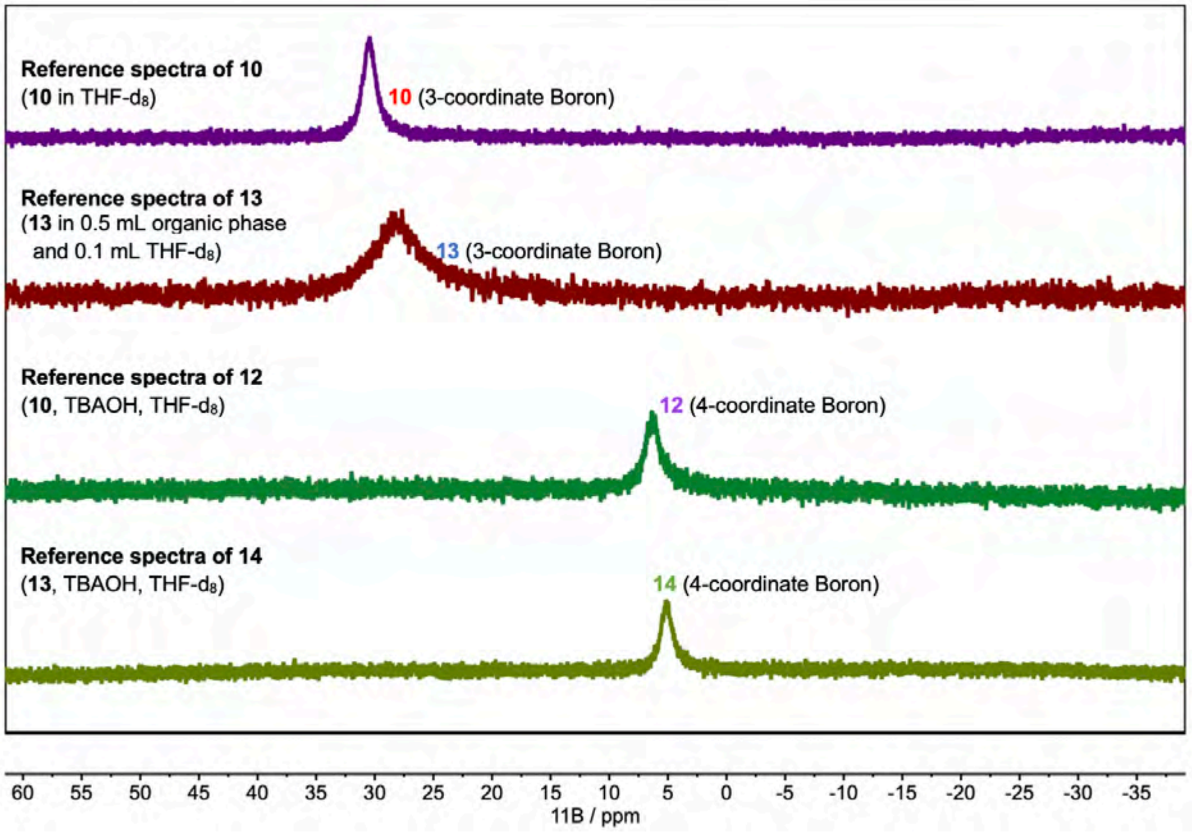
Reference spectra of 10
(10 in THF-d8)
10 (3-coordinate Boron)
Reference spectra of 13
(13 in 0.5 mL organic phase
and 0.1 mL THF-d8)
13 (3-coordinate Boron)
Reference spectra of 12
(10, TBAOH, THF-d8)
12 (4-coordinate Boron)
Reference spectra of 14
(13, TBAOH, THF-d8)
14 (4-coordinate Boron)
60 55 50 45 40 35 30 25 20 15 10 5 0 -5 -10 -15 -20 -25 -30 -35
11B / ppm

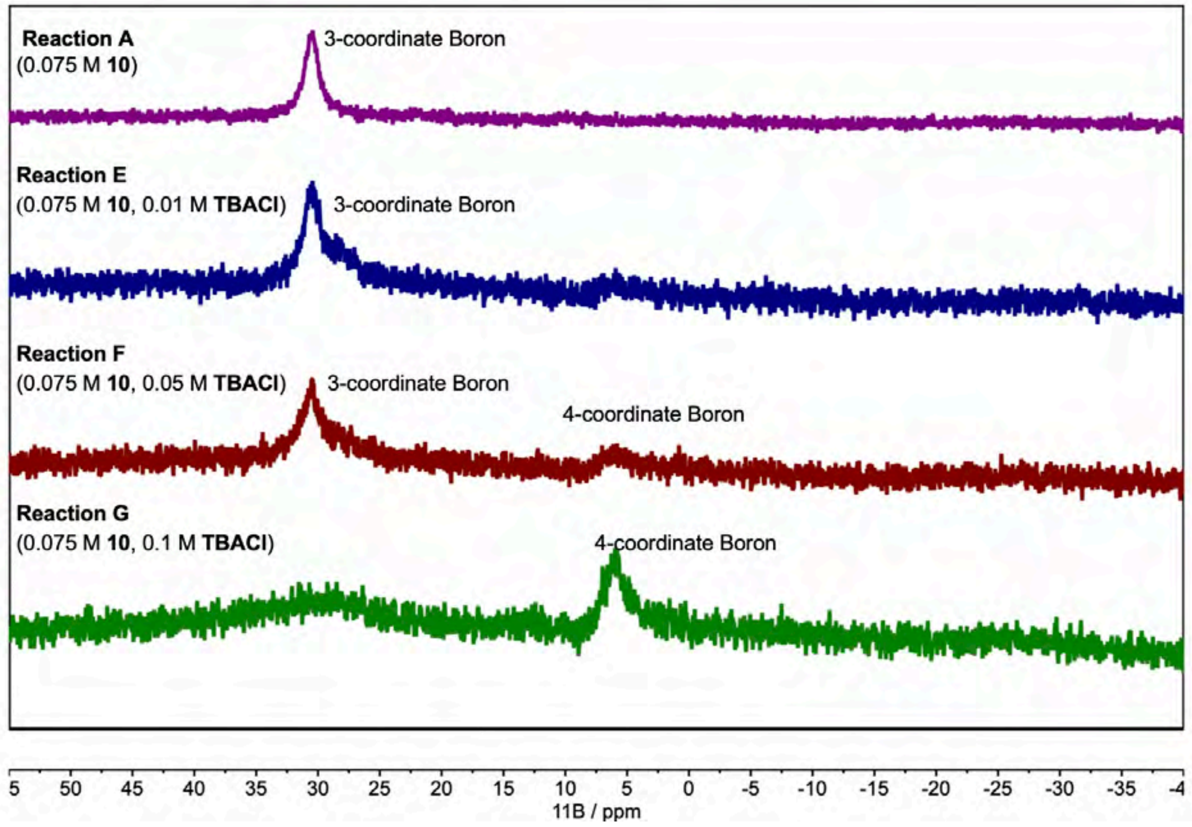
Reaction A
(0.075 M 10)
3-coordinate Boron
Reaction E
(0.075 M 10, 0.01 M TBACl)
3-coordinate Boron
Reaction F
(0.075 M 10, 0.05 M TBACl)
3-coordinate Boron
4-coordinate Boron
Reaction G
(0.075 M 10, 0.1 M TBACl)
4-coordinate Boron
5 50 45 40 35 30 25 20 15 10 5 0 -5 -10 -15 -20 -25 -30 -35 -4
11B / ppm

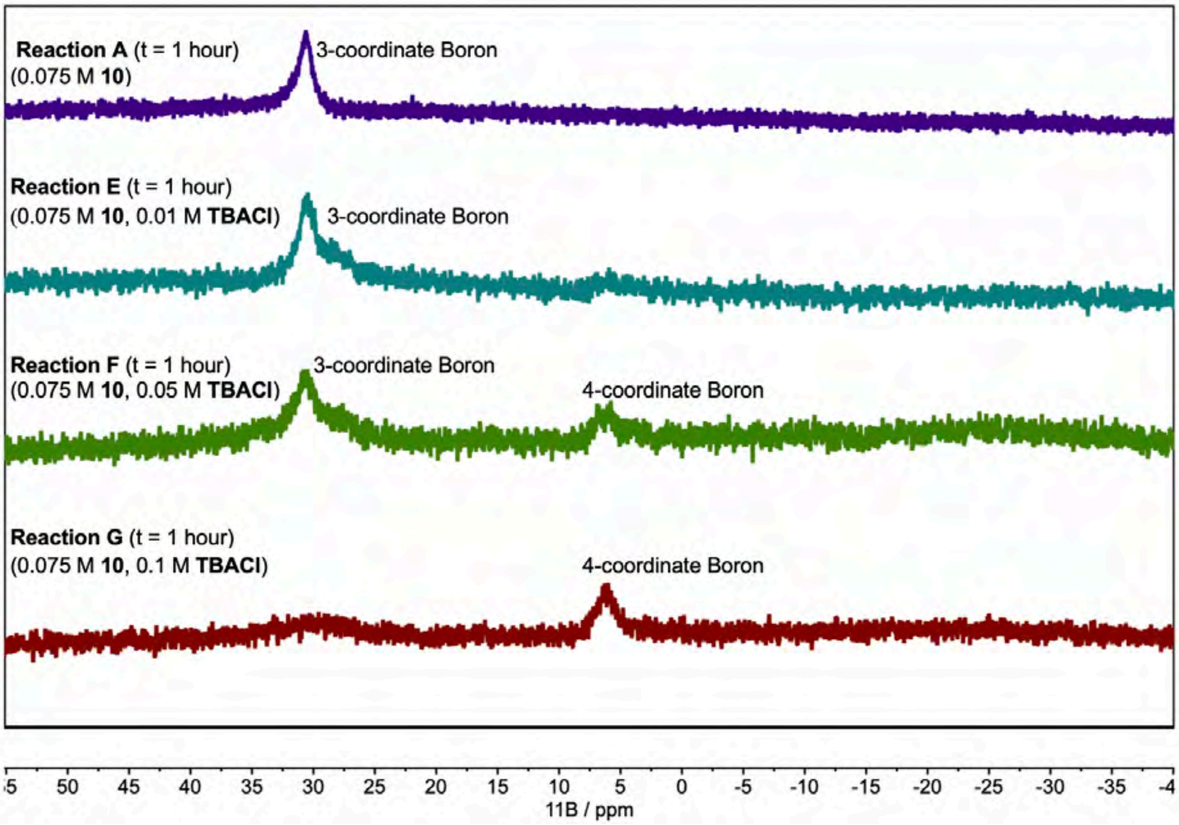

Supplementary Figure 39. Stacked $^{11}B\{^{1}H\}$ NMR spectra of standard compounds and organic phase aliquots

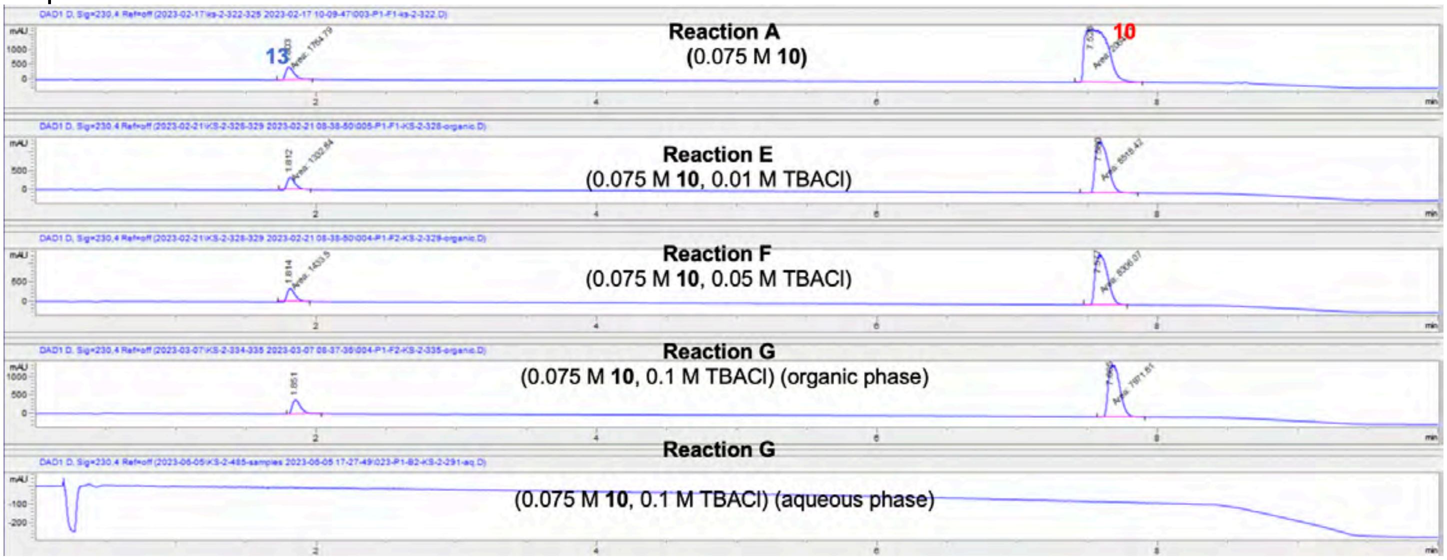

Supplementary Figure 40. Stacked HPLC profiles of organic phase aliquots

Conclusion from boron equilibration experiment in the presence of TBACl:

Under standard reaction conditions without the addition of the TBACl additive, the organic phase is made up predominately of **10** with small amounts of boronic acid **13** both present as 6-B-3 species. This is further corroborated by ^{11}B NMR which lacked any signal representative of 8-B-4 species. Upon sequential additions of TBACl to the solution, increasing amounts of the 4-coordinate boronate salt of **10** is observed. This is consistent with both ^{11}B and ^{19}F NMR spectra. Although some unknowns are present within the ^{19}F NMR, analysis of each sample *via* HPLC reveal only the presence of **10** and **13**

suggesting these unknowns are equilibrium species of the parent 3- or 4-coordinate species. Moreover, neither **10** or **13** were detectable in the aqueous phase by HPLC analysis. These results coupled with the impact of TBACl on catalyst speciation (see below) strongly support a transmetalation dominated by path A in the presence of TBA additives.

Study of Speciation of Palladium Catalyst

Equilibrium between SPhos-Pd(Ph)Cl [0.02 M] and SPhos-Pd(Ph)OH complexes

15 and **16** were synthesized according to the prior literature procedure, and the measured NMR data are consistent with the previously reported data.[7] To two separate oven-dried 2 mL reaction vials with magnetic stir bars were both charged with SPhos-Pd(Ph)Cl (12.56 mg, 0.02 mmol, 1 equiv.). One vial was added TBACl (55.6 mg, 0.2 mmol, 10 equiv.). Then two reaction vials were sealed with open top screw caps fitted with a Teflon septum and attached to a Schlenk line via needles. The reaction vials were evacuated for five minutes and backfilled with argon three times. Meanwhile, a base stock solution was made by adding K_2CO_3 (165.8 mg, 1.2 mmol, 120 equiv.) to an oven-dried 2 mL vial. The vial with K_2CO_3 was evacuated *via* a needle for five minutes and backfilled with argon three times before 0.6 mL of water was added. Then, 0.85 ml of 2-MeTHF and 0.15 mL of base solution containing 41.4 mg K_2CO_3 (0.3 mmol, 30 equiv.) were added through syringe to the reaction vials under argon. The reactions were stirred at room temperature for 20 minutes, and stopped to allow phase separation. 0.5 mL of each organic phase and 0.1 mL THF-d8 were added to the NMR tubes for NMR analysis. The equilibrium between **15** and **16** complexes was monitored by ^{31}P NMR spectroscopy.

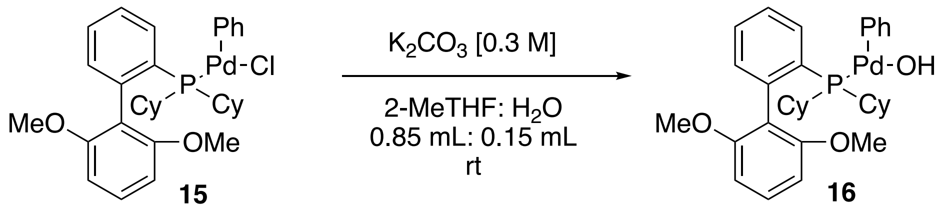

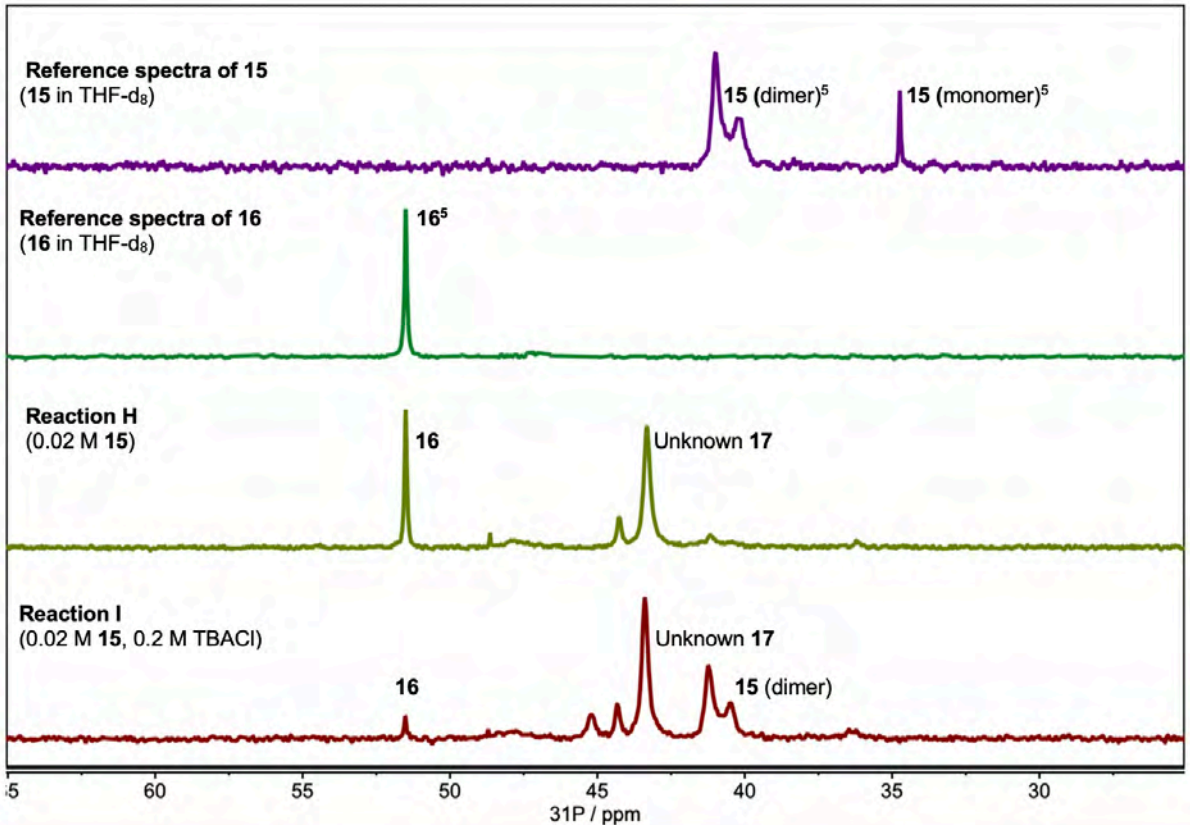

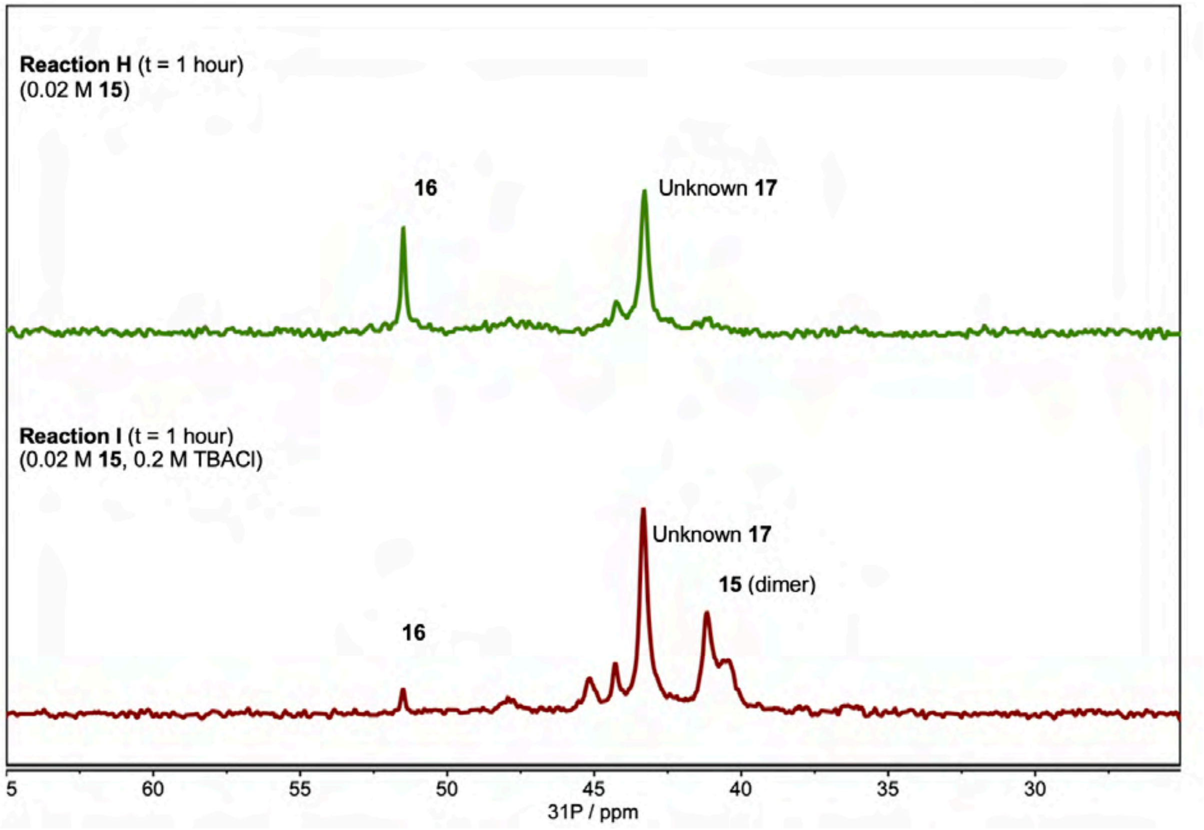

Supplementary Figure 41. Stacked $^{31}P\{^{1}H\}$ spectrum of standard compounds and organic phase aliquots

Equilibrium between SPhos-Pd(Ph)Cl [0.01 M] and SPhos-Pd(Ph)OH

To two separate oven-dried 2 mL reaction vials with magnetic stir bars were both charged with SPhos-Pd(Ph)Cl (12.56 mg, 0.02 mmol, 1 equiv.). One vial was added tetrabutylammonium chloride (55.6 mg, 0.2 mmol, 10 equiv.). Then two reaction vials were sealed with open top screw caps fitted with a Teflon septum and attached to a Schlenk line via needles. The reaction vials were evacuated for five minutes and backfilled with argon three times. Meanwhile, a base stock solution was made by adding K_2CO_3 (165.8 mg, 1.2 mmol, 120 equiv.) to an oven-dried 2 mL vial. The vial with K_2CO_3 was evacuated *via* a needle for five minutes and backfilled with argon three times before 0.6 mL of water was added. Then, 0.85 ml of 2-MeTHF and 0.15 mL of base solution containing 41.4 mg K_2CO_3 (0.3 mmol, 30 equiv.) were added through syringe to the reaction vials under argon. The reactions were stirred at room temperature for 20 minutes, and stopped to allow phase separation. 0.5 mL of each organic phase and 0.1 mL THF-d_8 were added to the NMR tubes for NMR analysis. The equilibrium between **15** and **16** complexes was monitored by ^{31}P NMR spectroscopy. The concentration of each palladium species was obtained using triphenyl phosphate as ^{31}P qNMR external standard.

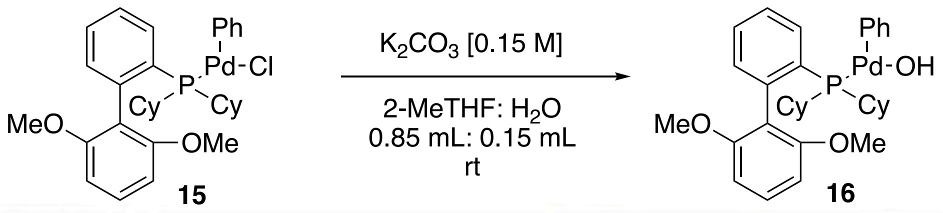
Ph
Pd-Cl
P
Cy
Cy
MeO
OMe
15
K2CO3 [0.15 M]
2-MeTHF: H2O
0.85 mL: 0.15 mL
rt
Ph
Pd-OH
P
Cy
Cy
MeO
OMe
16

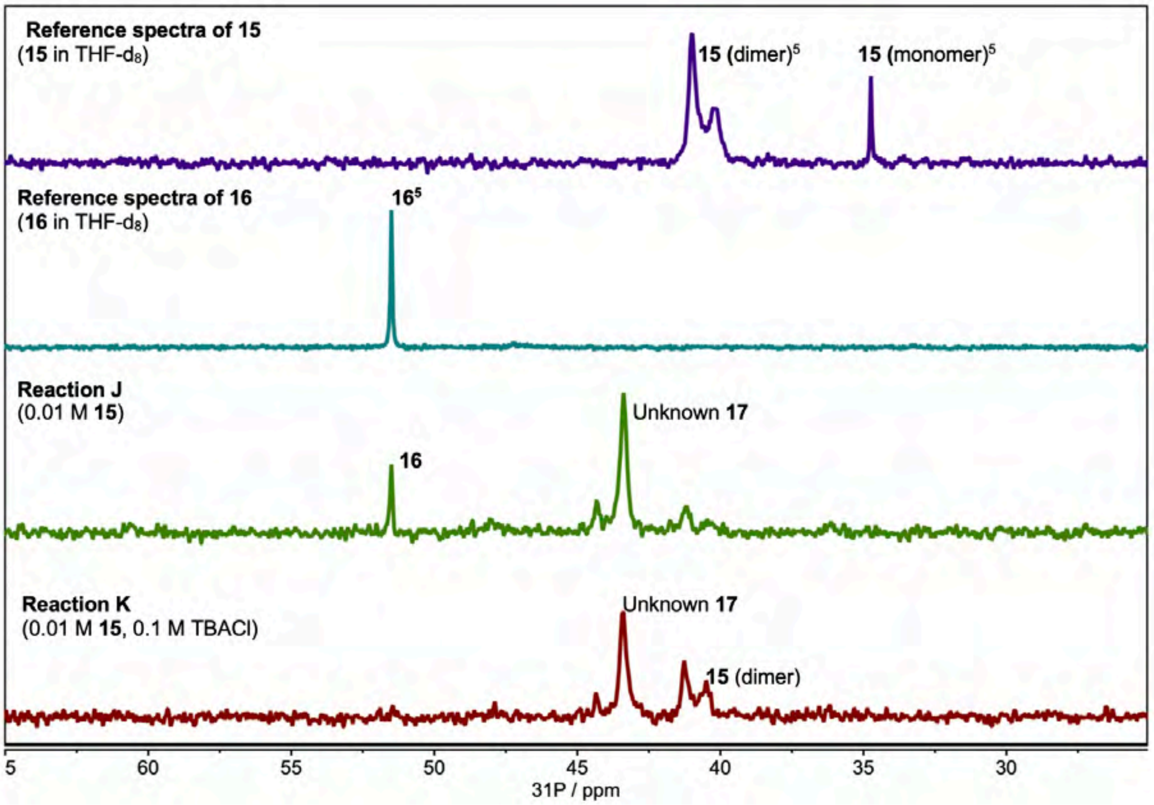
Reference spectra of 15
(15 in THF-d8)
15 (dimer)5
15 (monomer)5
Reference spectra of 16
(16 in THF-d8)
165
Reaction J
(0.01 M 15)
16
Unknown 17
Reaction K
(0.01 M 15, 0.1 M TBACl)
Unknown 17
15 (dimer)
5
60
55
50
45
40
35
30
31P / ppm

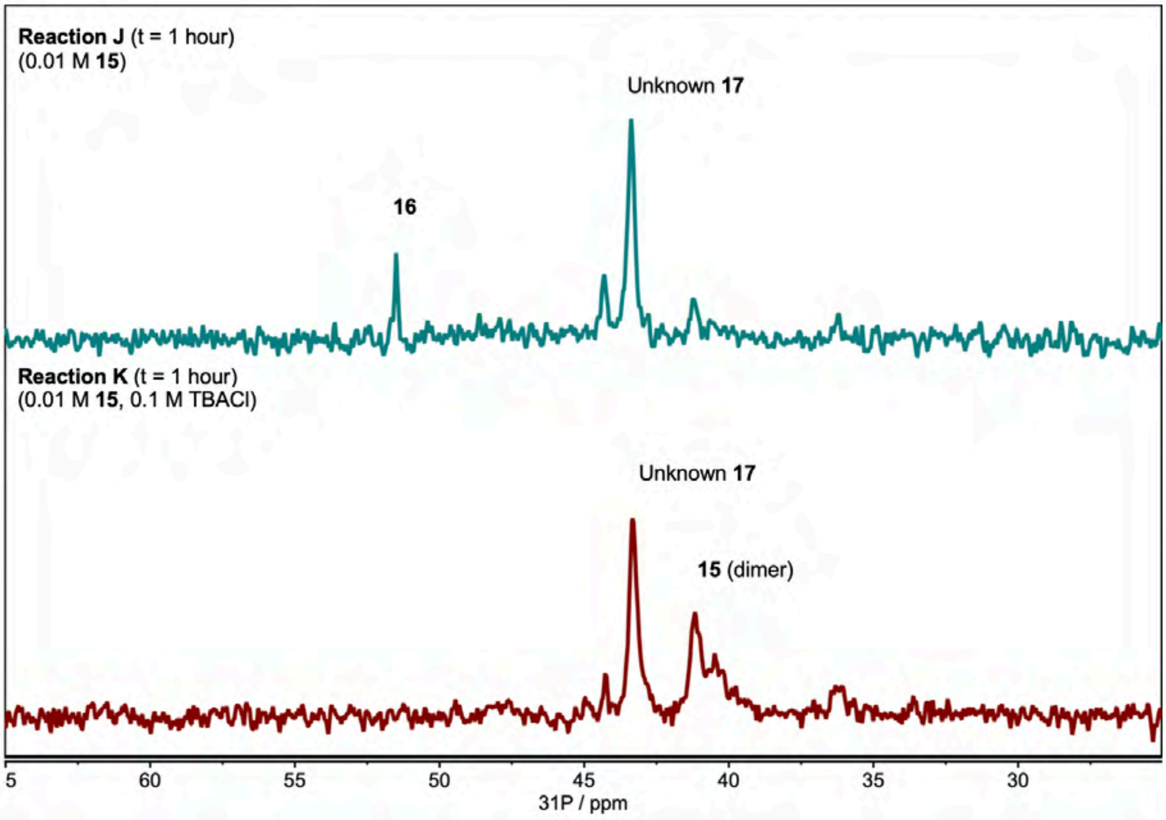
Reaction J (t = 1 hour)
(0.01 M 15)
Unknown 17
16
Reaction K (t = 1 hour)
(0.01 M 15, 0.1 M TBACl)
Unknown 17
15 (dimer)
5
60
55
50
45
40
35
30
31P / ppm

Supplementary Figure 42. Stacked $^{31}P\{^{1}H\}$ spectrum of standard compounds and organic phase aliquots

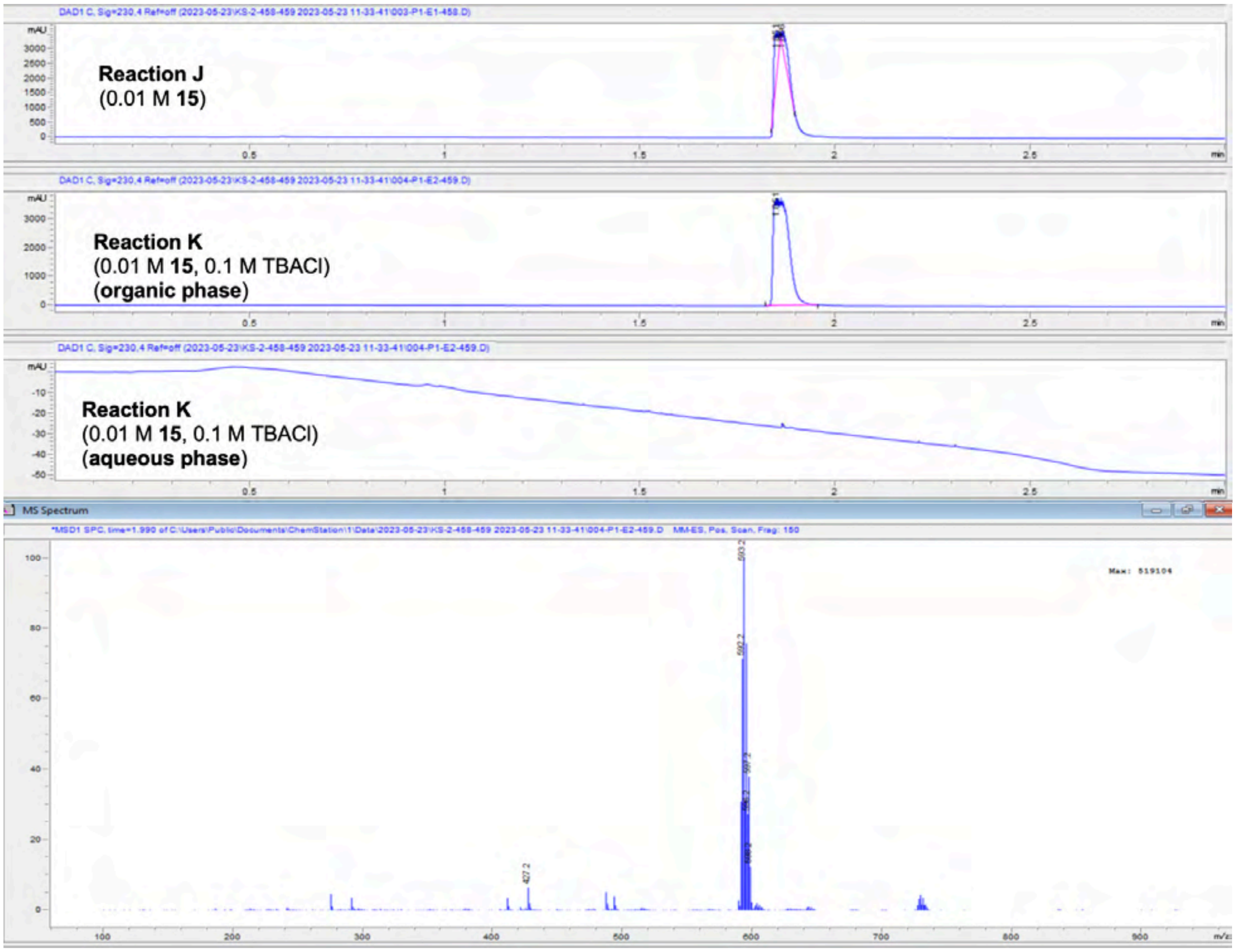

Supplementary Figure 43. HPLC profiles and MS spectrum of reaction aliquots

Dosing experiments (Equilibration between SPhos-Pd(Ph)Cl and SPhos-Pd(Ph)OH complexes)

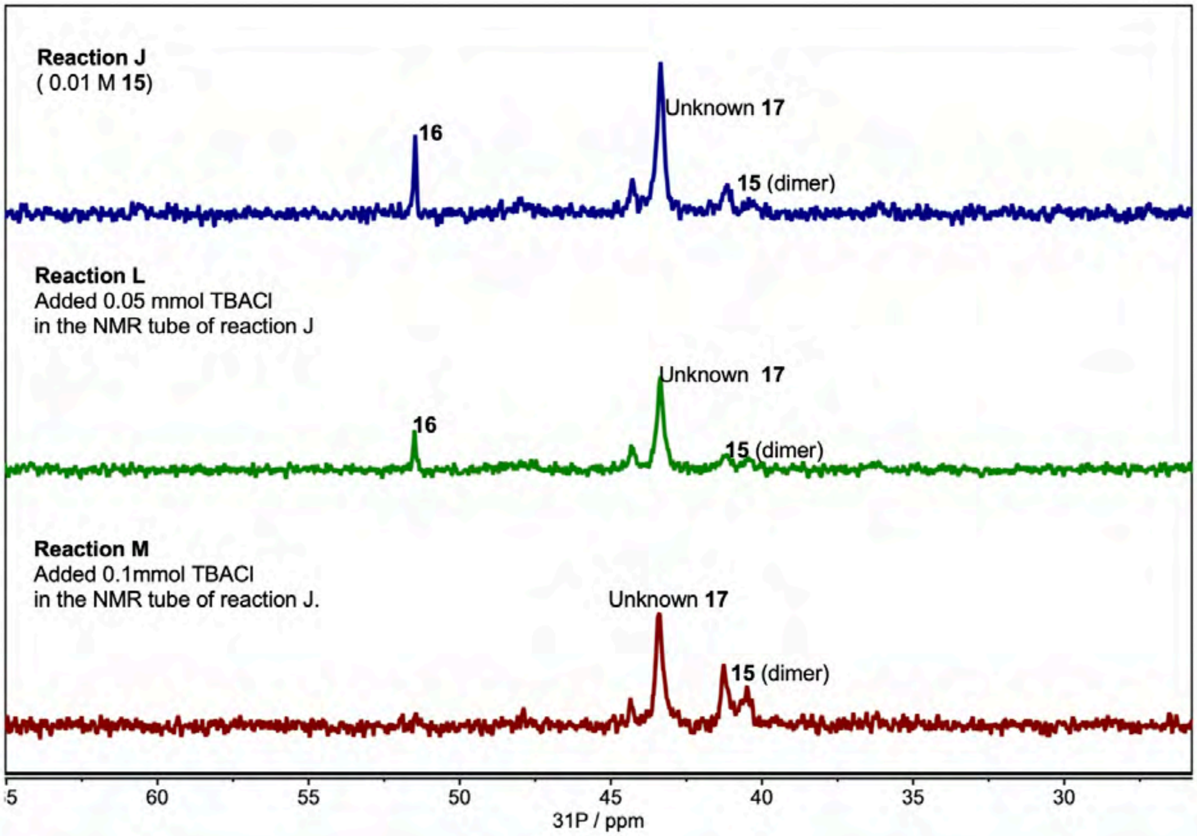

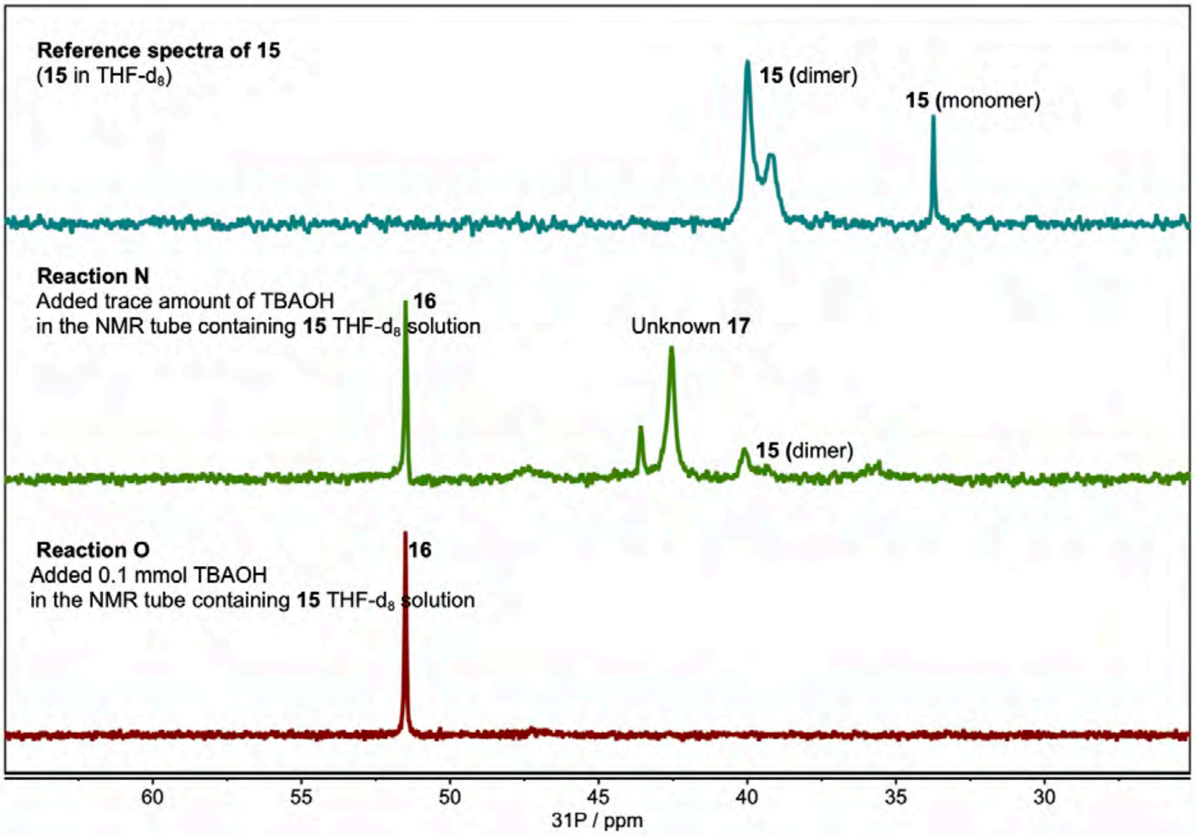

Supplementary Figure 44. Stacked $^{31}P\{^{1}H\}$ spectra of shifting the equilibrium between **15** and **16**

Investigation of palladium unknown 17

A).

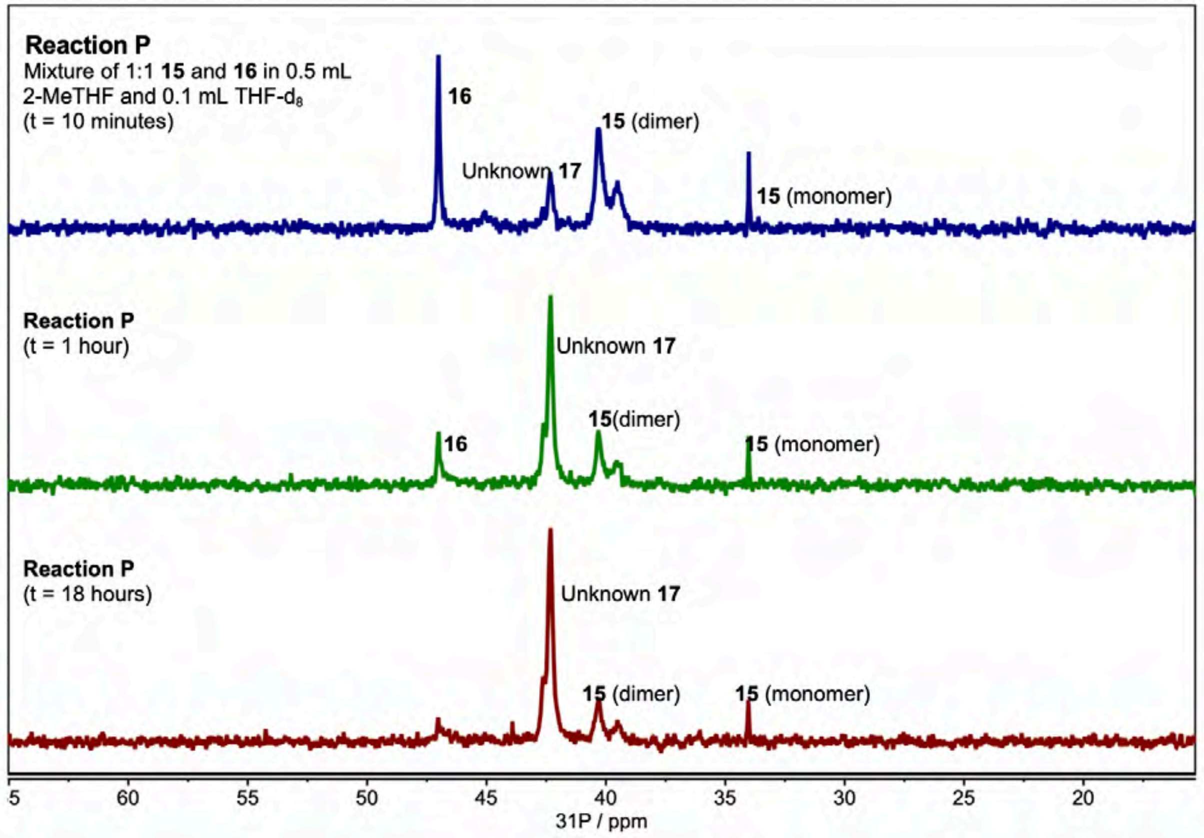

B).

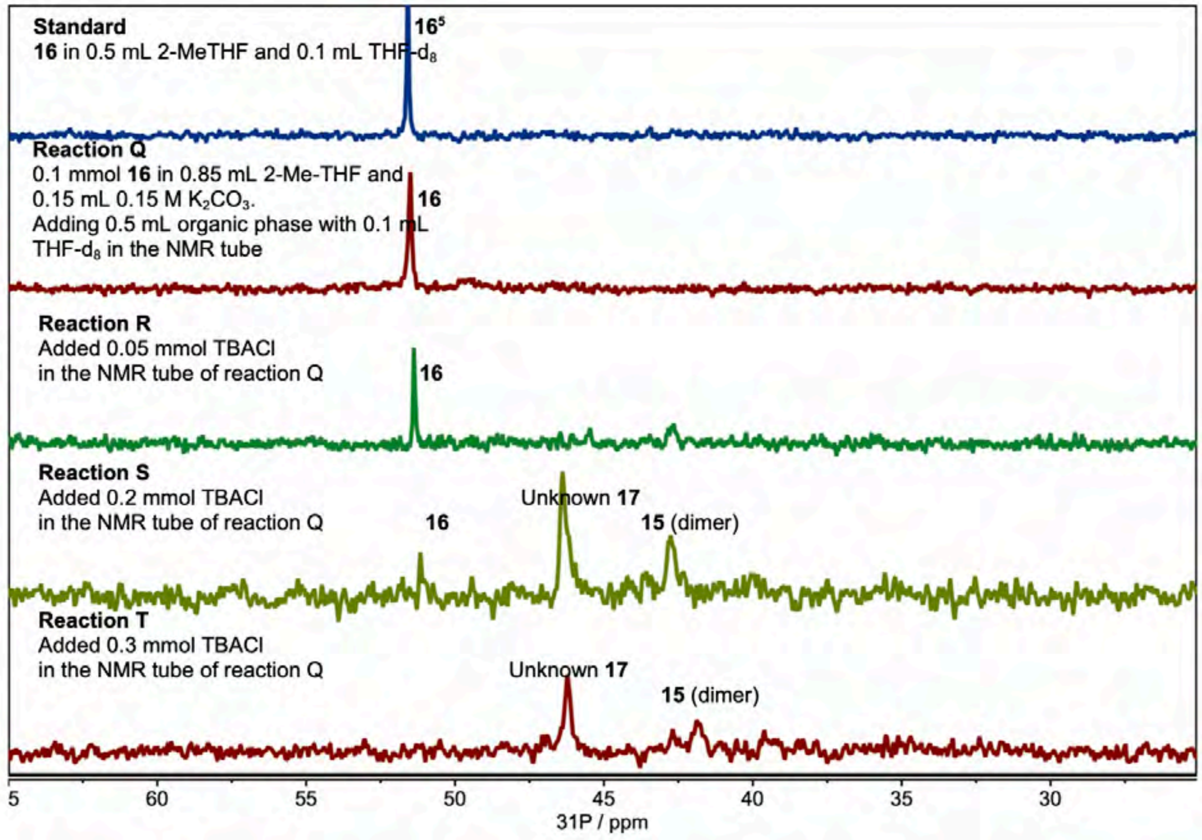

Supplementary Figure 45. Stacked $^{31}P\{^{1}H\}$ spectra of the investigation of the palladium **17** unknown

Conclusion from palladium equilibration experiment:
Quantitative ^{31}P NMR studies of the equilibration of the palladium oxidative addition complex (**15**) reveal that under standard conditions with no TBACl additive, the palladium has a slight preference for the Pd-Cl dimer over the Pd-OH (**16**). In contrast to the boron equilibration, the addition of TBACl results in a significant increase in **15** largely as the dimeric species while **16** becomes almost undetectable under such conditions. This suggests that although TBACl increases the concentration of hydroxide in the organic phase (confirmed by the boron equilibration studies), it also increases the concentration of the halide ion in the organic phase (further supported by the observation of competitive benzyl halide speciation from Supplementary Figure 15). Consequently, the greater strength of the Pd-Cl bond leads to its preferential formation. To further support the equilibration of **15** and **16**, sequential doses of TBACl were added to the NMR tube of trial 15 which then showed the complete disappearance of **16**. Moreover, sequential doses of TBAOH were added to a pure sample of **15** in 2-MeTHF where its complete transformation to **16** was observed. Notably, this experiment revealed significant formation of the unknown **17** prior to the complete transformation to **16** which suggest **17** is likely an equilibrium species. This is further supported through HPLC-MS analysis of NMR samples containing mixtures of **15**, **16**, and **17** showing a single peak whose m/z corresponds to the $[SPhos\text{-}Pd\text{-}Ar]^+$. We hypothesized that the unknown may largely arise from dimeric species with mixed OH/Cl bridging ligands. Thus, we synthesized the authentic Pd-OH species and made a 1:1 mixture of **15** and **16** in 2-MeTHF which resulted in the formation of **17** as expected. Although the ^{31}P NMR chemical shifts in this case were slightly shifted relative to the initial experiments, we confirmed this to be a result of the lack of base under such conditions through the addition of TBAOH to the authentic sample of **16**. Moreover, we were again able to show that sequential dosing of TBACl results in the formation of **17** and **15** in this sample as well. Notably, long equilibration times were observed during reaction P due to conducting the experiment in an NMR tube which lacked any stirring (Supplementary Figure 45A). The initial speciation experiments were confirmed to have equilibrated rapidly by comparing an initial sample (t=0 min) to a sample taken an hour later with minimal change observed (Supplementary Figure 42).

Overall conclusion from speciation experiments
Although both the Pd-OH and Pd-Cl oxidative addition complex are present in similar concentrations under standard conditions with no TBACl, the lack of any detectable 4-coordinate boron species in the organic phase suggest that the major pathway to product formation lies through pathway B. In contrast, upon the addition of TBACl, a significant increase in both the Pd-Cl and 4-coordinate boronate in the organic phase is observed, concomitant with a drastic increase in turnover rate. Thus, this suggest TBA additives enable a shift towards a lower energy boronate pathway.

Reaction Scope

General procedure B
To an oven-dried 3 mL glass vial with a magnetic stir bar, boronic esters (**2a-r**) (1.0 mmol) and $TBAOH \cdot 30H_2O$ (800 mg, 1.0 mmol) were added. The reaction vial was sealed with an open top screw cap fitted with a Teflon septum and attached to a Schlenk line via a needle. The system was evacuated for five minutes and backfilled with argon three times. Meanwhile, a base solution was made by adding K_2CO_3 (1.658 g, 12 mmol) to an oven-dried 6 mL vial. The vial with K_2CO_3 was evacuated via a needle for five minutes and backfilled with argon three times before 3.0 mL of water was added to the vial. A precatalyst solution was also made by adding XPhos Pd G2 (7.868 mg, 0.01 mmol) to an oven-dried HPLC vial. The vial with catalyst was evacuated via needle for five minutes and backfilled with argon three times before 1.0 mL of 2-MeTHF was added to the vial. Then, 0.9 ml of 2-MeTHF, benzyl bromide (**1a**) (128.3 mg, 0.75 mmol) were added through syringe to the reaction flask under argon. Finally, 0.1 mL of the catalyst solution, containing XPhos Pd G2 (0.7868 mg, 0.001 mmol), and 1.5 mL of K_2CO_3 base were injected via syringe to initiate the reaction. The reaction was stirred at 1200 rpm in a heat

block for 16 hours at 80 °C. After cooling, 3 mL of water was added to the mixture and then it was extracted 3x with 5 mL CH_2Cl_2. The organic layer was passed through a 3 x 1 cm silica plug, rinsed with 3 mL CH_2Cl_2 dried with anhydrous sodium sulfate, and concentrated by rotary evaporation. Reaction products were purified by silica gel column chromatograph or Buchi reverse phase chromatograph system.

General procedure C

To an oven-dried 3 mL glass vial with a magnetic stir bar, 4-methoxyphenyl boronic esters (**2**) (175 mg, 0.75 mmol) and TBAOH·30H_2O (800 mg, 1.0 mmol) were added. The reaction vial was sealed with an open top screw cap fitted with a Teflon septum and attached to a Schlenk line via a needle. The system was evacuated for five minutes and backfilled with argon three times. Meanwhile, a base solution was made by adding K_2CO_3 (1.658 g, 12 mmol) to an oven-dried 6 mL vial. The vial with K_2CO_3 was evacuated via a needle for five minutes and backfilled with argon three times before 3.0 mL of water was added to the vial. A precatalyst solution was also made by adding XPhos Pd G2 (7.868 mg, 0.01 mmol) to an oven-dried HPLC vial. The vial with catalyst was evacuated via needle for five minutes and backfilled with argon three times before 1.0 mL of 2-MeTHF was added to the vial. Then, 0.9 ml of 2-MeTHF, electrophiles (**1a-v**) (1.0 mmol) were added through syringe to the reaction flask under argon. Finally, 0.1 mL of the catalyst solution, containing XPhos Pd G2 (0.7868 mg, 0.001 mmol), and 1.5 mL of K_2CO_3 base were injected via syringe to initiate the reaction. Under an argon funnel, the pierced cap was replaced with a Teflon lined screw cap. The cap was sealed with electrical tape and then the reaction was stirred at 1200 rpm in a heat block for 16 hours at 80 °C. After cooling, 3 mL of water was added to the mixture and then it was extracted 3x with 5 mL CH_2Cl_2. The organic layer was passed through a 3 x 1 cm silica plug, rinsed with 3 mL CH_2Cl_2 dried with anhydrous sodium sulfate, and concentrated by rotary evaporation. Reaction products were purified by silica gel column chromatograph or an automated BUCHI Pure C-810 Flash Chromatography with Redisep Rf Gold C18 reversed phase column.

Characterization data

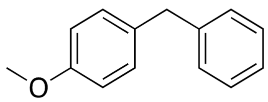

1-benzyl-4-methoxybenzene (**3aa**)

Following the Cross-Coupling condition B. The crude residue was purified *via* flash chromatography to afford a clear oil (138 mg, 0.69 mmol, 93% yield).

TLC Rf= 0.35 (5% EtOAc/petroleum ether)

^{1}H NMR (300 MHz, $CDCl_3$) δ_H 7.40 – 7.30 (m, 2H), 7.29 – 7.20 (m, 3H), 7.17 (d, J = 8.8 Hz, 2H), 6.90 (d, J = 7.9 Hz, 2H), 3.99 (s, 2H), 3.83 (s, 3H);

^{13}C{^{1}H} NMR (75 MHz, $CDCl_3$) δ_C 158.04, 141.65, 133.31, 129.93, 128.88, 128.49, 126.04, 113.94, 55.29, 41.10;

HRMS ESI+: (*m/z* calc. for $C_{14}H_{14}O$ $[M]^+$ = 198.1045); found = 198.1047

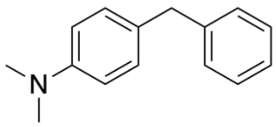

4-benzyl-*N*,*N*-dimethylaniline (**3ba**)

Following the Cross-Coupling condition B. The crude residue was purified *via* flash chromatography to afford a pale yellow oil (194 mg, 0.69 mmol, 92% yield).

TLC Rf= 0.5 (6% EtOAc/petroleum ether)

^{1}H NMR (400 MHz, $CDCl_3$) δ_H 7.29 (m, *J* = 7.5 Hz, 2H), 7.21 (m, *J* = 7.5 Hz, 3H), 7.09 (d, *J* = 8.2 Hz, 2H), 6.74 (d, *J* = 8.1 Hz, 2H), 3.92 (s, 2H), 2.94 (s, *J* = 1.0 Hz, 6H);
$^{13}C\{^1H\}$ NMR (101 MHz, $CDCl_3$) δ_C 149.16, 142.10, 129.59, 129.41, 128.85, 128.38, 125.82, 113.07, 40.97, 40.91;
HRMS ESI+: (*m/z* calc. for $C_{15}H_{17}N$ $[M]^+$ = 211.1366); found = 211.1361

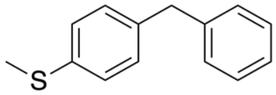
(4-benzylphenyl)(methyl)sulfane (**3ca**)
Following the Cross-Coupling condition B. The crude residue was purified *via* flash chromatography to afford a clear oil (142 mg, 0.667 mmol, 89% yield).
TLC Rf= 0.40 (33% EtOAc/petroleum ether)
^{1}H NMR (300 MHz, $CDCl_3$) δ_H 7.23 – 7.16 (m, 2H), 7.15 – 7.06 (m, 5H), 7.03 (d, *J* = 8.3 Hz, 2H), 3.86 (s, 2H), 2.37 (s, 3H);
$^{13}C\{^1H\}$ NMR (75 MHz, $CDCl_3$) δ_C 141.00, 138.23, 135.76, 129.47, 128.89, 128.51, 127.15, 126.15, 41.39, 16.24;
HRMS ESI+: (*m/z* calc. for $C_{14}H_{14}S$ $[M]^+$ = 214.0816); found = 214.0813

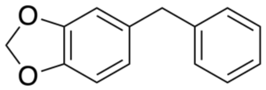
5-benzylbenzo[*d*][1,3]dioxole (**3da**)
Following the Cross-Coupling condition B. The crude residue was purified *via* flash chromatography to afford a clear oil (144 mg, 0.682 mmol, 91% yield).
TLC Rf= 0.40 (5% EtOAc/petroleum ether)
^{1}H NMR (400 MHz, $CDCl_3$) δ_H 7.47 – 7.16 (m, 5H), 6.94 – 6.61 (m, 3H), 5.95 (s, 2H), 3.95 (s, 2H);
$^{13}C\{^1H\}$ NMR (101 MHz, $CDCl_3$) δ_C 147.91, 146.07, 141.47, 135.19, 128.99, 128.68, 126.31, 121.92, 109.61, 108.35, 101.01, 41.81;
HRMS ESI+: (*m/z* calc. for $C_{14}H_{12}O_2$ $[M]^+$ = 212.0837); found = 212.0837

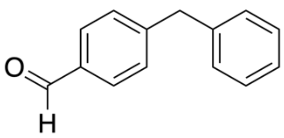
4-benzylbenzaldehyde (**3ea**)
Following the Cross-Coupling condition B. The crude residue was purified *via* flash chromatography to afford an orange oil (132 mg, 0.675 mmol, 90% yield).
TLC Rf= 0.40 (2% EtOAc/petroleum ether)
^{1}H NMR (300 MHz, $CDCl_3$) δ_H 9.86 (s, 1H), 7.69 (d, *J* = 8.2 Hz, 2H), 7.26 – 7.11 (m, 5H), 7.08 (d, *J* = 6.6 Hz, 2H), 3.95 (s, 2H);
$^{13}C\{^1H\}$ NMR (75 MHz, $CDCl_3$) δ_C 192.03, 148.47, 139.79, 134.68, 130.08, 129.60, 129.01, 128.73, 126.56, 42.11;
HRMS ESI+: (*m/z* calc. for $C_{14}H_{12}O$ $[M]^+$ = 196.0888); found = 196.0882

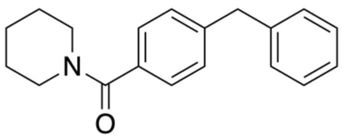
(4-benzylphenyl)(piperidin-1-yl)methanone (**3fa**)

Following the Cross-Coupling condition B with TBAB (322 mg, 1.0 mmol) as additive. The crude residue was purified *via* flash chromatography to afford a clear oil (194 mg, 0.697 mmol, 93% yield).
TLC Rf= 0.20 (20% EtOAc/petroleum ether)
^{1}H NMR (300 MHz, $CDCl_3$) δ_H 7.43 – 7.27 (m, 4H), 7.27 – 7.15 (m, 5H), 4.01 (s, 2H), 3.54 (d, *J* = 64.1 Hz, 4H), 1.79 – 1.43 (m, 6H);
^{13}C{^{1}H} NMR (75 MHz, $CDCl_3$) δ_C 169.33, 143.04, 141.28, 134.69, 129.25, 129.04, 128.97, 127.39, 126.56, 45.71, 41.34, 26.1, 24.54;
HRMS ESI+: (*m/z* calc. for $C_{19}H_{21}NO$ $[M]^+$ = 279.1623); found = 279.1627

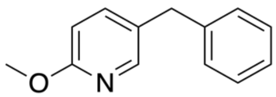

5-benzyl-2-methoxypyridine (**3ga**)
Following the Cross-Coupling condition B. The crude residue was purified *via* BUCHI C18 reversed phase column to afford a clear oil (107 mg, 0.54 mmol, 72% yield).
Using a gradient method from 5-55% water/acetonitrile.
^{1}H NMR (300 MHz, $CDCl_3$) δ_H 7.98 (d, *J* = 1.6 Hz, 1H), 7.30 (dd, *J* = 8.5, 2.5 Hz, 1H), 7.26 – 7.18 (m, 2H), 7.18 – 7.06 (m, 3H), 6.61 (dd, *J* = 8.5, 0.8 Hz, 1H), 3.86 (s, 3H), 3.83 (s, 2H);
^{13}C{^{1}H} NMR (75 MHz, $CDCl_3$) δ_C 162.60, 146.06, 140.32, 139.21, 128.95, 128.50, 128.38, 126.09, 110.53, 53.15, 37.88;
HRMS ESI+: (*m/z* calc. for $C_{13}H_{13}NO$ $[M]^+$ = 199.0997); found = 199.1000

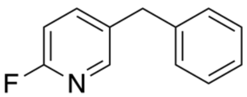

5-benzyl-2-fluoropyridine (**3ha**)
Following the Cross-Coupling condition B. The crude residue was purified *via* BUCHI C18 reversed phase column to afford a pale yellow oil (149 mg, 0.6 mmol, 80% yield).
Using a gradient method from 5-55% water/acetonitrile.
^{1}H NMR (300 MHz, $CDCl_3$) δ_H 8.11 (d, *J* = 2.5 Hz, 1H), 7.58 (td, *J* = 8.2, 2.6 Hz, 1H), 7.40 – 7.13 (m, 5H), 6.85 (dd, *J* = 8.4, 3.0 Hz, 1H), 3.98 (s, 2H);
^{13}C{^{1}H} NMR (75 MHz, $CDCl_3$) δ_C 162.44 (d, J = 237.8 Hz), 147.28 (d, J = 14.3 Hz), 141.57 (d, J = 8.3 Hz), 139.6 (d, J = 1.5 Hz), 134.2 (d, J = 4.5 Hz), 128.79, 128.78, 126.66, 109.29 (d, J = 37.7 Hz), 38.02 (d, J = 1.5 Hz);
HRMS ESI+: (*m/z* calc. for $C_{12}H_{10}FN$ $[M]^+$ = 187.0797); found = 187.0801

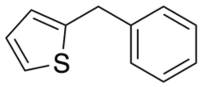

2-benzylthiophene (**3ia**)
Following the Cross-Coupling condition B. The crude residue was purified *via* BUCHI C18 reversed phase column to afford a pale yellow oil (113 mg, 0.652 mmol, 87% yield).
Using a gradient method from 5-55% water/acetonitrile.
^{1}H NMR (300 MHz, $CDCl_3$) δ_H 7.23 – 7.10 (m, 5H), 7.05 (dd, *J* = 5.2, 1.2 Hz, 1H), 6.83 (dd, *J* = 5.1, 3.4 Hz, 1H), 6.71 (dd, *J* = 3.5, 1.1 Hz, 1H), 4.07 (s, 2H);
^{13}C{^{1}H} NMR (75 MHz, $CDCl_3$) δ_C 141.54, 140.66, 128.82, 128.54, 128.51, 126.22, 125.66, 121.30, 36.59;
HRMS ESI+: (*m/z* calc. for $C_{11}H_{10}S$ $[M]^+$ = 174.0503); found = 174.0500

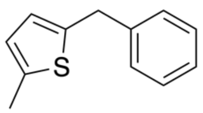

2-benzyl-5-methylthiophene (**3ja**)
Following the Cross-Coupling condition B. The crude residue was purified *via* BUCHI C18 reversed phase column to afford a pale yellow clear oil (119 mg, 0.637 mmol, 85% yield).
Using a gradient method from 5-55% water/acetonitrile.
^{1}H NMR (300 MHz, $CDCl_3$) δ_H 7.38 – 7.24 (m, 5H), 6.67 – 6.54 (m, 2H), 4.12 (s, 2H), 2.46 (d, *J* = 1.0 Hz, 3H);
^{13}C{^{1}H} NMR (75 MHz, $CDCl_3$) δ_C 141.77, 140.61, 138.42, 128.56, 128.52, 126.41, 124.86, 124.73, 36.29, 15.32;
HRMS ESI+: (*m/z* calc. for $C_{12}H_{12}S$ $[M]^+$ = 188.0660); found = 188.0658

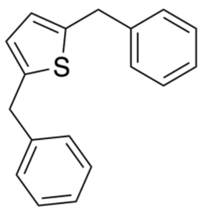

2,5-dibenzylthiophene (**3ka**)
Following the Cross-Coupling condition B. The crude residue was purified *via* BUCHI C18 reversed phase column to afford a clear oil (214 mg, 0.607 mmol, 81% yield).
Using a gradient method from 5-55% water/acetonitrile.
^{1}H NMR (300 MHz, $CDCl_3$) δ_H 7.24 – 7.08 (m, 10H), 6.50 (s, 2H), 3.97 (s, 4H);
^{13}C{^{1}H} NMR (75 MHz, $CDCl_3$) δ_C 142.74, 140.40, 128.63, 128.53, 126.45, 124.75, 36.36;
HRMS ESI+: (*m/z* calc. for $C_{18}H_{16}S$ $[M]^+$ = 264.0972); found = 264.0939

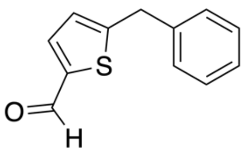

5-benzylthiophene-2-carbaldehyde (**3la**)
Following the Cross-Coupling condition B with 1 mol% catalyst loading. The crude residue was purified *via* BUCHI C18 reversed phase column to afford a pale yellow oil (127 mg, 0.472 mmol, 63% yield).
Using a gradient method from 5-55% water/acetonitrile.
^{1}H NMR (300 MHz, $CDCl_3$) δ_H 9.84 (s, 1H), 7.63 (d, *J* = 3.8 Hz, 1H), 7.39 – 7.26 (m, 5H), 6.93 (d, *J* = 3.8 Hz, 1H), 4.22 (s, 2H);
^{13}C{^{1}H} NMR (75 MHz, $CDCl_3$) δ_C 182.78, 155.88, 142.42, 138.67, 136.97, 128.84, 128.69, 127.08, 126.65, 36.91;
HRMS ESI+: (*m/z* calc. for $C_{12}H_{10}OS$ $[M]^+$ = 202.0452); found = 202.0418

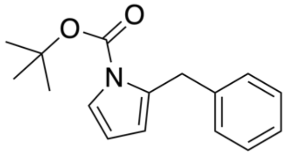

tert-butyl 2-benzyl-1*H*-pyrrole-1-carboxylate (**3ma**)
Following the Cross-Coupling condition B. The crude residue was purified *via* flash chromatography to afford a clear oil (218 mg, 0.637 mmol, 85% yield).
TLC Rf= 0.40 (petroleum ether)

^{1}H NMR (400 MHz, THF) δ_H 7.21 – 7.13 (m, 3H), 7.12 – 7.03 (m, 3H), 5.98 (t, *J* = 3.3 Hz, 1H), 5.66 (dt, *J* = 2.9, 1.5 Hz, 1H), 4.11 (s, 2H), 1.39 (s, 9H);
^{13}C{^{1}H} NMR (101 MHz, THF) δ_C 149.76, 140.07, 134.51, 129.01, 128.48, 126.29, 121.61, 113.40, 110.23, 83.73, 35.30, 28.14;
HRMS ESI+: (*m/z* calc. for $C_{16}H_{19}NO_2$ $[M]^+$ = 257.1416); found = 257.1424

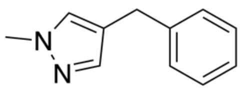

4-benzyl-1-methyl-1*H*-pyrazole (**3na**)
Following the Cross-Coupling condition B with 1 mol% catalyst loading using TBAB (322 mg, 1.0 mmol) as additive. The crude residue was purified *via* flash chromatography to afford a clear oil (112 mg, 0.487 mmol, 65% yield).
TLC Rf= 0.30 (20% EtOAc/petroleum ether)
^{1}H NMR (300 MHz, $CDCl_3$) δ_H 7.26 (s, 1H), 7.24 – 7.15 (m, 2H), 7.15 – 7.04 (m, 3H), 7.01 (s, 1H), 3.75 (s, 3H), 3.71 (s, 2H);
^{13}C{^{1}H} NMR (75 MHz, $CDCl_3$) δ_C 140.87, 138.43, 129.32, 128.54, 128.47, 126.20, 121.13, 38.64, 30.53;
HRMS ESI+: (*m/z* calc. for $C_{11}H_{12}N_2$ $[M]^+$ = 172.1000); found = 172.1007

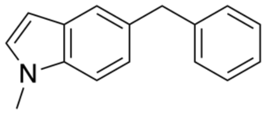

5-benzyl-1-methyl-1*H*-indole (**3oa**)
Following the Cross-Coupling condition B. The crude residue was purified *via* flash chromatography to afford a yellow oil (166 mg, 0.69 mmol, 92% yield).
TLC Rf= 0.40 (5% EtOAc/petroleum ether)
^{1}H NMR (400 MHz, $CDCl_3$) δ_H 7.50 (dd, *J* = 1.7, 0.8 Hz, 1H), 7.35 – 7.26 (m, 5H), 7.26 – 7.20 (m, 1H), 7.12 (dd, *J* = 8.4, 1.7 Hz, 1H), 7.07 (d, *J* = 2.7 Hz, 1H), 6.47 (d, *J* = 3.1 Hz, 1H), 4.15 (s, 2H), 3.81 (s, 3H);
^{13}C{^{1}H} NMR (101 MHz, $CDCl_3$) δ_C 142.46, 135.49, 132.02, 129.02, 128.94, 128.72, 128.37, 125.81, 123.05, 120.84, 109.20, 100.67, 42.06, 32.88;
HRMS ESI+: (*m/z* calc. for $C_{16}H_{15}N$ $[M]^+$ = 221.1204); found = 221.1210

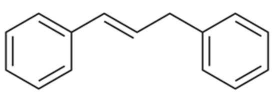

(*E*)-prop-1-ene-1,3-diyldibenzene (**3pa**)
Following the Cross-Coupling condition B. The crude residue was purified *via* flash chromatography to afford a clear oil (178 mg, 0.69 mmol, 92% yield).
TLC Rf= 0.70 (petroleum ether)
^{1}H NMR (300 MHz, $CDCl_3$) δ_H 7.31 – 7.04 (m, 10H), 6.42 – 6.20 (m, 2H), 3.46 (d, *J* = 6.4 Hz, 2H);
^{13}C{^{1}H} NMR (75 MHz, $CDCl_3$) δ_C 140.23, 137.54, 131.14, 129.29, 128.74, 128.56, 127.17, 126.25, 126.19, 39.42, 29.79;
HRMS ESI+: (*m/z* calc. for $C_{15}H_{14}$ $[M]^+$ = 194.1096); found = 194.1090

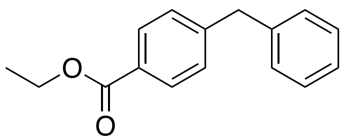

Ethyl 4-benzylbenzoate (**3qa**)

Following the Cross-Coupling condition B. The crude residue was purified *via* flash chromatography to afford a clear oil (166 mg, 0.69 mmol, 92% yield).
TLC Rf= 0.3 (10% EtOAc/petroleum ether)
^{1}H NMR (300 MHz, $CDCl_3$) δ_H 7.88 (d, *J* = 8.0 Hz, 2H), 7.26 – 7.01 (m, 7H), 4.27 (q, *J* = 7.1 Hz, 2H), 3.93 (s, 2H), 1.28 (t, *J* = 7.1 Hz, 3H);
^{13}C{^{1}H} NMR (75 MHz, $CDCl_3$) δ_C 166.59, 146.41, 140.20, 129.81, 128.95, 128.93, 128.62, 128.48, 126.38, 60.83, 42.21, 14.37;
HRMS ESI+: (*m/z* calc. for $C_{16}H_{16}O_2$ $[M]^+$ = 240.1105); found = 240.1159

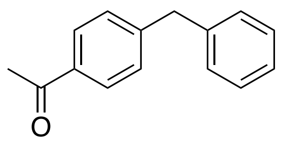

1-(4-benzylphenyl)ethan-1-one (**3ra**)
Following the Cross-Coupling condition B. The crude residue was purified *via* flash chromatography to afford a clear oil (149 mg, 0.71 mmol, 95% yield).
TLC Rf= 0.30 (10% EtOAc/petroleum ether)
^{1}H NMR (300 MHz, $CDCl_3$) δ_H 7.83 – 7.76 (m, 2H), 7.26 – 7.00 (m, 7H), 3.94 (s, 2H), 2.48 (s, 3H);
^{13}C{^{1}H} NMR (75 MHz, $CDCl_3$) δ_C 197.82, 146.84, 140.06, 135.27, 129.13, 128.96, 128.66, 126.44, 41.92, 26.58;
HRMS ESI+: (*m/z* calc. for $C_{15}H_{14}O$ $[M]^+$ = 210.1044); found = 210.1053

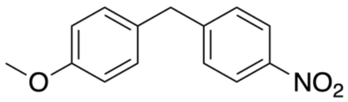

1-methoxy-4-(4-nitrobenzyl)benzene (**3ab**)
Following the Cross-Coupling condition C. The crude residue was purified *via* flash chromatography to afford a pale yellow oil (156 mg, 0.645 mmol, 86% yield).
TLC Rf= 0.20 (5% EtOAc/petroleum ether)
^{1}H NMR (300 MHz, $CDCl_3$) δ_H 8.13 (d, *J* = 8.7 Hz, 2H), 7.32 (d, *J* = 8.7 Hz, 2H), 7.09 (d, *J* = 8.6 Hz, 2H), 6.86 (d, *J* = 8.6 Hz, 2H), 4.02 (s, 2H), 3.80 (s, 3H);
^{13}C{^{1}H} NMR (75 MHz, $CDCl_3$) δ_C 158.53, 149.51, 146.54, 131.36, 130.06, 129.62, 123.82, 114.31, 55.38, 40.95;
HRMS ESI+: (*m/z* calc. for $C_{14}H_{13}NO_3$ $[M]^+$ = 243.0895); found = 243.0903

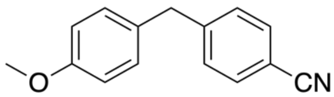

4-(4-methoxybenzyl)benzonitrile (**3ac**)
Following the Cross-Coupling condition C. The crude residue was purified *via* flash chromatography to afford a pale yellow oil (148 mg, 0.667 mmol, 89% yield).
TLC Rf= 0.20 (5% EtOAc/petroleum ether)
^{1}H NMR (300 MHz, $CDCl_3$) δ_H 7.47 (d, *J* = 8.2 Hz, 2H), 7.18 (d, *J* = 8.3 Hz, 2H), 6.99 (d, *J* = 8.6 Hz, 2H), 6.77 (d, *J* = 8.6 Hz, 2H), 3.89 (s, 2H), 3.70 (s, 3H);
^{13}C{^{1}H} NMR (75 MHz, $CDCl_3$) δ_C 158.38, 147.27, 132.29, 131.41, 129.97, 129.53, 119.05, 114.18, 109.92, 55.29, 41.10;
HRMS ESI+: (*m/z* calc. for $C_{15}H_{13}NO$ $[M]^+$ = 223.0996); found = 223.0997

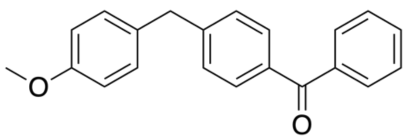

(4-(4-methoxybenzyl)phenyl)(phenyl)methanone (**3ad**)
Following the Cross-Coupling condition C. The crude residue was purified *via* flash chromatography to afford a white solid (208 mg, 0.69 mmol, 92% yield).
Using a gradient method from 5-55% water/acetonitrile.
^{1}H NMR (300 MHz, $CDCl_3$) δ_H 7.83 – 7.71 (m, 4H), 7.62 – 7.53 (m, 1H), 7.52 – 7.39 (m, 2H), 7.30 (d, *J* = 8.2 Hz, 2H), 7.14 (d, *J* = 8.6 Hz, 2H), 6.87 (d, *J* = 8.6 Hz, 2H), 4.01 (s, 2H), 3.80 (s, 3H);
^{13}C{^{1}H} NMR (75 MHz, $CDCl_3$) δ_C 196.46, 158.23, 146.72, 137.84, 135.44, 132.27, 132.21, 130.49, 129.99, 128.73, 128.25, 114.07, 55.28, 41.08;
HRMS ESI+: (*m/z* calc. for $C_{21}H_{18}O_2$ $[M]^+$ = 302.1307); found = 302.1312

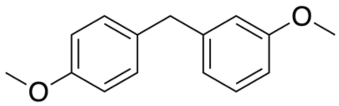

1-methoxy-3-(4-methoxybenzyl)benzene (**3ae**)
Following the Cross-Coupling condition C. The crude residue was purified *via* BUCHI C18 reversed phase column to afford a clear oil (155 mg, 0.682 mmol, 91% yield).
Using a gradient method from 5-55% water/acetonitrile.
^{1}H NMR (300 MHz, $CDCl_3$) δ_H 7.31 – 7.20 (m, 1H), 7.15 (d, *J* = 8.6 Hz, 2H), 6.87 (d, *J* = 8.6 Hz, 2H), 6.84 – 6.67 (m, 3H), 3.94 (s, 2H), 3.81 (d, *J* = 3.8 Hz, 6H);
^{13}C{^{1}H} NMR (75 MHz, $CDCl_3$) δ_C 159.73, 158.01, 143.23, 133.07, 129.88, 129.40, 121.28, 114.68, 113.90, 111.24, 55.27, 55.14, 41.07;
HRMS ESI+: (*m/z* calc. for $C_{15}H_{16}O_2$ $[M]^+$ = 228.1150); found = 228.1147

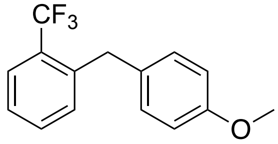

1-(4-methoxybenzyl)-2-(trifluoromethyl)benzene (**3af**)
Following the Cross-Coupling condition C. The crude residue was purified *via* BUCHI C18 reversed phase column to afford a clear oil (188 mg, 0.705 mmol, 94% yield).
Using a gradient method from 5-65% water/acetonitrile.
^{1}H NMR (300 MHz, MeOD) δ_H 7.62 – 7.54 (m, 1H), 7.39 (td, *J* = 7.8, 1.4 Hz, 1H), 7.31 – 7.21 (m, 1H), 7.19 – 7.09 (m, 1H), 7.00 – 6.90 (m, 2H), 6.80 – 6.72 (m, 2H), 4.01 (s, 2H), 3.67 (s, 3H);
^{13}C{^{1}H} NMR (75 MHz, MeOD) δ_C 159.74, 141.38, 133.22, 133.14, 133.04, 131.01, 129.50 (q, J = 58.5 Hz), 127.47, 126.76 (q, J = 11.7 Hz), 124.36 (t, J = 271.58 Hz), 114.97, 55.67, 37.85;

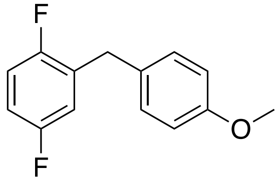

1,4-difluoro-2-(4-methoxybenzyl)benzene (**3ag**)
Following the Cross-Coupling condition C. The crude residue was purified *via* BUCHI C18 reversed phase column to afford a clear oil (162 mg, 0.69 mmol, 92% yield).
Using a gradient method from 5-65% water/acetonitrile.

^{1}H NMR (300 MHz, $CDCl_3$) δ_H 7.17 – 7.10 (m, 2H), 6.98 (td, *J* = 8.9, 4.6 Hz, 1H), 6.83 (ddt, *J* = 18.2, 8.9, 2.9 Hz, 4H), 3.91 (d, *J* = 1.5 Hz, 2H), 3.80 (s, 3H).
^{13}C{^{1}H} NMR (75 MHz, $CDCl_3$) δ_C 159.3 (d, J = 137.25 Hz), 158.3, 156.15 (d, J = 138.50 Hz), 131.01, 130.39 (q, J = 18.56 Hz), 129.86, 117.11 (q, J = 23.7 Hz), 116.15 (q, J = 25.13 Hz), 114.09, 114.07 (J =23.98 Hz), 55.25, 33.92;

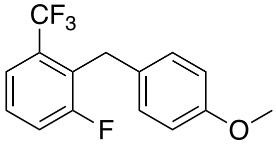

1-fluoro-2-(4-methoxybenzyl)-3-(trifluoromethyl)benzene (**3ah**)
Following the Cross-Coupling condition C. The crude residue was purified *via* BUCHI C18 reversed phase column to afford a clear oil (192 mg, 0.675 mmol, 90% yield).
Using a gradient method from 5-65% water/acetonitrile.
^{1}H NMR (300 MHz, MeOD) δ_H 7.47 (dt, *J* = 7.9, 1.0 Hz, 1H), 7.43 – 7.24 (m, 2H), 6.95 – 6.86 (m, 2H), 6.74 – 6.68 (m, 2H), 4.03 (d, *J* = 2.2 Hz, 2H), 3.65 (s, 3H);
^{13}C{^{1}H} NMR (75 MHz, MeOD) δ_C 163.42 (d, J = 3.26 Hz), 159.59, 132.24, 129.92, 129.80, 128.31 (d, J = 17.25 Hz), 122.21, 123.59, 122.99 (t, J = 5.25 Hz), 120.55 (d, J = 32.25 Hz), 114.77, 55.64, 31.22;

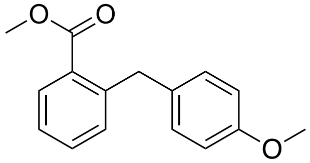

Methyl 2-(4-methoxybenzyl)benzoate (**3ai**)
Following the Cross-Coupling condition C with TBAB (322 mg, 1.0 mmol) as additive. The crude residue was purified *via* BUCHI C18 reversed phase column to afford a white solid (161 mg, 0.63 mmol, 84% yield).
Using a gradient method from 5-55% water/acetonitrile.
^{1}H NMR (300 MHz, $CDCl_3$) δ_H 7.89 (dd, *J* = 7.8, 1.5 Hz, 1H), 7.42 (td, *J* = 7.5, 1.5 Hz, 1H), 7.31 – 7.18 (m, 2H), 7.10 – 7.05 (m, 2H), 6.84 – 6.79 (m, 2H), 4.32 (s, 2H), 3.84 (s, 3H), 3.78 (s, 3H);
^{13}C{^{1}H} NMR (75 MHz, $CDCl_3$) δ_C 168.16, 157.85, 142.67, 133.01, 131.94, 131.38, 130.62, 129.91, 129.87, 126.14, 113.76, 55.22, 51.94, 38.70;

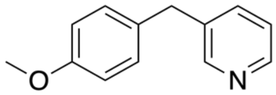

3-(4-methoxybenzyl)pyridine (**3aj**)
Following the Cross-Coupling condition C. The crude residue was purified *via* BUCHI C18 reversed phase column to afford a pale yellow oil (129 mg, 0.652 mmol, 87% yield).
Using a gradient method from 0-30% water/acetonitrile.
^{1}H NMR (300 MHz, $CDCl_3$) δ_H 8.82 – 8.62 (m, 2H), 8.18 (d, *J* = 7.9 Hz, 1H), 7.83 (dd, *J* = 8.0, 5.5 Hz, 1H), 7.12 (d, *J* = 8.5 Hz, 2H), 6.91 (d, *J* = 8.5 Hz, 2H), 4.14 (s, 2H), 3.82 (s, 3H);
^{13}C{^{1}H} NMR (75 MHz, $CDCl_3$) δ_C 159.09, 145.29, 142.48, 141.52, 139.63, 130.08, 128.40, 126.55, 114.79, 55.32, 37.81;
HRMS ESI+: (*m/z* calc. for $C_{13}H_{13}NO$ $[M]^+$ = 199.0997); found = 199.1001

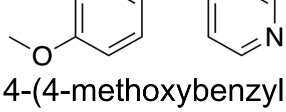

4-(4-methoxybenzyl)pyridine (**3ak**)

Following the Cross-Coupling condition C. The crude residue was purified *via* BUCHI C18 reversed phase column to afford a pale yellow oil (129 mg, 0.652 mmol, 82% yield).
Using a gradient method from 0-30% water/acetonitrile.
^{1}**H NMR** (300 MHz, $CDCl_3$) δ_H 8.47 (d, *J* = 5.0 Hz, 2H), 7.18 – 6.98 (m, 4H), 6.84 (d, *J* = 8.1 Hz, 2H), 3.89 (s, 2H), 3.77 (s, 3H);
^{13}**C{^{1}H} NMR** (75 MHz, $CDCl_3$) δ_C 158.38, 150.49, 149.81, 130.92, 130.03, 124.08, 114.12, 55.25, 40.34;
HRMS ESI+: (*m/z* calc. for $C_{13}H_{13}NO$ $[M]^+$ = 199.0997); found = 199.0977

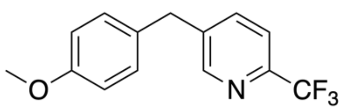

5-(4-methoxybenzyl)-2-(trifluoromethyl)pyridine (**3al**)
Following the Cross-Coupling condition C. The crude residue was purified *via* BUCHI C18 reversed phase column to afford a clear oil (170 mg, 0.637 mmol, 85% yield).
Using a gradient method from 5-55% water/acetonitrile.
^{1}**H NMR** (300 MHz, $CDCl_3$) δ_H 8.64 – 8.53 (m, 1H), 7.67 – 7.52 (m, 2H), 7.09 (d, *J* = 8.6 Hz, 2H), 6.86 (d, *J* = 8.6 Hz, 2H), 3.99 (s, 2H), 3.79 (s, 3H);
^{13}**C{^{1}H} NMR** (75 MHz, CDCl3) δ_C 158.56, 150.25, 146.05 (q, J=34.4 Hz), 140.52, 137.37, 130.76, 129.90, 123.58, 120.25 (q, J=2.7 Hz), 119.96, 55.20, 37.93;
HRMS ESI+: (*m/z* calc. for $C_{14}H_{12}F_3NO$ $[M]^+$ = 267.0871); found = 267.0875

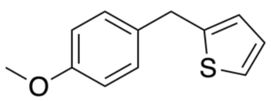

2-(4-methoxybenzyl)thiophene (**3am**)
Following the Cross-Coupling condition C. The crude residue was purified *via* flash chromatography to afford a pale yellow oil (131 mg, 0.645 mmol, 86% yield).
Using a gradient method from 5-55% water/acetonitrile.
^{1}**H NMR** (300 MHz, $CDCl_3$) δ_H 7.34 – 7.25 (m, 1H), 7.17 (d, *J* = 8.8 Hz, 2H), 6.95 (d, *J* = 4.4 Hz, 2H), 6.89 (d, *J* = 8.6 Hz, 2H), 3.97 (s, 2H), 3.83 (s, 3H);
^{13}**C{^{1}H} NMR** (75 MHz, $CDCl_3$) δ_C 158.05, 142.07, 132.76, 129.70, 128.42, 125.58, 121.03, 113.91, 55.28, 35.67;
HRMS ESI+: (*m/z* calc. for $C_{12}H_{12}OS$ $[M]^+$ = 204.0611); found = 204.0609

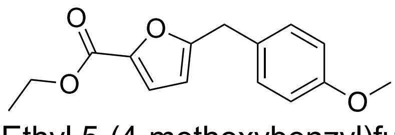

Ethyl 5-(4-methoxybenzyl)furan-2-carboxylate (**3an**)
Following the Cross-Coupling condition C with TBAB (322 mg, 1.0 mmol) as additive. The crude residue was purified *via* BUCHI C18 reversed phase column to afford a clear oil (148 mg, 0.57 mmol, 76% yield).
Using a gradient method from 5-55% water/acetonitrile.
^{1}**H NMR** (300 MHz, MeOD) δ_H 7.08 – 6.96 (m, 3H), 6.77 – 6.67 (m, 2H), 6.03 (dt, *J* = 3.4, 0.8 Hz, 1H), 4.17 (q, *J* = 7.1 Hz, 2H), 3.82 (s, 2H), 3.63 (s, 3H), 1.20 (t, *J* = 7.1 Hz, 3H).
^{13}**C{^{1}H} NMR** (75 MHz, $CDCl_3$) δ_C 161.89, 160.47, 160.09, 144.75, 130.86, 130.29, 120.38, 115.08, 109.68, 61.89, 55.68, 34.52, 14.63;

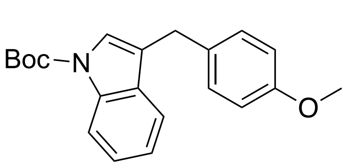

tert-butyl 3-(4-methoxybenzyl)-1*H*-indole-1-carboxylate (**3ao**)
Following the Cross-Coupling condition C. The crude residue was purified *via* BUCHI C18 reversed phase column to afford a white solid (220 mg, 0.65 mmol, 87% yield).
Using a gradient method from 5-60% water/acetonitrile.
^{1}H NMR (300 MHz, MeOD) δ_H 7.98 (d, *J* = 8.3 Hz, 1H), 7.29 – 7.22 (m, 1H), 7.20 – 7.08 (m, 2H), 7.05 – 6.97 (m, 3H), 6.75 – 6.61 (m, 2H), 3.78 (s, 2H), 3.59 (s, 3H), 1.51 (s, 9H);
^{13}C{^{1}H} NMR (75 MHz, MeOD) δ_C 159.58, 151.13, 137.16, 133.06, 131.79, 130.66, 125.34, 124.22, 123.48, 122.33, 120.56, 116.13, 114.85, 84.62, 55.64, 31.31, 28.44;

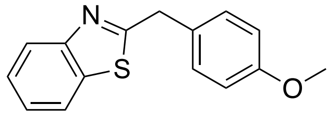

2-(4-methoxybenzyl)benzo[*d*]thiazole (**3ap**)
Following the Cross-Coupling condition C with TBAB (322 mg, 1.0 mmol) as additive. The crude residue was purified *via* BUCHI C18 reversed phase column to afford a yellow oil (149 mg, 0.585 mmol, 78% yield).
Using a gradient method from 5-55% water/acetonitrile.
^{1}H NMR (300 MHz, THF-d$_8$) δ_H 7.90 (ddd, *J* = 8.1, 1.3, 0.6 Hz, 1H), 7.83 (ddd, *J* = 7.9, 1.4, 0.7 Hz, 1H), 7.40 (ddd, *J* = 8.2, 7.2, 1.3 Hz, 1H), 7.33 – 7.24 (m, 3H), 6.89 – 6.82 (m, 2H), 4.33 (s, 2H), 3.74 (s, 3H);
^{13}C{^{1}H} NMR (75 MHz, THF-d$_8$) δ_C 167.89, 155.85, 150.57, 132.63, 126.75, 126.32, 122.23, 121.12, 119.30, 118.04, 110.59, 51.14, 35.92;

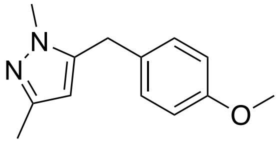

5-(4-methoxybenzyl)-1,3-dimethyl-1*H*-pyrazole (**3aq**)
Following the Cross-Coupling condition C. The crude residue was purified *via* BUCHI C18 reversed phase column to afford a white solid (143 mg, 0.66 mmol, 88% yield).
Using a gradient method from 5-50% water/acetonitrile.
^{1}H NMR (300 MHz, MeOD) δ_H 7.01 – 6.94 (m, 2H), 6.78 – 6.72 (m, 2H), 5.72 – 5.66 (m, 1H), 3.78 (s, 2H), 3.65 (s, 3H), 3.49 (s, 3H), 2.05 (s, 3H);
^{13}C{^{1}H} NMR (75 MHz, MeOD) δ_C 159.94, 148.29, 144.87, 131.00, 130.55, 115.10, 106.44, 55.69, 35.96, 31.52, 13.20;

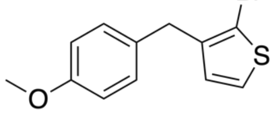

2-bromo-3-(4-methoxybenzyl)thiophene (**3ar**)
Following the Cross-Coupling condition C with TBAB (322 mg, 1.0 mmol) as additive. The crude residue was purified *via* flash chromatography to afford a clear oil (179 mg, 0.637 mmol, 85% yield).
TLC Rf= 0.20 (5% EtOAc/petroleum ether)
^{1}H NMR (300 MHz, $CDCl_3$) δ_H 7.21 – 7.05 (m, 3H), 6.87 – 6.81 (m, 2H), 6.70 (d, *J* = 5.6 Hz, 1H), 3.87 (s, 2H), 3.79 (s, 3H);

$^{13}C\{^1H\}$ NMR: (101 MHz, $CDCl_3$) δ_C 158.13, 140.76, 131.66, 129.54, 128.67, 125.54, 113.96, 109.46, 55.26, 34.59;
HRMS ESI+: (*m/z* calc. for $C_{12}H_{11}BrSO$ $[M]^+$ = 281.9716); found = 281.9714

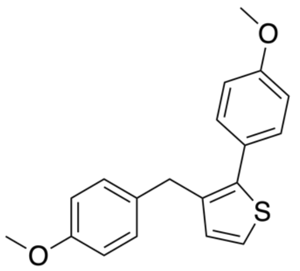

3-(4-methoxybenzyl)-2-(4-methoxyphenyl)thiophene (**3as**)
Following the Cross-Coupling condition B with TBAB (322 mg, 1.0 mmol) as additive. The crude residue was purified *via* flash chromatography to afford a clear oil (204 mg, 0.660 mmol, 88% yield).
TLC Rf= 0.20 (5% EtOAc/petroleum ether)
^1H NMR (300 MHz, $CDCl_3$) δ_H 7.45 – 7.38 (m, 2H), 7.21 (d, *J* = 5.2 Hz, 1H), 7.09 (d, *J* = 8.8 Hz, 2H), 6.97 (d, *J* = 8.7 Hz, 2H), 6.91 – 6.83 (m, 3H), 3.99 (s, 2H), 3.87 (s, 3H), 3.82 (s, 3H);
$^{13}C\{^1H\}$ NMR (75 MHz, $CDCl_3$) δ_C 159.17, 157.93, 138.73, 136.11, 133.32, 130.53, 130.19, 129.44, 126.88, 123.33, 114.03, 113.91, 55.34, 55.28, 33.71;
HRMS ESI+: (*m/z* calc. for $C_{19}H_{18}O_2S$ $[M]^+$ = 310.1026); found = 310.1027

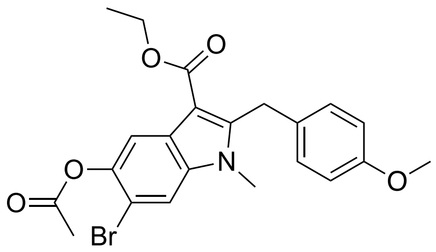

Ethyl 5-acetoxy-6-bromo-2-(4-methoxybenzyl)-1-methyl-1*H*-indole-3-carboxylate (**3at**)
Following the Cross-Coupling condition C with TBAB (322 mg, 1.0 mmol) as additive. The crude residue was purified *via* BUCHI C18 reversed phase column to afford a brown solid (317 mg, 0.69 mmol, 92% yield).
Using a gradient method from 5-65% water/acetonitrile.
^1H NMR (300 MHz, $CDCl_3$) δ_H 7.92 (s, 1H), 7.51 (s, 1H), 7.07 – 7.01 (m, 2H), 6.82 – 6.78 (m, 2H), 4.60 (s, 2H), 4.40 (t, *J* = 7.1 Hz, 2H), 3.76 (s, 3H), 3.54 (s, 3H), 2.40 (s, 3H), 1.40 (t, *J* = 7.1 Hz, 3H);
$^{13}C\{^1H\}$ NMR (75 MHz, $CDCl_3$) δ_C 169.54, 165.24, 158.29, 148.26, 143.04, 135.33, 129.11, 128.95, 126.41, 115.97, 114.19, 113.48, 110.30, 105.25, 59.80, 55.27, 30.51, 30.28, 20.90, 14.57;

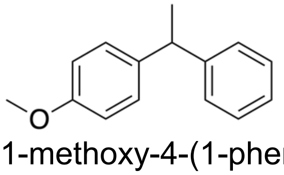

1-methoxy-4-(1-phenylethyl)benzene (**3au**)
Following the Cross-Coupling condition C with 1 mol% catalyst loading. The crude residue was purified *via* flash chromatography to afford a white solid (97 mg, 0.457 mmol, 61% yield).
TLC Rf= 0.20 (5% EtOAc/petroleum ether)
^1H NMR (300 MHz, $CDCl_3$) δ_H 7.34 – 7.15 (m, 7H), 6.92 – 6.81 (m, 2H), 4.14 (q, *J* = 7.2 Hz, 1H), 3.81 (s, 3H), 1.65 (d, *J* = 7.2 Hz, 3H);
$^{13}C\{^1H\}$ NMR (75 MHz, $CDCl_3$) δ_C 157.81, 146.78, 138.57, 128.53, 128.36, 127.55, 125.96, 113.72, 55.26, 43.94, 22.09;
HRMS ESI+: (*m/z* calc. for $C_{15}H_{16}O$ $[M]^+$ = 212.1202); found = 212.1201

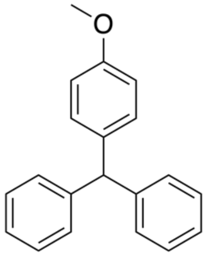

((4-methoxyphenyl)methylene)dibenzene (**3av**)

Following the Cross-Coupling condition C with TBAB (322 mg, 1.0 mmol) as additive. The crude residue was purified *via* flash chromatography to afford a white solid (104 mg, 0.382 mmol, 51% yield).

TLC Rf= 0.20 (petroleum ether)

^{1}H NMR (300 MHz, $CDCl_3$) δ_H 7.34 – 7.27 (m, 4H), 7.26 – 7.18 (m, 2H), 7.15 (d, *J* = 7.3 Hz, 4H), 7.06 (d, *J* = 8.2 Hz, 2H), 6.86 (d, *J* = 8.7 Hz, 2H), 5.54 (s, 1H), 3.81 (s, 3H);

^{13}C{^{1}H} NMR (75 MHz, $CDCl_3$) δ_C 158.05, 144.27, 136.12, 130.38, 129.39, 128.28, 126.23, 113.69, 56.04, 55.24;

HRMS ESI+: (*m/z* calc. for $C_{20}H_{18}O$ $[M]^+$ = 273.1279); found = 273.1285

Spectra see the following pages

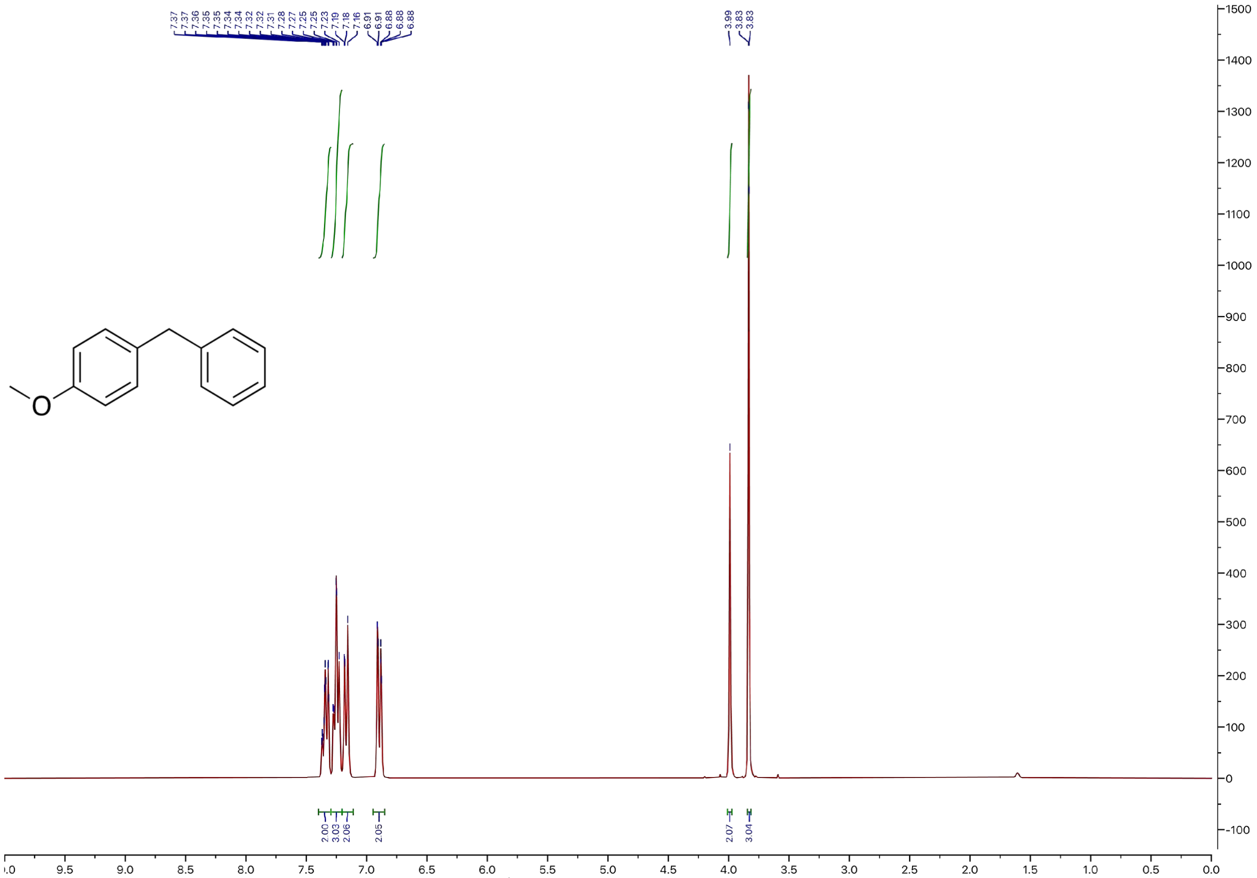

Supplementary Figure 46. ^{1}H NMR (300 MHz, $CDCl_3$) spectrum of **3aa**.

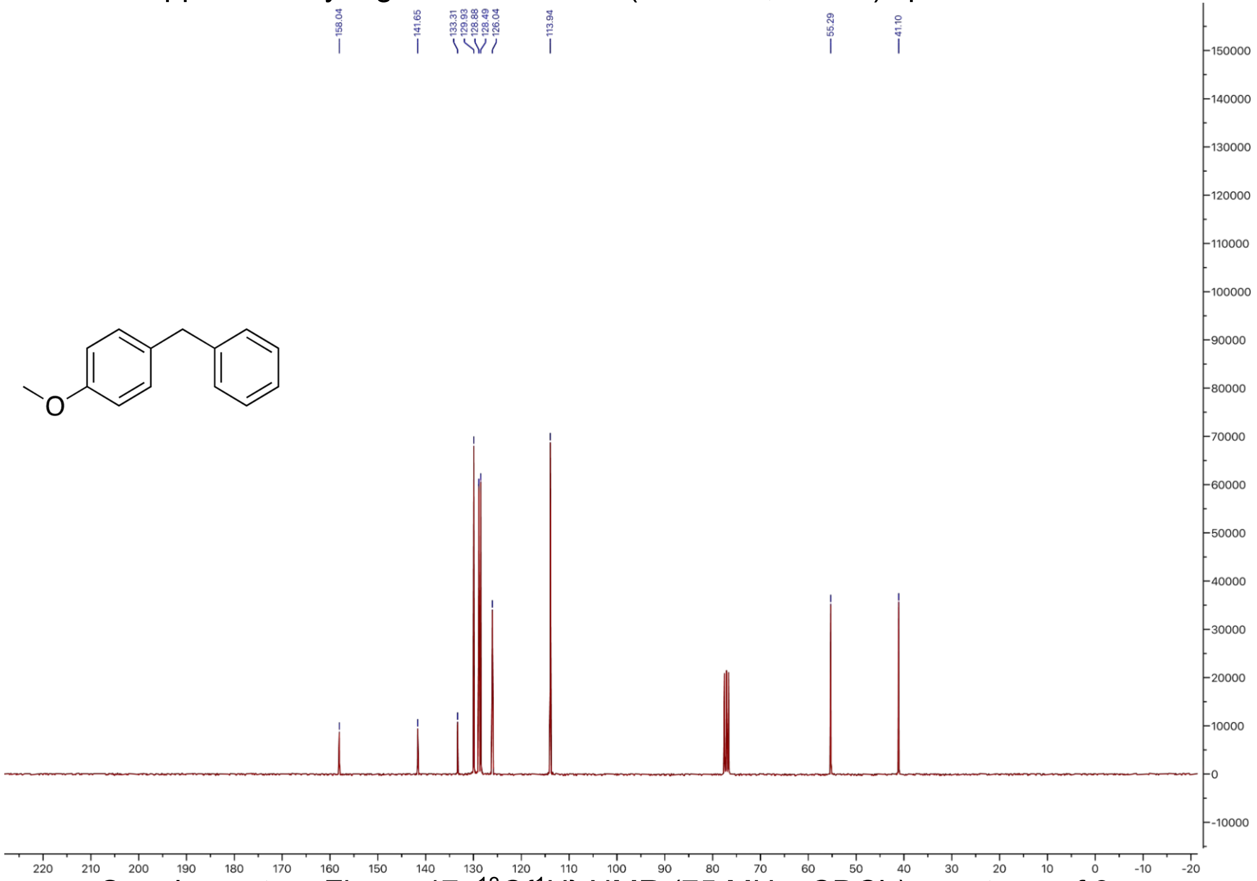

Supplementary Figure 47. $^{13}C\{^{1}H\}$ NMR (75 MHz, $CDCl_3$) spectrum of **3aa**.

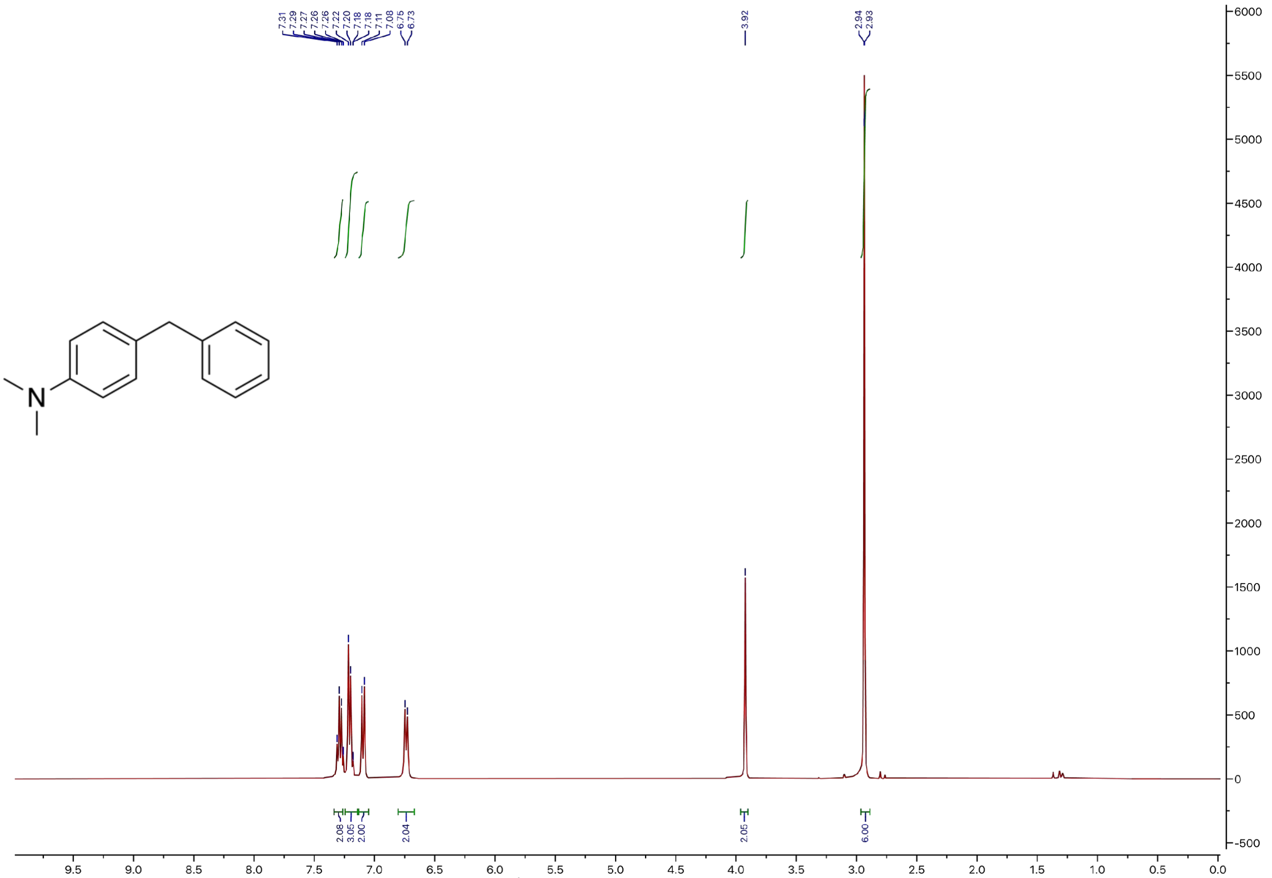

Supplementary Figure 48. ^{1}H NMR (400 MHz, $CDCl_3$) spectrum of **3ba**.

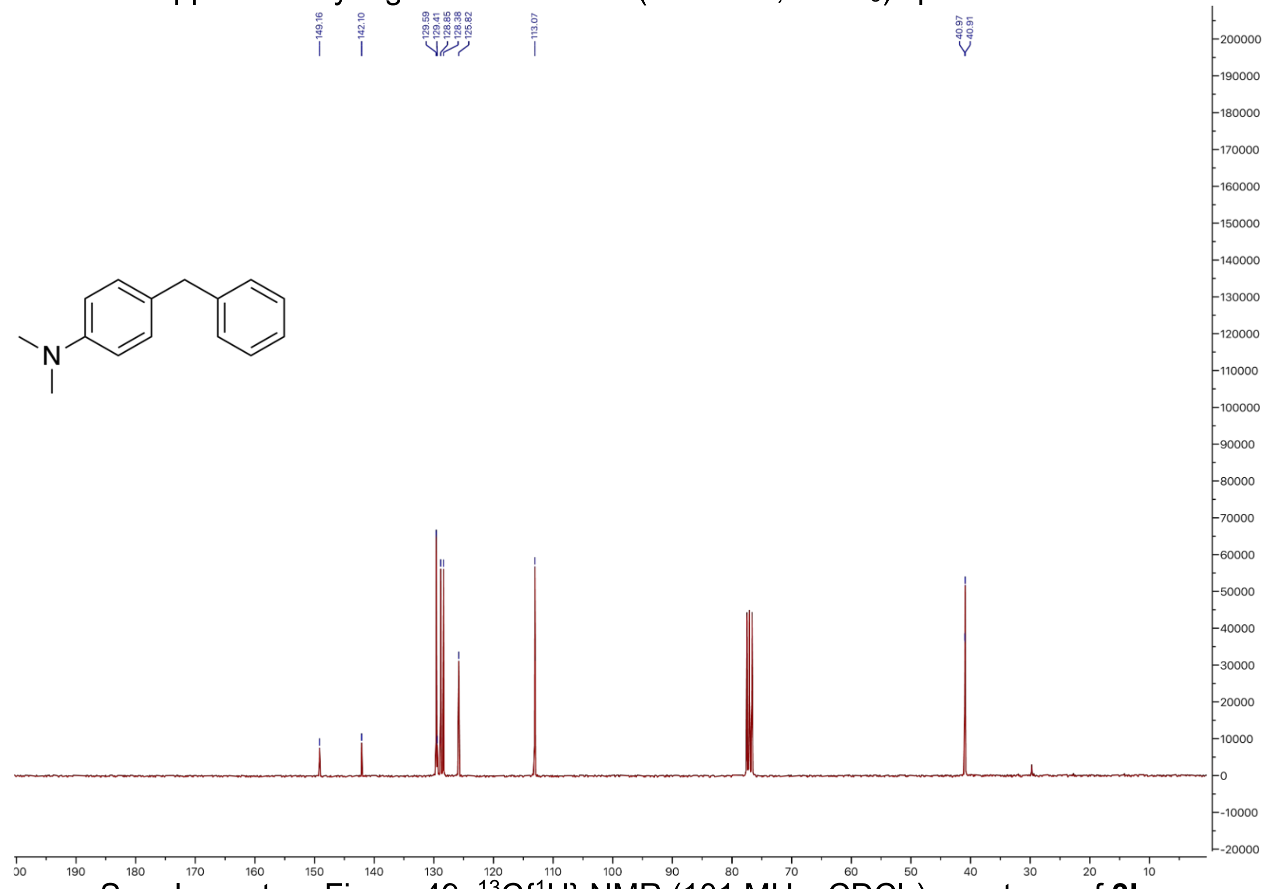

Supplementary Figure 49. $^{13}C\{^{1}H\}$ NMR (101 MHz, $CDCl_3$) spectrum of **3ba**.

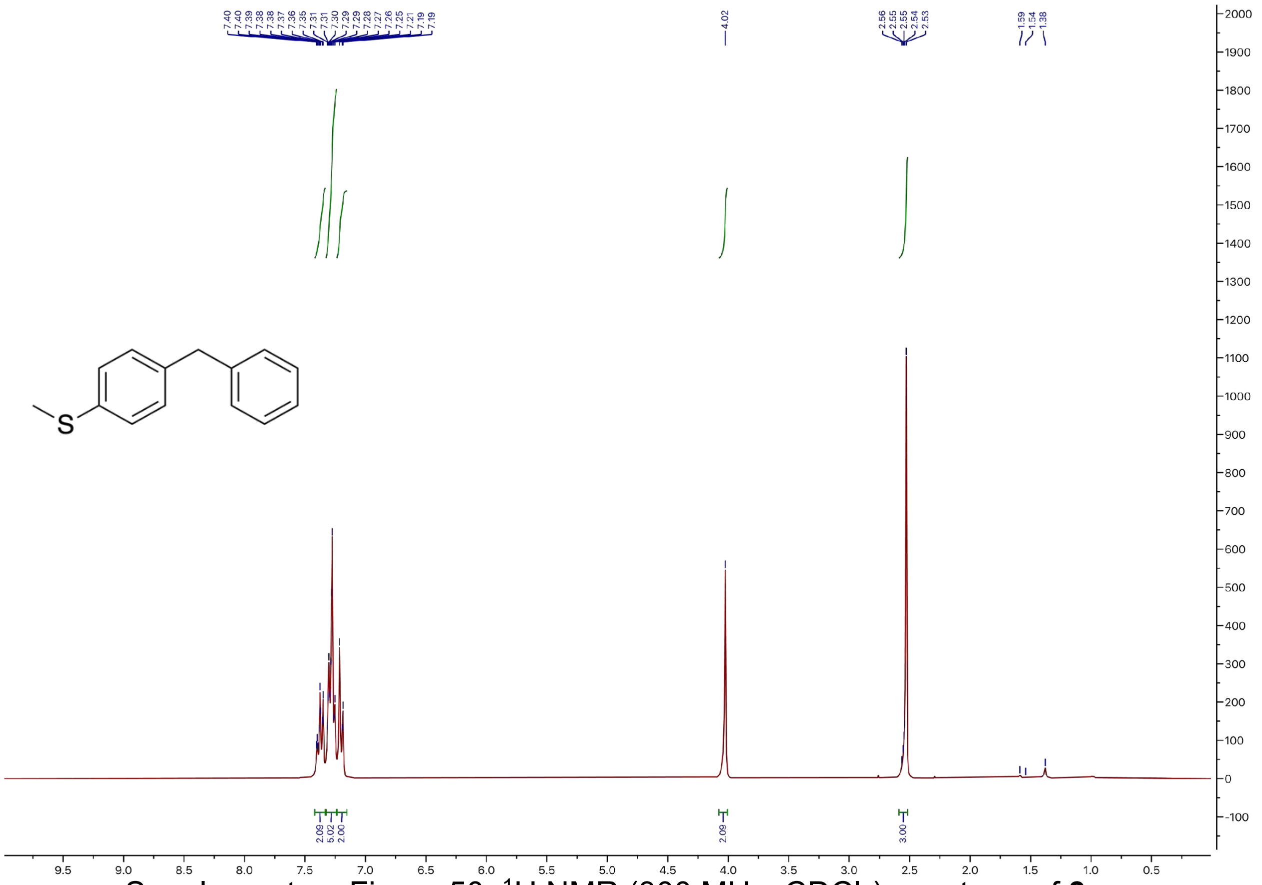

Supplementary Figure 50. ^{1}H NMR (300 MHz, $CDCl_3$) spectrum of **3ca**.

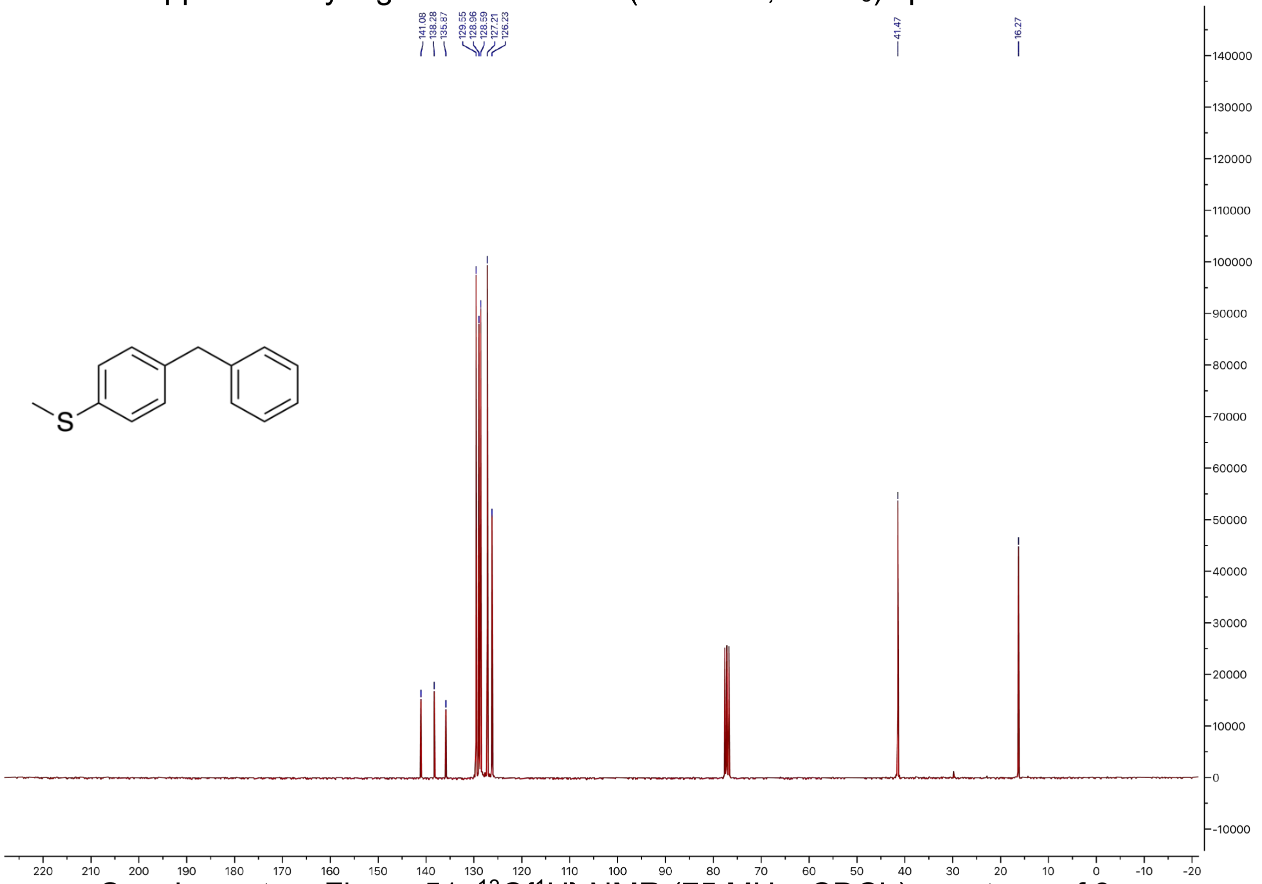

Supplementary Figure 51. $^{13}C\{^{1}H\}$ NMR (75 MHz, $CDCl_3$) spectrum of **3ca**.

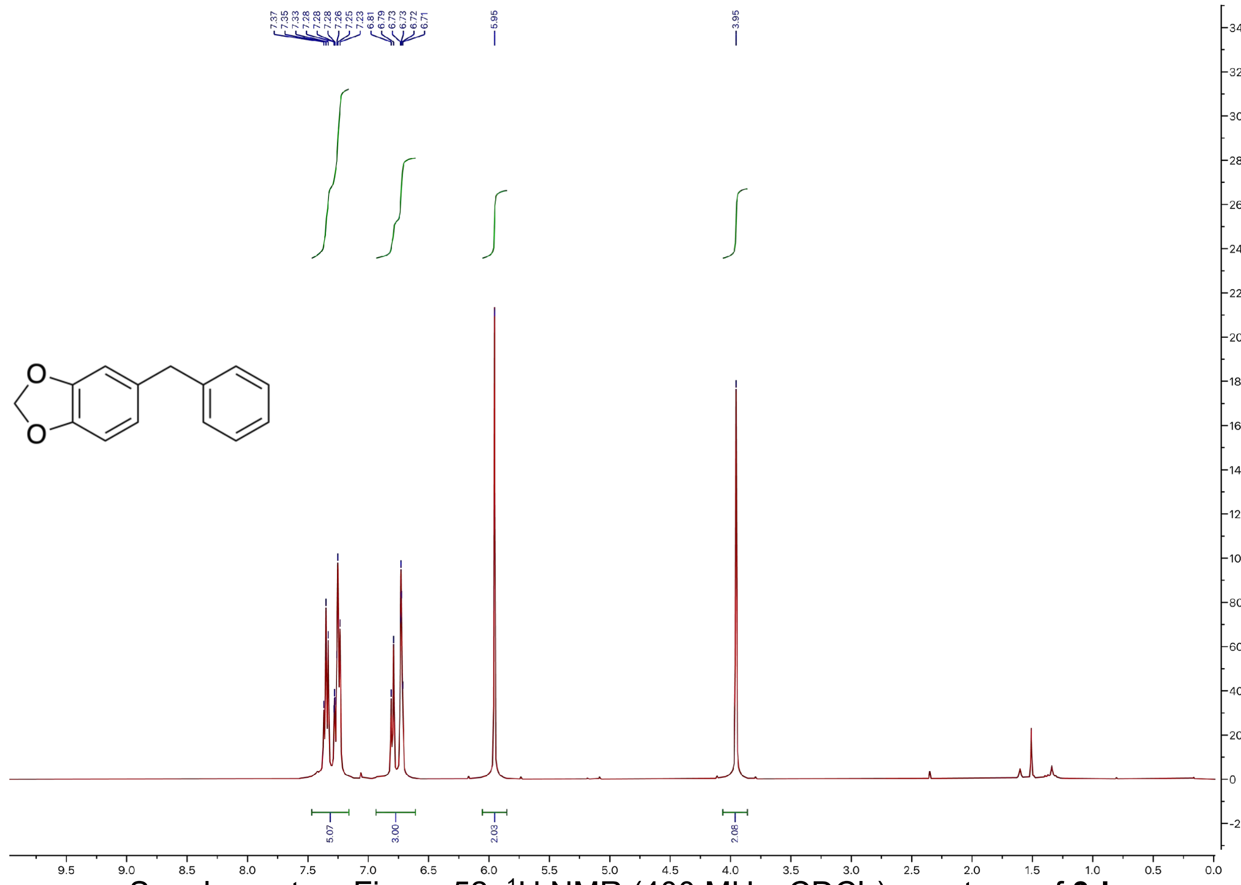

Supplementary Figure 52. ^{1}H NMR (400 MHz, $CDCl_3$) spectrum of **3da**.

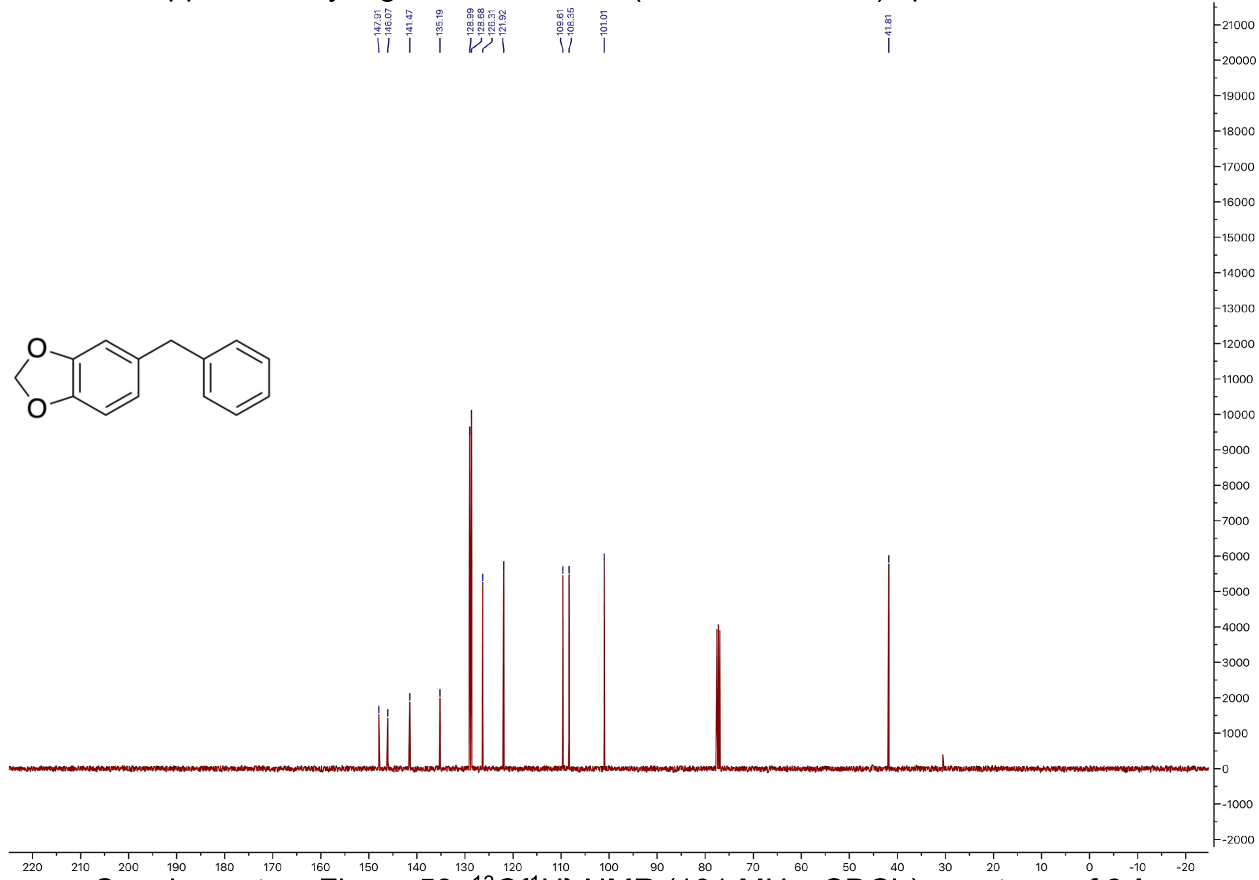

Supplementary Figure 53. $^{13}C\{^{1}H\}$ NMR (101 MHz, $CDCl_3$) spectrum of **3da**.

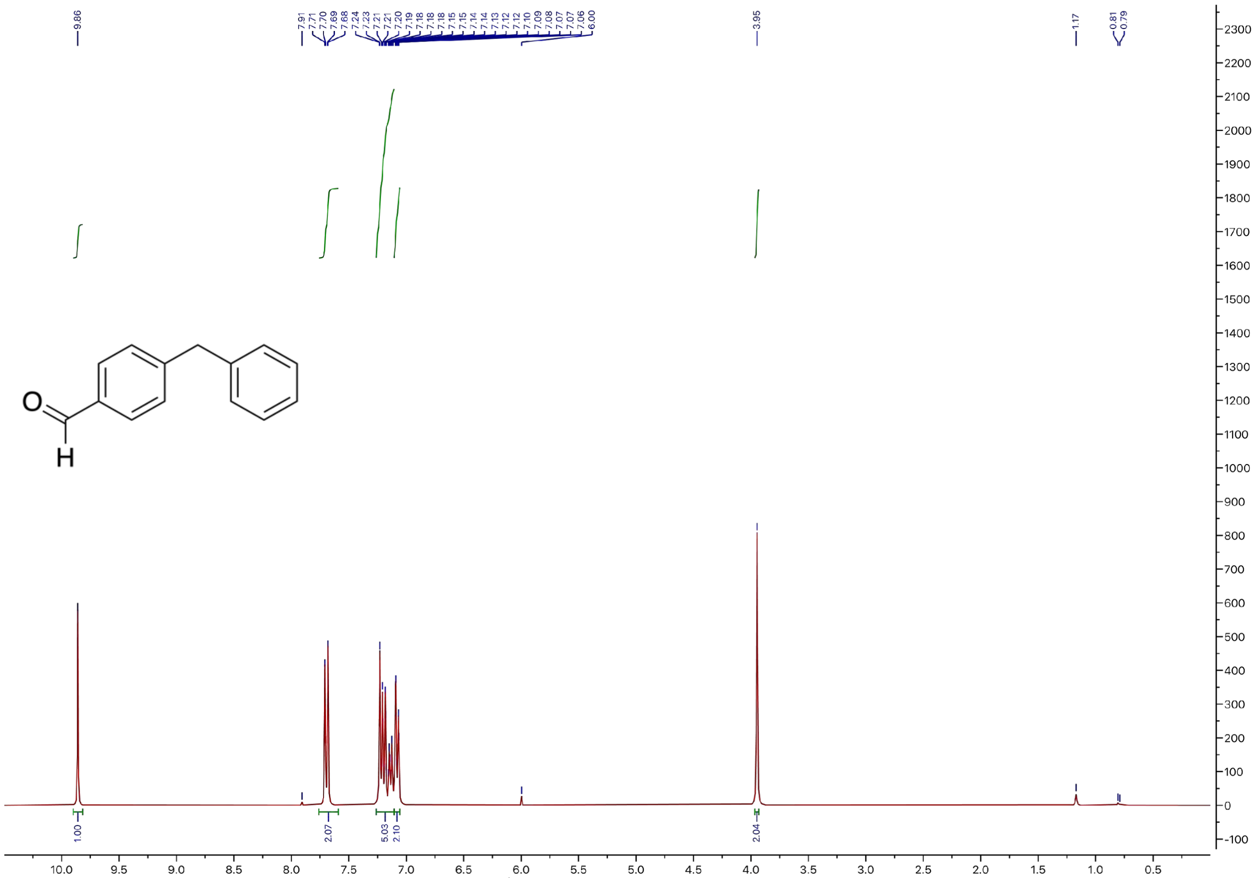

Supplementary Figure 54. ^{1}H NMR (300 MHz, $CDCl_3$) spectrum of **3ea**.

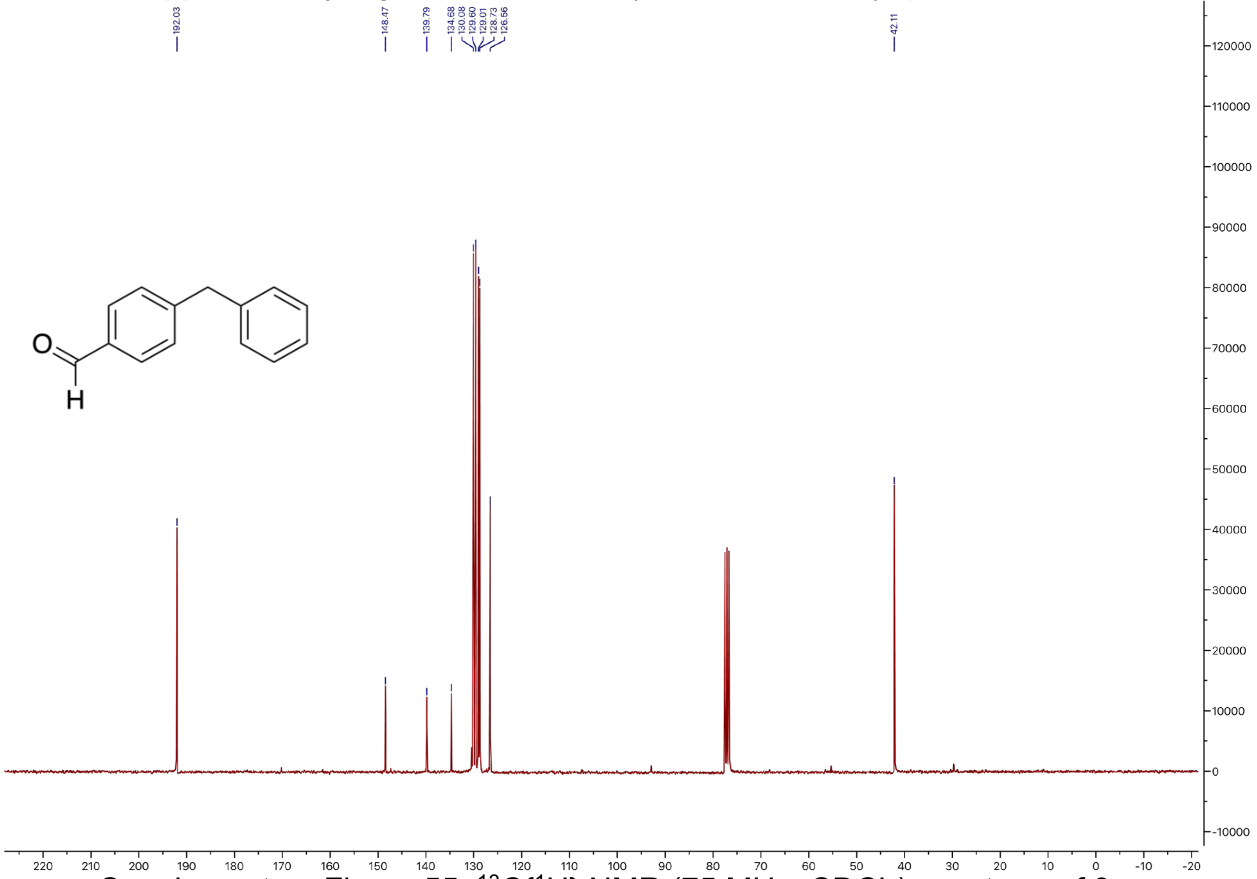

Supplementary Figure 55. $^{13}C\{^{1}H\}$ NMR (75 MHz, $CDCl_3$) spectrum of **3ea**.

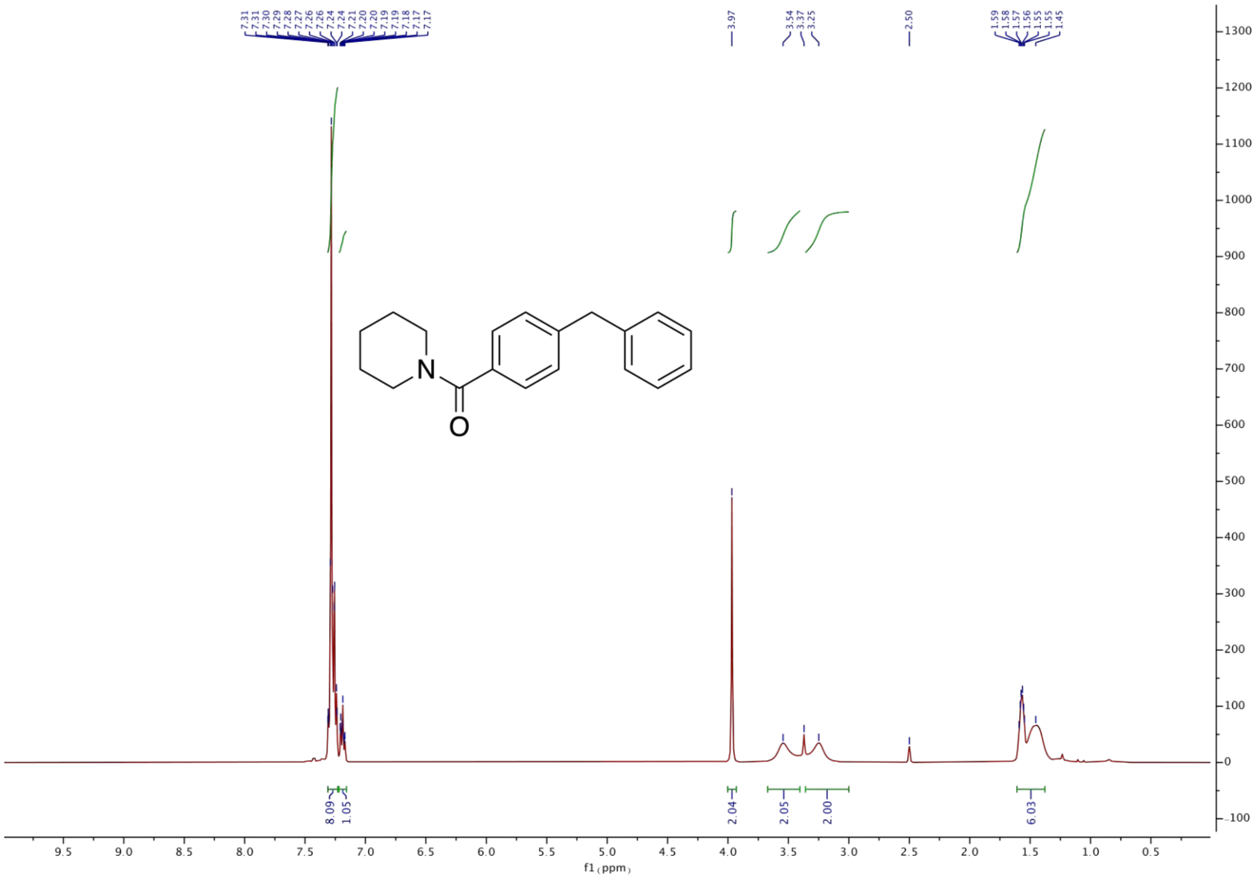

Supplementary Figure 56. ^{1}H NMR (400 MHz, DMSO-d6) spectrum of **3fa**.

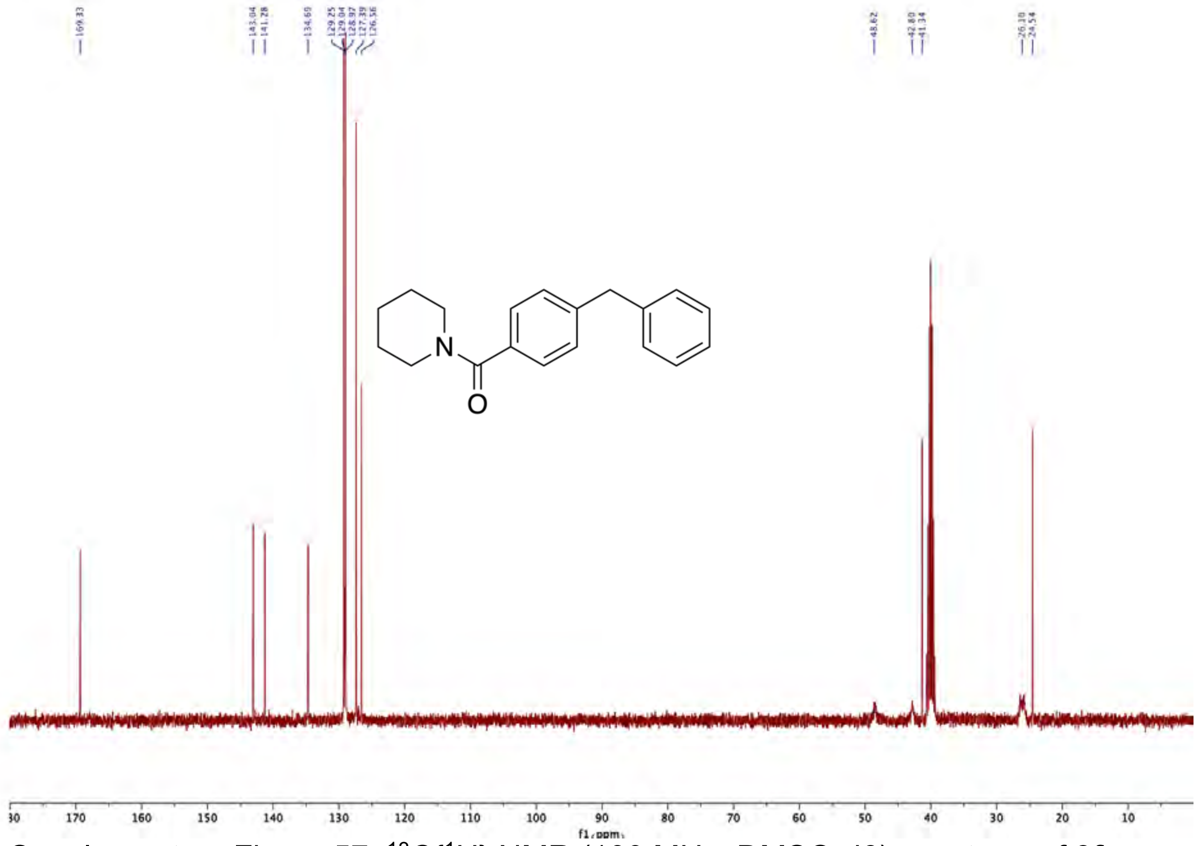

Supplementary Figure 57. $^{13}C\{^{1}H\}$ NMR (100 MHz, DMSO-d6) spectrum of **3fa**.

a.

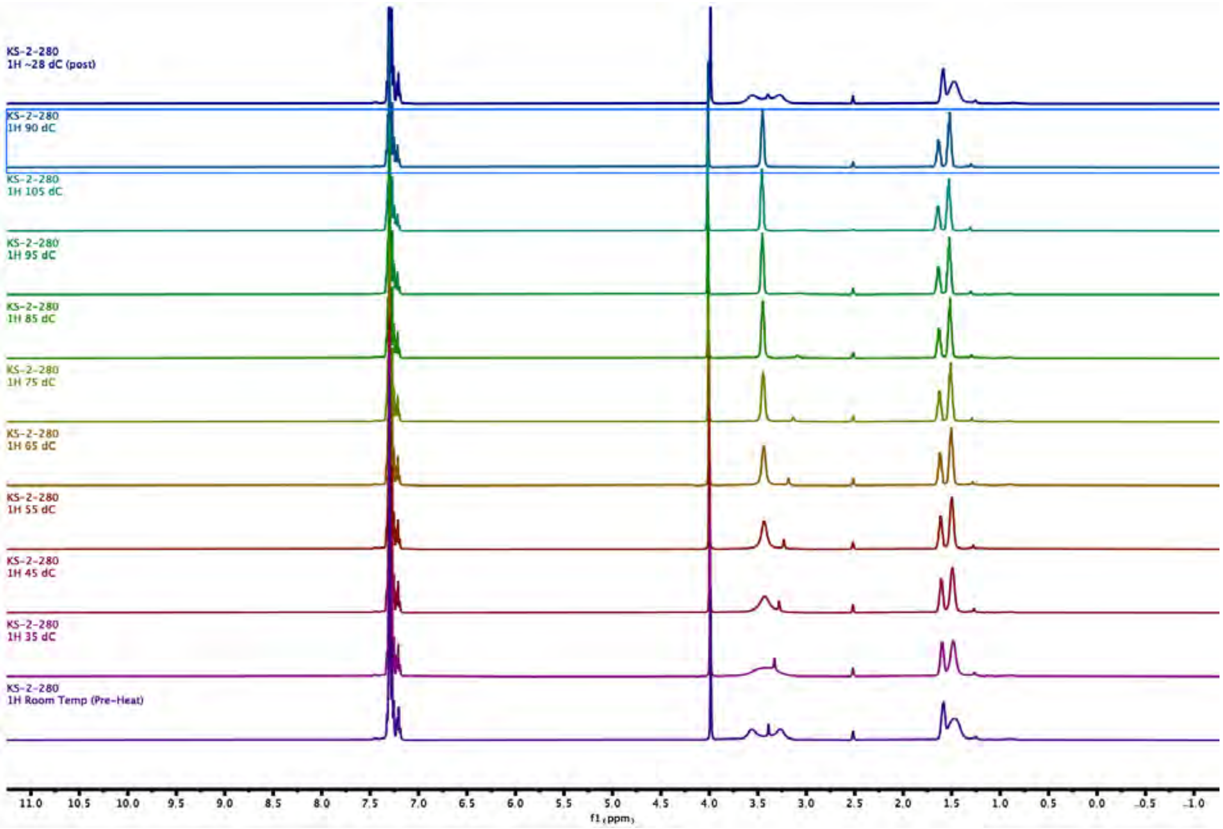

b.

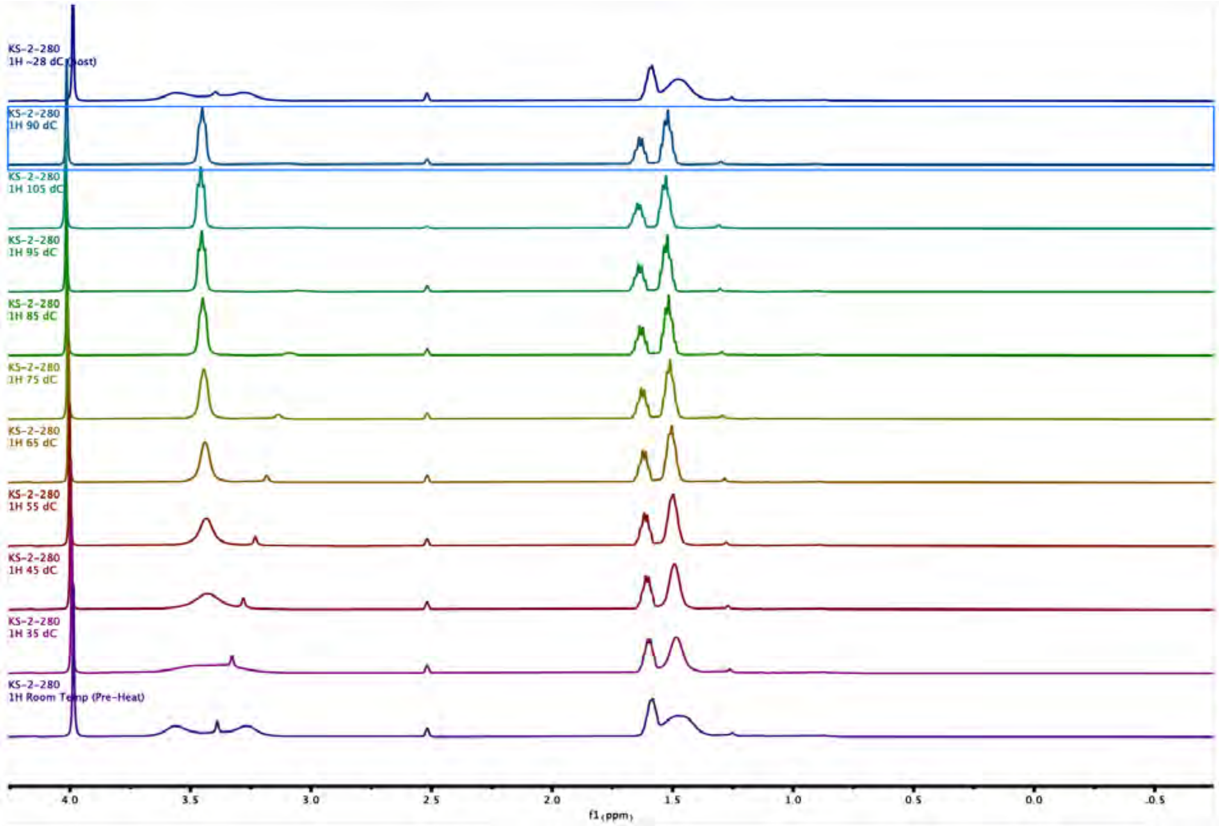

Supplementary Figure 58. a. Stacked ^{1}H NMR (400 MHz, DMSO-d6) spectrum of **3fa** measured at variable temperatures. b. Expansion of region of interest.

a.

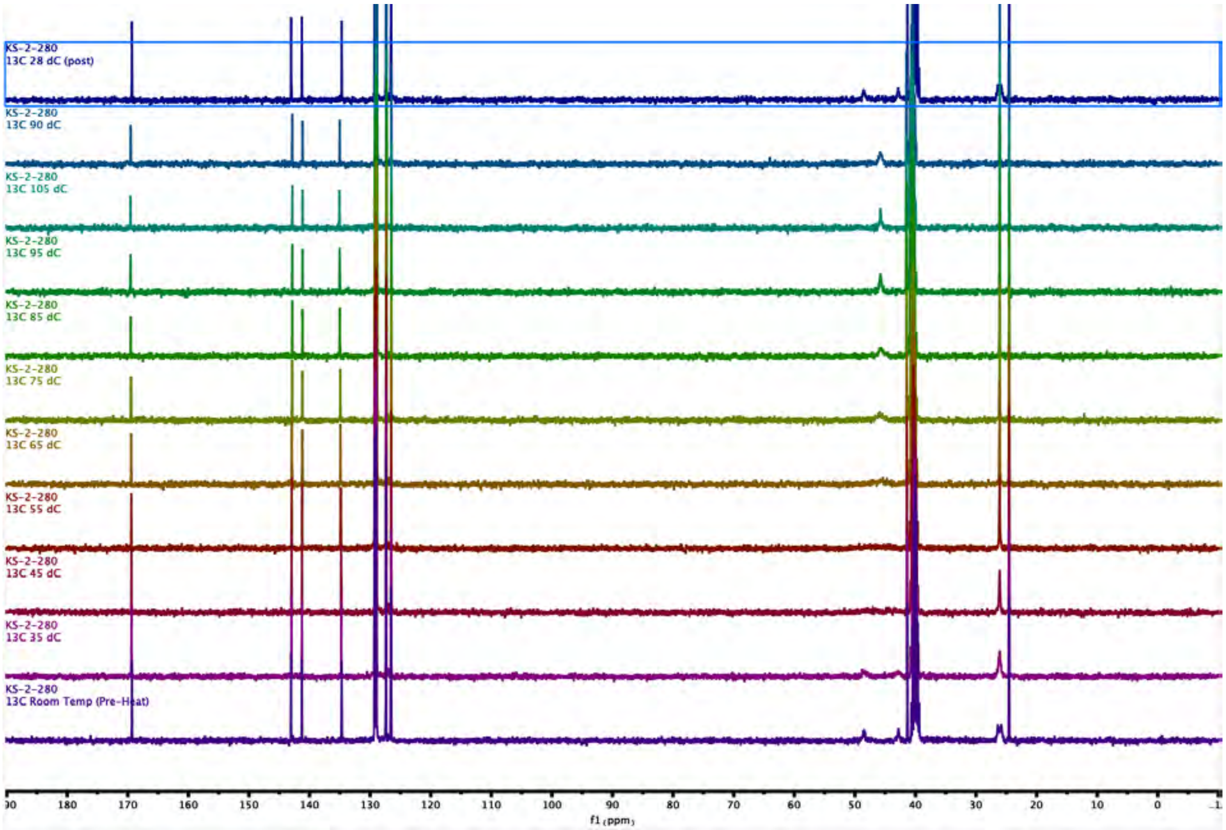

b.

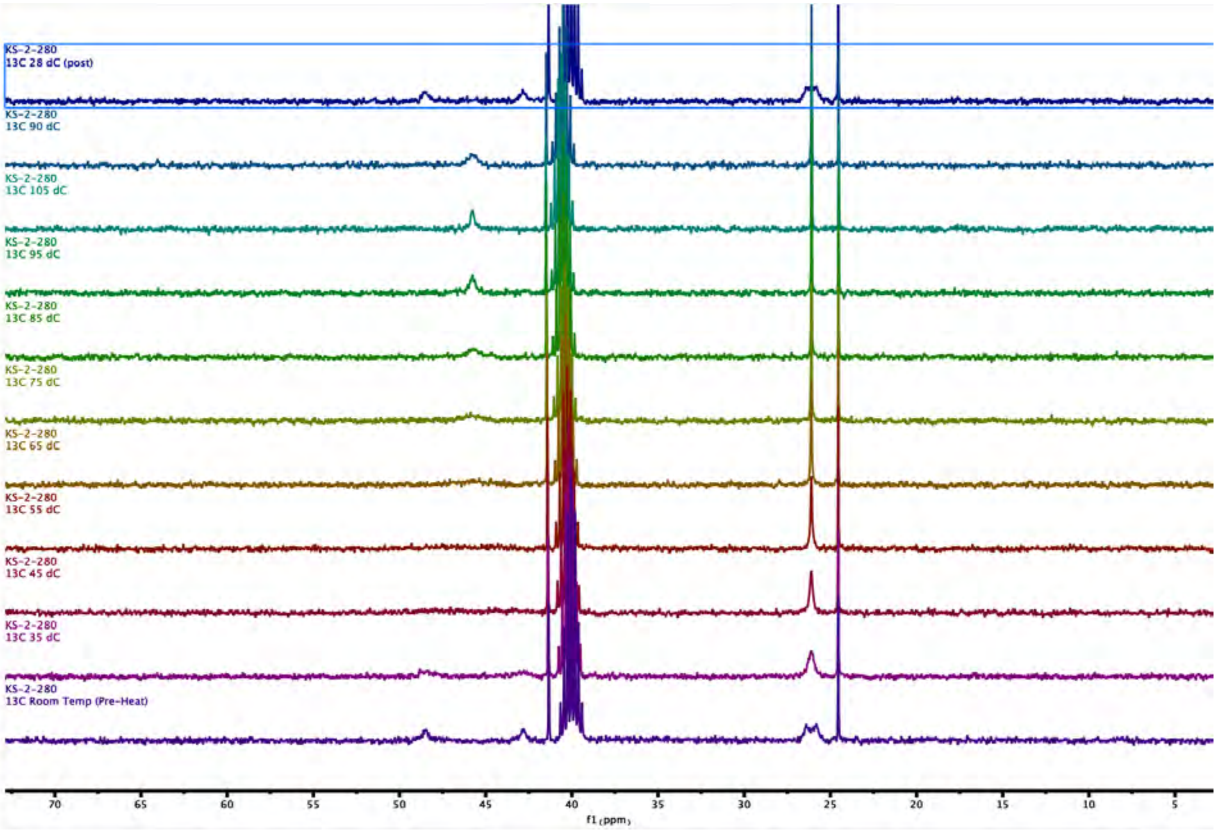

Supplementary Figure 59. a. Stacked $^{13}C\{^{1}H\}$ NMR (100 MHz, DMSO-d6) spectrum of **3fa** measured at variable temperatures. b. Expansion of region of interest.

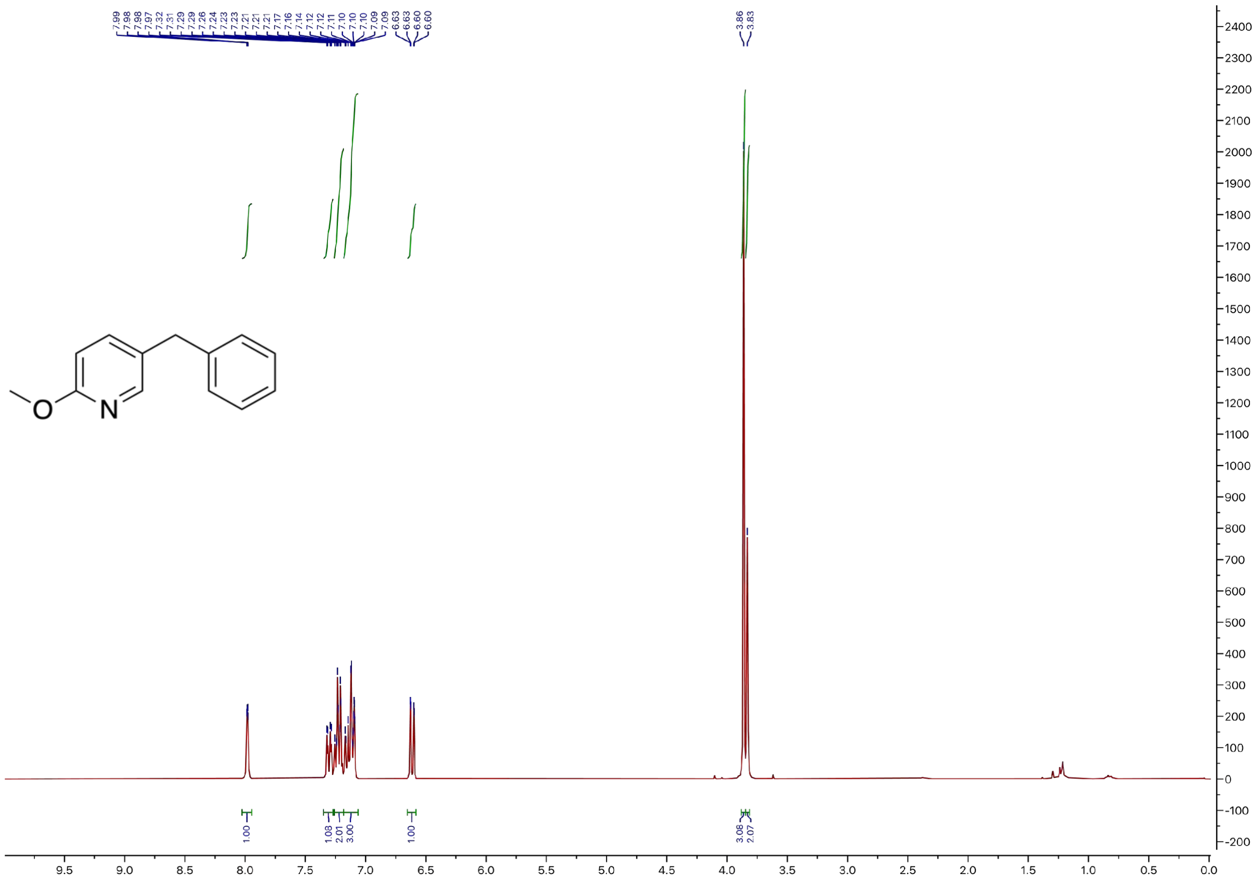

Supplementary Figure 60. ^{1}H NMR (300 MHz, $CDCl_3$) spectrum of **3ga**.

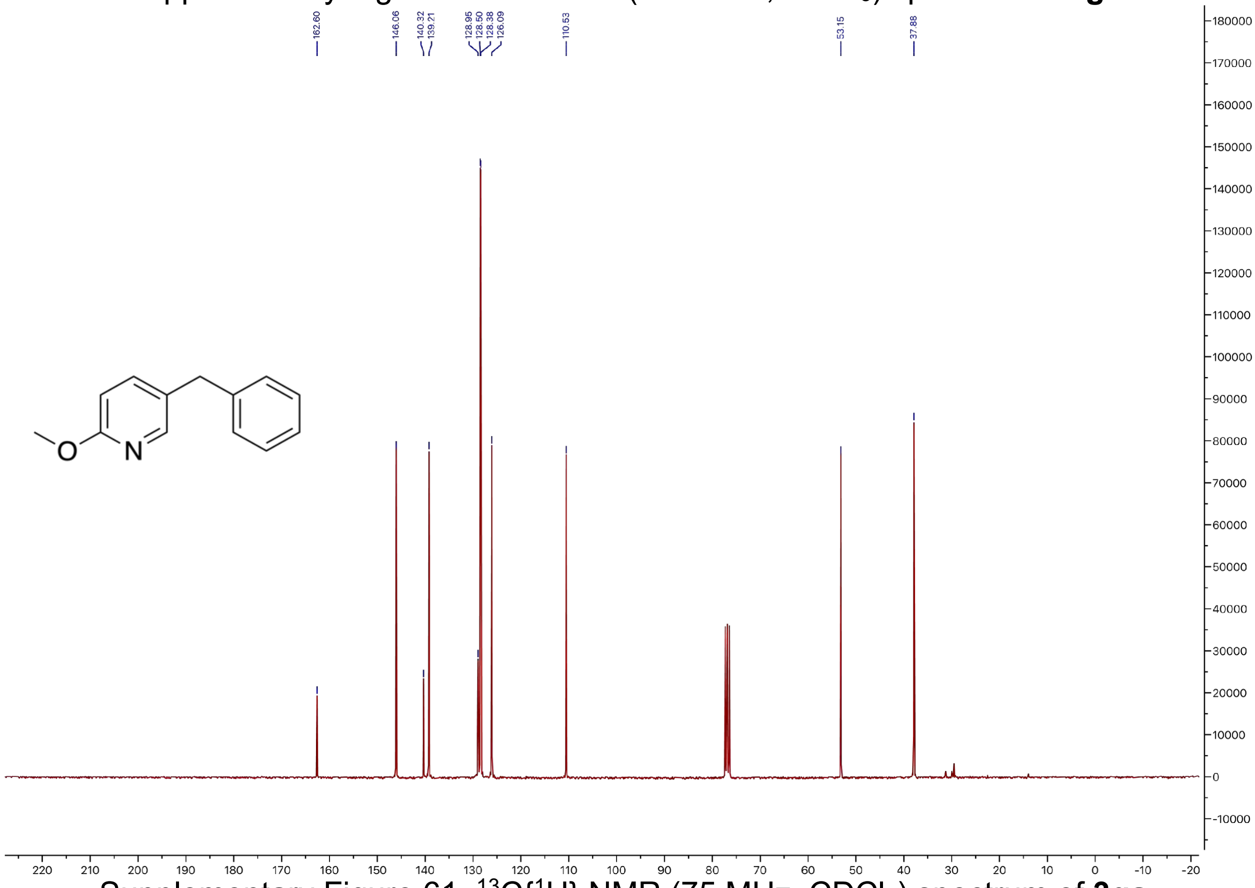

Supplementary Figure 61. $^{13}C\{^{1}H\}$ NMR (75 MHz, $CDCl_3$) spectrum of **3ga**.

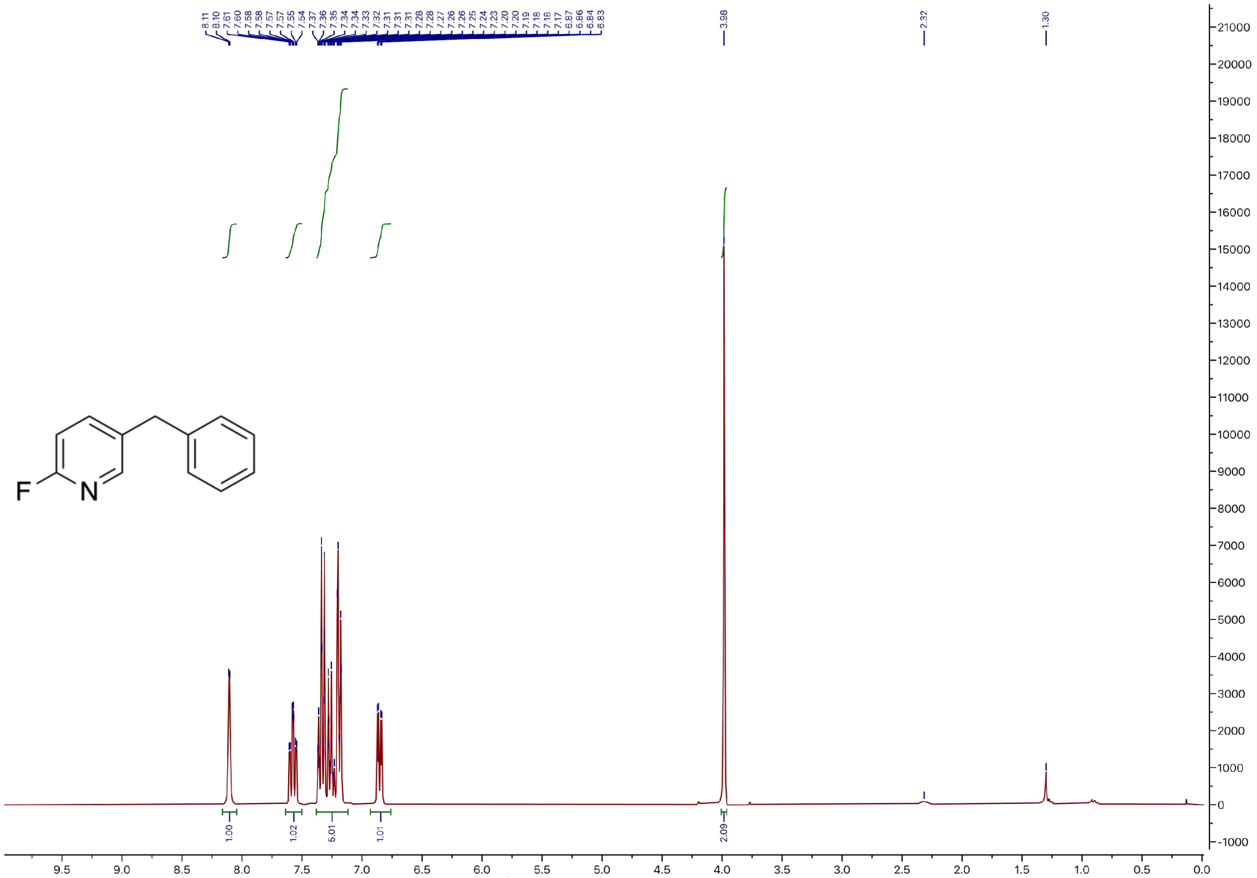

Supplementary Figure 62. ^{1}H NMR (300 MHz, $CDCl_3$) spectrum of **3ha**.

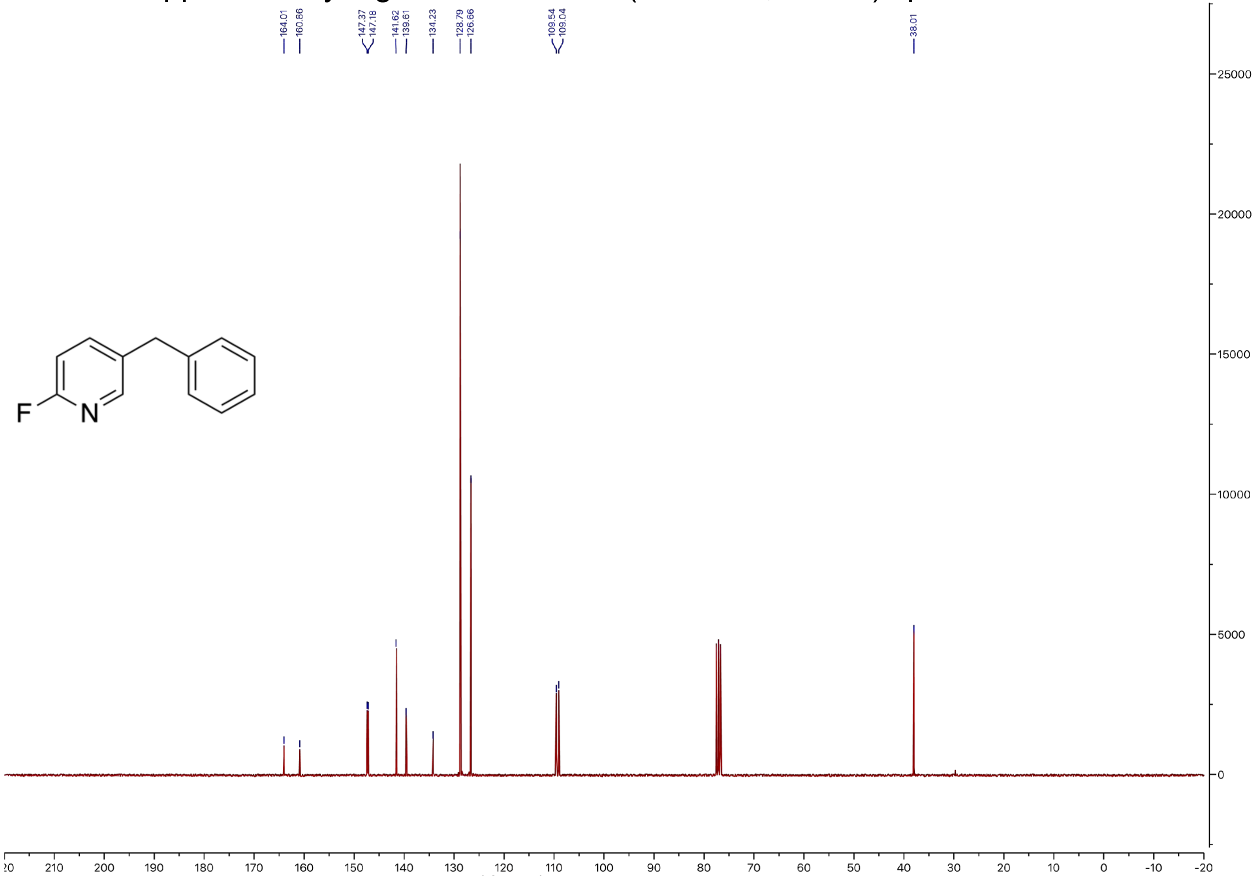

Supplementary Figure 63. $^{13}C\{^{1}H\}$ NMR (75 MHz, $CDCl_3$) spectrum of **3ha**.

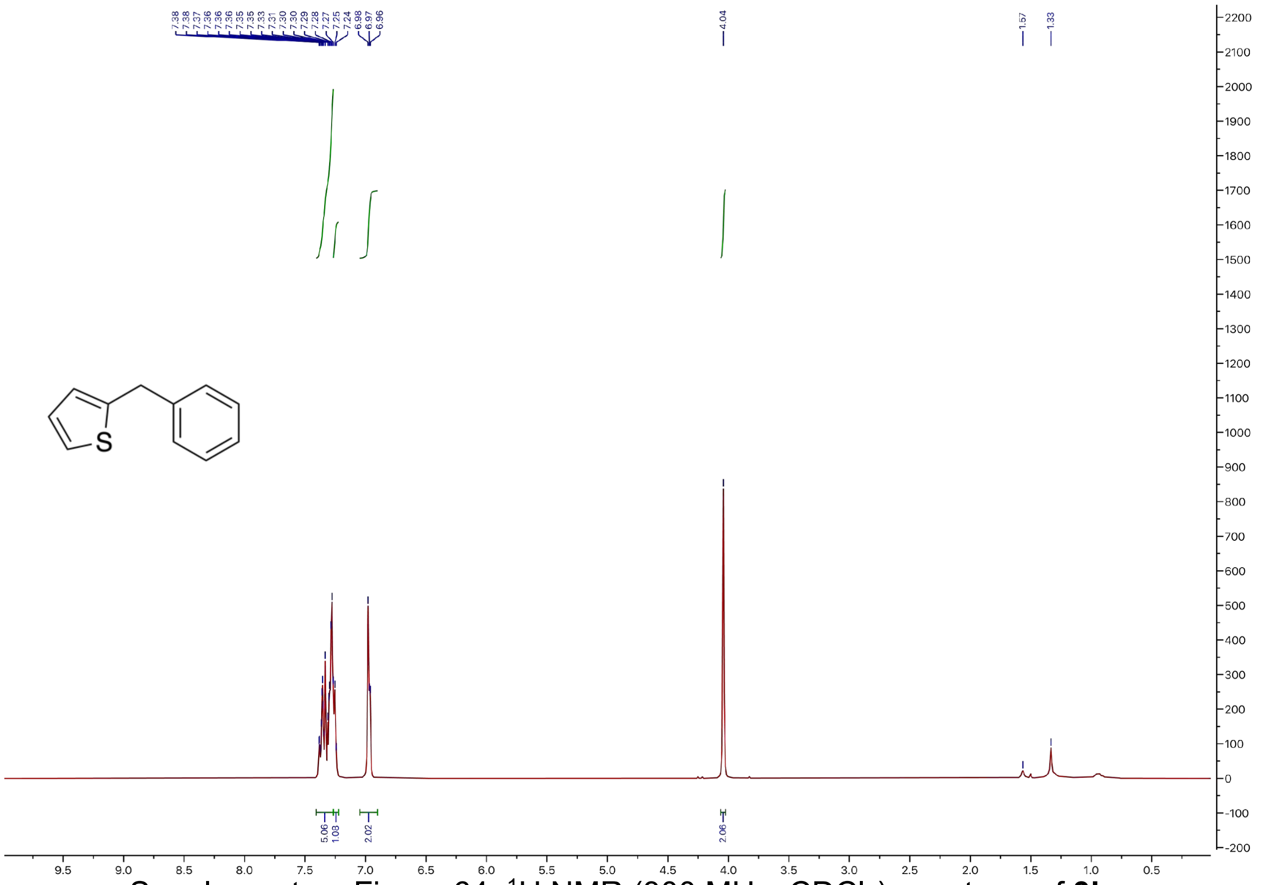

Supplementary Figure 64. ^{1}H NMR (300 MHz, $CDCl_3$) spectrum of **3ia**.

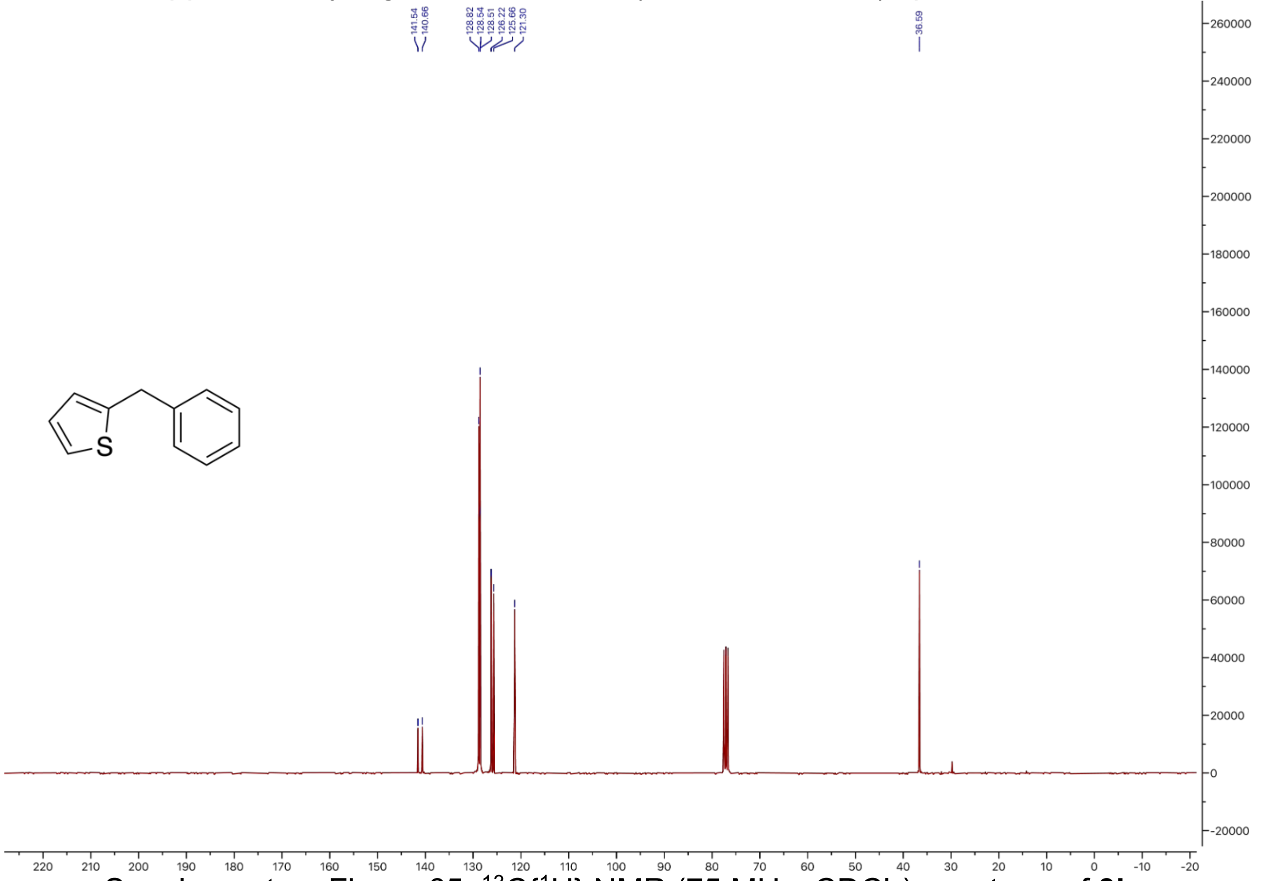

Supplementary Figure 65. $^{13}C\{^{1}H\}$ NMR (75 MHz, $CDCl_3$) spectrum of **3ia**.

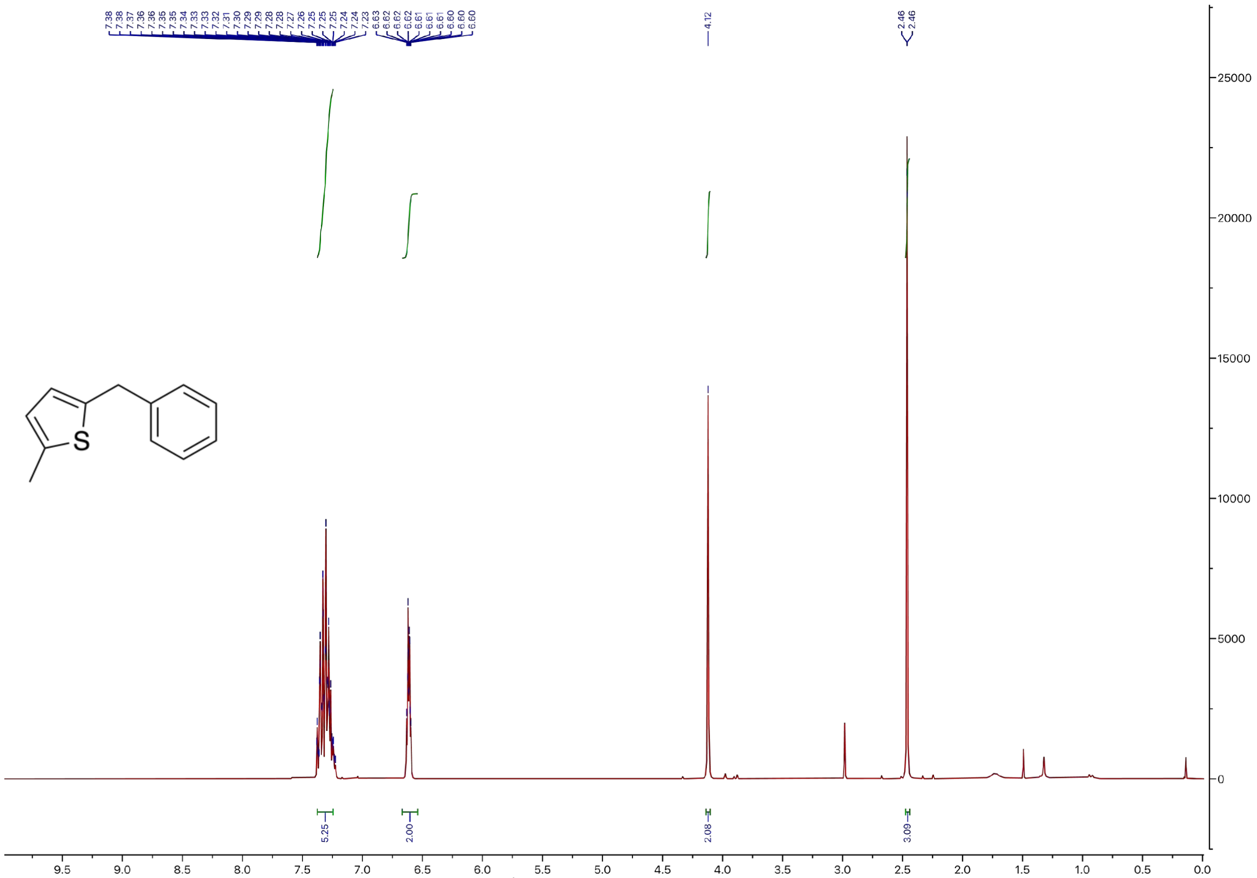

Supplementary Figure 66. ^{1}H NMR (300 MHz, $CDCl_3$) spectrum of **3ja**.

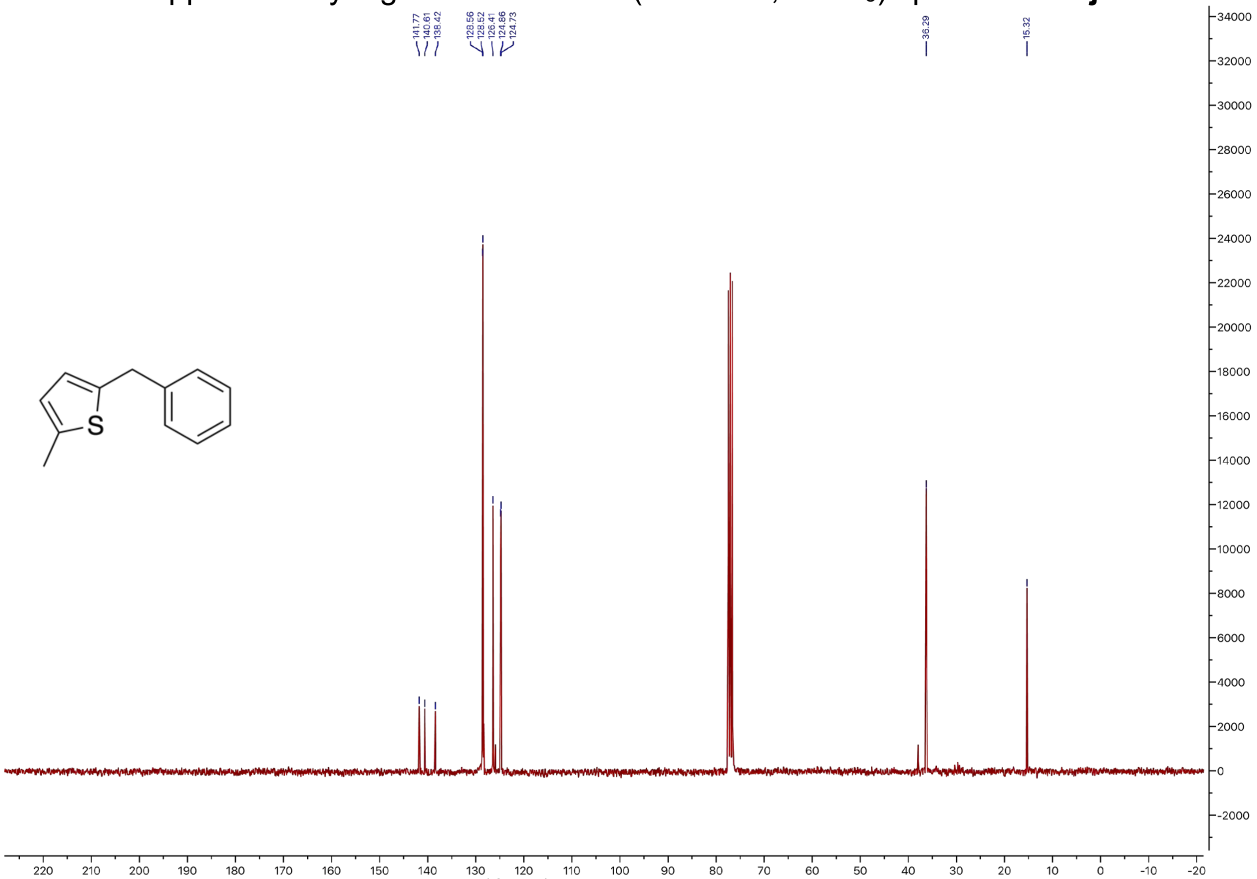

Supplementary Figure 67. $^{13}C\{^{1}H\}$ NMR (75 MHz, $CDCl_3$) spectrum of **3ja**.

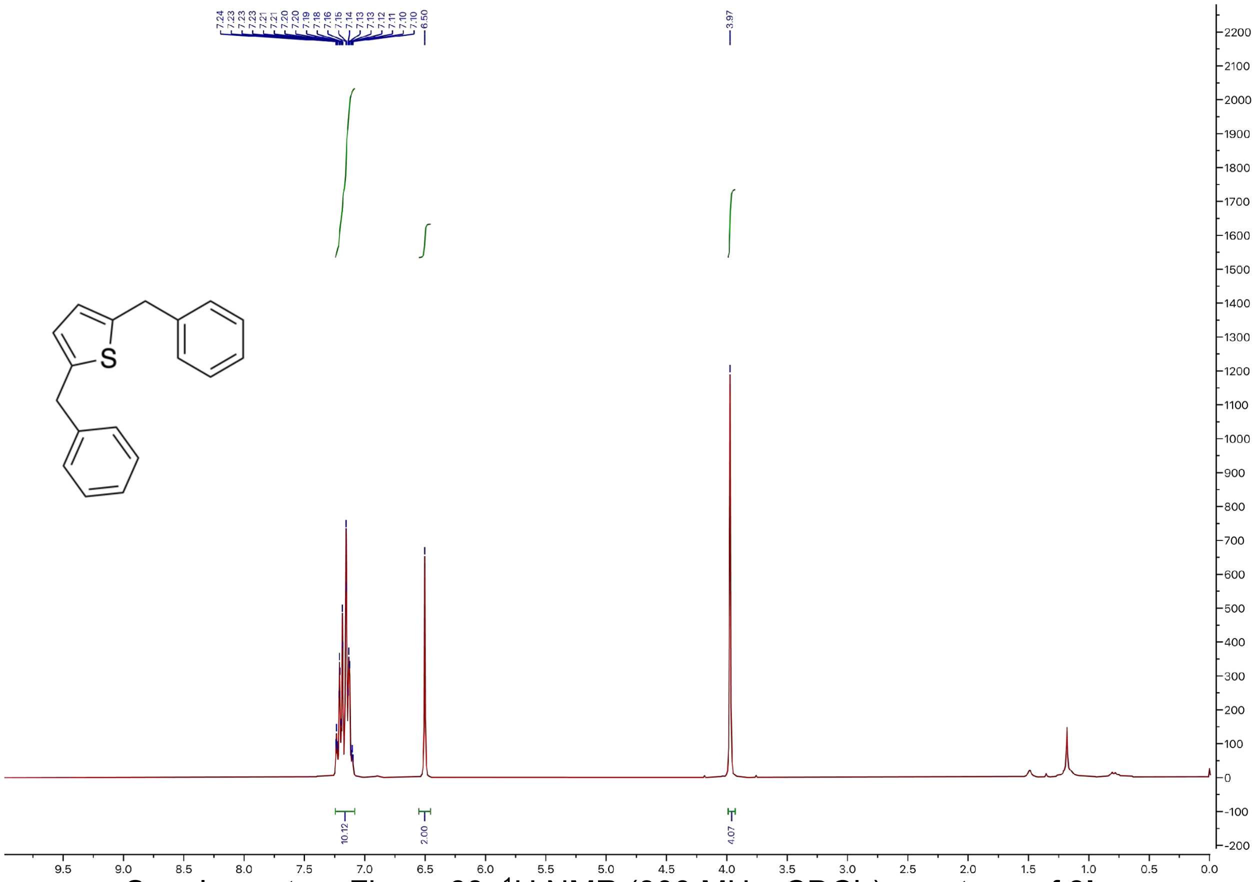

Supplementary Figure 68. ^{1}H NMR (300 MHz, $CDCl_3$) spectrum of **3ka**.

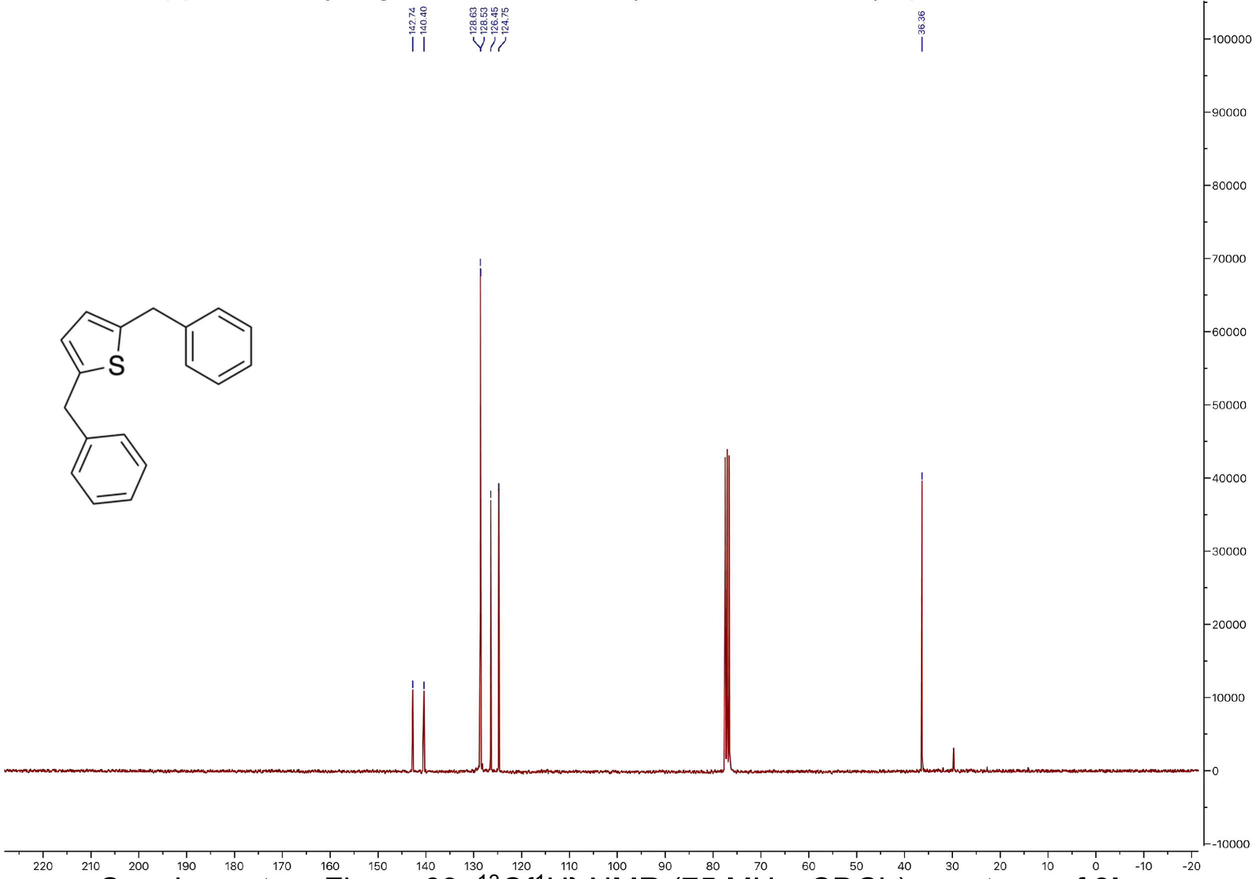

Supplementary Figure 69. $^{13}C\{^{1}H\}$ NMR (75 MHz, $CDCl_3$) spectrum of **3ka**.

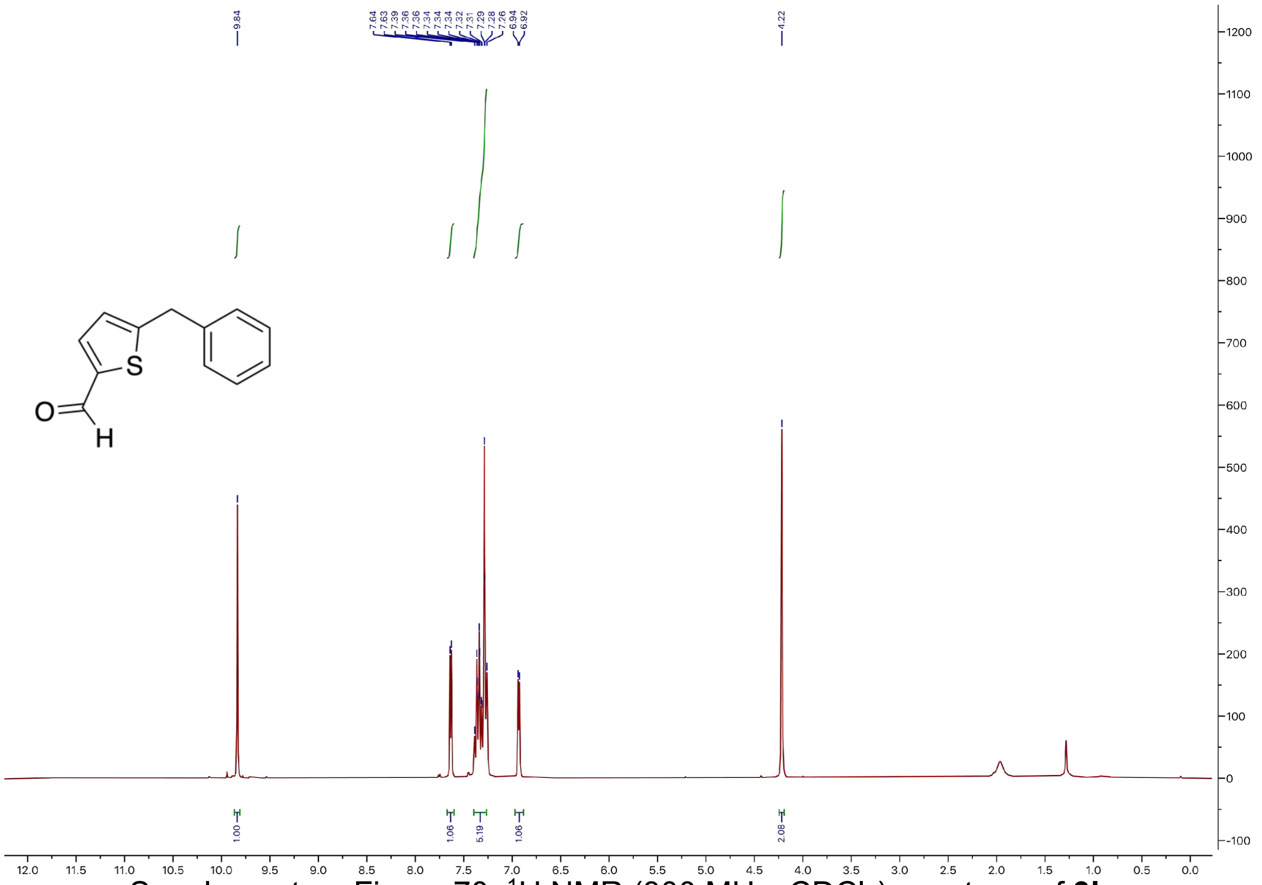

Supplementary Figure 70. ^{1}H NMR (300 MHz, $CDCl_3$) spectrum of **3la**.

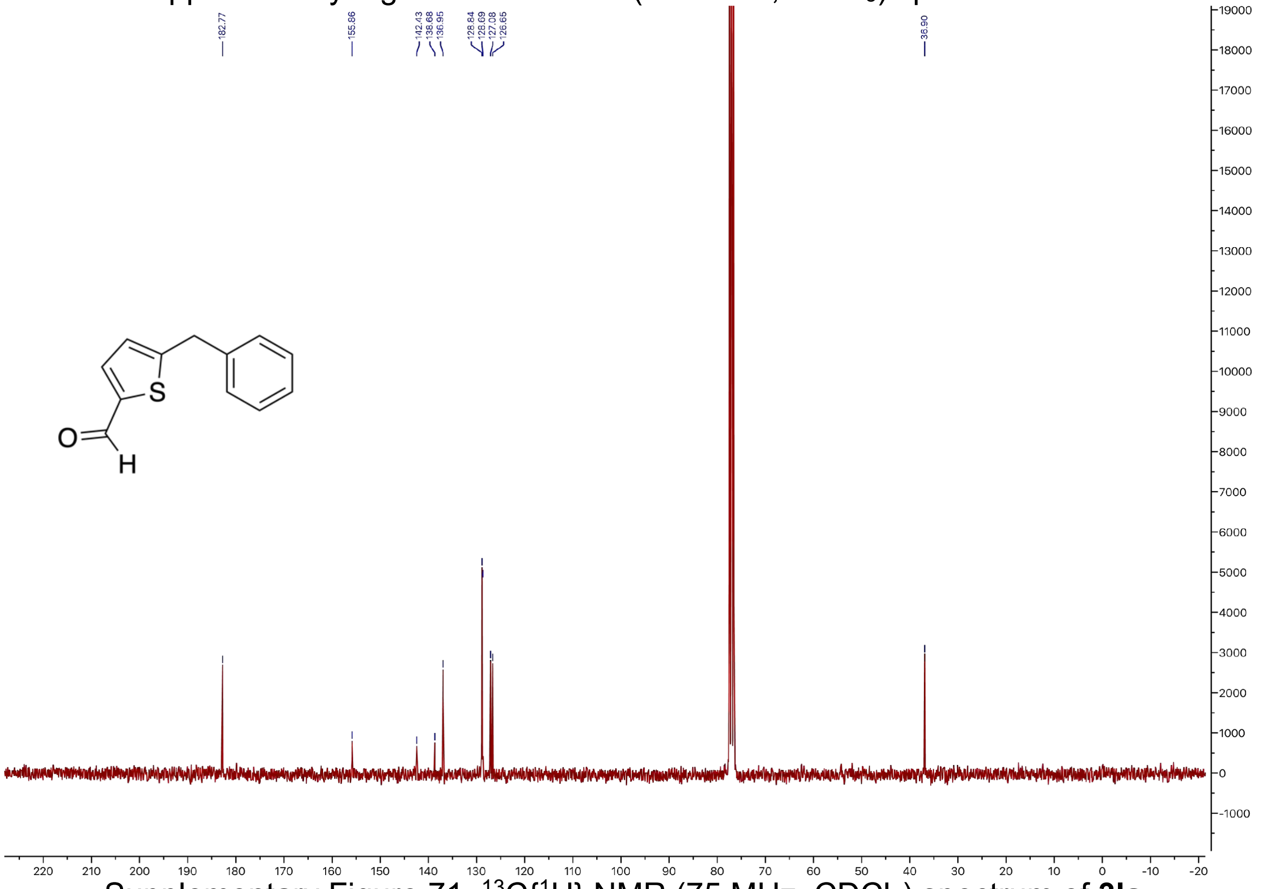

Supplementary Figure 71. $^{13}C\{^{1}H\}$ NMR (75 MHz, $CDCl_3$) spectrum of **3la**.

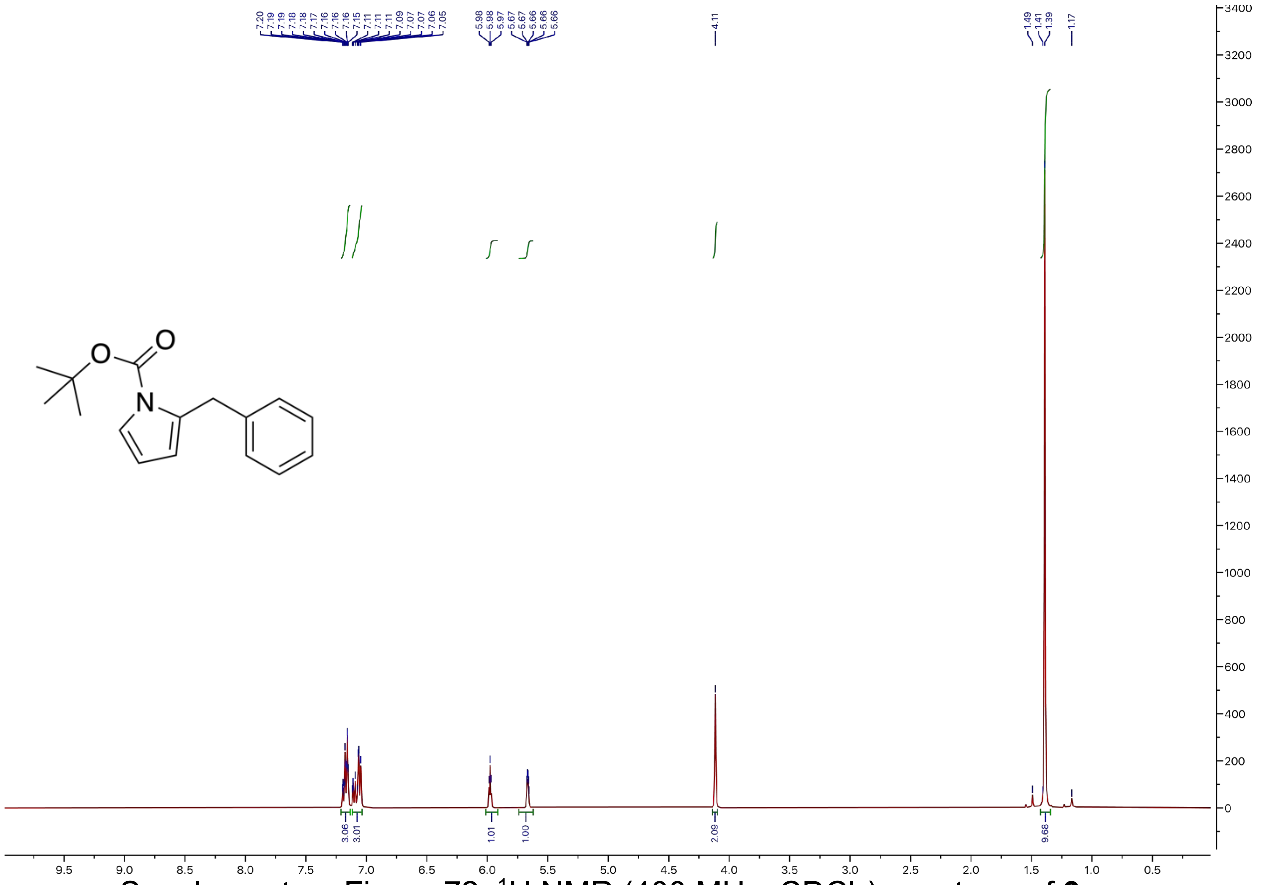

Supplementary Figure 72. ^{1}H NMR (400 MHz, $CDCl_3$) spectrum of **3ma**.

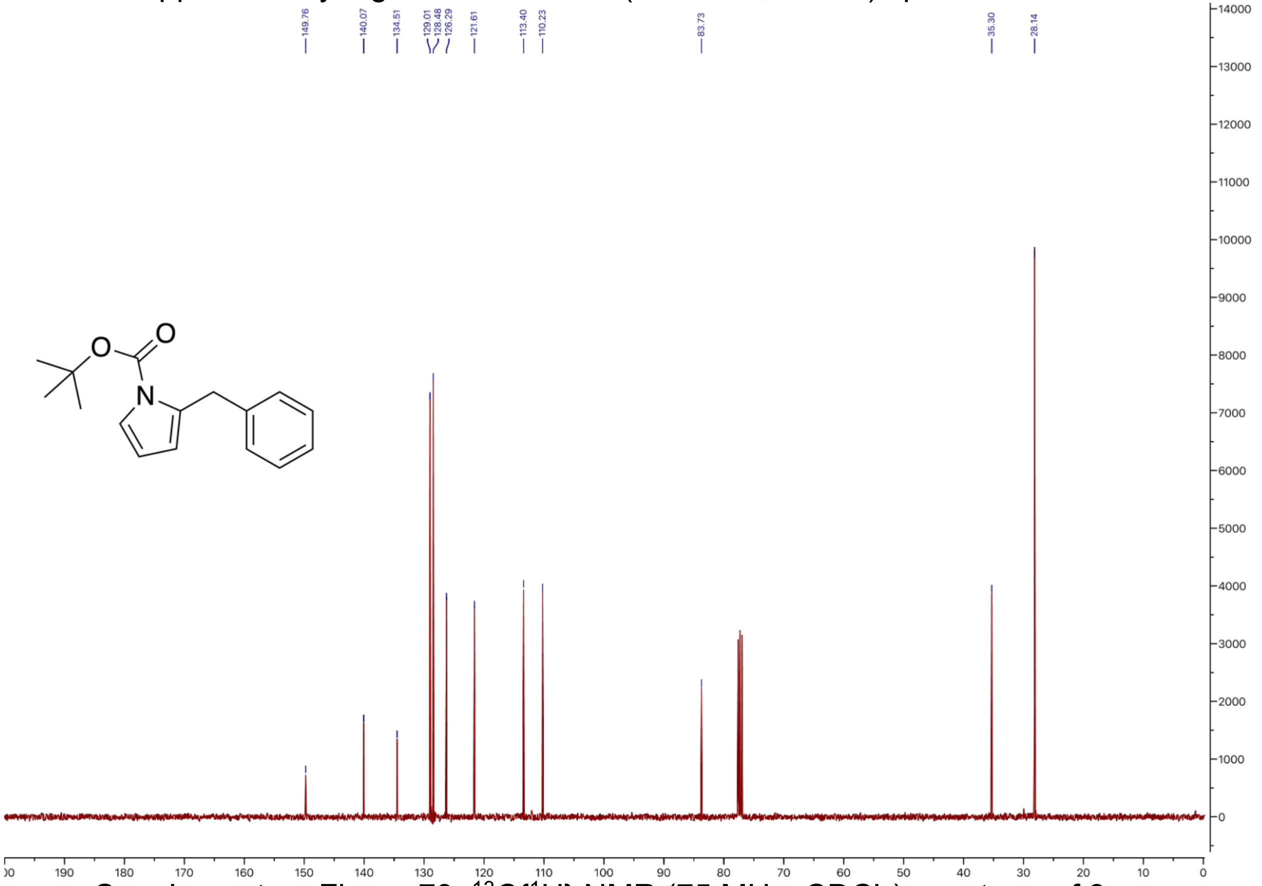

Supplementary Figure 73. $^{13}C\{^{1}H\}$ NMR (75 MHz, $CDCl_3$) spectrum of **3ma**.

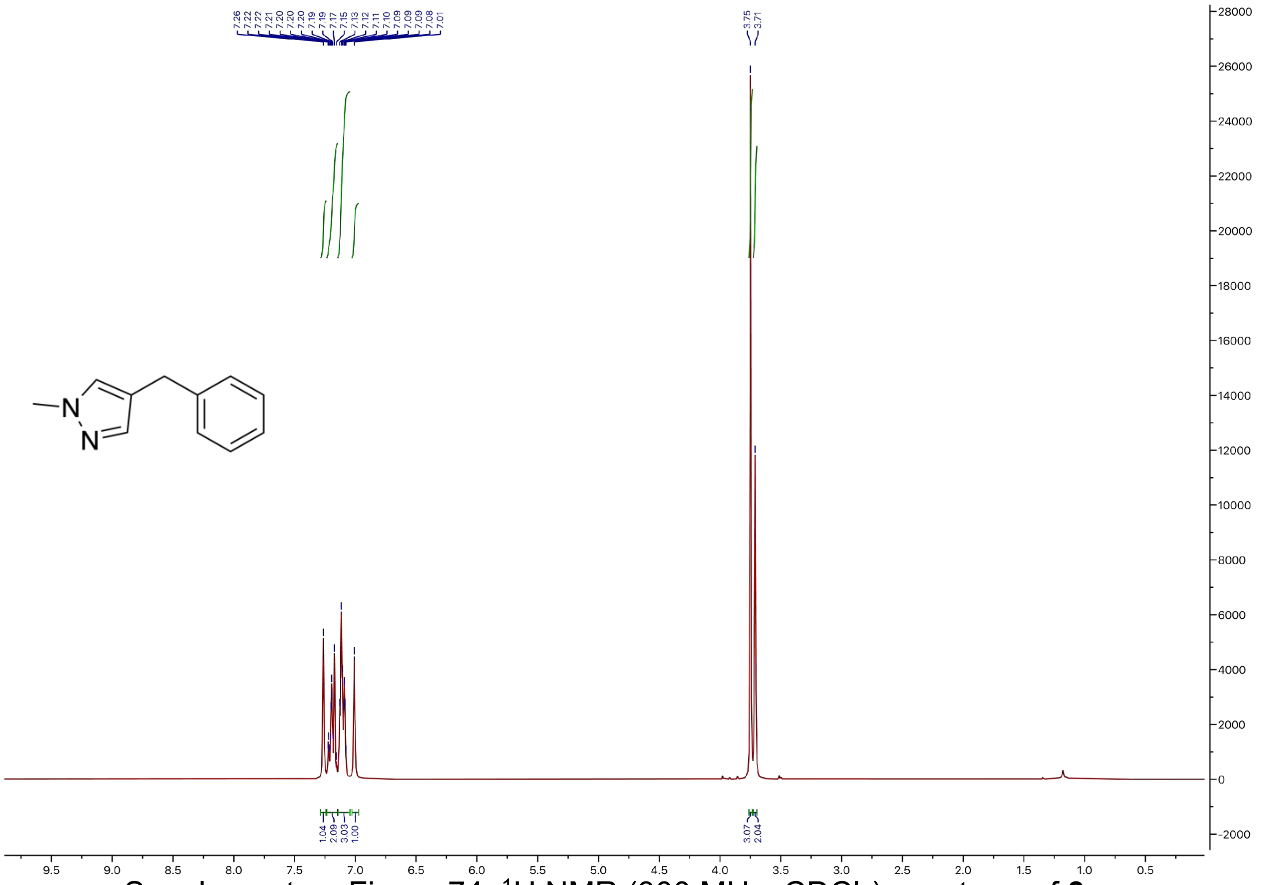

Supplementary Figure 74. ^1H NMR (300 MHz, $CDCl_3$) spectrum of **3na**.

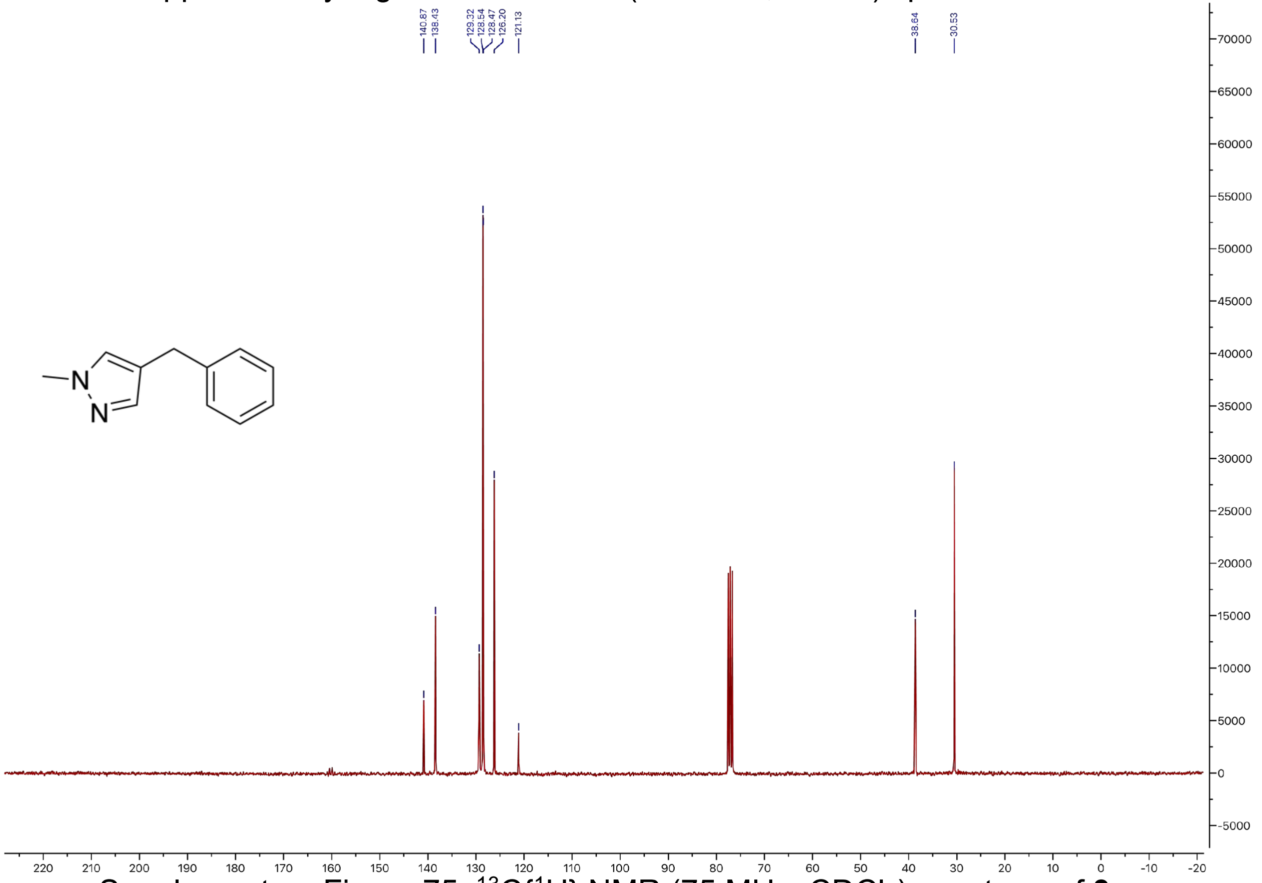

Supplementary Figure 75. $^{13}C\{^1H\}$ NMR (75 MHz, $CDCl_3$) spectrum of **3na**.

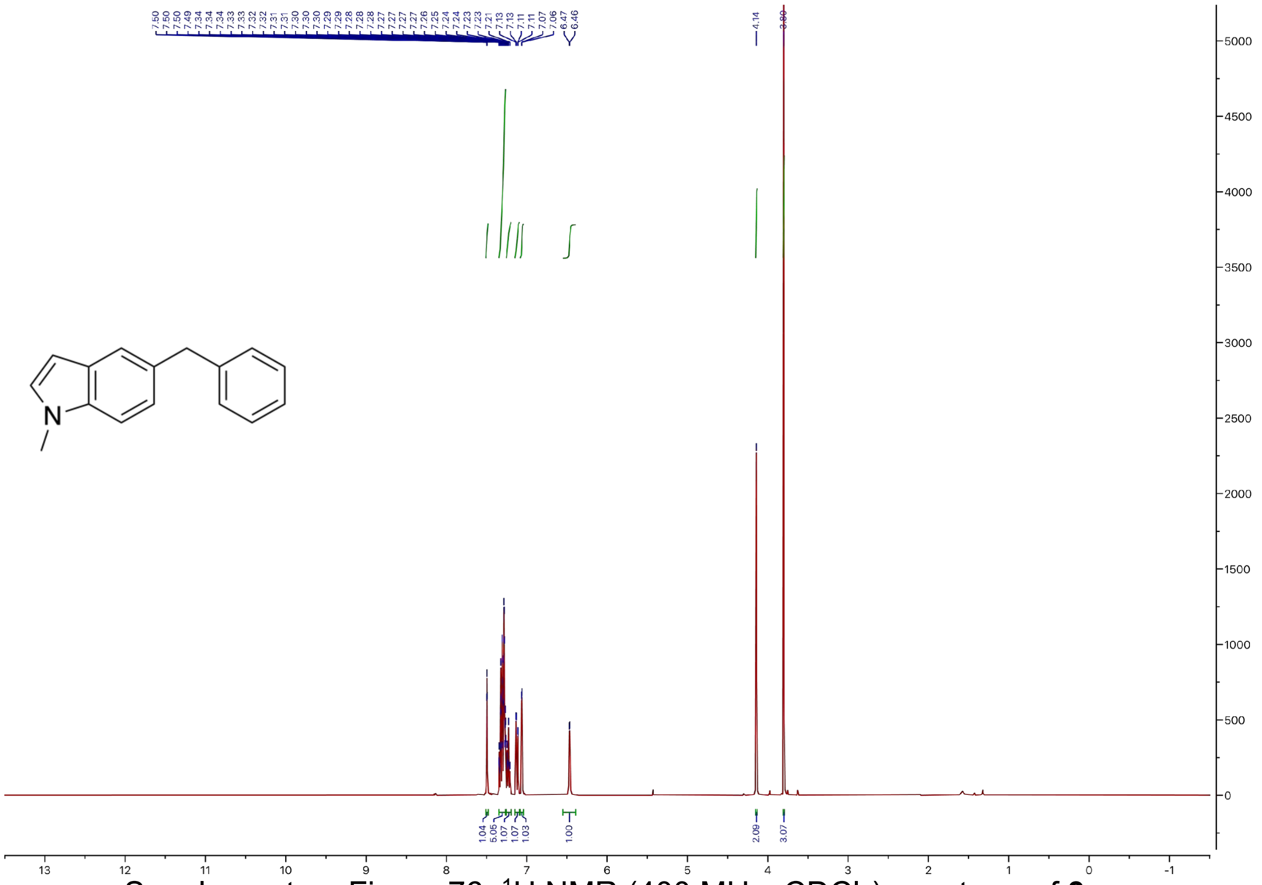

Supplementary Figure 76. ^{1}H NMR (400 MHz, $CDCl_3$) spectrum of **3oa**.

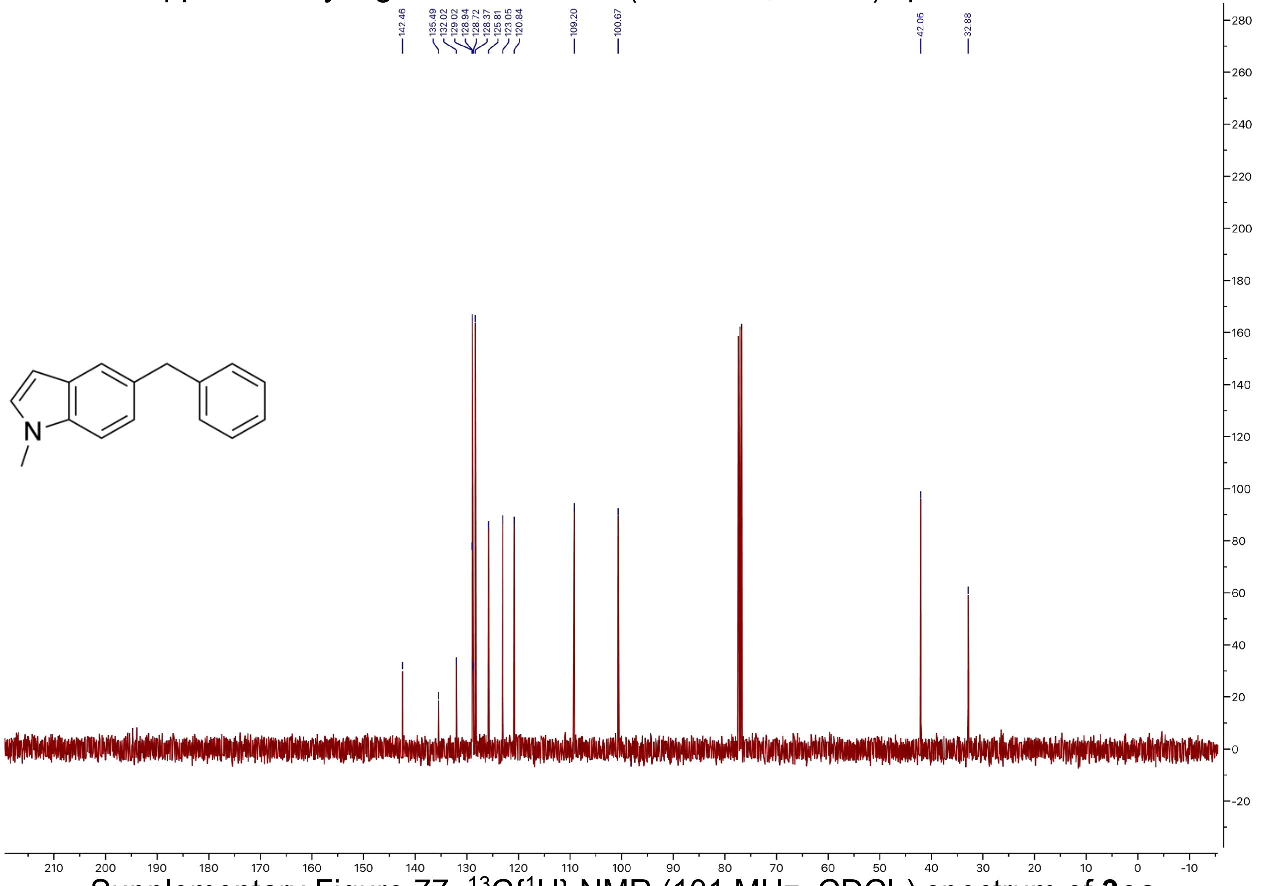

Supplementary Figure 77. $^{13}C\{^1H\}$ NMR (101 MHz, $CDCl_3$) spectrum of **3oa**.

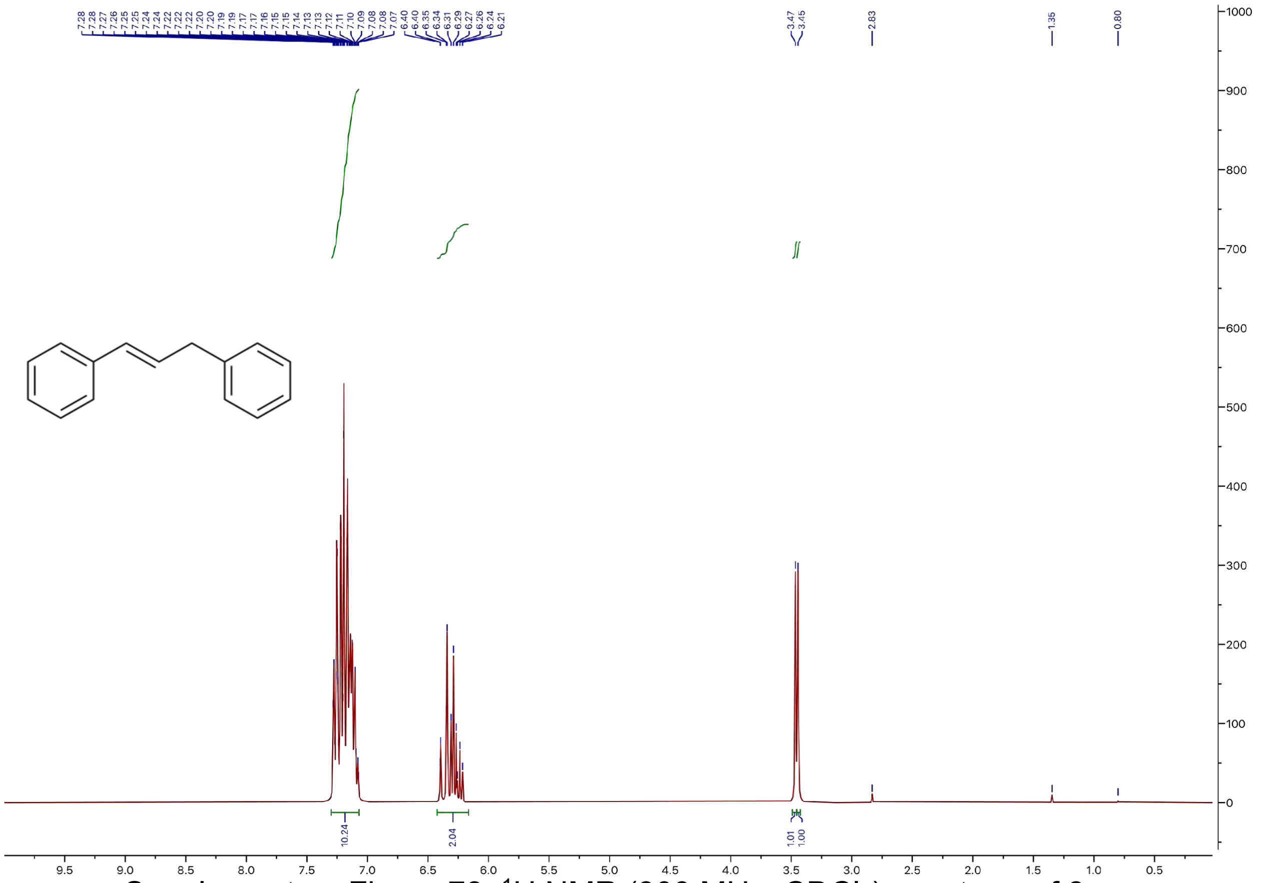

Supplementary Figure 78. ^{1}H NMR (300 MHz, $CDCl_3$) spectrum of **3pa**.

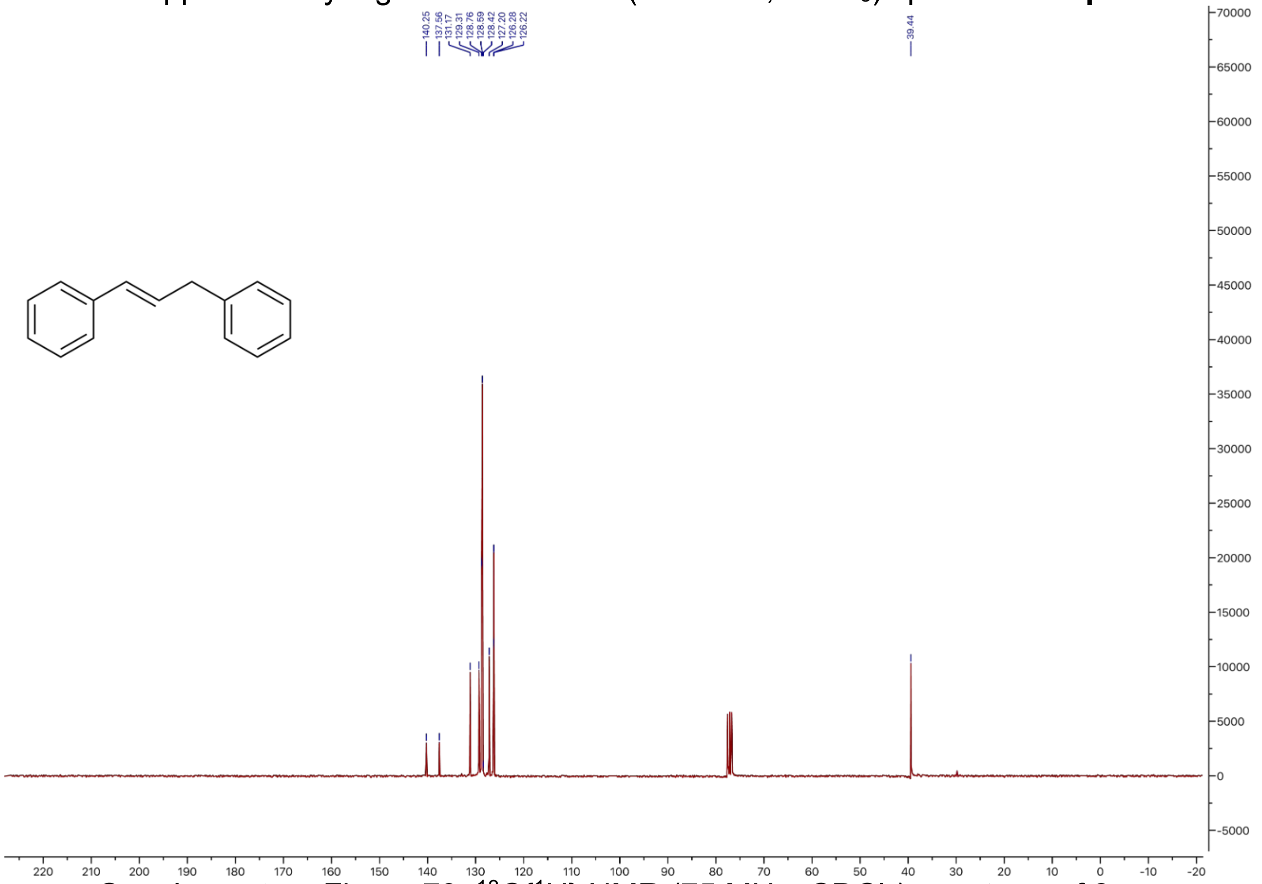

Supplementary Figure 79. $^{13}C\{^1H\}$ NMR (75 MHz, $CDCl_3$) spectrum of **3pa**.

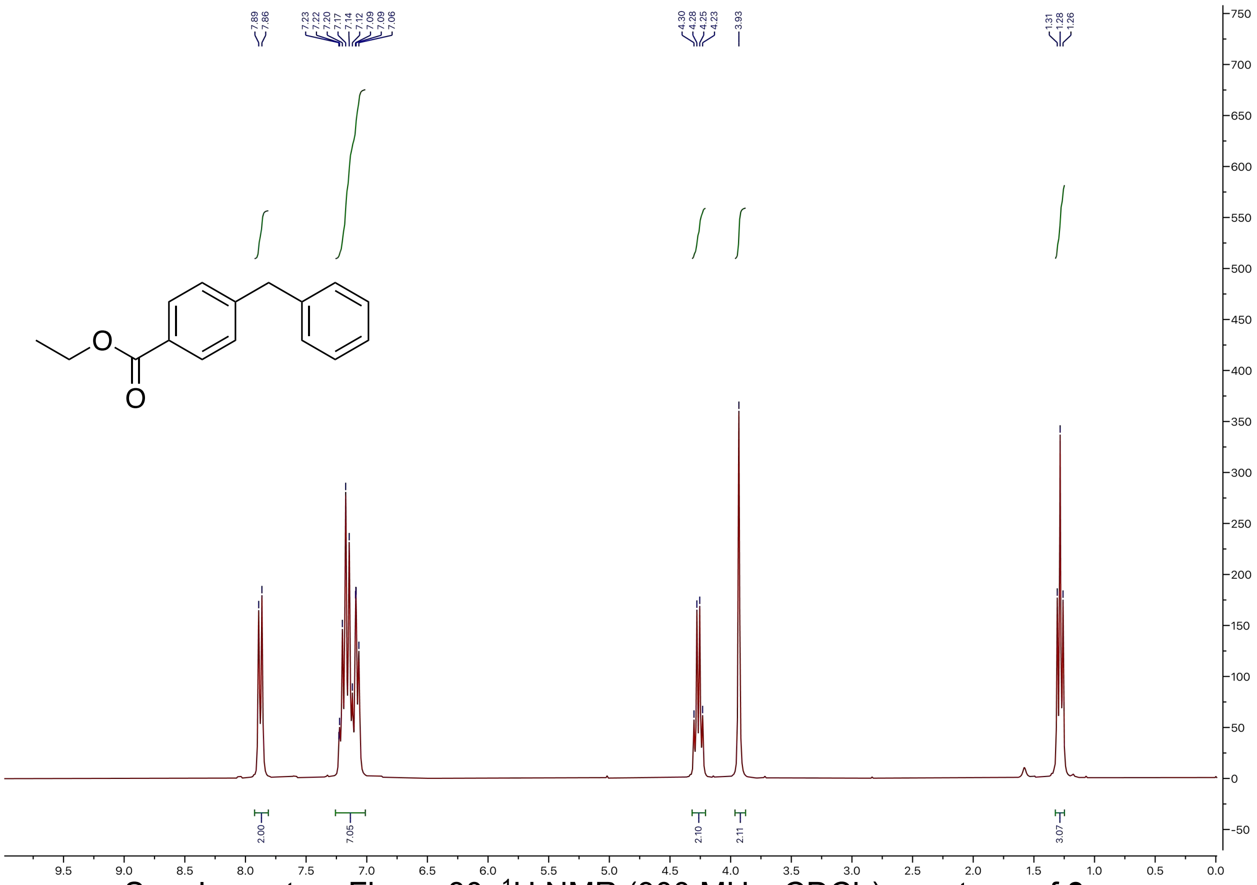

Supplementary Figure 80. ^{1}H NMR (300 MHz, $CDCl_3$) spectrum of **3qa**.

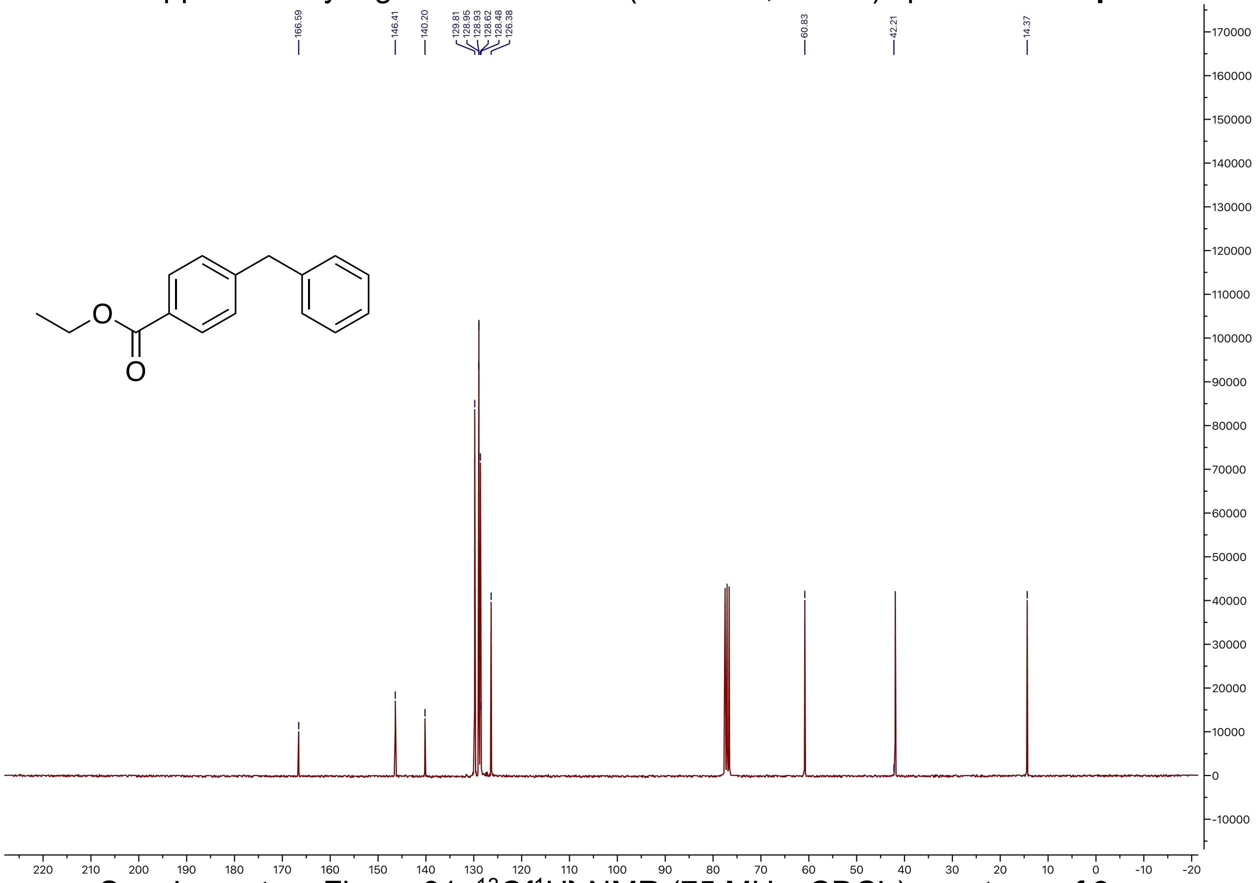

Supplementary Figure 81. $^{13}C\{^{1}H\}$ NMR (75 MHz, $CDCl_3$) spectrum of **3qa**.

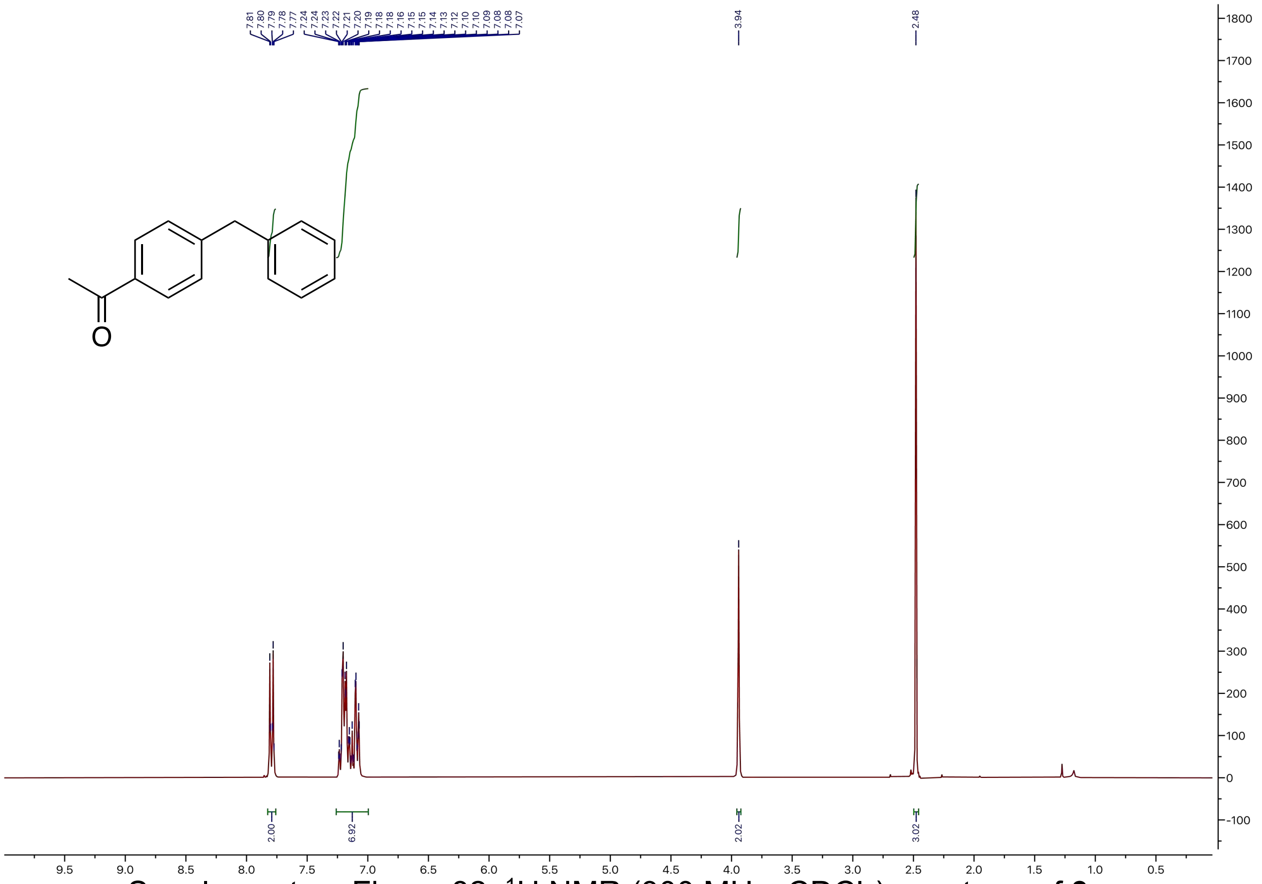

Supplementary Figure 82. ^{1}H NMR (300 MHz, $CDCl_3$) spectrum of **3ra**.

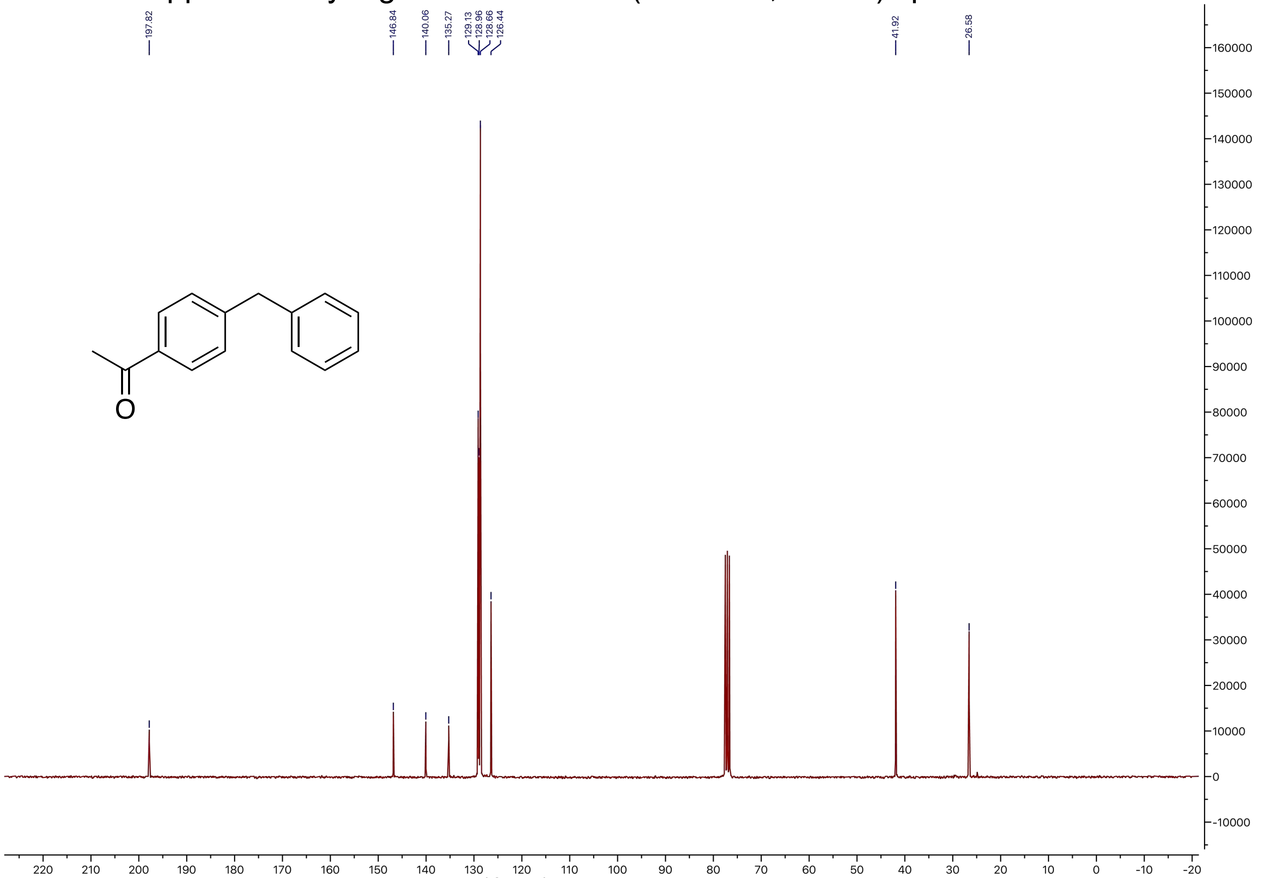

Supplementary Figure 83. $^{13}C\{^{1}H\}$ NMR (75 MHz, $CDCl_3$) spectrum of **3ra**.

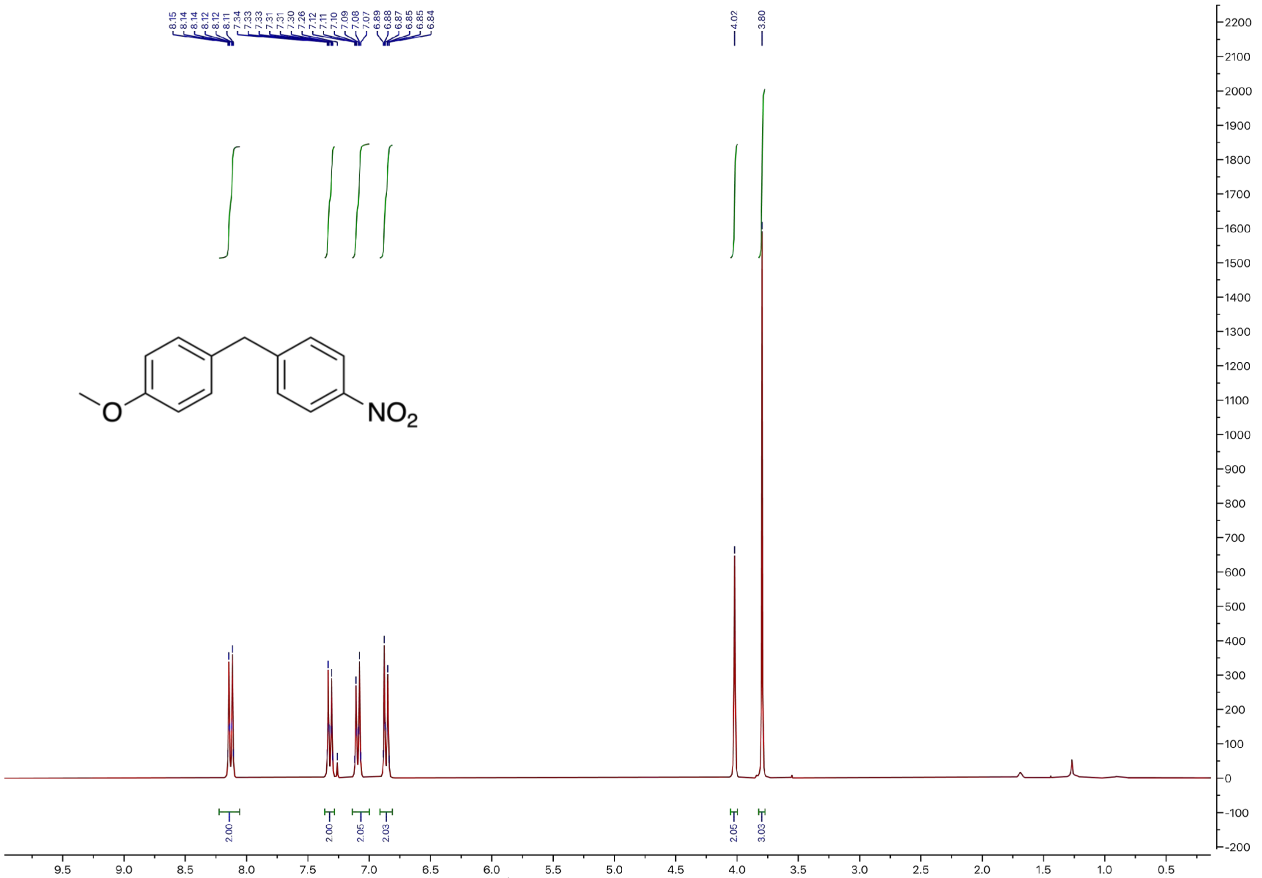

Supplementary Figure 84. ^{1}H NMR (300 MHz, $CDCl_3$) spectrum of **3ab**.

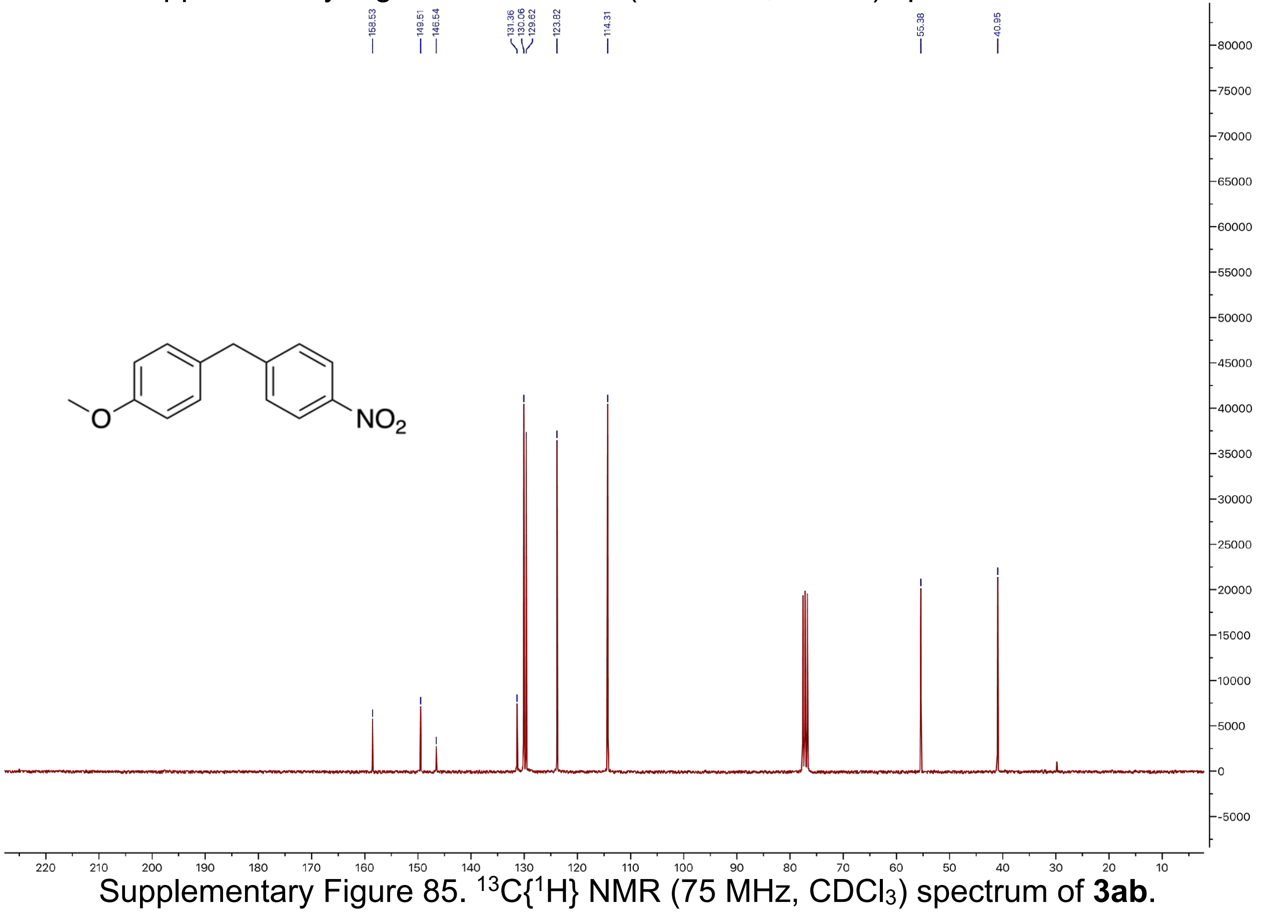

Supplementary Figure 85. $^{13}C\{^{1}H\}$ NMR (75 MHz, $CDCl_3$) spectrum of **3ab**.

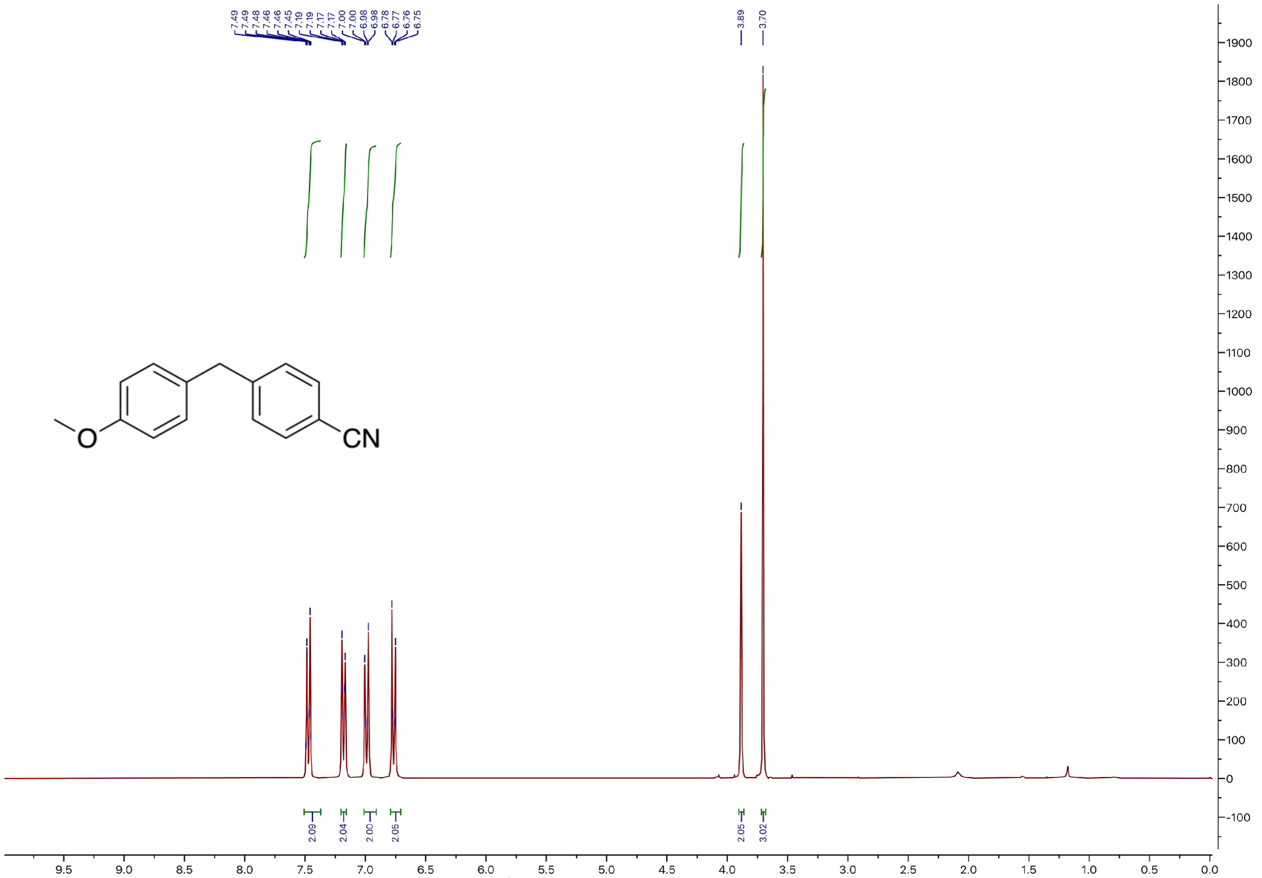

Supplementary Figure 86. ^{1}H NMR (300 MHz, $CDCl_3$) spectrum of **3ac**.

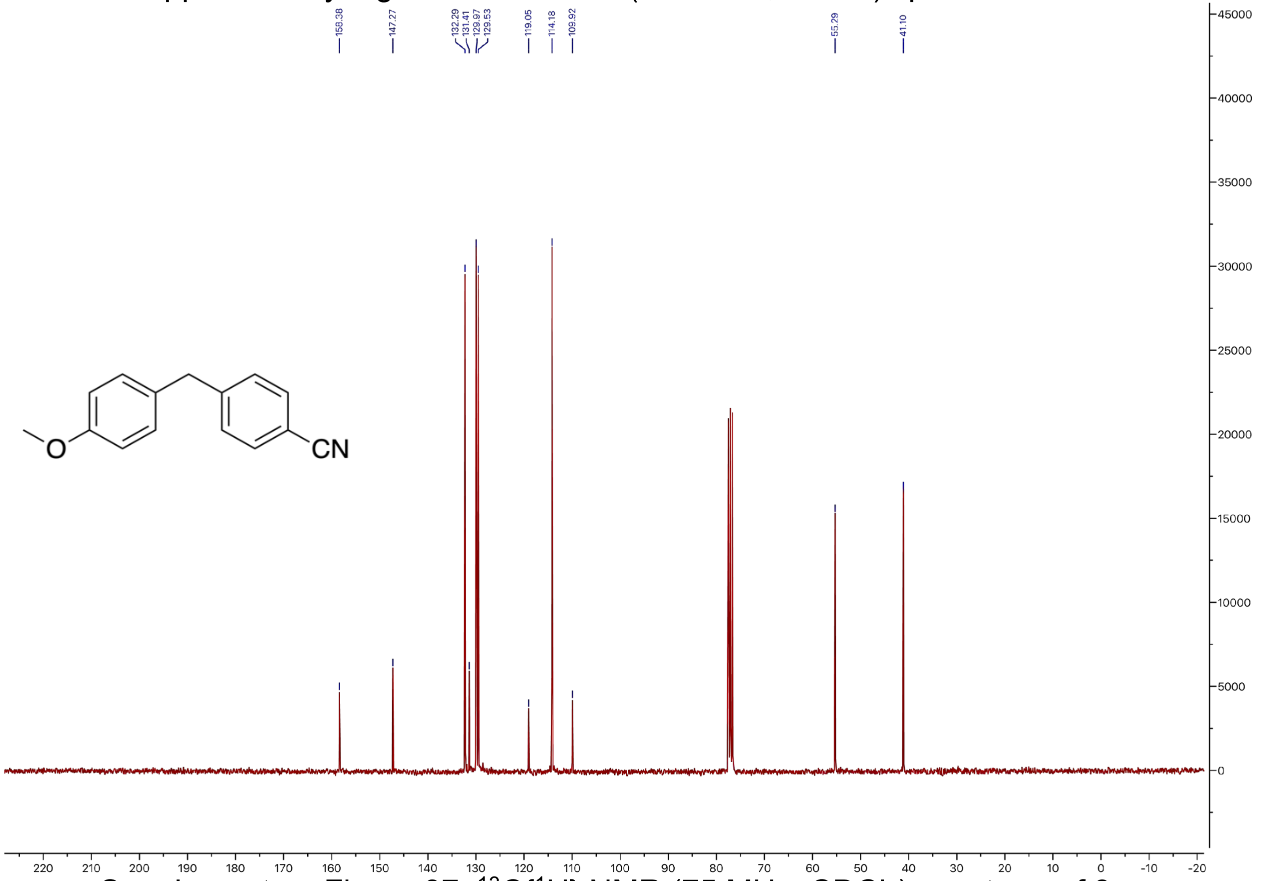

Supplementary Figure 87. $^{13}C\{^{1}H\}$ NMR (75 MHz, $CDCl_3$) spectrum of **3ac.**

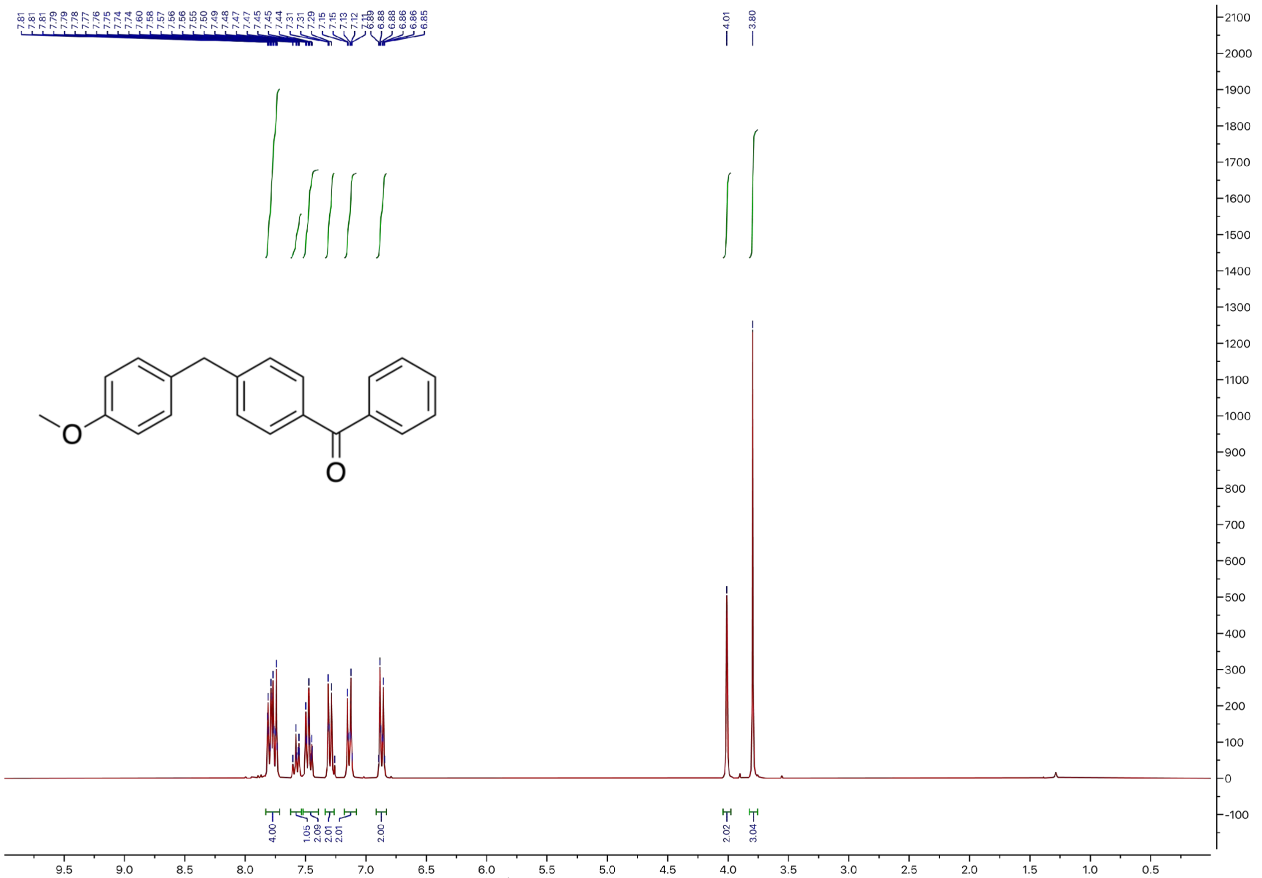

Supplementary Figure 88. ^{1}H NMR (300 MHz, $CDCl_3$) spectrum of **3ad**.

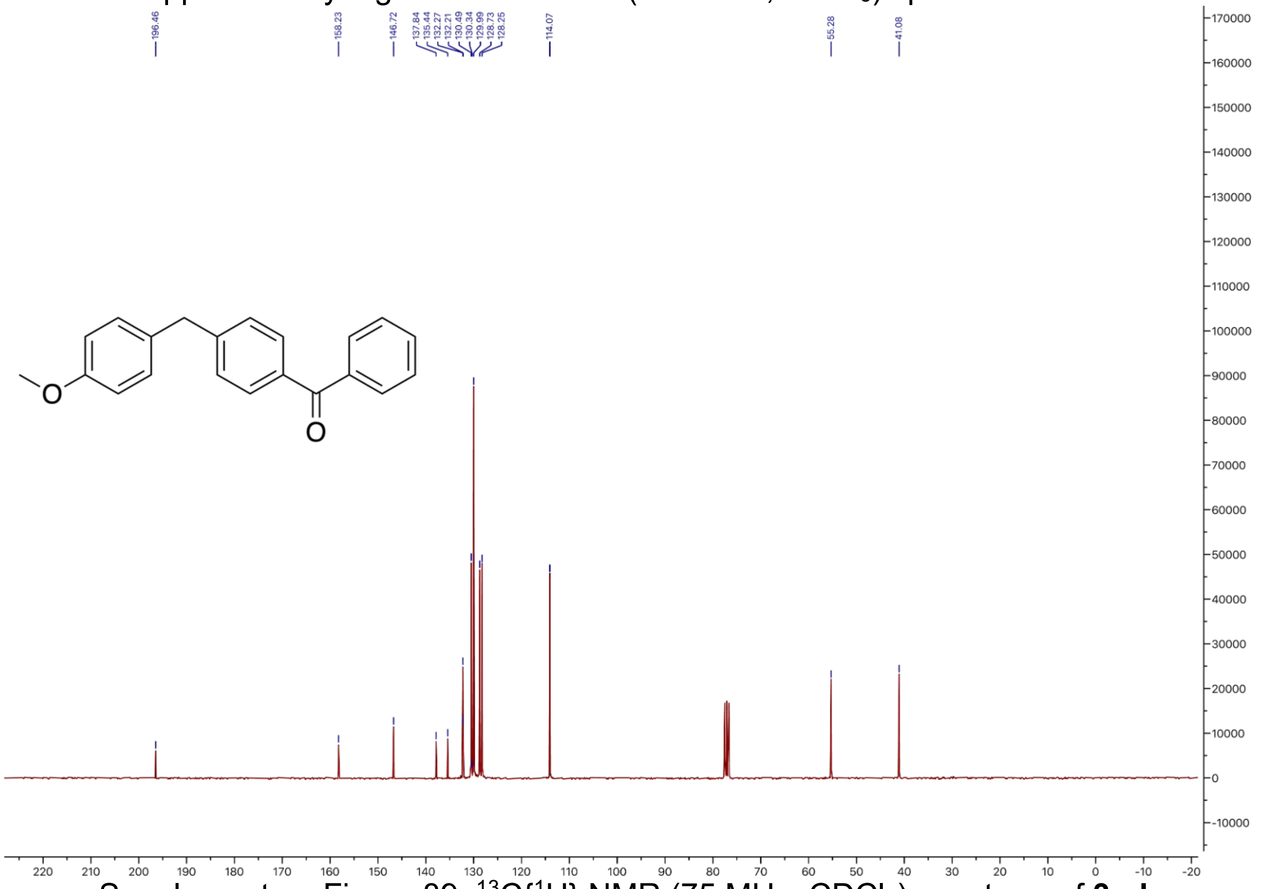

Supplementary Figure 89. $^{13}C\{^{1}H\}$ NMR (75 MHz, $CDCl_3$) spectrum of **3ad**.

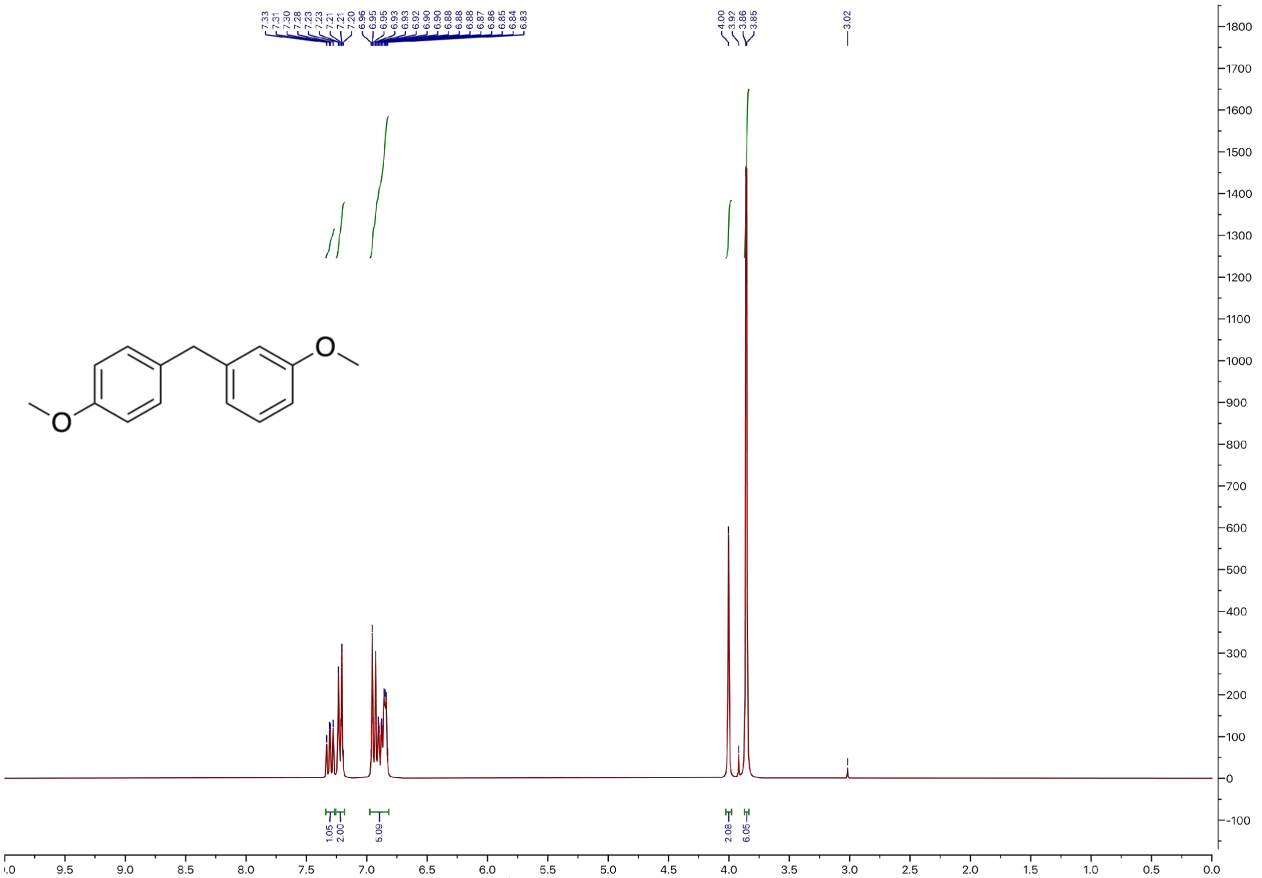

Supplementary Figure 90. ^{1}H NMR (300 MHz, $CDCl_3$) spectrum of **3ae**.

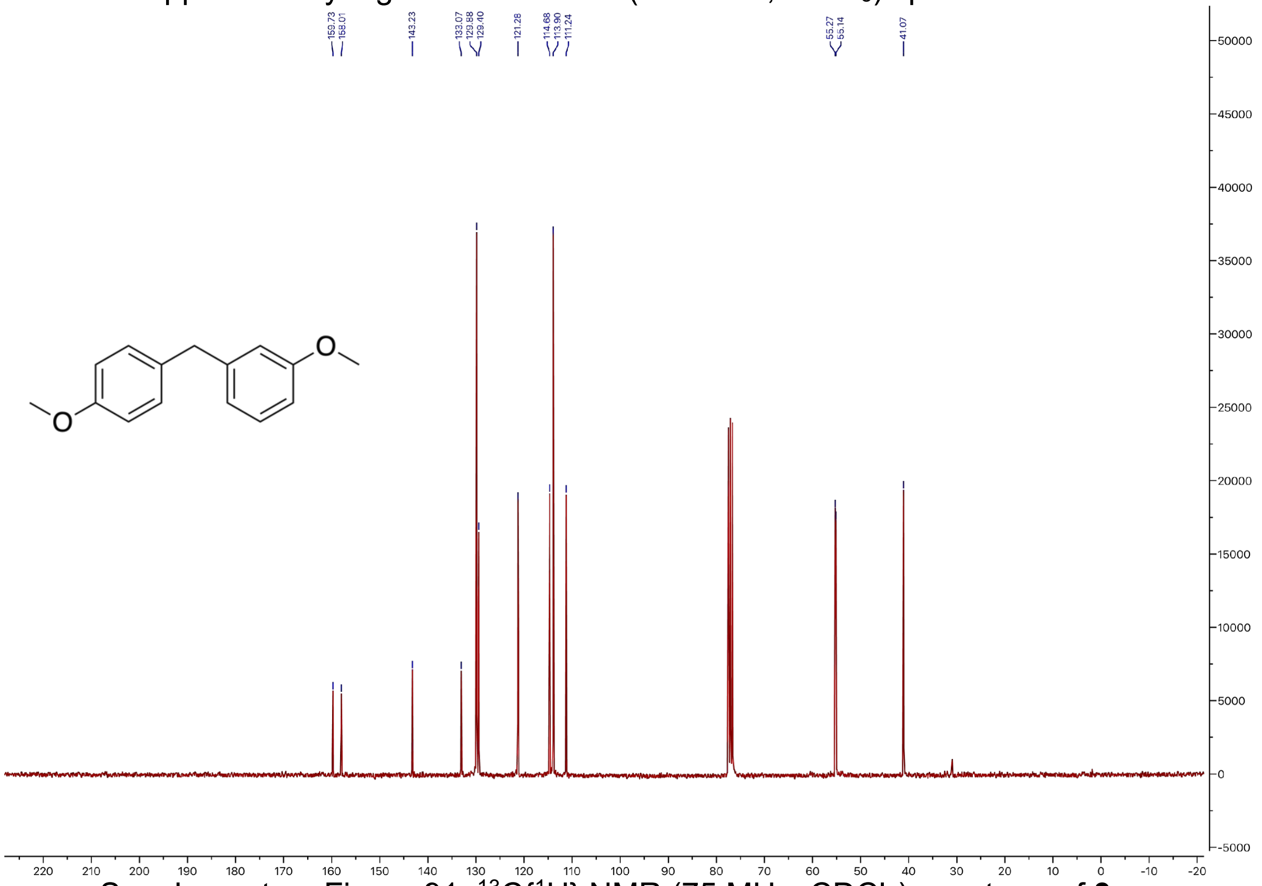

Supplementary Figure 91. $^{13}C\{^{1}H\}$ NMR (75 MHz, $CDCl_3$) spectrum of **3ae**.

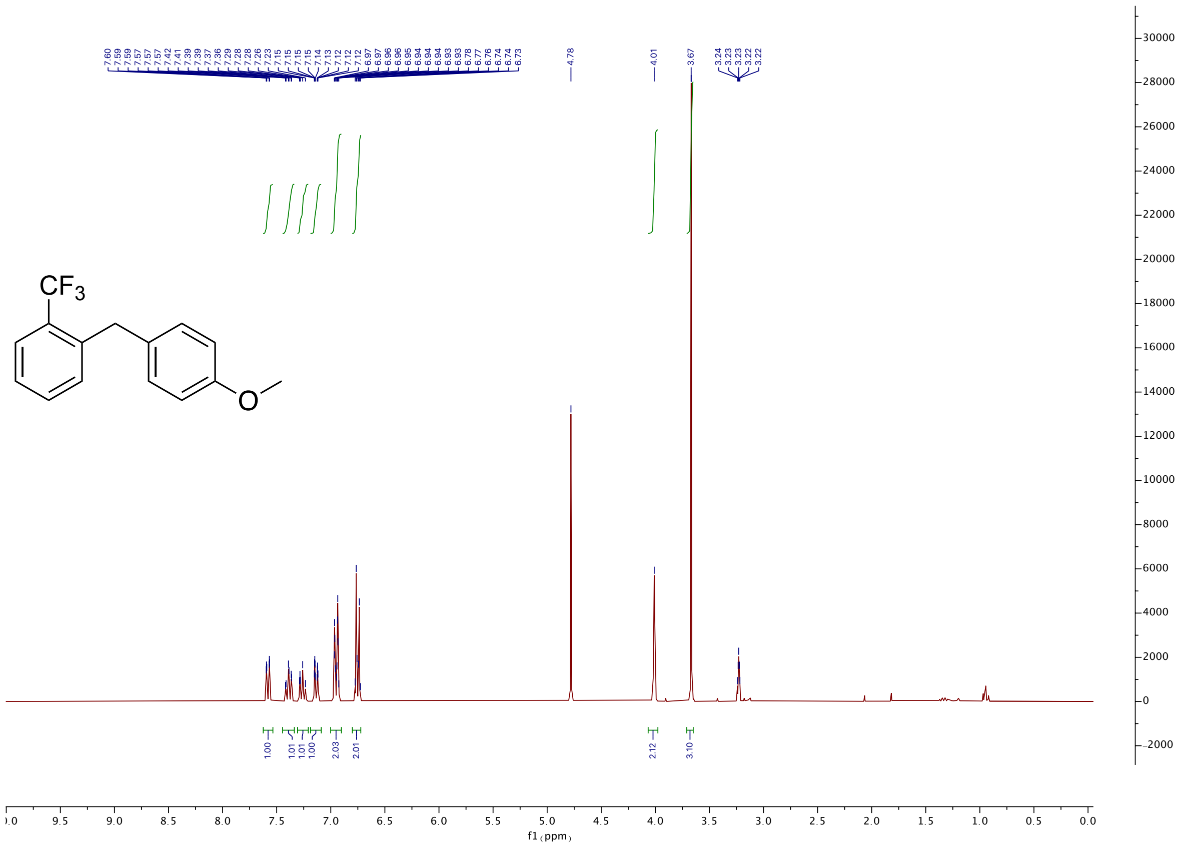

Supplementary Figure 92. ^{1}H NMR (300 MHz, MeOD) spectrum of **3af**.

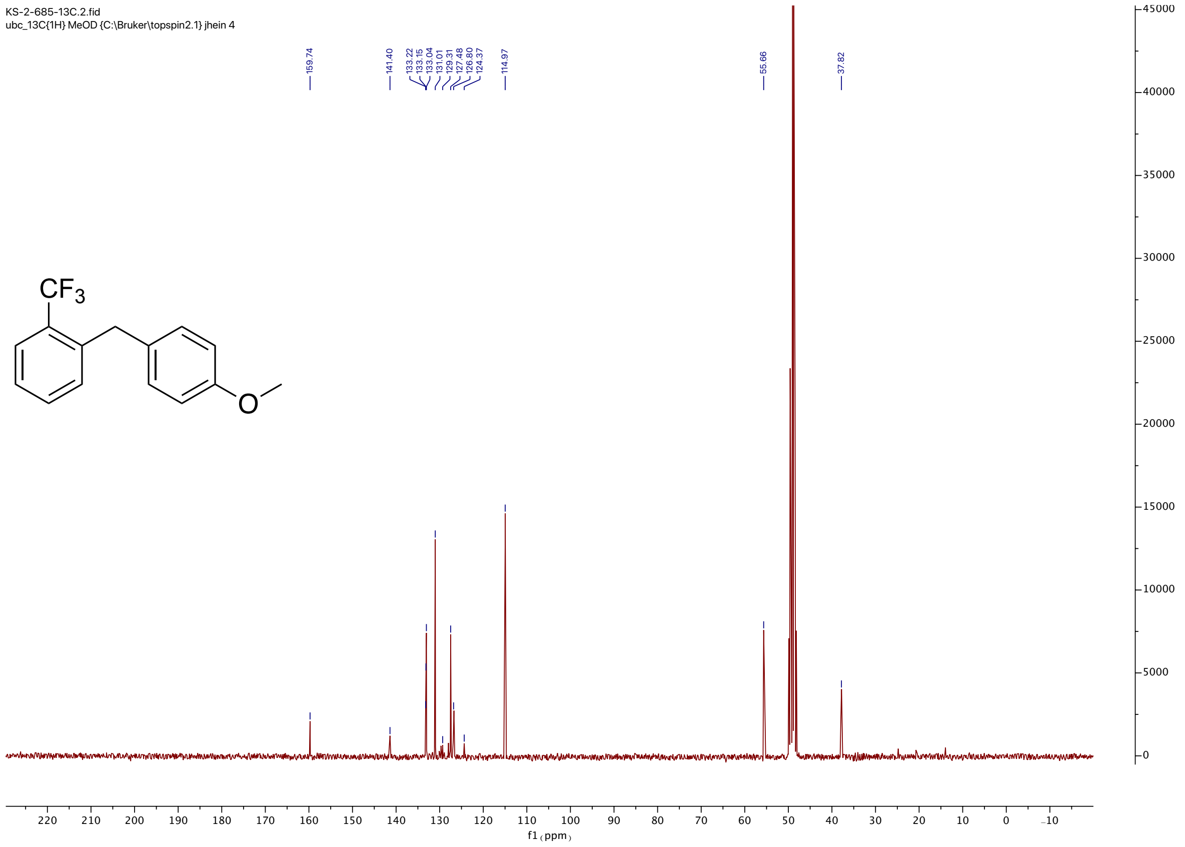

Supplementary Figure 93. $^{13}C\{^{1}H\}$ NMR (75 MHz, MeOD) spectrum of **3af**.

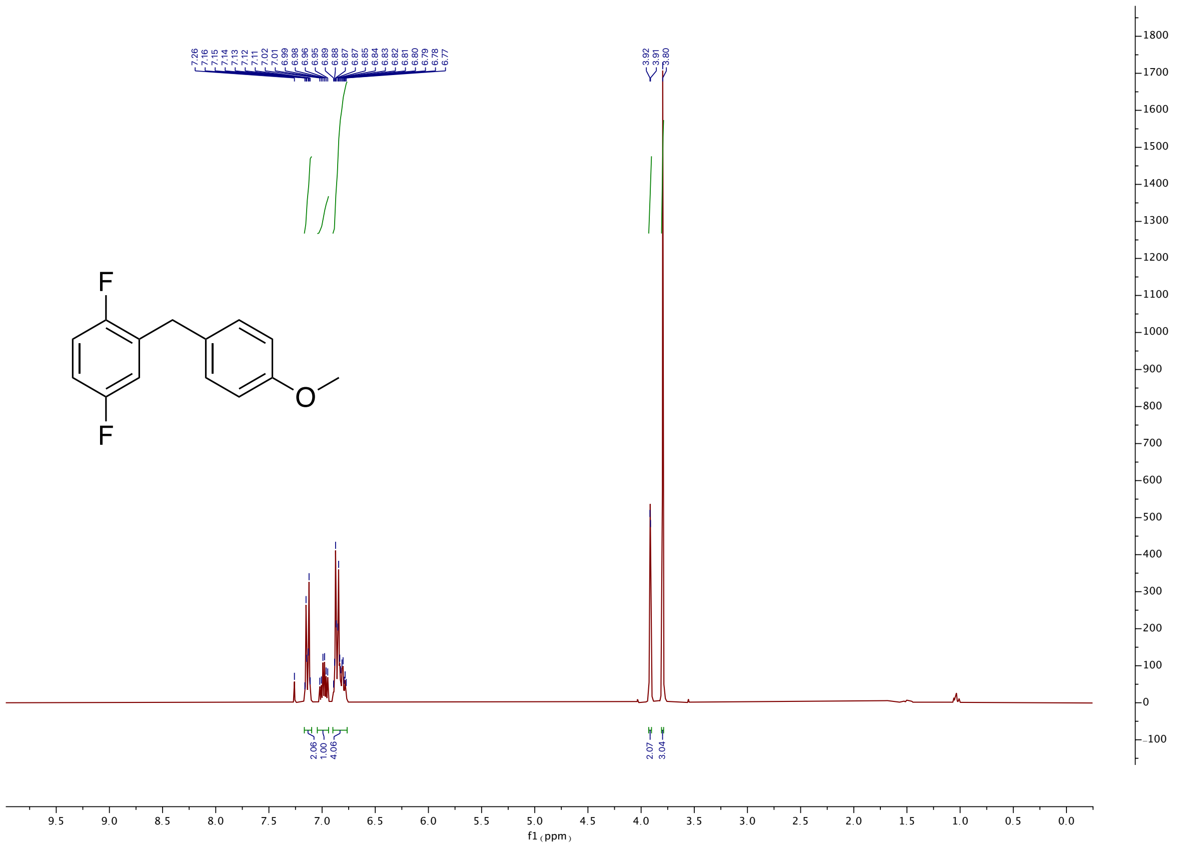

Supplementary Figure 94. ^{1}H NMR (300 MHz, $CDCl_3$) spectrum of **3ag**.

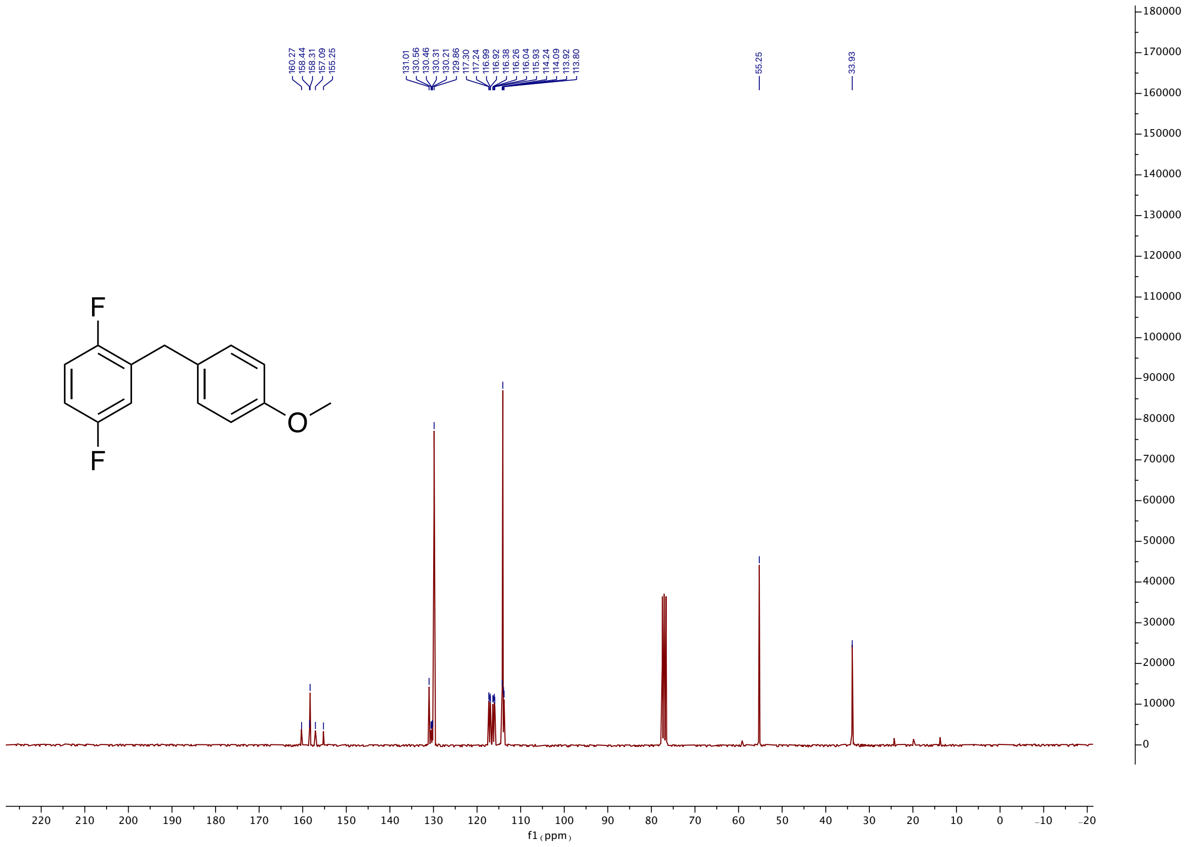

Supplementary Figure 95. $^{13}C\{^{1}H\}$ NMR (75 MHz, $CDCl_3$) spectrum of **3ag.**

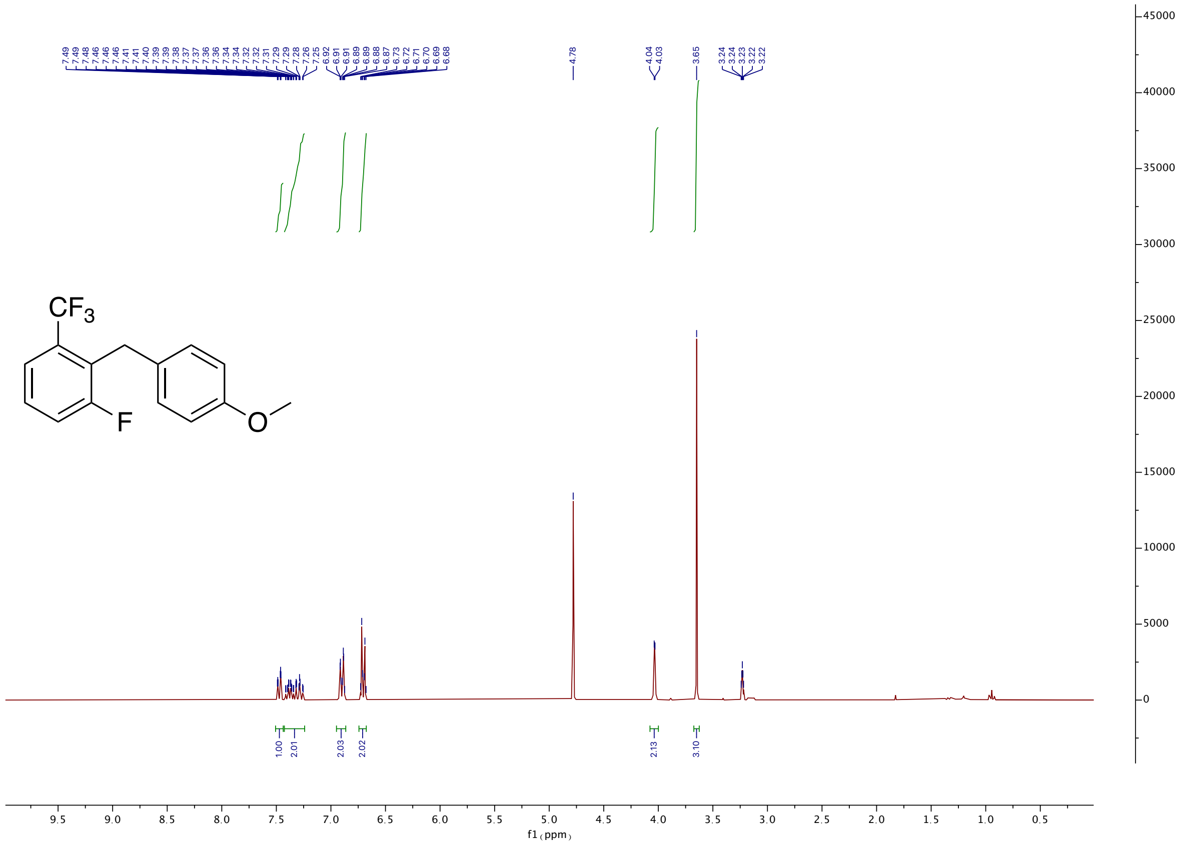

Supplementary Figure 96. ^{1}H NMR (300 MHz, MeOD) spectrum of **3ah**.

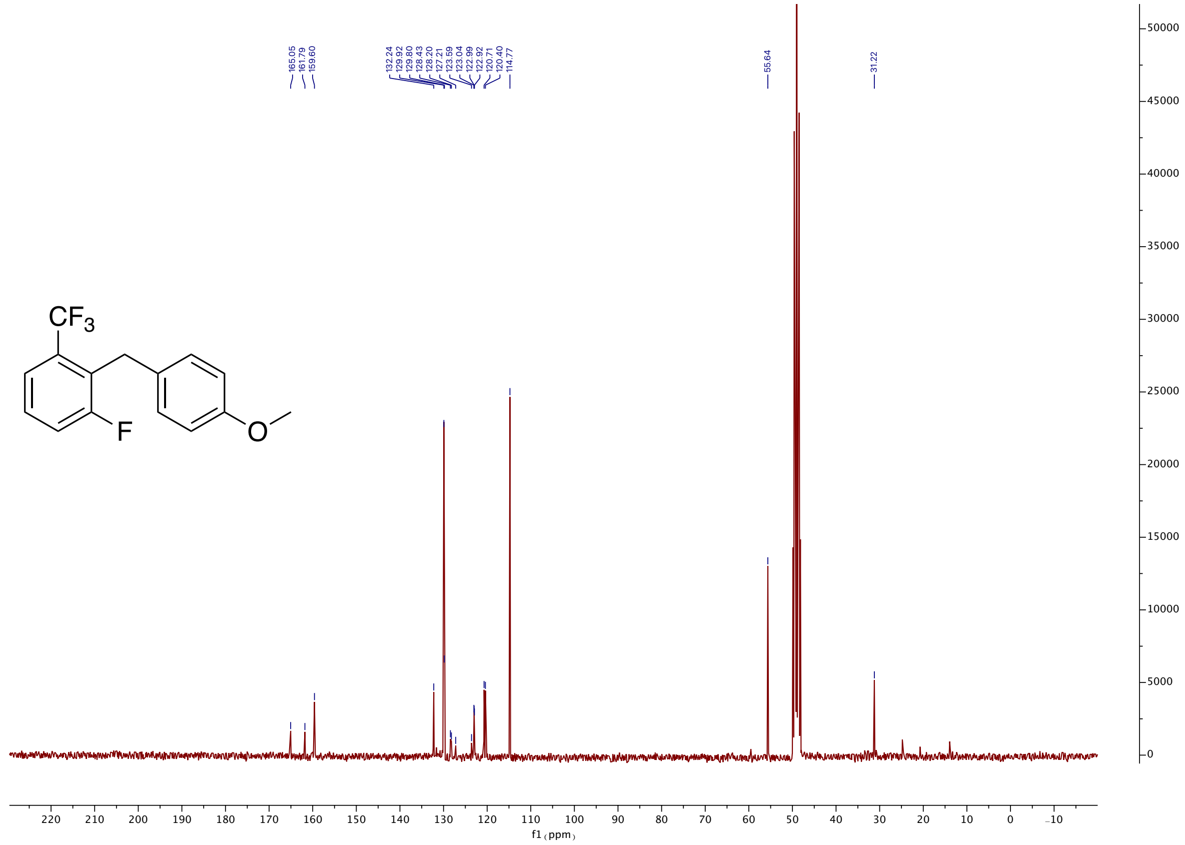

Supplementary Figure 97. $^{13}C\{^{1}H\}$ NMR (75 MHz, MeOD) spectrum of **3ah**.

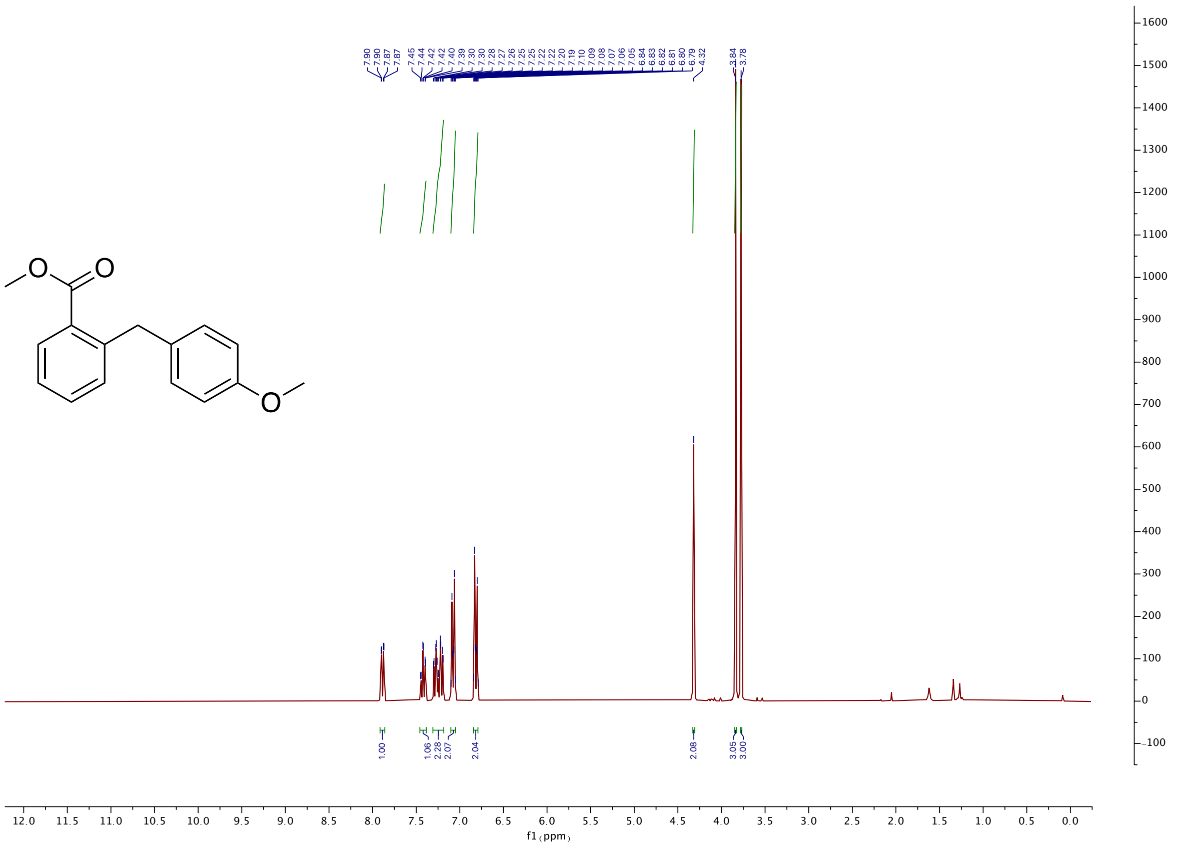

Supplementary Figure 98. ^{1}H NMR (300 MHz, $CDCl_3$) spectrum of **3ai**.

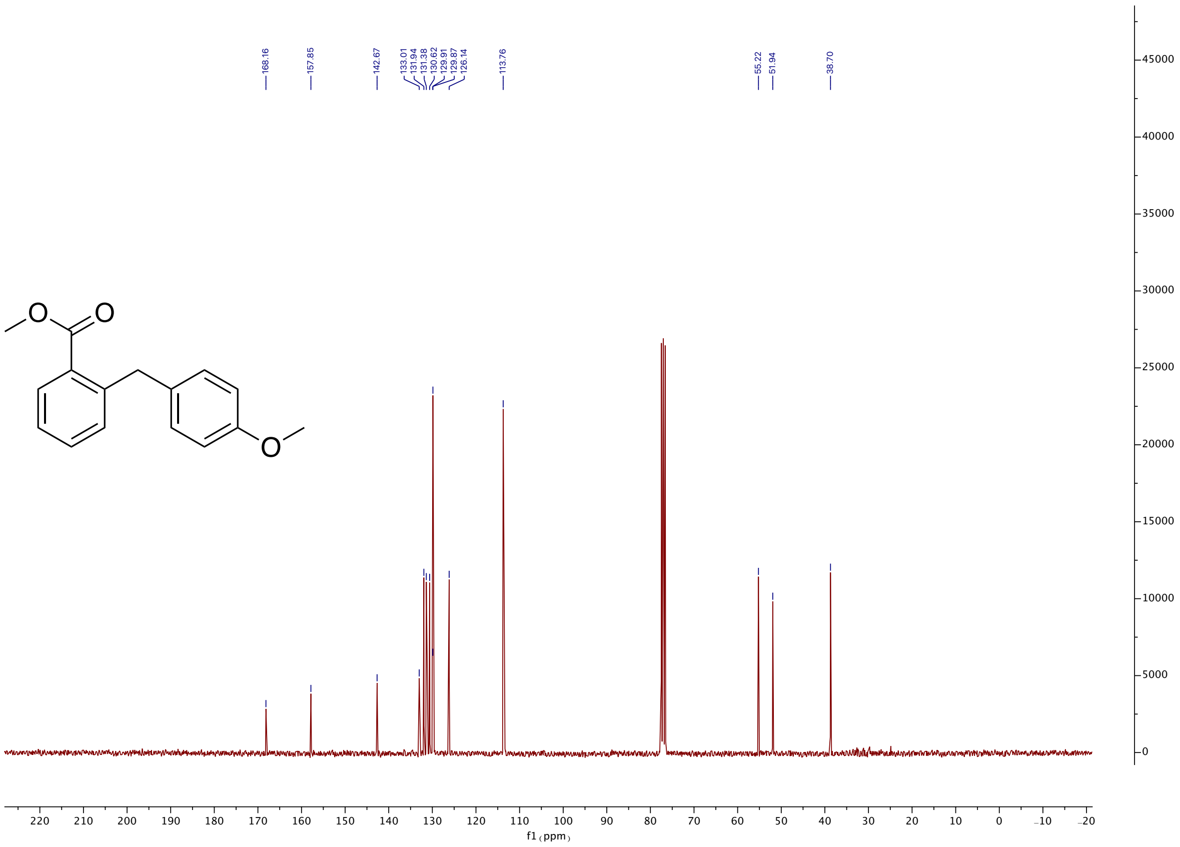

Supplementary Figure 99. $^{13}C\{^{1}H\}$ NMR (75 MHz, $CDCl_3$) spectrum of **3ai**.

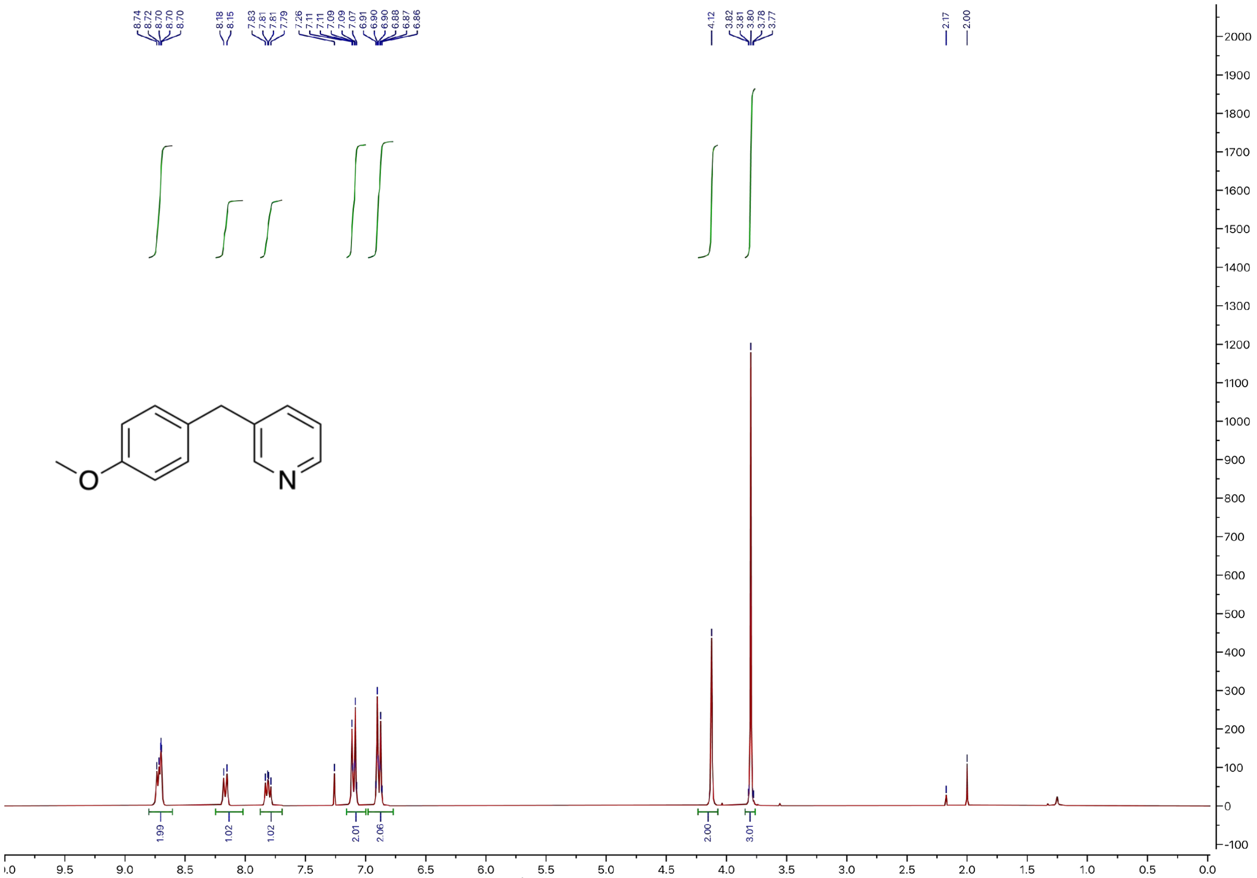

Supplementary Figure 100. ^{1}H NMR (300 MHz, $CDCl_3$) spectrum of **3aj**.

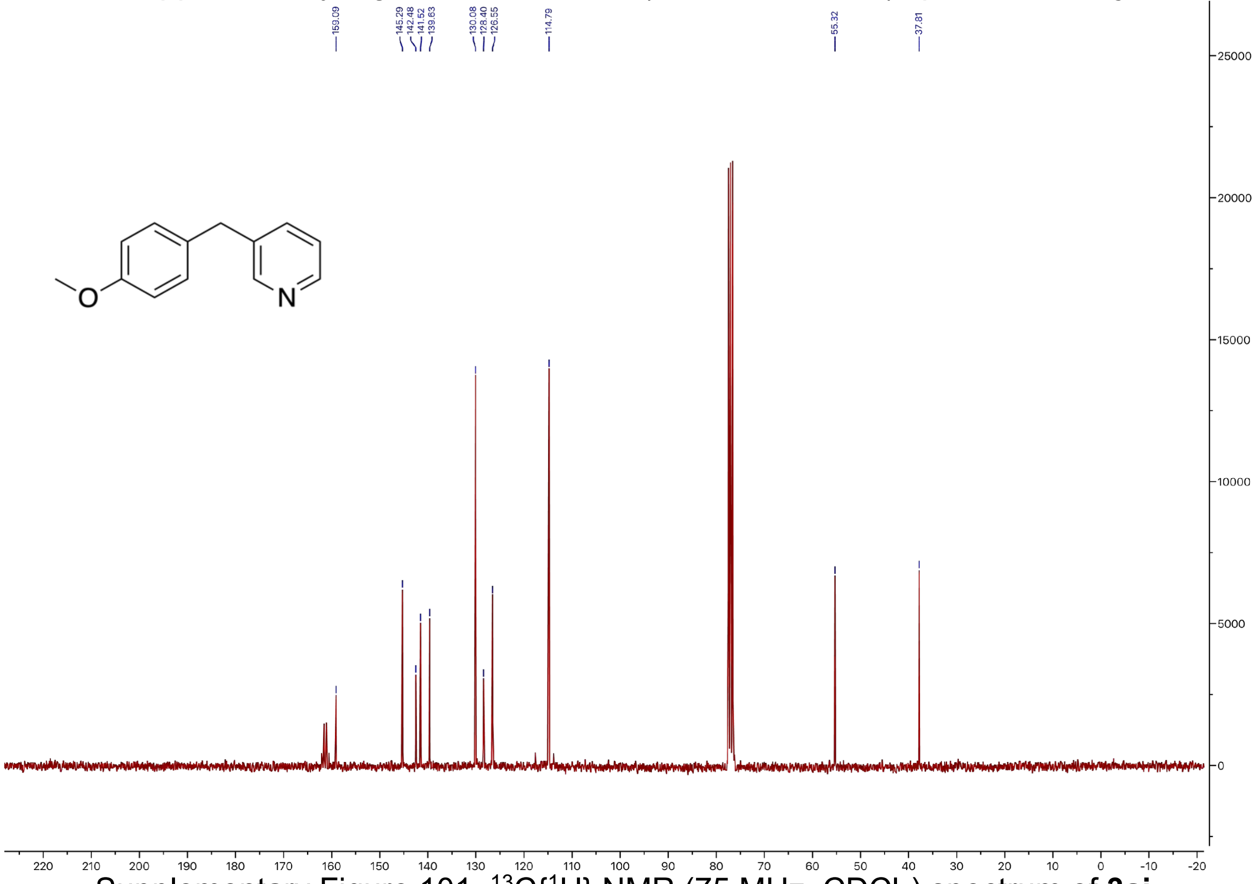

Supplementary Figure 101. $^{13}C\{^{1}H\}$ NMR (75 MHz, $CDCl_3$) spectrum of **3aj**.

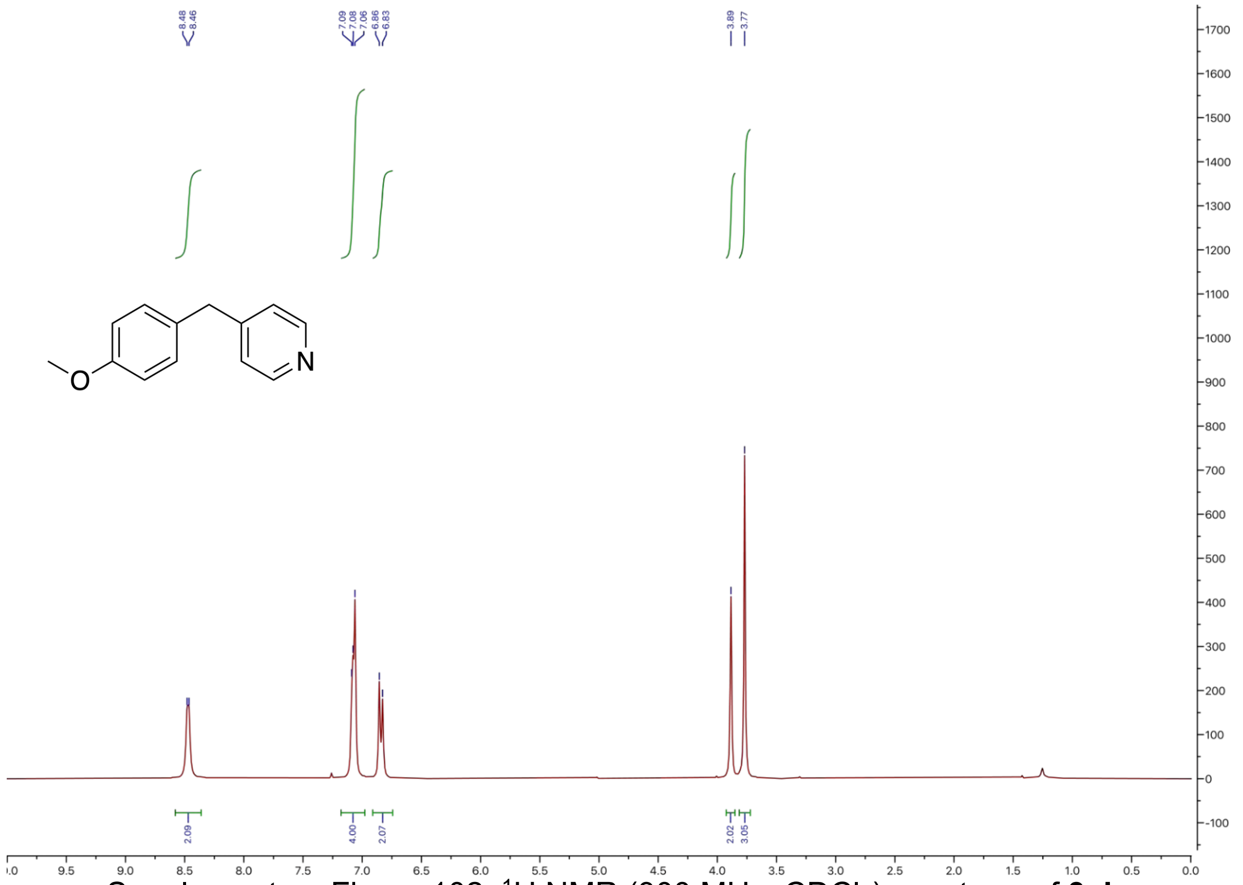

Supplementary Figure 102. ^{1}H NMR (300 MHz, $CDCl_3$) spectrum of **3ak**.

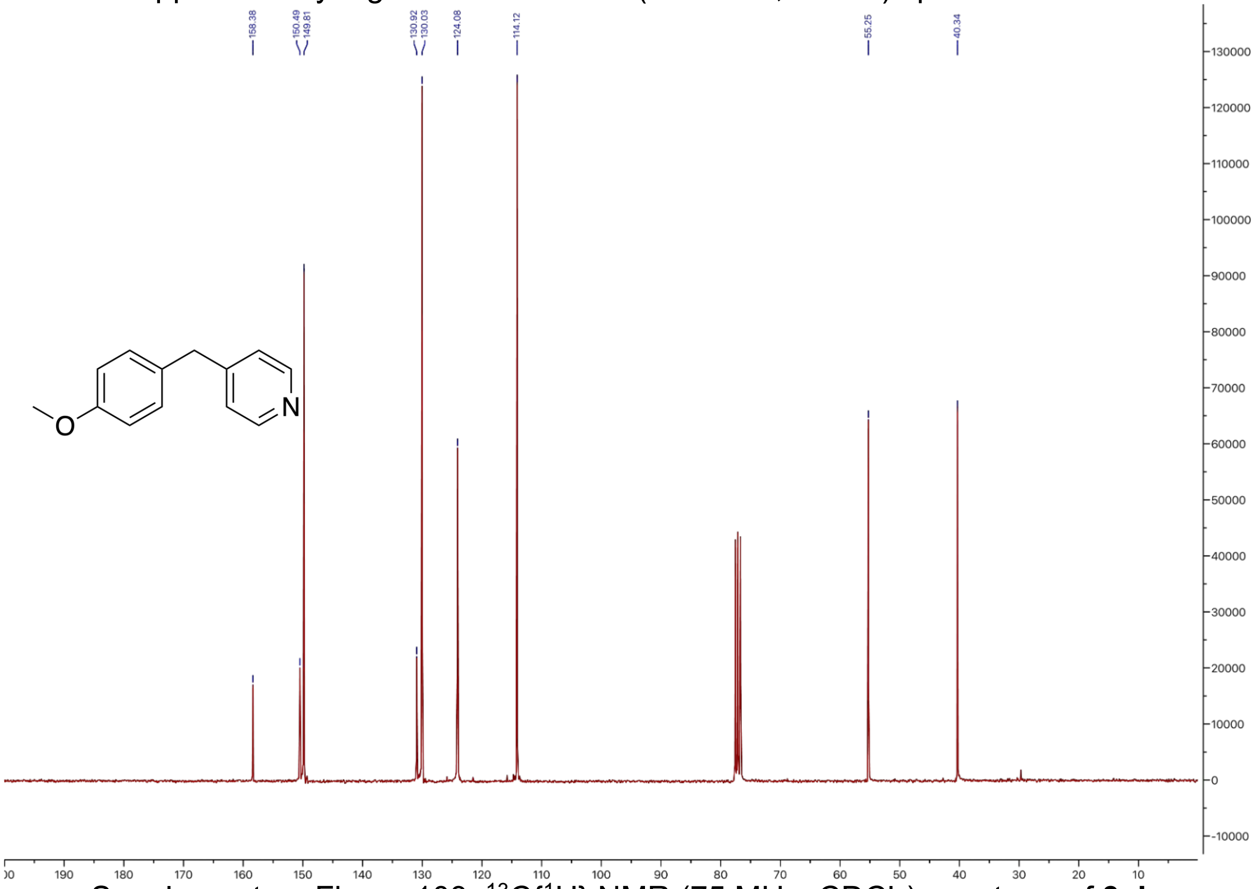

Supplementary Figure 103. $^{13}C\{^{1}H\}$ NMR (75 MHz, $CDCl_3$) spectrum of **3ak**.

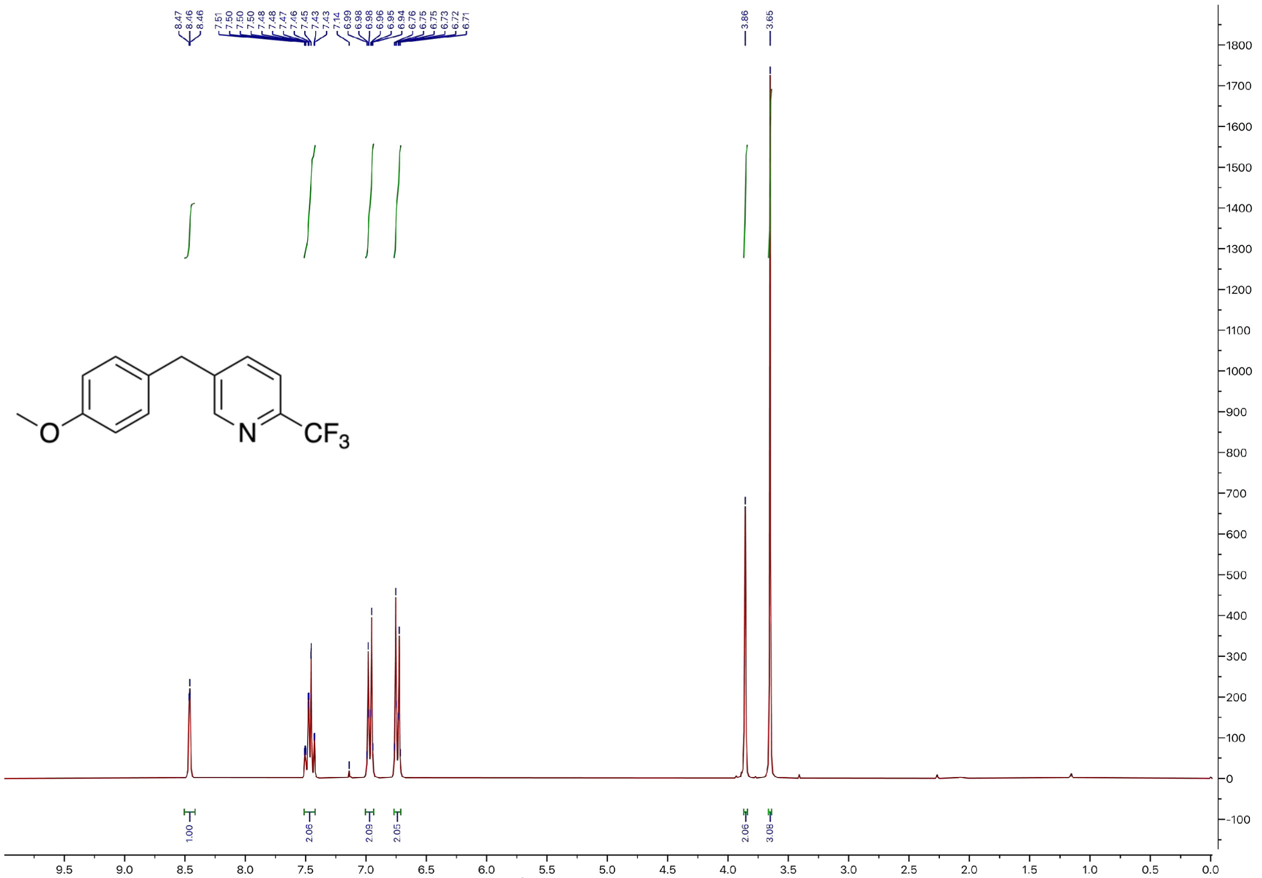

Supplementary Figure 104. ^{1}H NMR (300 MHz, $CDCl_3$) spectrum of **3al**.

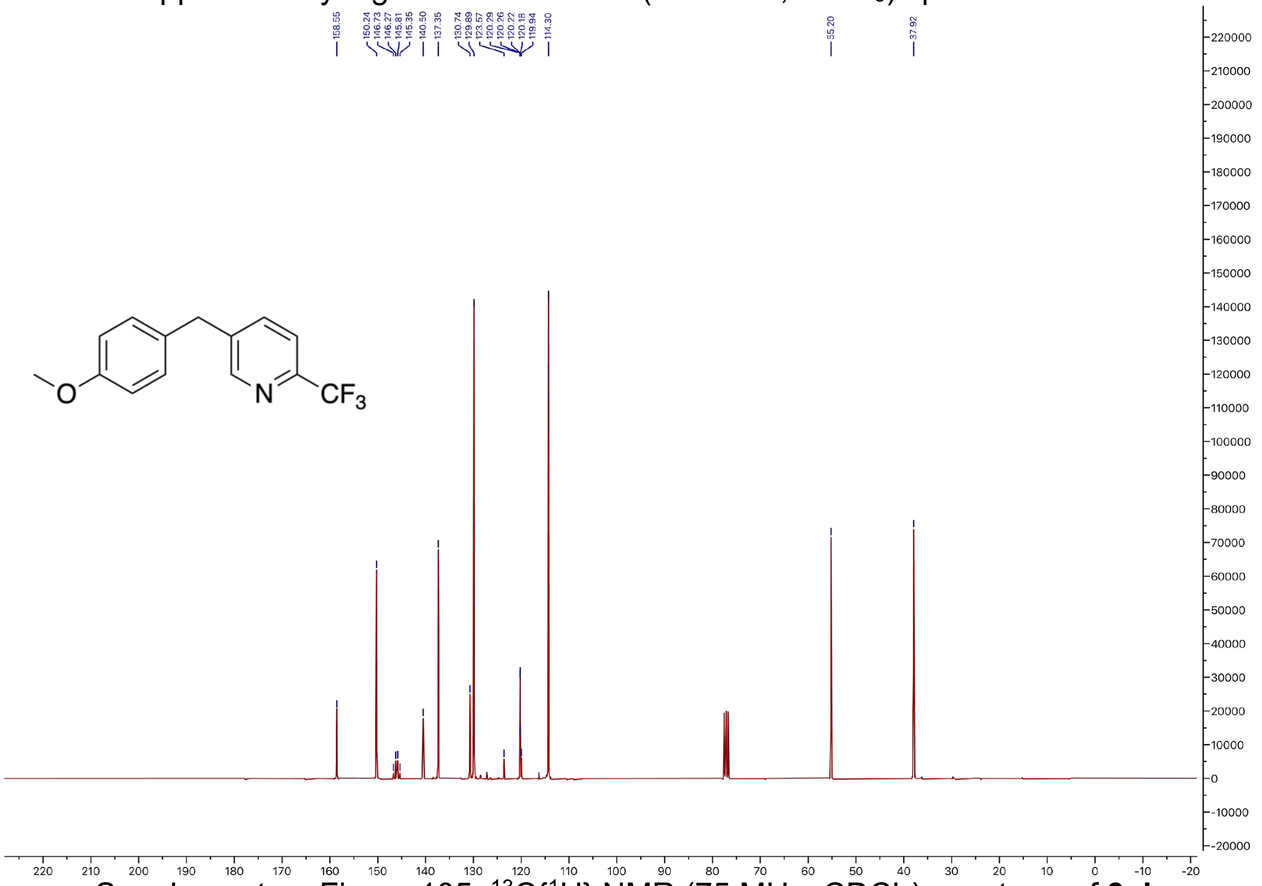

Supplementary Figure 105. $^{13}C\{^{1}H\}$ NMR (75 MHz, $CDCl_3$) spectrum of **3al**.

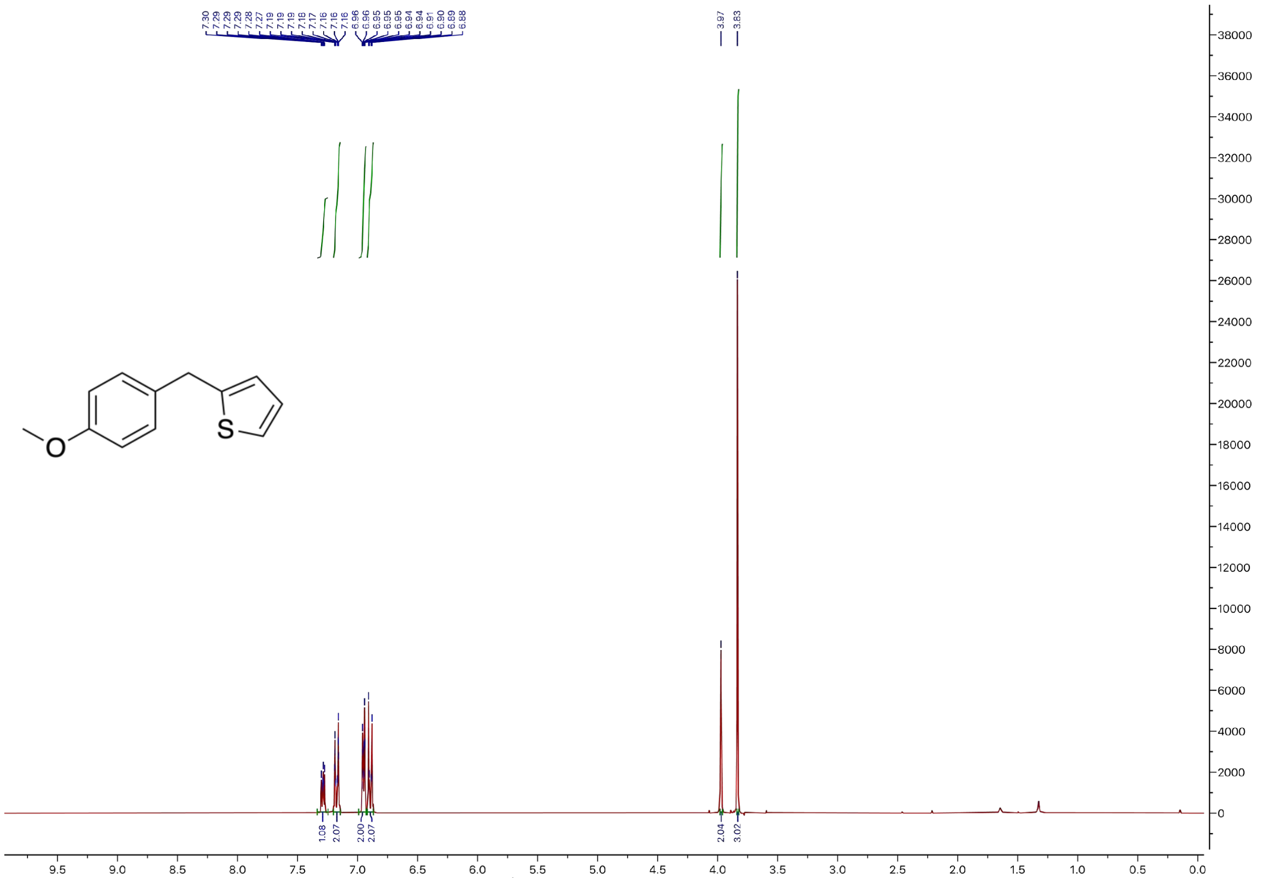

Supplementary Figure 106. ^{1}H NMR (300 MHz, $CDCl_3$) spectrum of **3am**.

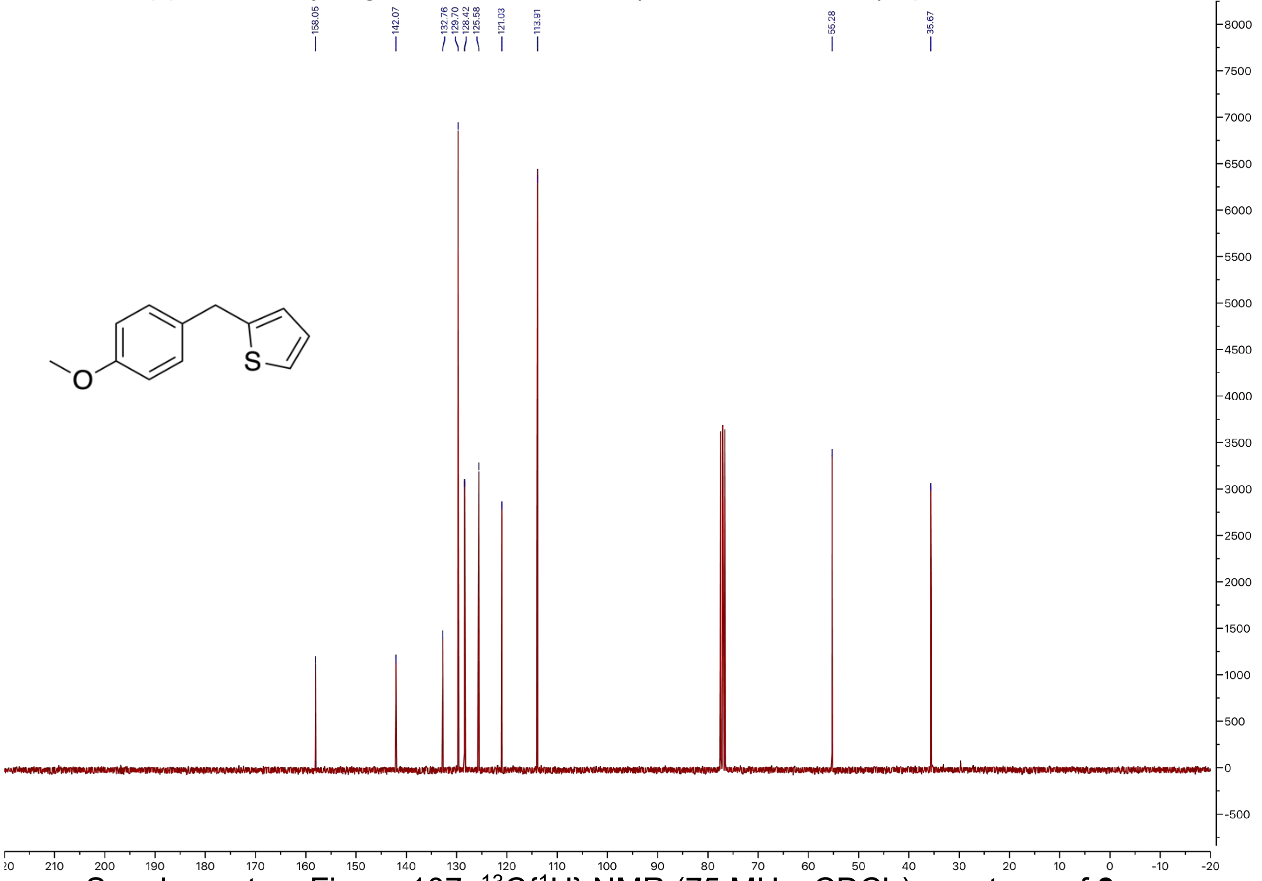

Supplementary Figure 107. $^{13}C\{^{1}H\}$ NMR (75 MHz, $CDCl_3$) spectrum of **3am**.

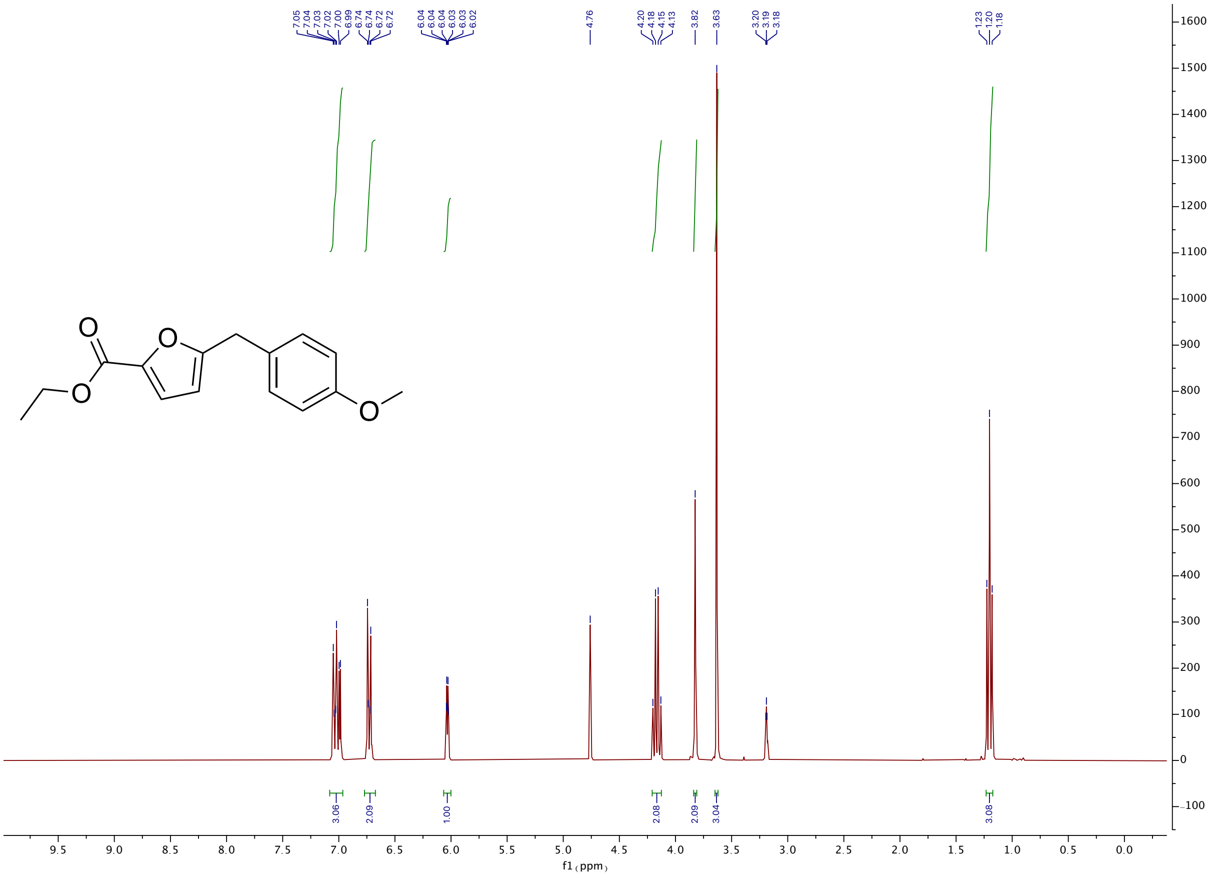

Supplementary Figure 108. ^{1}H NMR (300 MHz, MeOD) spectrum of **3an**.

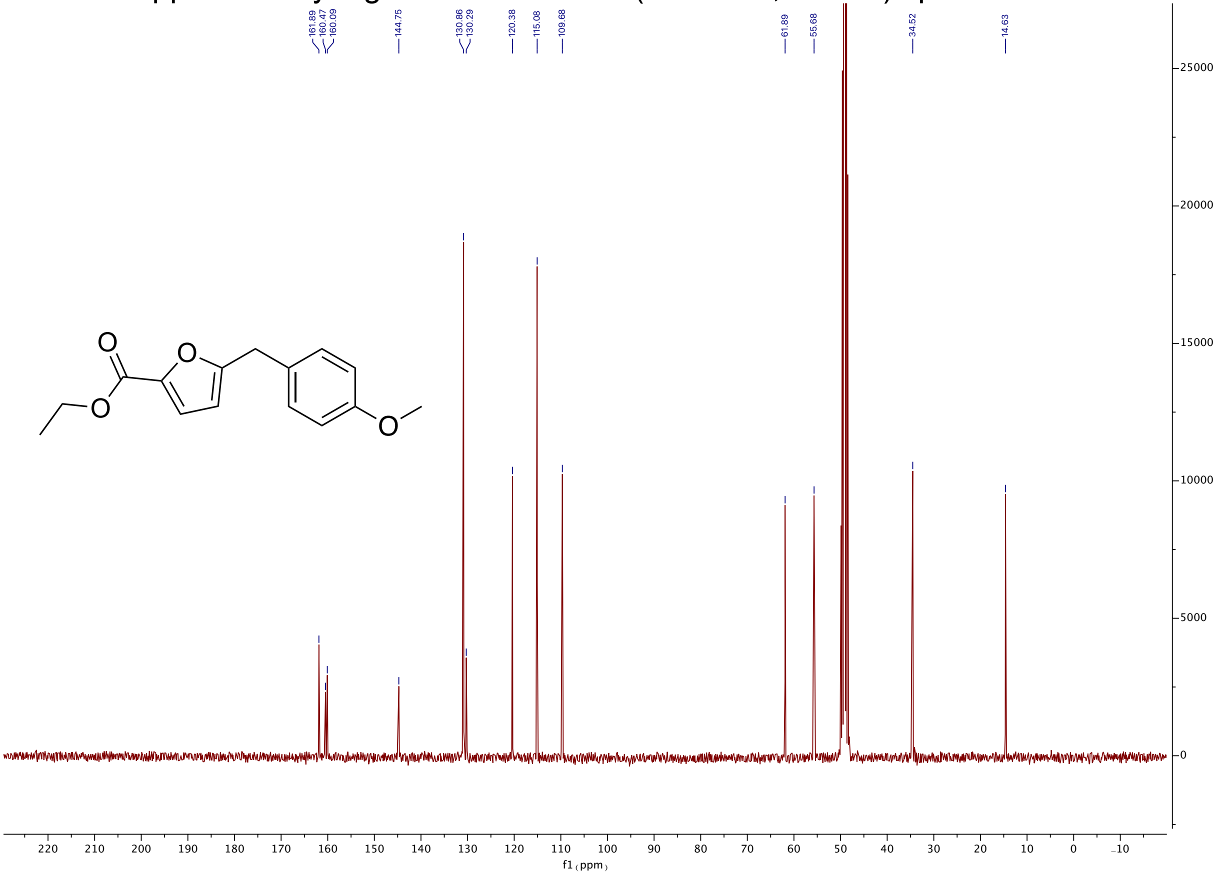

Supplementary Figure 109. $^{13}C\{^{1}H\}$ NMR (75 MHz, MeOD) spectrum of **3an**.

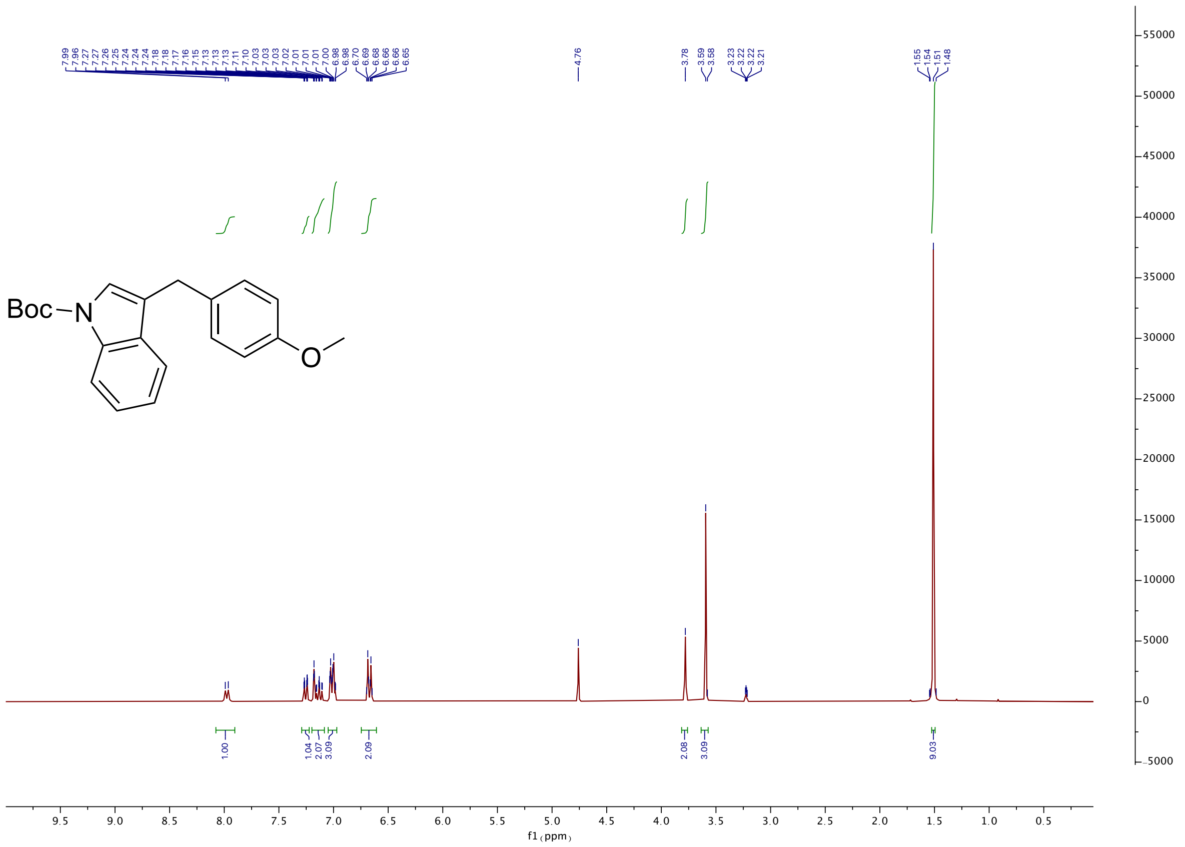

Supplementary Figure 110. ^{1}H NMR (300 MHz, MeOD) spectrum of **3ao**.

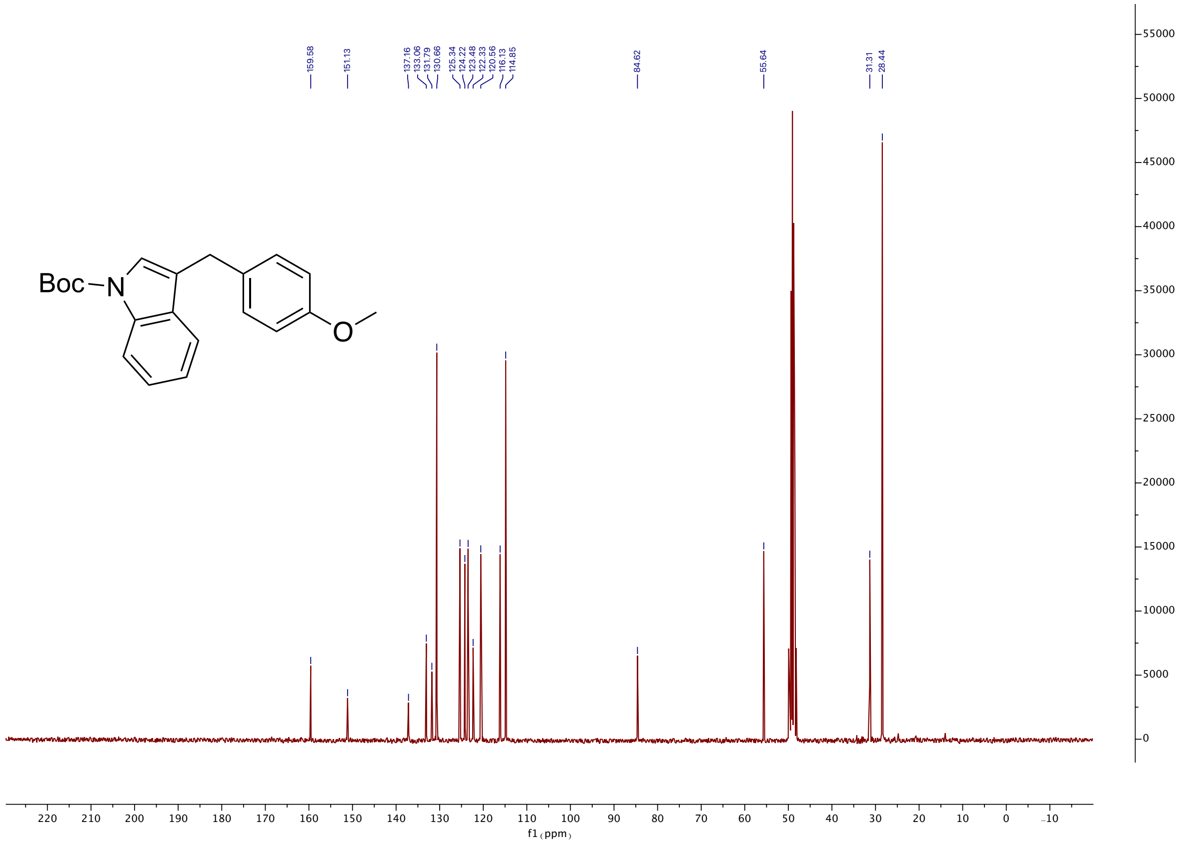

Supplementary Figure 111. ^{13}C{^{1}H} NMR (75 MHz, MeOD) spectrum of **3ao**.

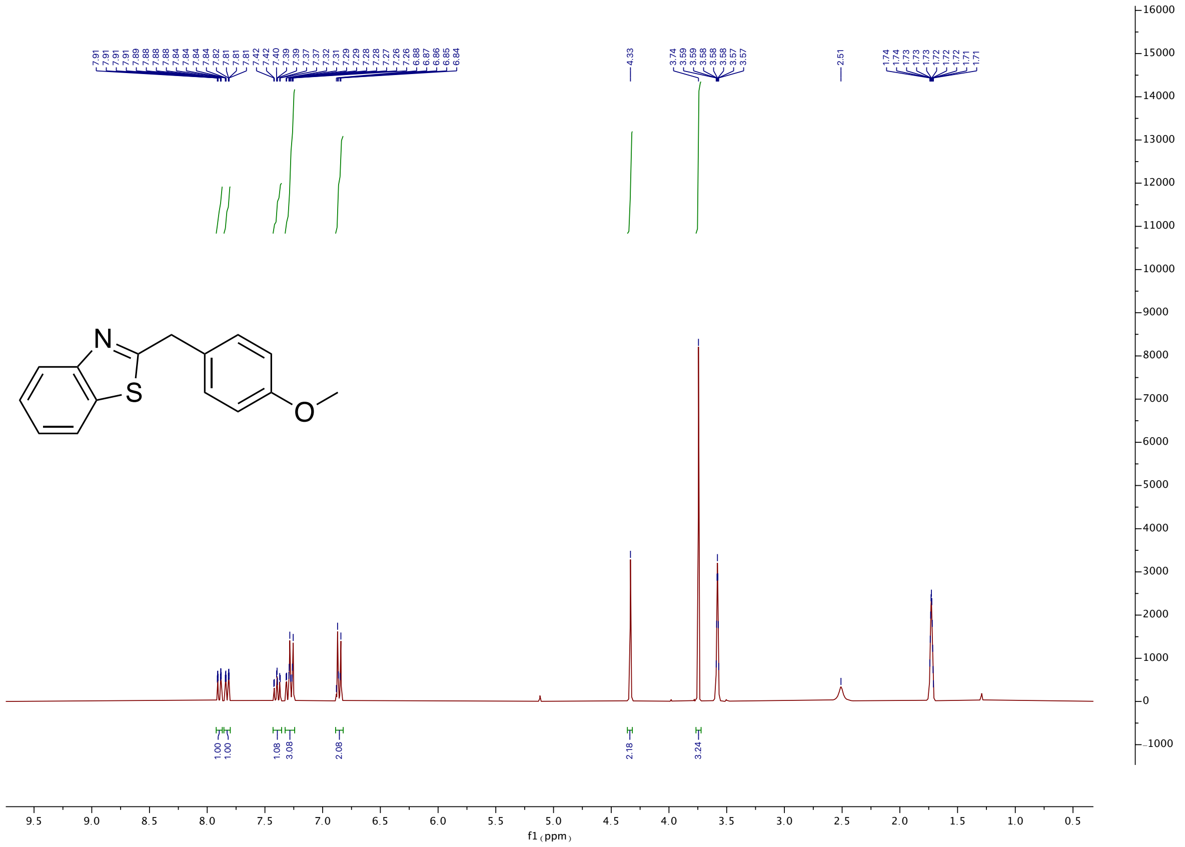

Supplementary Figure 112. ^{1}H NMR (300 MHz, THF-d_8) spectrum of **3ap**.

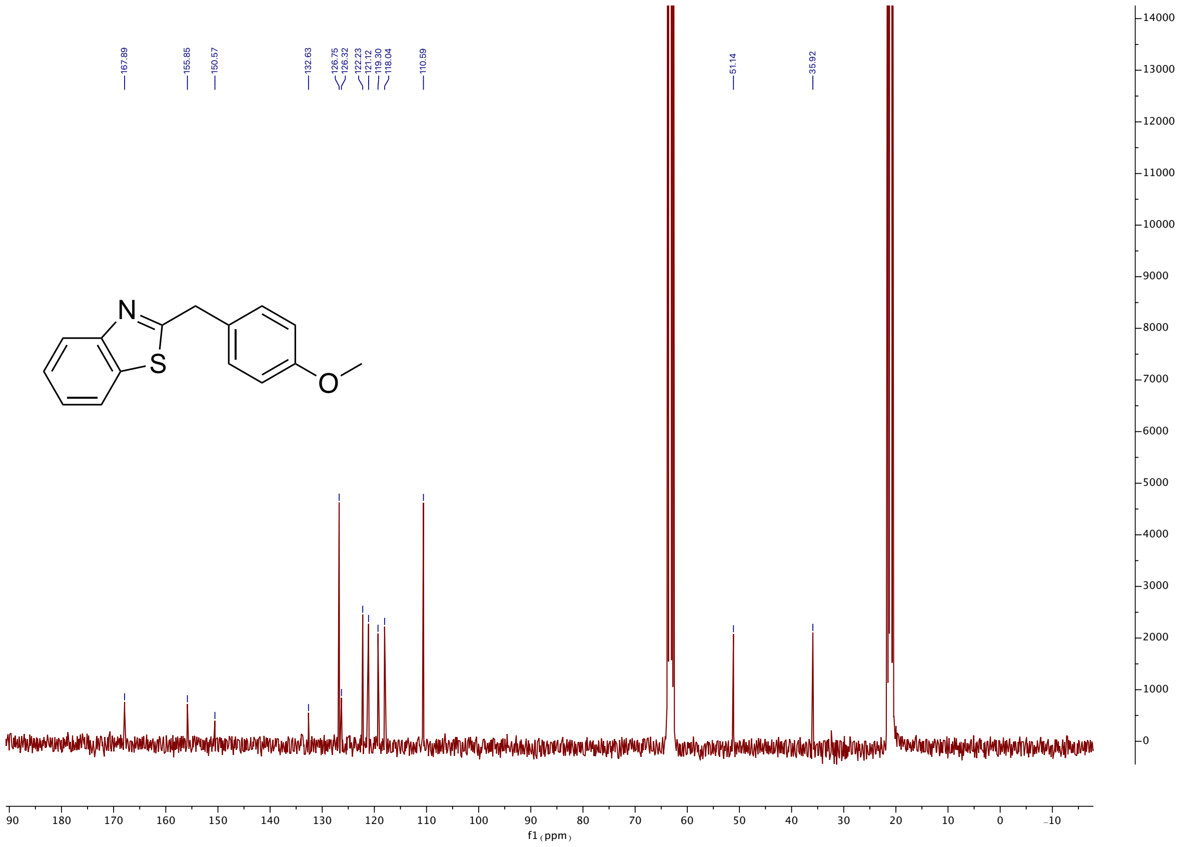

Supplementary Figure 113. $^{13}C\{^{1}H\}$ NMR (75 MHz, THF-d_8) spectrum of **3ap**.

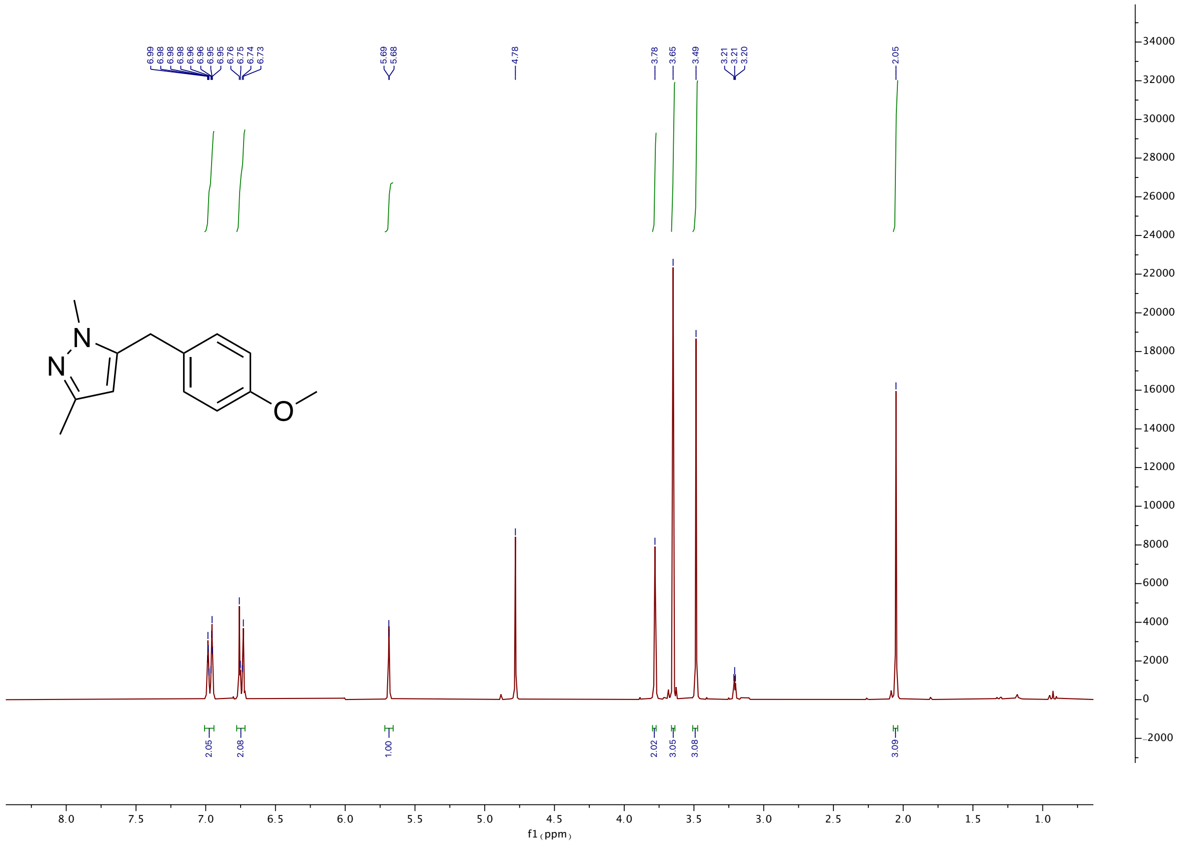

Supplementary Figure 114. ^{1}H NMR (300 MHz, MeOD) spectrum of **3aq.**

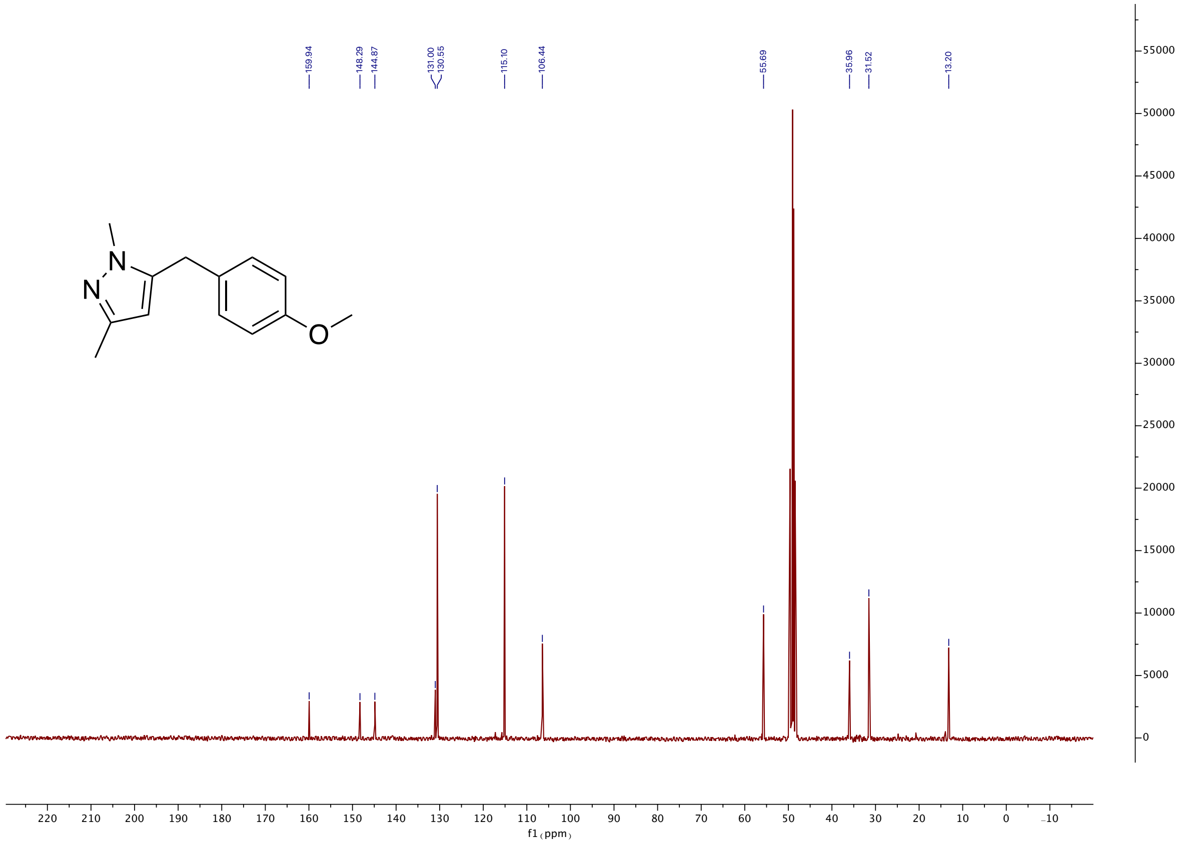

Supplementary Figure 115. $^{13}C\{^{1}H\}$ NMR (75 MHz, MeOD) spectrum of **3aq.**

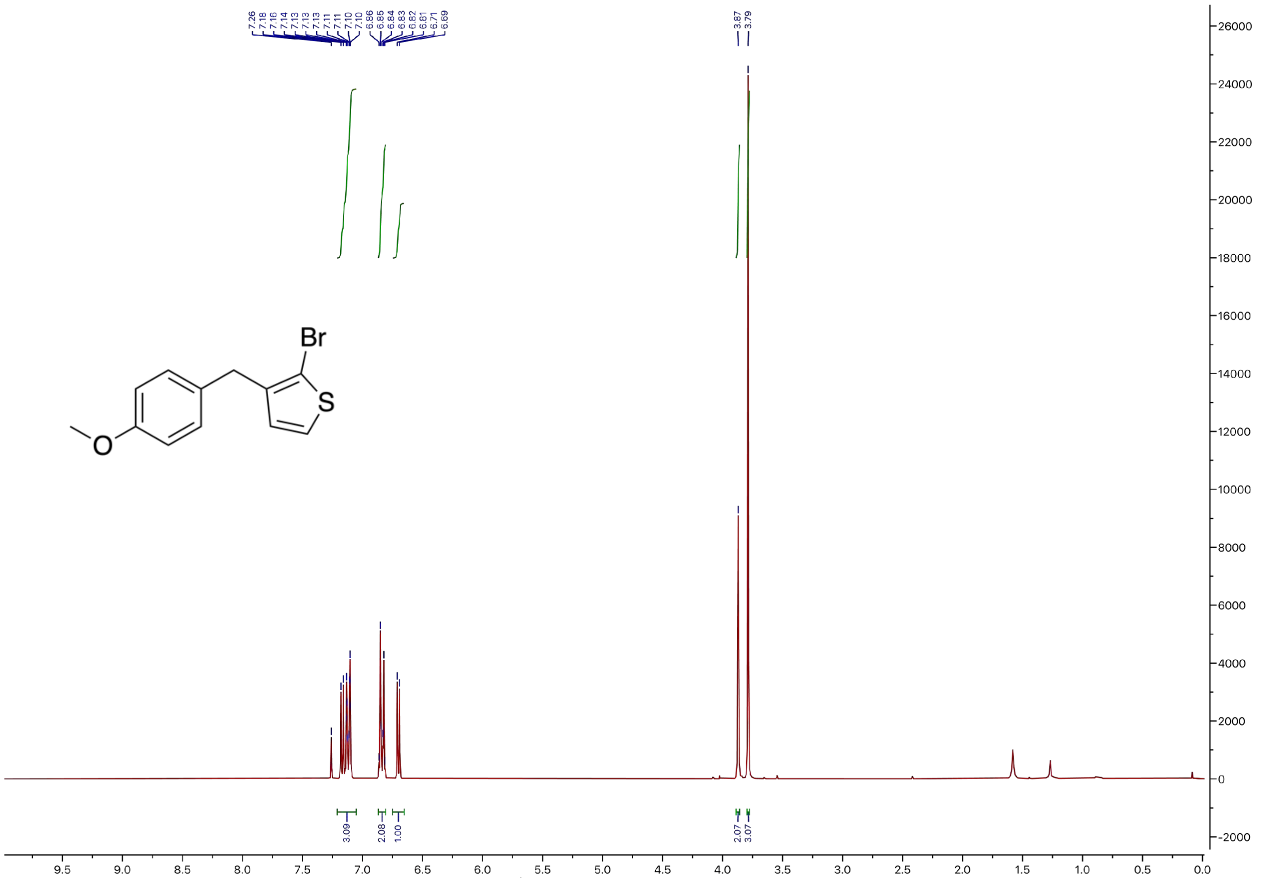

Supplementary Figure 116. ^{1}H NMR (300 MHz, $CDCl_3$) spectrum of **3ar**.

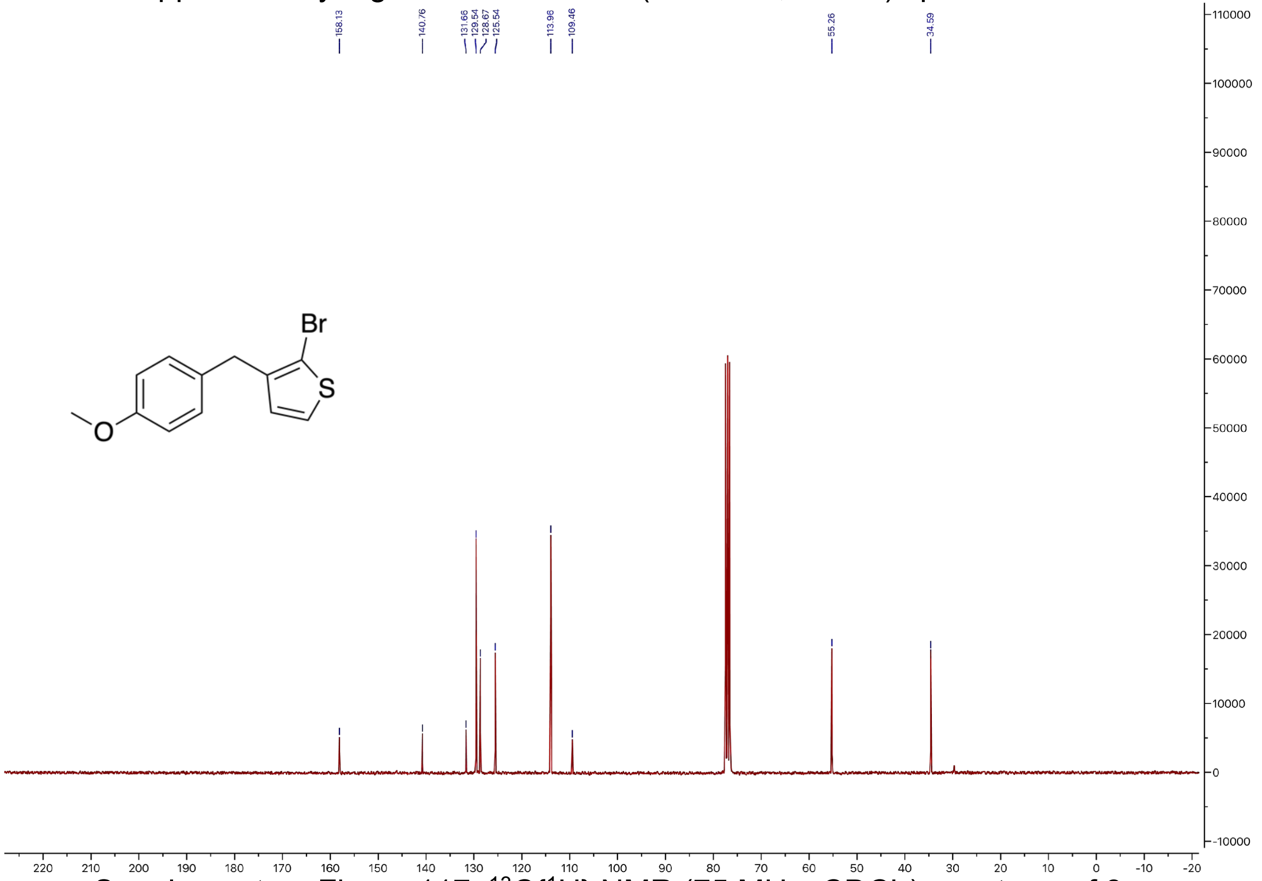

Supplementary Figure 117. $^{13}C\{^{1}H\}$ NMR (75 MHz, $CDCl_3$) spectrum of **3ar**.

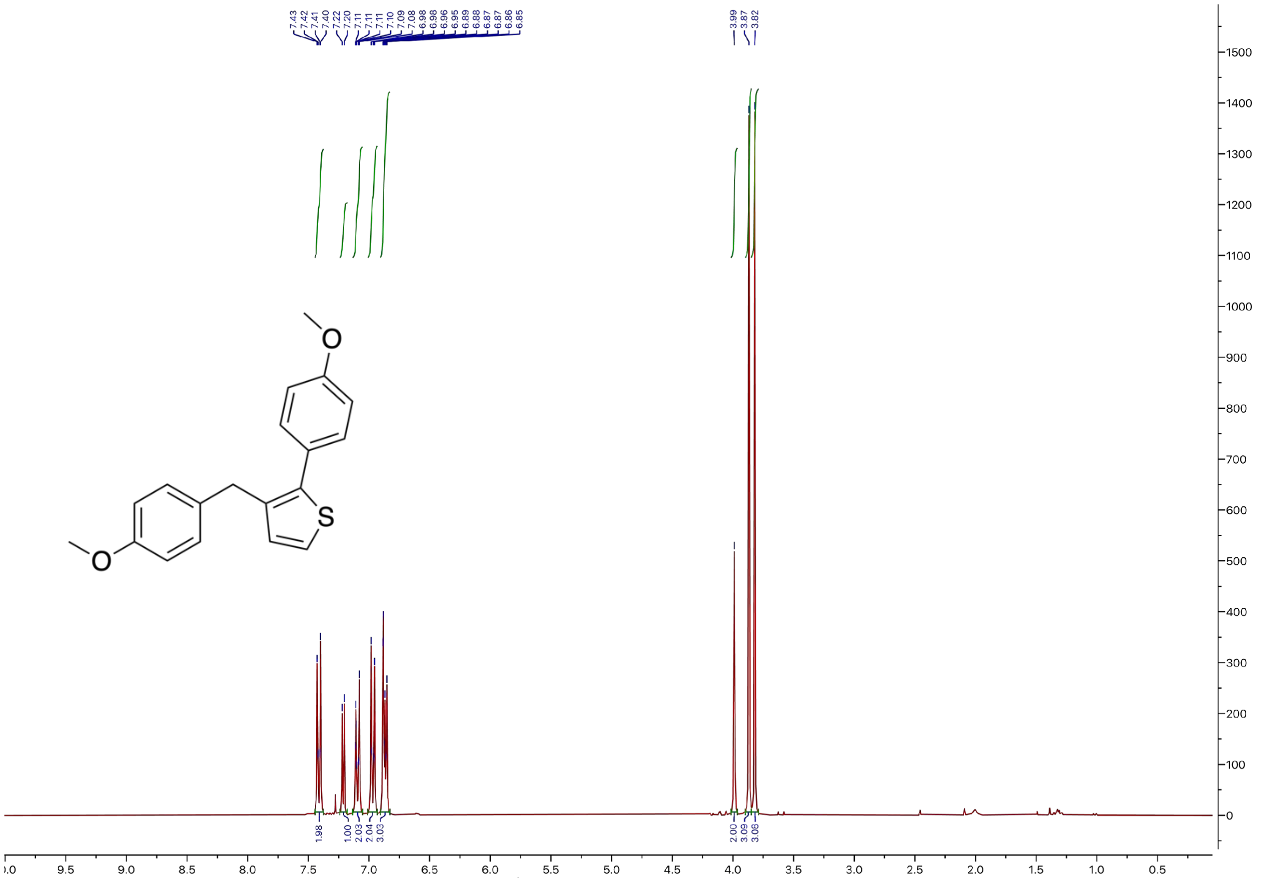

Supplementary Figure 118. ^{1}H NMR (300 MHz, $CDCl_3$) spectrum of **3as**.

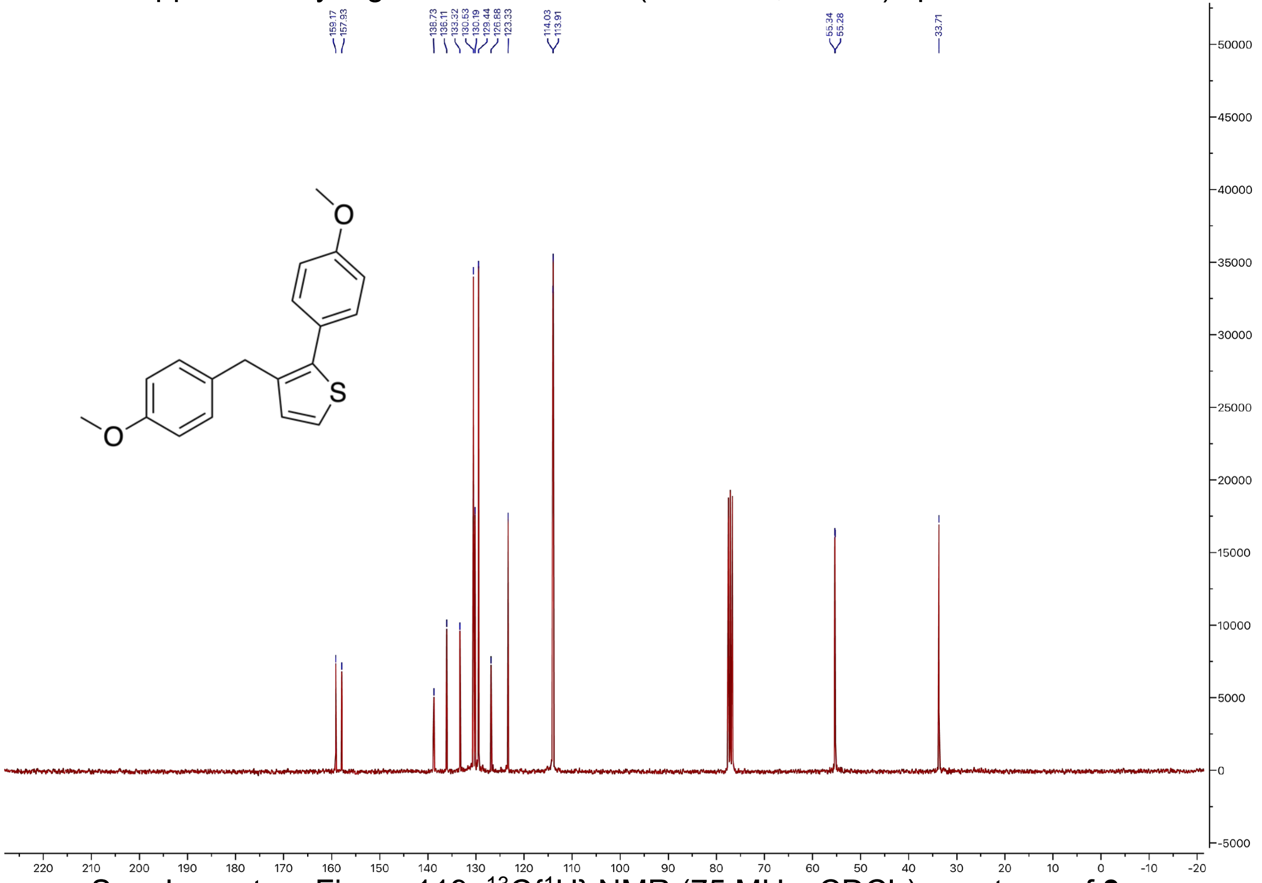

Supplementary Figure 119. $^{13}C\{^{1}H\}$ NMR (75 MHz, $CDCl_3$) spectrum of **3as**.

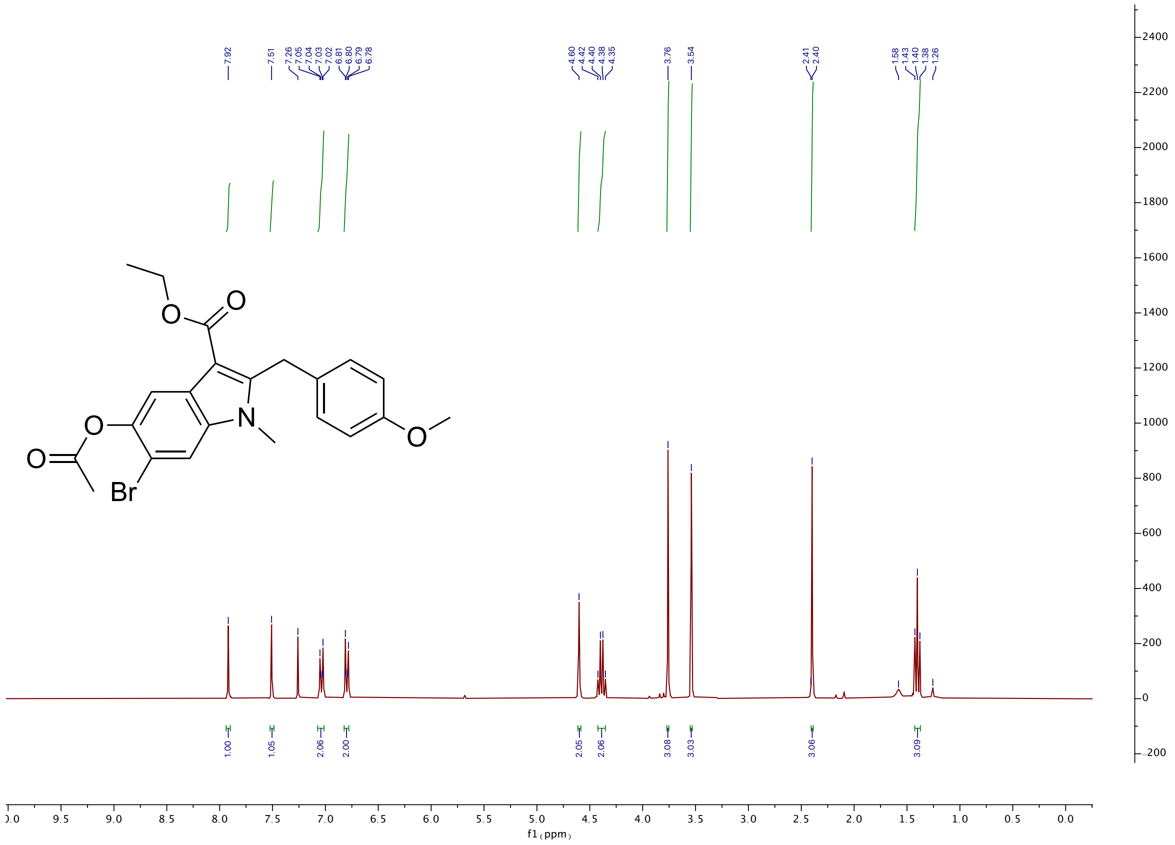

Supplementary Figure 120. ^{1}H NMR (300 MHz, $CDCl_3$) spectrum of **3at**.

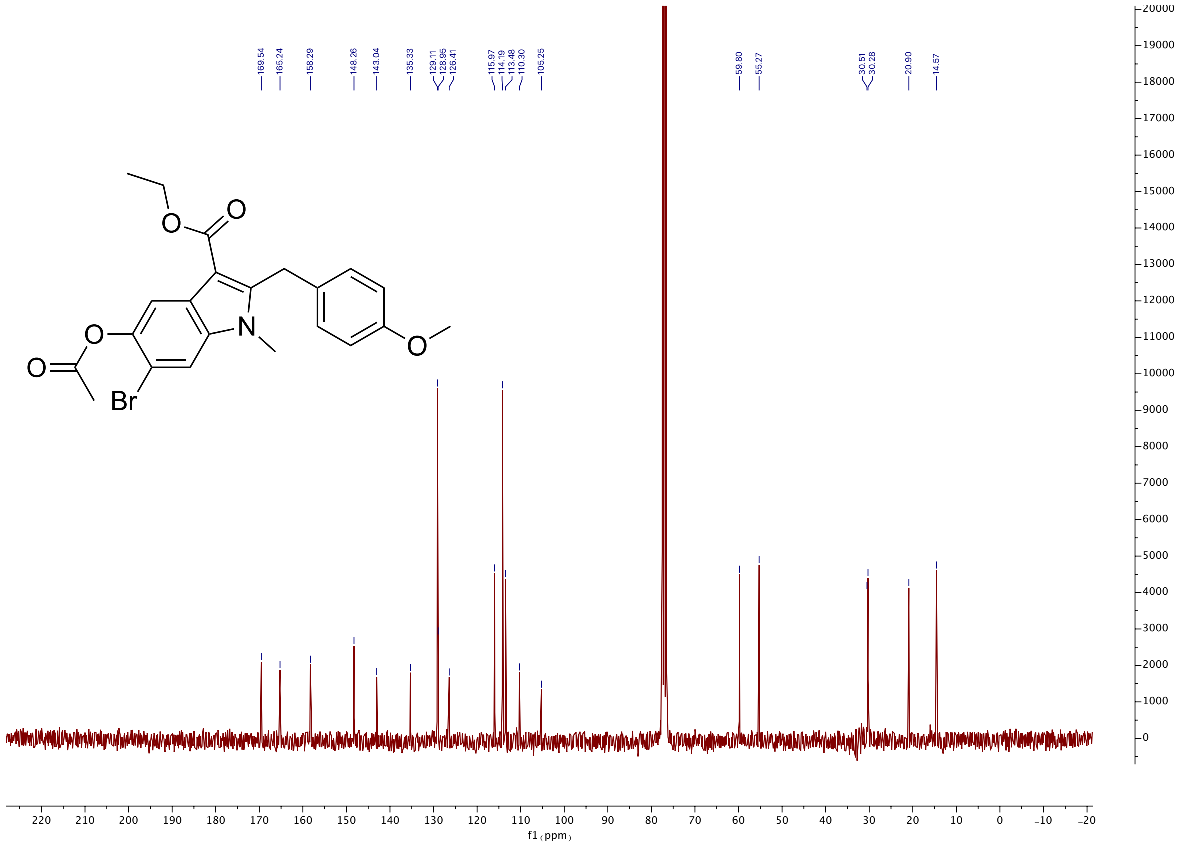

Supplementary Figure 121. $^{13}C\{^{1}H\}$ NMR (75 MHz, $CDCl_3$) spectrum of **3at**.

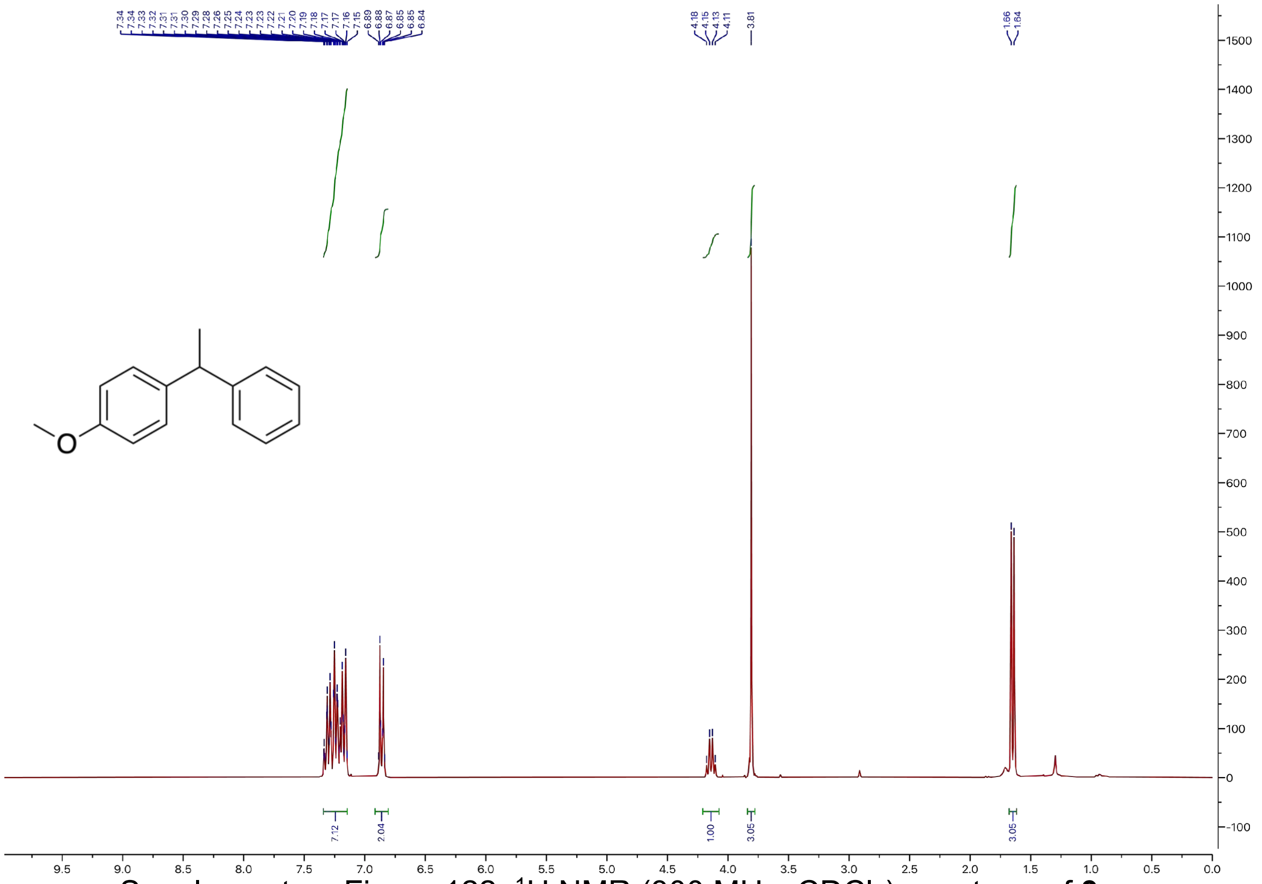

Supplementary Figure 122. ^{1}H NMR (300 MHz, $CDCl_3$) spectrum of **3au**

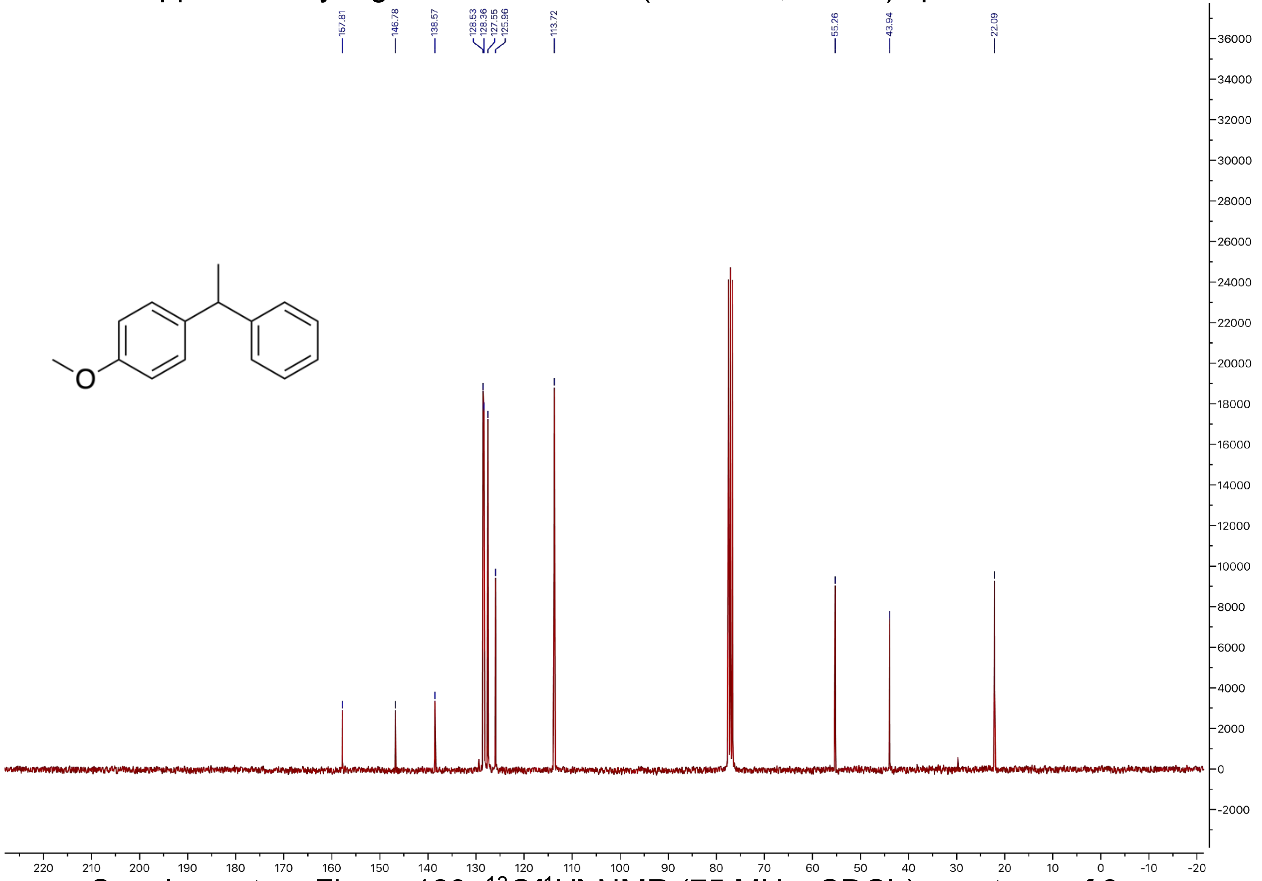

Supplementary Figure 123. $^{13}C\{^{1}H\}$ NMR (75 MHz, $CDCl_3$) spectrum of **3au**.

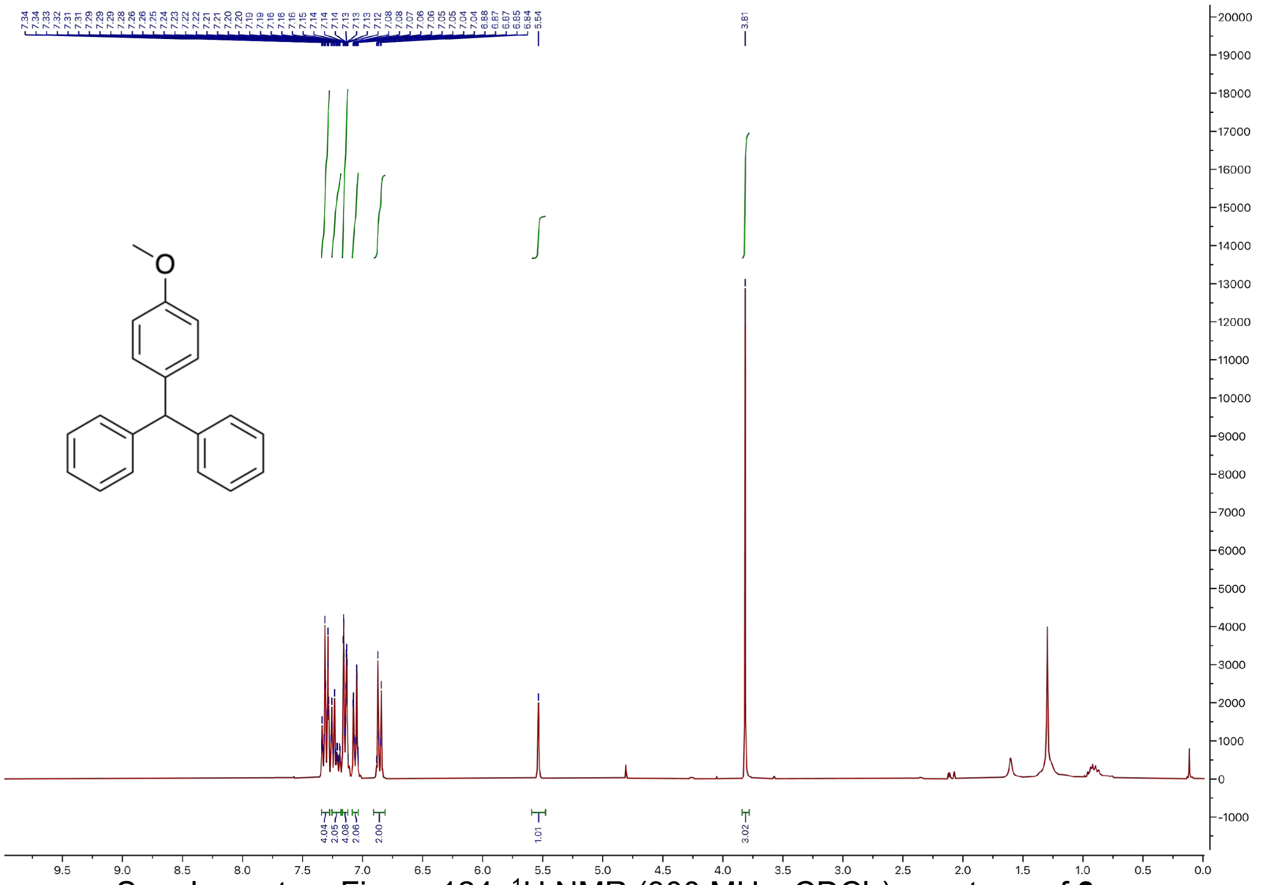

Supplementary Figure 124. ^{1}H NMR (300 MHz, $CDCl_3$) spectrum of **3av**.

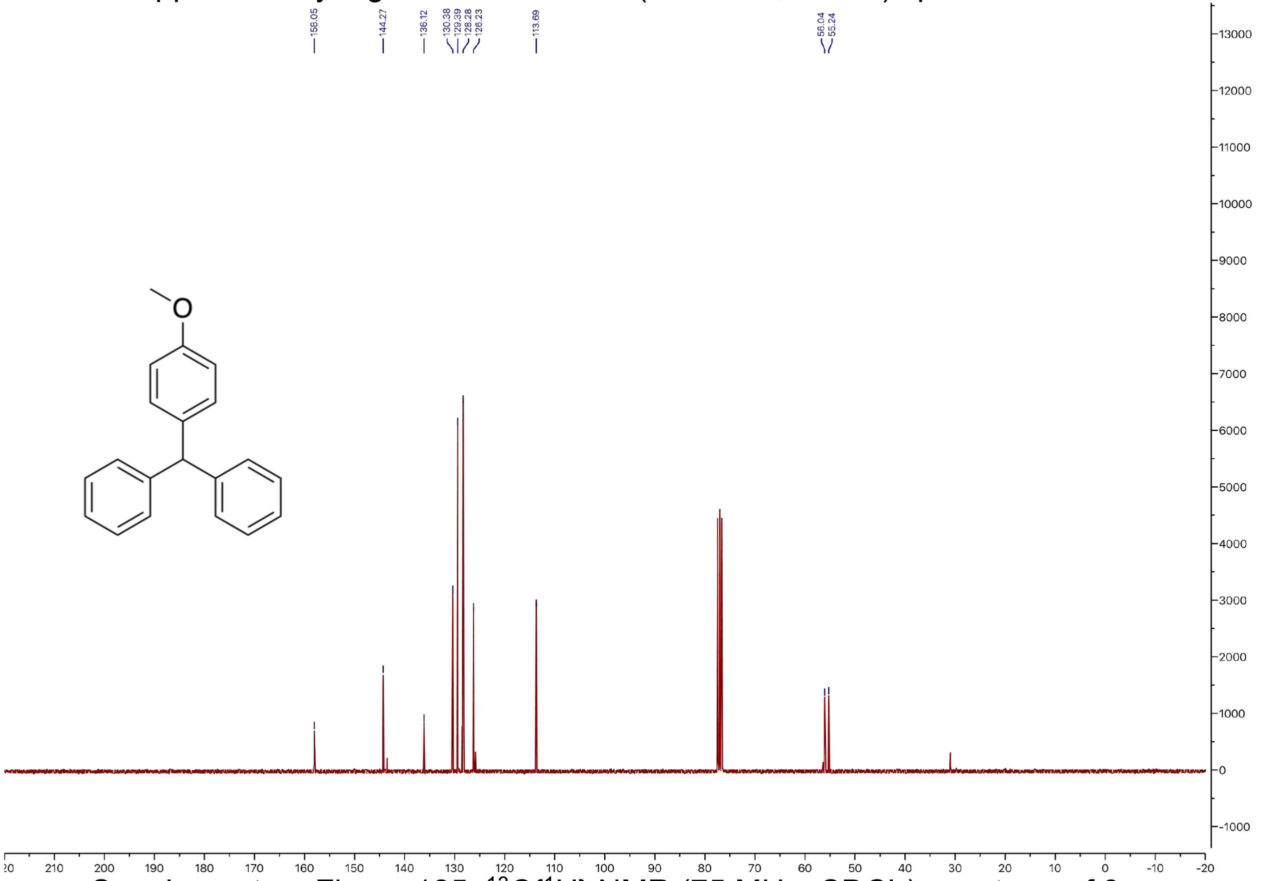

Supplementary Figure 125. $^{13}C\{^{1}H\}$ NMR (75 MHz, $CDCl_3$) spectrum of **3av**.

Supplementary References

1. Deem, M. C. *et al.* Ring walking as a regioselectivity control element in Pd-catalyzed C-N cross-coupling. *Nat. Commun.* **13,** 2869 (2022).
2. Daponte, J. A., Guo, Y., Ruck, R. T. & Hein, J. E. Using an automated monitoring platform for investigations of biphasic reactions. *ACS Catal.* **9,** 11484–11491 (2019).
3. Nielsen, C. D. T. & Burés, J. Visual kinetic analysis, Chem. *Sci* **10,** 348–353 (2019).
4. Molloy, J. J. *et al.* Chemoselective oxidation of aryl organoboron systems enabled by boronic acid-selective phase transfer. *Chem. Sci.* **8,** 1551–1559 (2017).
5. London, R. E. & Gabel, S. A. Fluorine-19 NMR studies of fluorobenzeneboronic acids. 1. interaction kinetics with biologically significant ligands. *J. Am. Chem. Soc.* **116,** 2562–2569 (1994).
6. Fuentes-Rivera, J. J., Zick, M. E., Düfert, M. A. & Milner, P. J. Overcoming halide inhibition of Suzuki–Miyaura couplings with biaryl monophosphine-based catalysts. *Org. Process Res. Dev.* **23,** 1631–1637 (2019).
7. Düfert, M. A., Billingsley, K. L. & Buchwald, S. L. Suzuki-Miyaura cross-coupling of unprotected, nitrogen-rich heterocycles: substrate scope and mechanistic investigation. *J. Am. Chem. Soc.* **135,** 12877–12885 (2013).
